# Genome-wide chromatin interaction map for *Trypanosoma cruzi*

**DOI:** 10.1038/s41564-023-01483-y

**Published:** 2023-10-12

**Authors:** Florencia Díaz-Viraqué, María Laura Chiribao, María Gabriela Libisch, Carlos Robello

**Affiliations:** 1 Laboratorio de Interacciones Hospedero–Patógeno—UBM, Institut Pasteur de Montevideo, Montevideo, Uruguay; 2 Departamento de Bioquímica, Facultad de Medicina, Universidad de la República, Montevideo, Uruguay

**Keywords:** Parasite biology, Molecular medicine

## Abstract

Trypanosomes are eukaryotic, unicellular parasites, such as *Trypanosoma brucei*, which causes sleeping sickness, and *Trypanosoma cruzi*, which causes Chagas disease. Genomes of these parasites comprise core regions and species-specific disruptive regions that encode multigene families of surface glycoproteins. Few transcriptional regulators have been identified in these parasites, and the role of spatial organization of the genome in gene expression is unclear. Here we mapped genome-wide chromatin interactions in *T. cruzi* using chromosome conformation capture (Hi-C), and we show that the core and disruptive regions form three-dimensional chromatin compartments named C and D. These chromatin compartments differ in levels of DNA methylation, nucleosome positioning and chromatin interactions, affecting genome expression dynamics. Our data reveal that the trypanosome genome is organized into chromatin-folding domains and transcription is affected by the local chromatin structure. We propose a model in which epigenetic mechanisms affect gene expression in trypanosomes.

## Abstract (web summary)

Spatial organization of the *Trypanosoma cruzi* genome and how it affects gene expression.

## Main

Gene organization, regulation of genome expression and RNA metabolism in trypanosomes differ from that in other eukaryotes. Genes are organized into directional gene clusters (DGCs) separated by strand-switch regions (SSRs), where the transcription sense converges or diverges. The DGCs are transcribed as large polycistronic transcription units (PTUs)^1^, and messenger RNA maturation occurs by co-transcriptional trans-splicing of spliced leader RNA and polyadenylation^2^. It is widely accepted that post-transcriptional regulation is the main mode of gene expression regulation^3–5^. However, recent reports have highlighted the impact of histone post-translational modifications, histone variants, nucleosome positioning, base J and chromatin organization on the regulation of gene expression, cell cycle control and differentiation^6–13^.

Epigenetic mechanisms are important for transcriptional regulation in several protozoan parasites^14,15^, including trypanosomes^8^. Recently, it was reported that specific spatial genome organization is required for the monogenic expression of the variant surface glycoprotein (VSG) in *Trypanosoma brucei*^9^, denoting the importance of the genome architecture on gene expression in trypanosomes. The surface of trypanosomes is covered with glycoproteins^16^ whose composition varies among different species, probably owing to molecular mechanisms that underpin the regulation of the expression of these genes. *T. brucei* is extracellular during completion of its life cycle and relies on antigenic variation of VSGs to evade the host immune system^17,18^. By contrast, *Trypanosoma cruzi*, which has intracellular and extracellular life-cycle stages, simultaneously expresses thousands of slightly different surface proteins to evade host immunity^19–21^.

The genomes of trypanosomes are partitioned into two large regions, formally known as compartments^22,23^. In *T. cruzi*, the core compartment (which presents synteny among trypanosomatids) is composed of conserved genes and the disruptive (loss of synteny) compartment is where most of the surface coding genes are located; in particular, the disruptive compartment is enriched in genes coding for mucins, mucin-associated surface protein and trans-sialidases^22^. In *T. brucei*, genes encoding for VSGs are also located in a particular compartment in the genome, which is located mostly in the extreme ends (subtelomeres) of the chromosomes^23^. While regions have been demarcated, the role of chromatin organization in controlling the expression of non-antigenic genes, general gene expression and pathogenesis in *T. cruzi* is still largely uncharted.

Stage-specific expression of surface proteins is essential for trypanosomes to complete their life cycles, so surface-protein-encoding genes need to be tightly regulated. Physical separation in the genome would be an efficient mechanism to regulate these genes. We hypothesized that specialized genome organization might enable gene regulation mechanisms in trypanosomes. To test this hypothesis, we analysed how the spatial organization of the genome influences gene expression in the context of previously defined genome compartments and report our results here.

## *T. cruzi* genome is organized into chromatin-folding domains

**Fig. 1:**
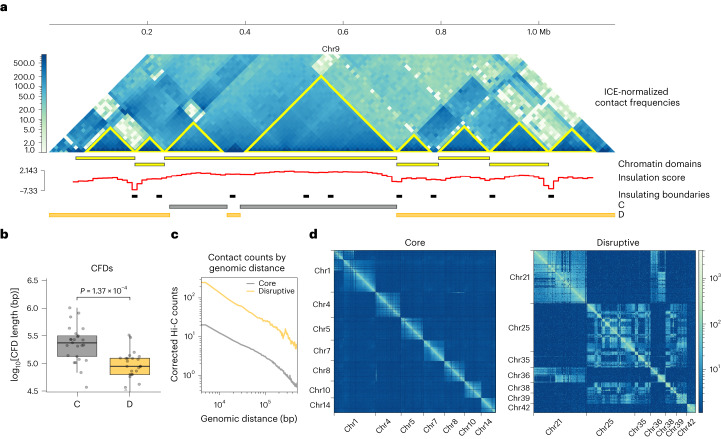
Chromatin interaction in *T. cruzi* genome compartments. **a**, Normalized Hi-C interaction frequencies of a representative chromosome (chr9) shown as a two-dimensional heatmap at 10 kb resolution. CFDs identified using HiCExplorer and TADtools (triangles on the Hi-C map) are indicated in yellow. There is a correlation between the local minimum of the insulation score calculated using FAN-C, which represents the region between two self-interacting domains, and the CFDs identified with HiCExplorer and TADtools. The genomic positions of the core and disruptive genome compartments are represented by grey and yellow bars, respectively. ICE, iterative correction and eigenvector decomposition. **b**, Box plot of CFD length, depending on whether the domain is in the core or disruptive compartment. Centre lines of the box plots represent medians, box limits represent 25th and 75th percentiles, and whiskers span minimum-to-maximum values. CFD core median length = 230 kb (*N* = 25); CFD disruptive median length = 85 kb (*N* = 24). Significance was determined using an unpaired two-sample Wilcoxon test (two-sided). **c**, Mean interaction frequencies at all genomic distances at 5 kb resolution. For the analysis, chromosomes were classified into core (>80% core compartment), disruptive (>80% disruptive compartment) or mixed (Supplementary Table 2). **d**, Intra- and inter-chromosomal interactions between core and disruptive chromosomes.

**Extended Data Fig. 1:**
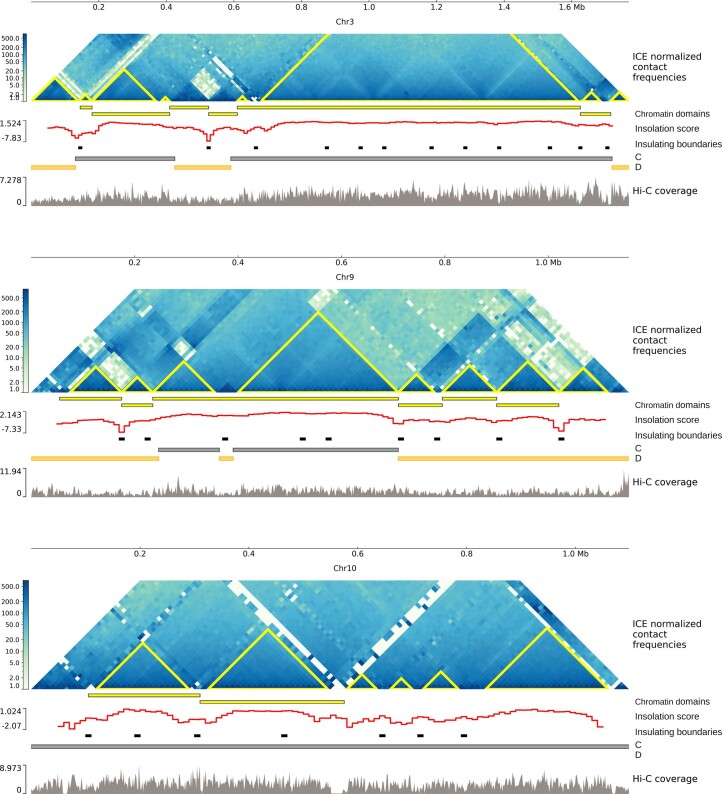
Normalized Hi-C interaction frequencies of chromosomes 3, 9 and 10. Results are displayed as a two-dimensional heatmap at 10 kb resolution. Chromatin Folding Domains (CFDs) identified using HiCExplorer and TADtools (triangles on Hi-C map) are indicated in yellow. The local minimum insulation score calculated using FAN-C (representing the region between two self-interacting domains) is indicated as a black dash. The genomic position of core and disruptive genome compartments are represented in grey and yellow, respectively. Hi-C coverage is plotted.

On the basis of our previous observation that the *T. cruzi* genome is partitioned into compartments^22^, we wondered whether this linear organization correlates with three-dimensional conformation. To characterize chromatin conformation in *T. cruzi* cells, we mapped genome-wide interactions by analysing chromosome conformation capture (Hi-C) data that were previously used for genome assembly^24^. The analysis of interaction matrices at various bin sizes revealed that there are several regions in close spatial proximity indicating the presence of chromatin-folding conformations (Fig. 1a). To identify all topological domains, we explored the formation of chromatin-folding domains (CFDs) with three different algorithms (Supplementary Table 1 and Extended Data Fig. 1). As in other organisms^25^, the structural domains showed hierarchical organization, suggesting multiple levels of genome organization. Using a Hi-C map with 5 kb resolution, we identified 173 domains with 129 kb mean size, occupying 60% of the genome. We noticed that a considerable fraction of the genome remains structured in these folding domains, representing a prominent feature of genome organization. Notably, the structures we identified are smaller than topologically associating domains (TADs) usually described in other eukaryotes^26^. TAD predictor algorithms failed to detect domains in the smallest chromosomes (<1.1 Mb) and at common resolutions for TAD identification. This result is reasonable as the chromosomes of *T. cruzi* are smaller (<3 Mb) and exhibit reduced non-coding regions (for example, lack of introns, and short intergenic and inter-DGC regions). In addition, the concept of TADs has been mainly developed for complex eukaryotes, mainly mammals^27,28^. For these reasons, we refer to these structures as CFDs. Both compartments contain CFDs, and remarkably, 80% of them belong to only one compartment, indicating that CFDs in each compartment are mutually exclusive (Supplementary Table 1). Therefore, the previously defined linear genome compartments^22^ correspond to three-dimensional compartments of the nucleus. From here, core and disruptive will refer to the linear organization of the trypanosome genome and C and D to the three-dimensional compartments. The D compartment exhibits smaller CFDs (almost half the size of those in the C compartment) (Fig. 1b) and a higher frequency of interactions at different distances (Fig. 1c). Examining chromosomal interactions, we found that the D compartment presents several inter-chromosomal contacts, while in the C compartment, interactions are predominantly intra-chromosomal (Fig. 1d).

## Genome regions have different patterns of gene expression

**Fig. 2:**
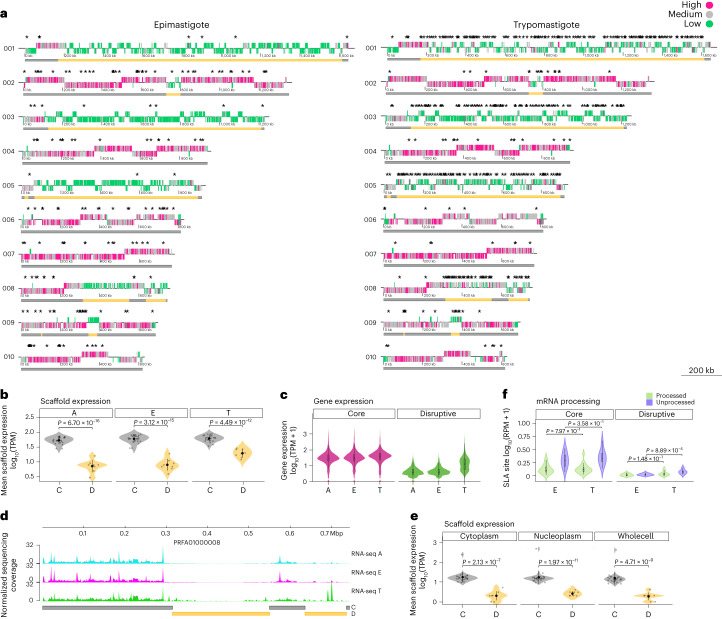
Transcriptional heterogeneity in the genomic compartments of *T. cruzi*. **a**, Genomic distribution of genes classified according to the number of transcripts in the largest ten scaffolds. Core and disruptive genomic compartments are indicated by grey and yellow bars, respectively. Differentially expressed genes are indicated by asterisks. **b**, Mean expression of scaffolds composed of core (*N* = 24) and disruptive (*N* = 8) compartments. Scaffolds were classified as core or disruptive when one of the genome compartments spans 80–100% of the length of the scaffold. Scaffolds containing the core compartment present a greater mean expression than the scaffolds defined as disruptive. Data are presented as mean ± s.d. *P* values are from the unpaired Welch two-sample *t*-test (two-sided). **c**, RNA expression of core and disruptive genes at the different stages of the parasite life cycle. Centre lines of the box plots represent medians, box limits represent 25th and 75th percentiles and whiskers span minimum-to-maximum values. The bimodality coefficient obtained for expression data of disruptive genes in trypomastigotes was 0.678. Values larger than 0.555 indicate the bimodality of data^62^. The mean expression (log_10_(TPM)) of core genes (*N* = 11,947) is 1.41, 1.43 and 1.49 in amastigotes (A), epimastigotes (E) and trypomastigotes (T), respectively. The mean expression (log_10_(TPM)) of disruptive genes (*N* = 2,991) is 0.59, 0.64 and 1.17 in A, E and T, respectively. **d**, Representative coverage plot normalized using CPM. Bin size, 10 bp. For each condition, two biological replicates are plotted. The genomic position of core and disruptive genome compartments are represented by grey and yellow bars, respectively. **e**, Mean scaffold expression of core (*N* = 24) and disruptive (*N* = 8) scaffolds using transcriptomic data of different subcellular compartments. Data are presented as mean ± s.d. *P* values are from the unpaired Welch two-sample *t*-test (two-sided). **f**, Quantification of the processed and unprocessed transcripts from RNA-seq reads covering the SLA site. RPM, reads per million. Centre lines of the box plots represent medians, box limits represent 25th and 75th percentiles and whiskers span minimum-to-maximum values. SLA regions with more reads were selected for the analysis (*N* = 10 core and *N* = 6 disruptive). The *P* values are from the Wilcoxon test (two-sided) for the mean difference in all cases.

**Extended Data Fig. 2:**
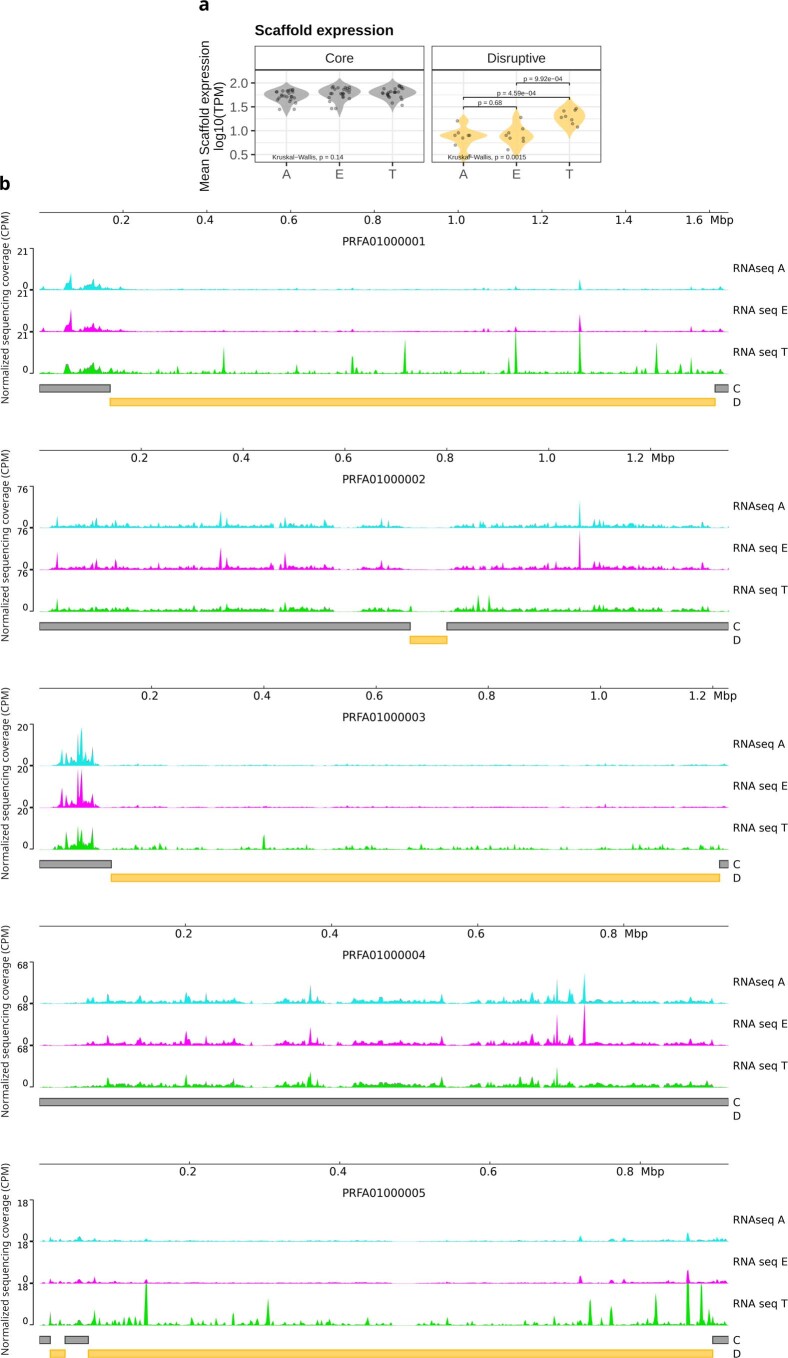
RNA expression. **a**, Mean scaffold expression of scaffold composed by core or disruptive compartment in amastigotes (A), epimastigotes (E) and trypomastigotes (T). Scaffolds were classified as core or disruptive when one of the genome compartments spans 80–100% length of the scaffold. Scaffolds containing the core compartment present similar mean expressions in the different stages while the mean scaffold expression increase in T in the disruptive scaffolds. Values are *p* value from Welch Two Sample *t*-test (two-sided). **b**, Representative RNA-seq sequencing coverage plots in entire scaffolds normalized using counts per million (CPM). Bin size 10 bp. For each condition two biological replicates are plotted.

To explore the influence of the three-dimensional genome organization on global gene expression, we performed RNA sequencing (RNA-seq) of different stages of the parasite, and we classified the genes into high-, medium- and low-expressed genes according to the number of transcripts detected. We found that the core compartment predominantly consists of moderate- and high-expressed genes in all the stages analysed. By contrast, the disruptive compartment, which in some cases comprises almost entire chromosomes, is enriched in low-expressed genes or genes with undetectable RNA levels (Fig. 2a). These differences are statistically significant in all the stages (Fig. 2b). Moreover, the core compartment exhibits similar levels of expression whereas the mean expression of the disruptive compartment increases in trypomastigotes (Extended Data Fig. 2a). We also evaluated the RNA levels of individual genes, and we observed that while core genes are expressed in similar proportions in all stages, the disruptive genes show lower overall expression (Fig. 2c,d and Extended Data Fig. 2b) and bimodal distribution in the trypomastigote stage, in which a discrete number of genes increase their expression (Fig. 2d and Extended Data Fig. 2b). Similar differences in expression between core and disruptive compartments were also obtained with the Brazil A4 strain (Supplementary Fig. 1). Thus, these differences in gene expression between genomic compartments are characteristic of *T. cruzi* and are not strain specific.

Regarding differentially expressed genes (DEGs), we found that 94% of genes enriched in epimastigotes belong to the core compartment, while in trypomastigotes, 20% of DEGs are in the core and 80% are in the disruptive compartment (Supplementary Table 3). Thereby, the core compartment presents stage-specific genes in both stages, whereas the disruptive compartment is characteristic of the trypomastigote stage, where long stretches of the genome concentrate many DEGs (Fig. 2a).

Next, we investigated whether these global differences of expression in the compartments are due to nuclear mechanisms of regulation or are mainly controlled by post-transcriptional mechanisms, generally cytoplasmic. To this end, we compared the expression of the compartments using cytoplasmic and nuclear transcriptomes^29^, and we found that the expression differences can already be observed in the nucleus (Fig. 2e and Supplementary Fig. 2). To further explore these results, we examined nascent mRNAs, by measuring unprocessed transcripts from RNA-seq reads spanning the spliced leader acceptor (SLA) site, allowing the quantification of nascent or immature transcripts^30^. We found similar levels of unprocessed transcripts corresponding to the core compartment in both epimastigotes and trypomastigotes and a reduction in the number of unprocessed transcripts from the disruptive compartment in epimastigotes, indicative of a reduced transcription or RNA maturation (Fig. 2f and Supplementary Fig. 2). Taken together, these results confirm that the reduced expression of the disruptive compartment is a consequence of nuclear mechanisms of regulation.

## Well-positioned nucleosomes are enriched in the disruptive compartment

**Fig. 3:**
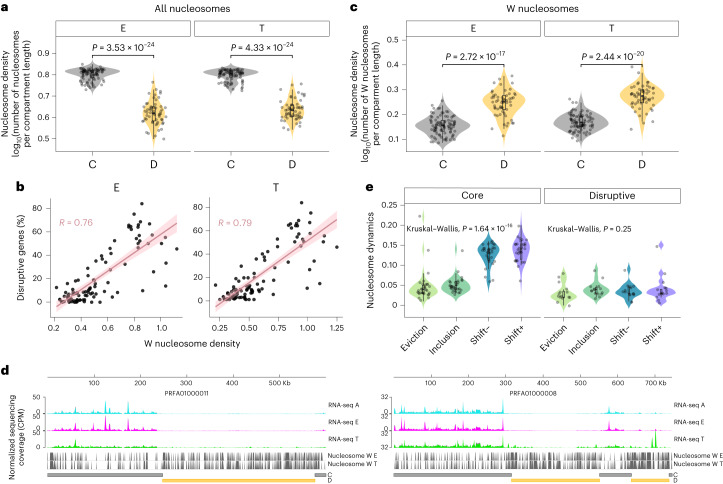
Nucleosome positioning in the genomic compartments of *T. cruzi*. **a**, Nucleosome occupancy in different regions of the genome. Centre lines of the box plots represent medians, box limits represent 25th and 75th percentiles and whiskers span minimum-to-maximum values. The difference between epimastigotes and trypomastigotes was not statistically significant (Mann–Whitney test, two-sided), neither in the core nor in the disruptive compartment. Each point represents the density of an analysed region: core (*N* = 90) or disruptive (*N* = 57). **b**, Correlation between the density of well-positioned nucleosomes and the percentage of disruptive genes in the scaffold. A linear trend line (linear model) is shown with 0.95 confidence interval. **c**, Well-positioned nucleosomes in the genome compartments (*N* = 90 core and *N* = 57 disruptive). Centre lines of the box plots represent medians, box limits represent 25th and 75th percentiles and whiskers span minimum-to-maximum values. Values above denote *P* values from the Mann–Whitney test (two-sided) to compare samples. **d**, Representative coverage plot normalized using CPM. Bin size, 10 bp. For each condition, two biological replicates are plotted. The positions of well-positioned nucleosomes in E and T are indicated by grey lines. The genomic positions of core and disruptive genome compartments are represented by grey and yellow bars, respectively. **e**, Nucleosome dynamics between epimastigote and trypomastigote. The Nucleosome Dynamics algorithm was used to detect changes (shifts, evictions and insertions) in nucleosome architectures. The number of changes detected was normalized for the length of the core or the disruptive region analysed (*N* = 120 core and *N* = 60 disruptive). Centre lines of the box plots represent medians, box limits represent 25th and 75th percentiles and whiskers span minimum-to-maximum values.

To determine whether the nucleosome landscape correlates with the transcriptional state, we assessed nucleosome positioning using micrococcal nuclease sequencing (MNase–seq) data^7^ from epimastigotes and trypomastigotes. In concordance with previous reports^8^, the core compartment exhibits a higher density of nucleosome occupancy in both stages (Fig. 3a). However, we identified a correlation between the percentage of disruptive genes in a scaffold and the density of well-positioned nucleosomes (Fig. 3b). Notably, the disruptive compartment exhibited double the density of well-positioned nucleosomes compared with the core compartment (Fig. 3c,d). To characterize the dynamic organization of nucleosomes, we compared nucleosome architecture local changes between epimastigotes and trypomastigotes. We found that the changes in the nucleosome map were more prominent in the core compartment (Fig. 3e); there were few differences in the presence and absence of nucleosomes between stages, and nucleosome shifting is the most abundant alteration. On the contrary, regarding nucleosome movement, few changes were detected in the disruptive compartment, consistent with more static regions.

## Genome-wide map of 5mC and 6mA marks in *T. cruzi*

**Fig. 4:**
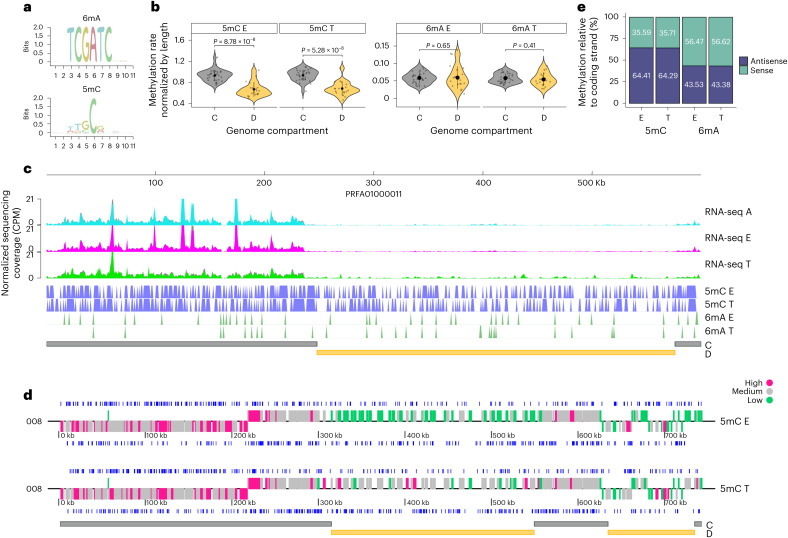
DNA methylation in the *T. cruzi* genome. **a**, Sequence logo of all methylated 11-mer sequences. **b**, Analysis of DNA methylation marks in the genome compartments. Significance was determined using the unpaired two-sample Wilcoxon test (two-sided). Data are presented as mean ± s.d. Core (*N* = 30) and disruptive (*N* = 19) regions were analysed. **c**, Distribution of 5mC and 6mA marks in a representative chromosome (PRFA01000011). RNA expression coverage plots were normalized using CPM. Bin size, 10 bp. For each condition, two biological replicates are plotted. The genomic positions of core and disruptive genome compartments are indicated by grey and yellow bars, respectively. **d**, Distribution of 5mC and 6mA marks on both DNA strands in a representative chromosome (PRFA01000008). Genes were classified according to the number of transcripts detected. The genomic positions of core and disruptive compartments are indicated by grey and yellow bars, respectively. **e**, Quantification of DNA methylation marks relative to the coding sequence.

As DNA methylation is a crucial determinant of spatial chromatin organization and gene expression^31^, we compared the genomic distribution of 5mC and 6mA DNA methylation marks in the infective and non-infective stages of *T. cruzi*. We found 6mA at low levels (0.04% of all adenines are methylated) both in epimastigotes and trypomastigotes and present at the TCG6mATC motif (Fig. 4a). In contrast, 5mC is the predominant DNA methylation mark we found—0.75% and 0.77% of all cytosines are methylated in epimastigotes and trypomastigotes, respectively—with a marked difference in distribution between compartments—a higher percentage of 5mC is present in the core in both stages (Fig. 4b,c). This modification is present mainly asymmetrically with the coding strand, with 64% of 5mC occurring in the antisense strand of coding regions (Fig. 4d,e), and most methylated cytosines were found at the dinucleotide GC (Fig. 4a). We found that 50.3% of methylated cytosines are located within genes, while this number rises to 65.4% in the case of adenines. In turn, 39% of the genes present 5mC modification, and 70% of them are shared between epimastigotes and trypomastigotes. Similarly, half of the genes with 6mA are shared between the two stages.

## Local chromatin structure affects global transcription in trypanosomes

**Fig. 5:**
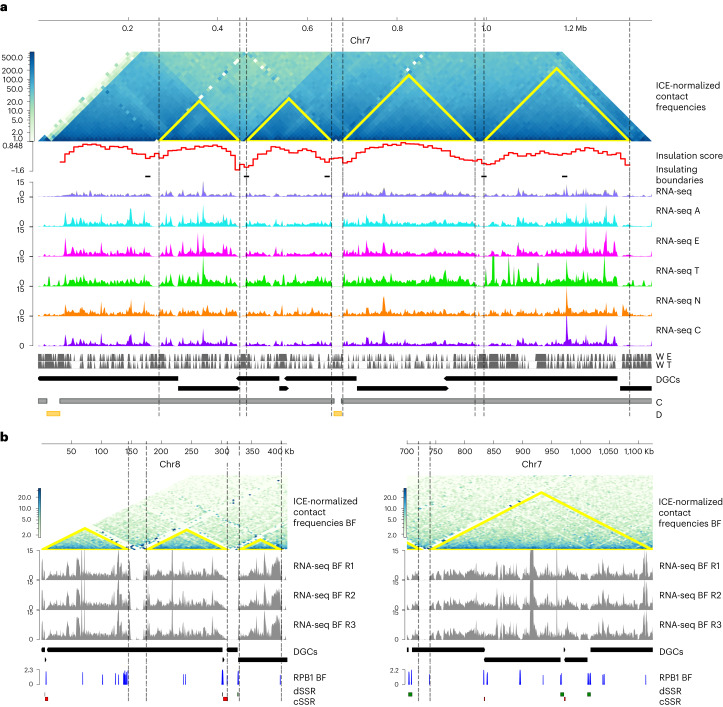
Chromatin organization of trypanosomes affects gene expression. **a**, Normalized Hi-C contact map of chr7 of *T. cruzi* at 10 kb resolution. CFDs identified using the insulation TAD-calling algorithm from TADtool are indicated in yellow triangles. Insulation score values were calculated with FAN-C. RNA expression (CPM) across the chromosome is depicted as coverage plots using a bin size of 10 bp. For each condition, two biological replicates are plotted. Vertical grey bars indicate the position of all well-positioned nucleosomes. The black vertical dashed lines highlight the CFD boundaries that align with the chromosome regions depleted for expression levels. Of the low-coverage regions, 26% correspond to SSR, 38% to CFD boundaries, 19% to SSRs and CFD boundaries, and 27% to unknown reasons. **b**, Normalized Hi-C interaction frequencies of chr7 and chr8 of the *T. brucei* BF shown as a two-dimensional heatmap at 5 kb resolution: chr7 from 700 kb to 1,125 kb and chr8 from 1 kb to 410 kb. RNA expression across the chromosomes in the BF is depicted as normalized (CPM) coverage plots using a bin size of 10 bp. RBP1 (the largest subunit RNA Pol II) enrichment from the BF of *T. brucei*. SSRs where the transcription sense converges (cSSR) or diverges (dSSR) are indicated by red and green, respectively.

**Extended Data Fig. 3:**
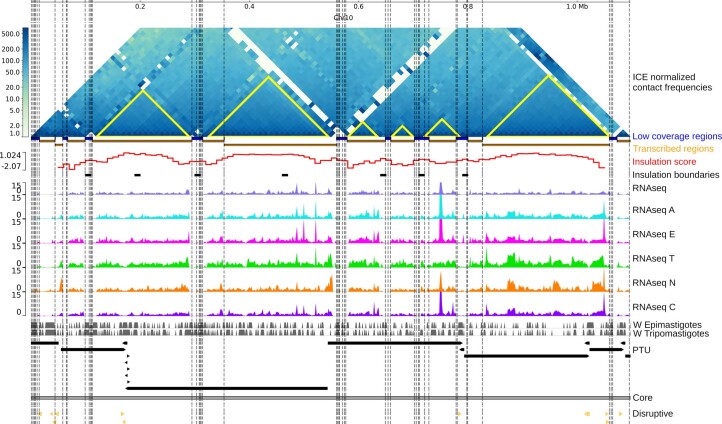
Data integration. Normalized Hi-C interaction frequencies of chromosome 10 of T. cruzi displayed as a two-dimensional heatmap at 10 kb resolution. Chromatin Folding Domains (CFDs) identified using TADtools are indicated as triangles on the Hi-C map. Insulation score values were calculated using FAN-C and the black horizontal dashes represent the local minima of the insulation score, indicatives of the domain boundaries. RNA expression across the chromosome is depicted as coverage plots using a bin size of 10 bp. For each condition, two biological replicates are plotted. A: amastigotes; E: epimastigote; and T: trypomastigote forms. Vertical gray bars indicate the position of all well-positioned nucleosomes. Polycistronic Transcription Units (PTUs). This is a core chromosome as the core compartment comprises the entire chromosome. Some disruptive genes present in this chromosome are indicated as yellow arrows. Zero coverage regions ($\ge$ 500 bp length) are shown as black vertical lines. Low coverage regions were determined considering all transcriptomes and are represented as blue blocks.

Global expression analysis in *T. cruzi* revealed the presence of large actively expressed genome stretches flanked by silent regions—with very low or undetectable RNA levels—of average length 14 kb throughout the life cycle (Extended Data Fig. 3 and Supplementary Table 4). Considering it is proposed that DGCs are transcribed as PTUs, we expected these expressed regions to coincide with the extremes of DGCs, and those depleted of RNAs to coincide with SSRs. Notably, most of these silent regions (65%) correspond to internal areas of the PTUs, indicating that in several cases, there is no coincidence between DGCs and PTUs. Strikingly, the boundaries of transcribed regions are very well correlated with the CFDs we described (Fig. 5a). Therefore, the transcriptionally active regions of the chromosome between two silent regions are flanked by genome sites in close three-dimensional proximity. These results show that transcription is driven by the local structure of the chromatin and that there is not necessarily a coincidence between DGCs and PTUs.

The transcriptionally active regions of the chromosomes in the core compartment can span, on average, 64 kb (up to 384 kb), and the number of genes they contain can vary from 1 up to 173 genes (mean of 29 genes; Supplementary Table 4). CFD boundaries suppress the expression of the genes, and the transcriptionally active region in between includes fractions of different DGCs (up to three different DGCs). The presence of these regions where a large set of linearly associated genes are transcribed is not as evident in the disruptive compartment that contains the antigenic genes, even in trypomastigotes.

To study whether this is a common feature of trypanosomes, we performed the same analyses in *T. brucei* Lister 427 using Hi-C and RNA-seq data from the bloodstream form (BF)^9,23^. The CFDs determined also coincide with several DGC-internal regions where the transcription is interrupted in *T. brucei* (Fig. 5b). To further characterize these results, we analysed chromatin immunoprecipitation sequencing (ChIP–seq) data^32^ of the RNA polymerase II large subunit, RBP1. As expected, an enrichment of RBP1 was found at divergent and convergent SSRs, where transcription starts and ends. However, a striking enrichment of RBP1 was also observed at the internal regions of the DGCs that are flanked by the chromatin domains, indicating that they constitute transcription start and end regions. These results indicate that transcription initiation and termination are determined not only by DGC boundaries but also by chromatin structural domains in *T. brucei*.

## Chromosome-folding domains are conserved across strains and stages

**Fig. 6:**
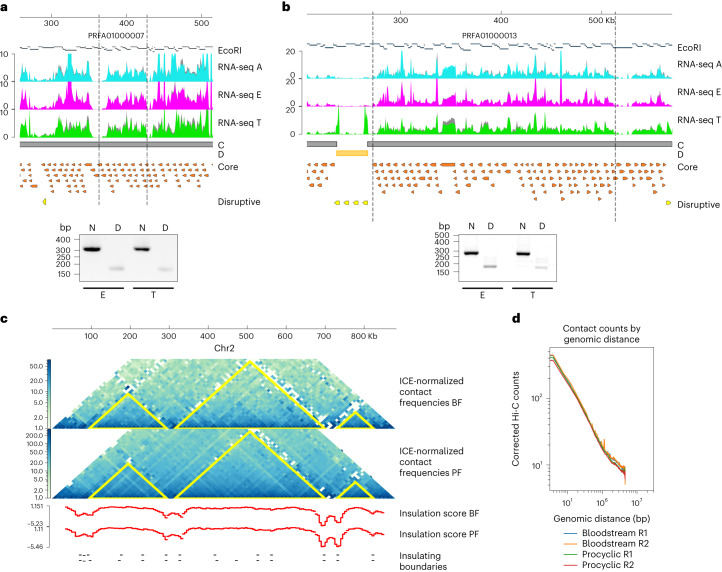
Self-interacting domain boundaries are conserved across cell types and strains. **a**–**d**, Results of the 3C assay. PCR amplification of cross-linked DNA template cut with EcoRI. PCR products were separated on 2% agarose gel stained with ethidium bromide. N, neighbour primers; D, distance primers. Two independent experiments were carried out. **a**, Region between position 363,000 and 427,000 of scaffold PRFA01000007. PCR products 300 nt and 166 nt in length were amplified with N and D primers, respectively. **b**, Region between position 272,663 and 513,267 of scaffold PRFA01000013. PCR products 275 nt and 171 nt in length were amplified with N and D primers, respectively. **c**, Normalized Hi-C maps of PFs and BFs at 10 kb resolution. CFD identified using TADtools are indicated with yellow triangles on Hi-C maps. The insulation score and local minima of the insulation score were calculated for both forms using FAN-C. Self-interacting chromosomal domains are conserved across African trypanosomes. **d**, We observed no differences (*P* > 0.05) in long-range (20 kb) versus short-range (<20 kb) contacts between PFs and BFs.

To determine the spatial genome organization at different stages of the *T. cruzi* life cycle and confirm that gene expression is affected by the local structure of the chromatin, we performed chromatin conformation capture (3C) experiments. To select which topological domain boundaries to analyse, we used the Hi-C maps from Brazil A4 to design 3C primers (Supplementary Table 5) in syntenic loci with the Dm28c strain (Supplementary Fig. 3). The chromatin interactions we observed in Brazil A4 epimastigotes by Hi-C analysis (chromosome (chr) 10: 660,000–700,000; chr29: 140,000–380,000) were also determined in 3C assays of Dm28c epimastigotes and trypomastigotes (PRFA01000007: 363,000–427,000; PRFA01000013: 272,663–513,267) (Fig. 6a,b). The resulting CFDs on Dm28c are 64 and 241 kb in length and are composed of 32 and 124 genes, respectively. Therefore, not only are collinearity and synteny blocks of genes at the sequence level present but also the three-dimensional organization of the genome is maintained between the strains and different stages of the parasite life cycle.

Finally, we investigated whether chromatin domain conservation also occurs during different stages of the *T. brucei* life cycle. We compared the locations of local minima of the insulation score (indicative of regions between two self-interacting domains) identified using Hi-C maps of procyclic forms (PFs) and BFs. Most of the boundary regions are shared between both stages (Fig. 6c). Of the 318 domain boundaries determined in the BF and 295 boundaries in the PF, 120 are common between both stages, corresponding to 37.7% and 40.5%, respectively. Moreover, we observed no variation in the contact counts by genomic distance, neither in short-range nor in long-range contacts (Fig. 6d). These results indicate that the overall three-dimensional organization of the core compartment of the genome is largely invariant in trypanosomes.

## Discussion

The genome organization of trypanosomes has a common property in that genomes are divided into a core, syntenic among trypanosomatids, and a disruptive, or non-syntenic, compartment. The disruptive compartment is located in the subtelomeres in the African trypanosomes^23^ while it is widely distributed along the genome in *T. cruzi*^22^. The disruptive compartment in *T. cruzi* comprises, in several cases, almost the entire chromosome, representing a relevant difference from *T. brucei*. This particular genome arrangement suggests an impact on three-dimensional chromosome organization. In the absence of enhancer–promoter regulation, spatial chromatin conformation could constitute a relevant mechanism for regulating gene expression in these parasites. Analysing the frequency of intra-chromosomal DNA–DNA interactions, we observed that the junctions between the core and disruptive compartments are prominent insulating boundaries of chromatin interactions. Therefore, the linear genome architecture (core and disruptive) has a three-dimensional—C and D—counterpart both in *T. cruzi* and *T. brucei*^23^.

The genome of *T. cruzi* is organized into CFDs. These CFDs are smaller in the D chromatin and present a higher frequency of contacts, indicating that chromatin is more compact in this compartment, which is in agreement with recent FAIRE (Formaldehyde-Assisted Isolation of Regulatory Elements)–seq studies^8^. In this sense, the D compartment is densely packed with well-positioned nucleosomes, suggesting a less permissive chromatin structure. In turn, the D compartment exhibits a high frequency of inter-chromosomal contacts. This higher compaction of D chromatin may have the role of both avoiding spurious transcription and facilitating DNA recombination events necessary for generating the antigenic variability essential for the immune system evasion strategy of *T. cruzi*. Comparably, the global mechanism in *T. brucei* implies the recombination of the VSG genes into subtelomeric regions to switch the expression of the protein that covers the parasite’s surface as the infection progresses and the immune system matures its response^33^. Therefore, both parasite genomes exhibit a D compartment composed of species- and stage-specific surface proteins, which would generate favourable conditions for generating antigenic diversity, by either recombination, allelic exclusion or differential transcription. On the contrary, it is expected that the C compartment, which does not undergo antigenic variation, presents less variability concerning the structure of the chromatin. In agreement with this, we observed well-conserved CFDs in this compartment in different stages and strains in both parasites. Thus, the main differences in genome architecture are those that allow *T. brucei* to survive extracellularly and *T. cruzi* to adapt to both intracellular and extracellular forms.

One mechanism to ensure coordinated regulation of gene expression of large stretches is the formation of higher-order three-dimensional chromatin structures^34^. We investigated whether this three-dimensional genome organization implies selective regulation of gene expression, and we found that the C chromatin is mainly composed of genes with high and medium expression, while most of the genes from the D chromatin exhibit low or undetectable expression and a discrete number of genes highly increase their expression in trypomastigotes. The C compartment functions as a constitutively expressed region as its genes are expressed similarly throughout the life cycle, whereas the D compartment constitutes a specialized region for the infective stage. These findings modify the accepted paradigm that these parasites transcribe indiscriminately and then modulate post-transcriptionally their expression. This is observed in the C compartment, but it does not apply to the D compartment as very few of the hundreds of genes in each multigenic family are highly expressed. Both in *T. cruzi* and *T. brucei*, the D compartment constitutes regions of high chromatin compaction, which could favour gene-to-gene regulation, preventing spurious transcription and favouring intra- and inter-chromosomal recombination. In *T. brucei*, multiple VSG genes are transcribed before a single gene is chosen^9^, and this choice relies on the formation of an extra-nucleolar structure that contains a local reservoir of RNA polymerase I and RNA processing factors^9,35^. In *T. cruzi*, the expression of the highly expressed surface genes is probably determined by the nucleoplasmic location of the loci and the proximity to the transcription and processing machinery, but Hi-C data of the infectious stage are necessary to study this and other aspects of the genome biology at this stage. Finally, the maintenance of three-dimensional organization along the infective and non-infective stages can constitute an advantage in driving life-cycle-specific transcriptional regulation.

The genome regions between two CFDs are characteristically RNA silent, while the regions contained in CFDs are actively transcribed, indicating that the expression is strongly determined by the three-dimensional organization of the chromatin and does not necessarily correlate with the DGCs. Therefore, the paradigm that there is a correspondence between DGCs and PTUs is not always fulfilled. Importantly, this is more than just a feature of *T. cruzi*. In *T. brucei*, when we eliminated the bias of DGCs as a reference for transcription start and end, we also found a similar correlation between the CFD and transcription in the C compartment, denoting a common feature of trypanosomes. RBP1 is enriched in these DGC-internal regions that are flanked by CFDs, endorsing that these are regions where transcription starts and ends. Transcription initiation and termination internal to DGC units were previously described in trypanosomes^36,37^, and future studies are necessary to determine whether these DGC-internal transcription start and stop sites present similar characteristics as those in the SSRs (for example, histone modifications, histone variants, base J, methylation). Taken together, these results strongly support that the flow of genetic information from DNA to RNA in trypanosomes is regulated at transcription initiation through epigenetic mechanisms.

The most accepted idea regarding the differential regulation of gene expression in trypanosomes is that transcription is constitutive and that transcript levels are individually modulated by post-transcriptional mechanisms. However, here we provide evidence that the expression of the D compartment in *T. cruzi* does not respond to this widely accepted model: most of their genes exhibit low and undetectable levels, and a discrete number greatly increase their expression in the trypomastigote stage. These results do not go against the statement ‘trypanosomes mainly regulate at the post-transcription level’, but rather, this would be the expression modulation model in the C compartment. We propose that there are two major regulation models and that they are spatially isolated in the nucleoplasm by the C and D compartments in which the chromatin is organized. The compartmentalization of the genome makes it possible to isolate the molecular mechanisms necessary for the regulation of each type of gene. Moving forwards, the incorporation of time-course data and single-cell studies will reveal further insights into the mechanisms of three-dimensional chromatin structure and gene regulation in trypanosomes.

## Methods

### Cell culture

*T. cruzi* Dm28c^38^ parasites were cultured axenically in liver infusion tryptose medium supplemented with 10% (v/v) inactivated fetal bovine serum (Gibco) at 28 °C. Trypomastigotes and intracellular amastigotes were collected from infected Vero cells as described^39^. Vero cells were cultivated in Dulbecco’s modified Eagle’s medium (DMEM (1×) + GlutaMAX-l, Gibco by Life Technologies) supplemented with 10% (v/v) fetal bovine serum, (Gibco), penicillin 100 U ml^−1^ and 100 µg ml^−1^ streptomycin (Thermo Fisher Scientific) at 37 °C in a humidified 5% CO_2_ atmosphere.

### Methylation analysis

High-molecular-weight genomic DNA was purified using phenol–chloroform–isoamyl alcohol extraction followed by EtOH precipitation^40^. Purified genomic DNA was fragmented to 10 kb using g-TUBEs (Covaris) to maximize the yield of nanopore sequencing. Genomic libraries were prepared using the Ligation Sequencing Kit (SQK-LSK109) and Native Barcoding Expansion Kit (EXP-NBD104, Oxford Nanopore Technologies) following the protocol for native barcoding of genomic DNA. Pooled samples were quantified using the Qubit dsDNA HS Assay Kit, loaded onto a R9.4.1 Flow Cell (FLO-MIN106D) and sequenced on an Oxford Nanopore Technologies (ONT) MinION platform for 72 h. The coverage per sample ranged from 29× to 34×. Raw data (multi_read_fast5 files) were converted to single_read_fast5 files using ont_fast5_api v3.1.6 (ONT) and base called using Guppy v3.6.0 (ONT) with dna_r9.4.1_450bps_modbases_dam-dcm-cpg_hac.cfg configuration. The strand-sensitive and single-nucleotide-based detection of DNA base modifications was performed with DeepMod^41^ v0.1.3, which detects m5C and m6A in DNA using the recurrent neural networks model. Only genomic positions with >5 read coverage and >90% of methylation percentage were considered for the analysis. Dm28c reference genome (TcruziDm28c2018 from TriTrypDB version 51) was used as input, and methylation positions were assigned to different genomic regions using BEDTools^42^ v2.27.1. Core and disruptive methylation quantifications were made in the largest 30 scaffolds of the Dm28c 2018 genome assembly, representing 38% of the genome size.

### Nucleosome position analysis

MNase–seq data^7^ was obtained from National Center for Biotechnology Information (NCBI; BioProject PRJNA665060) using fasterq-dump v3.0.0. Quality control was performed using FastQC v0.11.9, and reads with less than 70 bp after trimming low-quality nucleotides (–nextseq-trim = 20) and clipping the adapter sequence were removed using Cutadapt^43^ v2.7. For nucleosome calling, paired-end reads were aligned to both reference genomes Dm28c (TcruziDm28c2018 from TriTrypDB version 51) and Brazil A4 (TcruziBrazilA4 from TriTrypDB version 53) using Bowtie^44^ v1.3.1, allowing up to two mismatches and a maximum insert size of 500 bp. The resulting BAM files were imported and processed in R v4.1.3 to merge biological replicates by chromosome. After duplicate removal with Picards MarkDuplicates (Picard Toolkit 2019), Fourier transform filtering to remove noise and peak calling to accurately define and classify the location of nucleosomes across the genome were performed with nucleR^45^. Nucleosomes with sharp signals of the shape of the associated peaks were labelled as well-positioned (W) nucleosomes (high localization score), while flat peaks were labelled as fuzzy nucleosomes (low localization score)^45^. In this analysis, well-positioned nucleosomes were considered when the nucleR peak width score and height score were higher than 0.6 and 0.4, respectively. Finally, Nucleosome Dynamics^46^ v1.0 was used to study differences between stages: occupancy differences (insertions and evictions) and displacement of nucleosomes (shifts).

### 3C

Parasites (1 × 10^8^ trypomastigotes and 5 × 10^9^ epimastigotes) were collected through centrifugation at 2,200 × *g* for 10 min at room temperature and washed twice with PBS 1×. Cells were fixed with 1% formaldehyde for 15 min at room temperature on a shaker. The cross-linking was quenched by adding 2.5 ml of 1 M glycine (125 mM) and incubating for 5 min at room temperature, then on ice for 15 min. Fixed cells were then centrifuged, washed twice with ice-cold PBS 1× and resuspended in 1.4 ml of cold lysis buffer (10 mM Tris–HCl pH 8, 10 mM NaCl, 0.2% Nonidet P-40, 1× protease inhibitors). After homogenization with a Dounce homogenizer pestle A, the nuclei were washed twice with 1.25× rCutSmart buffer (New England BioLabs) and resuspended in 0.5 ml of rCutSmart buffer (New England BioLabs) containing 0.3% SDS. Samples were incubated for 40 min at 65 °C and then for 20 min at 37 °C. Then, 2% Triton X-100 was added, and the nuclei were further incubated for 1 h at 37 °C to sequester the SDS. Cross-linked DNA was digested using 400 U of EcoRI-HF (R3101 New England BioLabs) overnight at 37 °C, and the enzyme was inactivated with 1% SDS at 65 °C for 20 min. Digestion products were diluted by the addition of 4 ml 1.1× T4 DNA Ligase Reaction Buffer (New England BioLabs) with 1% Triton X-100 and incubated for 1 h at 37 °C. DNA was ligated for 4 h in a water bath at 16 °C (30 Weiss units of T4 DNA Ligase, M0202; New England BioLabs). Then, 300 μg of proteinase K was used to reverse the cross-linking overnight at 65 °C. Finally, samples were incubated with 300 μg RNase A, and the DNA was purified by phenol extraction followed by ethanol precipitation. The control library with all possible ligation products was prepared in parallel using non-cross-linked genomic DNA. Ligation products were detected and quantified in control and cross-linked libraries with PCR using locus-specific primers. We designed a set of six oligos excluding sequences from repetitive regions (Supplementary Table 5). The linear range of amplification was determined for all the libraries by serial dilution of DNA amounts.

### Transcriptomic analysis

Total RNA from cell-derived trypomastigotes, epimastigotes and intracellular amastigotes was treated with RiboZero Gold magnetic beads from a eukaryote kit (250 ng as input) as described previously^47^. Library preparation of purified RNA was then performed using the TruSeq Stranded Total RNA kit (Illumina) with random primers. A total of 80 bp paired-end reads were generated using an Illumina NextSeq 500 MID platform. Raw reads were processed with Cutadapt^43^ v2.7 to trim low-quality nucleotides (–nextseq-trim = 20) and clip the adapter sequence. The resulting reads with less than 70 bp were discarded (–minimum-length = 70). Trimmed reads were checked for per-base quality using FastQC v0.11.9. Cleaned reads were then aligned to the Dm28c reference genome (TcruziDm28c2018 from TriTrypDB version 51) using Hisat2 (ref. ^48^) v2.1.0 (–no-spliced-alignment–rna-strandness RF), and mapped reads with a mapping quality score <10 were discarded with SAMtools^49^ v1.16.1 as it was previously described^50^. The coverage files were smoothed, and counts per million (CPM) normalized in a bin size of 10 bp using the bamCoverage function from deepTools^51^. Salmon^52^ v1.5.1 was used to estimate transcript levels, and statistical analysis of differential expression of mRNAs was tested with DESeq2 (ref. ^53^) v1.38.3. The expression of each transcript was quantified in transcript per million (TPM) units. Consistency between replicates was assessed with Pearson correlation and principal component analysis. Genes were considered as differentially expressed when they were statistically significant (*P*_adj_ < 0.001) and had a fold change in transcript abundance of at least two (|log2FC| > 1). To study the dynamic of RNA levels in genome compartments along the stages, we classified the transcripts into three groups based on their relative abundance. High-, medium- and low-expression genes were determined using quantiles on normalized counts. Also, to investigate expression patterns, we classified the scaffolds into ‘core’, ‘disruptive’ and ‘mixed’ according to the percentage of compartments that make them up. The bimodality coefficient was calculated using the mousetrap^54^ v3.2.1 R package. The transcriptomic data corresponding to the different subcellular compartments of *T. cruzi*, Brazil A4 and *T. brucei* were analysed identically.

To study immature and nascent transcripts, 5′ untranslated regions (UTR) were first annotated and assigned to protein-coding genes using UTRme^55^ v1.1.0. SLA sites identified at the same positions in all transcriptomes were used for the analysis to avoid bias of differential processing between stages. Alignments of reads spanning SLA sites were quantified, normalized and classified as processed and unprocessed according to the presence or absence of spliced leader sequence in the read, respectively.

To identify RNA zero coverage regions, genome coverage for all positions in BEDGRAPH format was calculated from mapping-quality-filtered BAM files using the genomecov function from BEDTools^42^ v2.27.1 with the -bga option. Then, all regions of the genome with zero coverage were extracted, and intervals <500 bp in length were filtered out.

### Chromatin immunoprecipitation analysis

The 37 × 2 bp paired-end raw reads were processed with Cutadapt^43^ v2.7 to remove low-quality bases from both ends (-q 30), remove flanking N bases from each read (–trim-n) and discard reads containing more than 5 N bases (–max-n 5). The resulting reads with less than 35 bp were discarded (–minimum-length = 35). Raw and trimmed reads were checked for per-base quality using FastQC v0.11.9. Cleaned reads were then aligned to *T. brucei* reference genome (TbruceiLister427_2018 from TriTrypDB version 53) using Bowtie^44^ v1.3.1, allowing two mismatches (-v 2). Unique alignments reported (-m 1) were compressed and sorted using SAMtools^49^ v1.16.1, and duplicate reads were removed using Picard tools v2.25.5 (MarkDuplicates). Normalization and peaking calling were conducted using MACS2 (ref. ^56^) v2.2.7.1 with a false discovery rate of 5%, and peaks with low fold enrichment (-q 0.05 and –fe-cutoff 1.5) were filtered out. The –g parameter was set at 50081021.

### Chromatin conformation computational analysis

Hi-C datasets were processed from raw reads to normalized contact maps using HiC-Pro^57^ v3.1.0. Quality was assessed using MultiQC. TAD calling was performed with TADtools^58^ and the hicFindTADs function of HiCExplorer^59^ v3.7.2. The insulation score was calculated at 30, 50, 70, 100 and 150 kb sliding windows with FAN-C tool^60^ v0.9.26b2. The data integration, comparison and visualization of the different datasets were performed using pyGenomeTracks^61^ v3.7.

All figures were designed using GIMP v2.10.32.

### Reporting summary

Further information on research design is available in the Nature Portfolio Reporting Summary linked to this article.

### Supplementary information

Supplementary Information Supplementary Figs. 1–3 and Tables 1–5.

Reporting Summary

### Source data

Source Data Statistical source data and agarose gel.

## Data Availability

Raw sequencing data for Illumina and Oxford Nanopore Technologies platforms were deposited to NCBI: RNA-seq to BioProject ID PRJNA850400 and Methylation to BioProject ID PRJNA935260. Hi-C and RNA-seq data for *T. cruzi* Brazil A4 epimastigotes from a previous study^24^ are available from NCBI under accession numbers SRR11803985 and SRR12792489. Hi-C data for the *T. brucei* PF and BF, as well as RNA-seq data from the BF from previous studies^9,23^, are available from the NCBI SRA (Sequencing Read Archive) under accession numbers ERR3712002, ERR3712009, SRR7721317, SRR7721318 and SRR5809498–SRR5809500. RBP1 ChIP–Seq^32^ and MNase–seq^7^ data are available under SRR9022833, SRR9022834, SRR13260277, SRR13260279 and SRR12710803–SRR12710808 accession numbers. RNA-seq data from different subcellular compartments of *T. cruzi*^29^ are available from the NCBI under accession numbers SRR4232036–SRR4232038. Genome FASTA and GFF files were retrieved from TritrypDB (https://tritrypdb.org/tritrypdb/app): TcruziDm28c2018 version 51, TcruziBrazilA4 version 53 and TbruceiLister427_2018 version 53. Source data are provided with this paper.
