# Supplementary material for: Genome-wide chromatin interaction map for Trypanosoma cruzi
Source: Nat Microbiol. 2023 Oct 12;8(11):2103–14. doi: 10.1038/s41564-023-01483-y (PMC10627812; doi:10.1038/s41564-023-01483-y)
Supplement: Supplementary file 1 — Supplementary Figs. 1–3 and Tables 1–5. [file 41564_2023_1483_MOESM1_ESM.pdf]

nature microbiology

Article

https://doi.org/10.1038/s41564-023-01483-y

Genome-wide chromatin interaction map for *Trypanosoma cruzi*

In the format provided by the
authors and unedited

Supplementary Figures

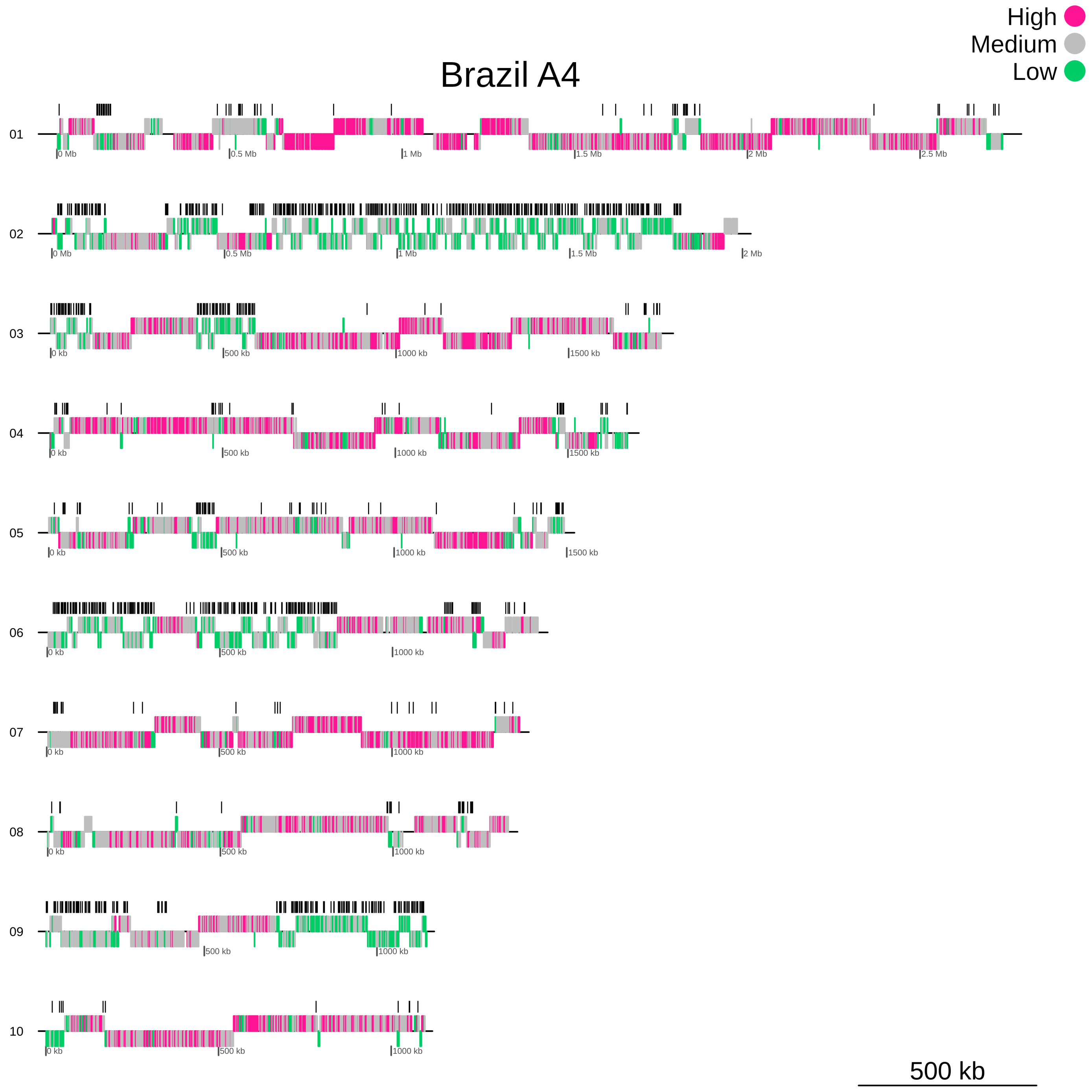

Supplementary Fig. 1: Genomic distribution of genes classified according to RNA expression (RNA-seq) in *T. cruzi* Brazil A4 strain. Chromosomes of Brazil A4 genome assembly. Disruptive genes (TS, TcMUC and MASP) are indicated in black. Low, medium, and high expressed genes are shown in green, gray, and magenta, respectively.

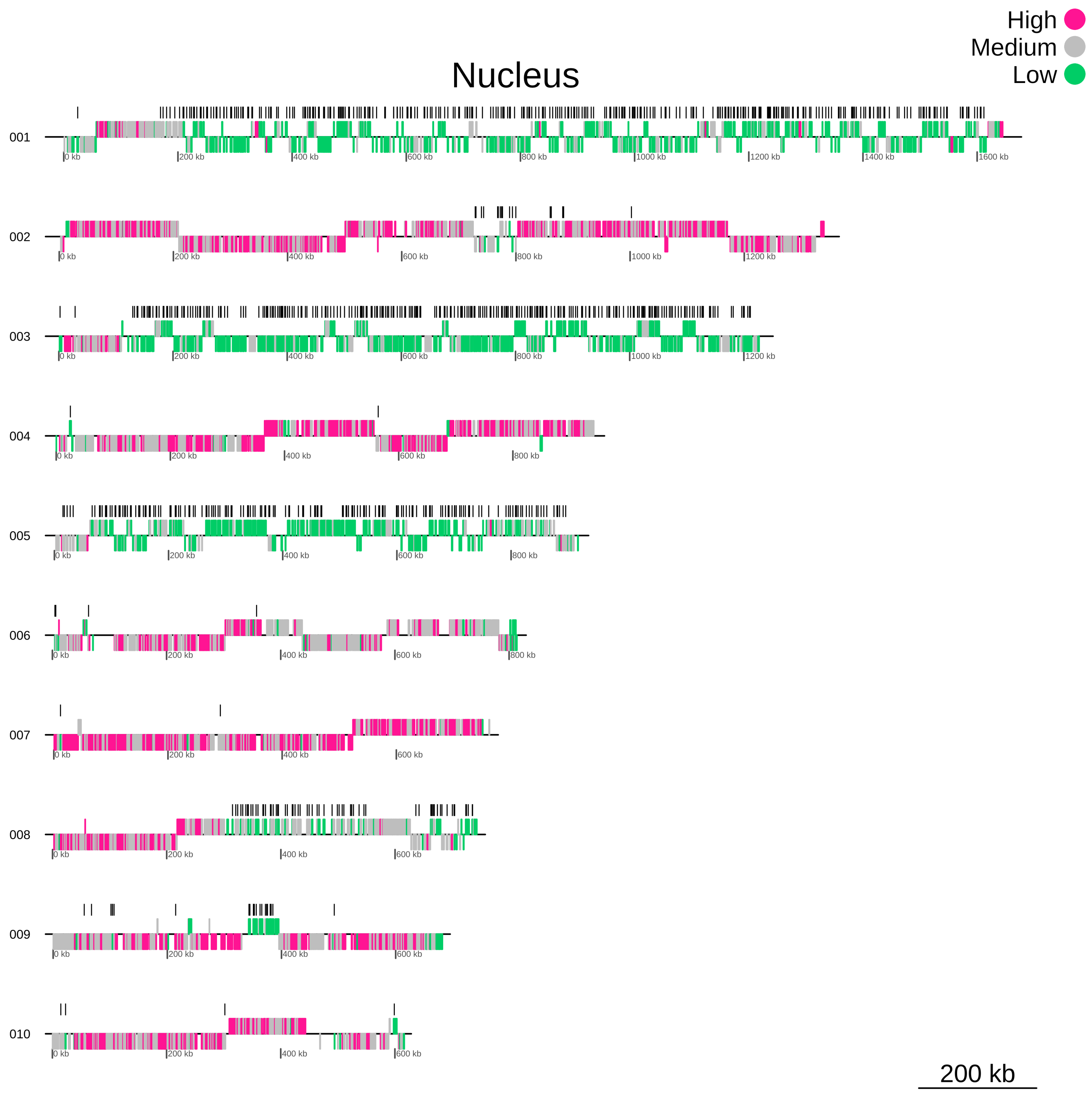

Nucleus
High
Medium
Low
001
002
003
004
005
006
007
008
009
010
0 kb
200 kb
400 kb
600 kb
800 kb
1000 kb
1200 kb
1400 kb
1600 kb
200 kb

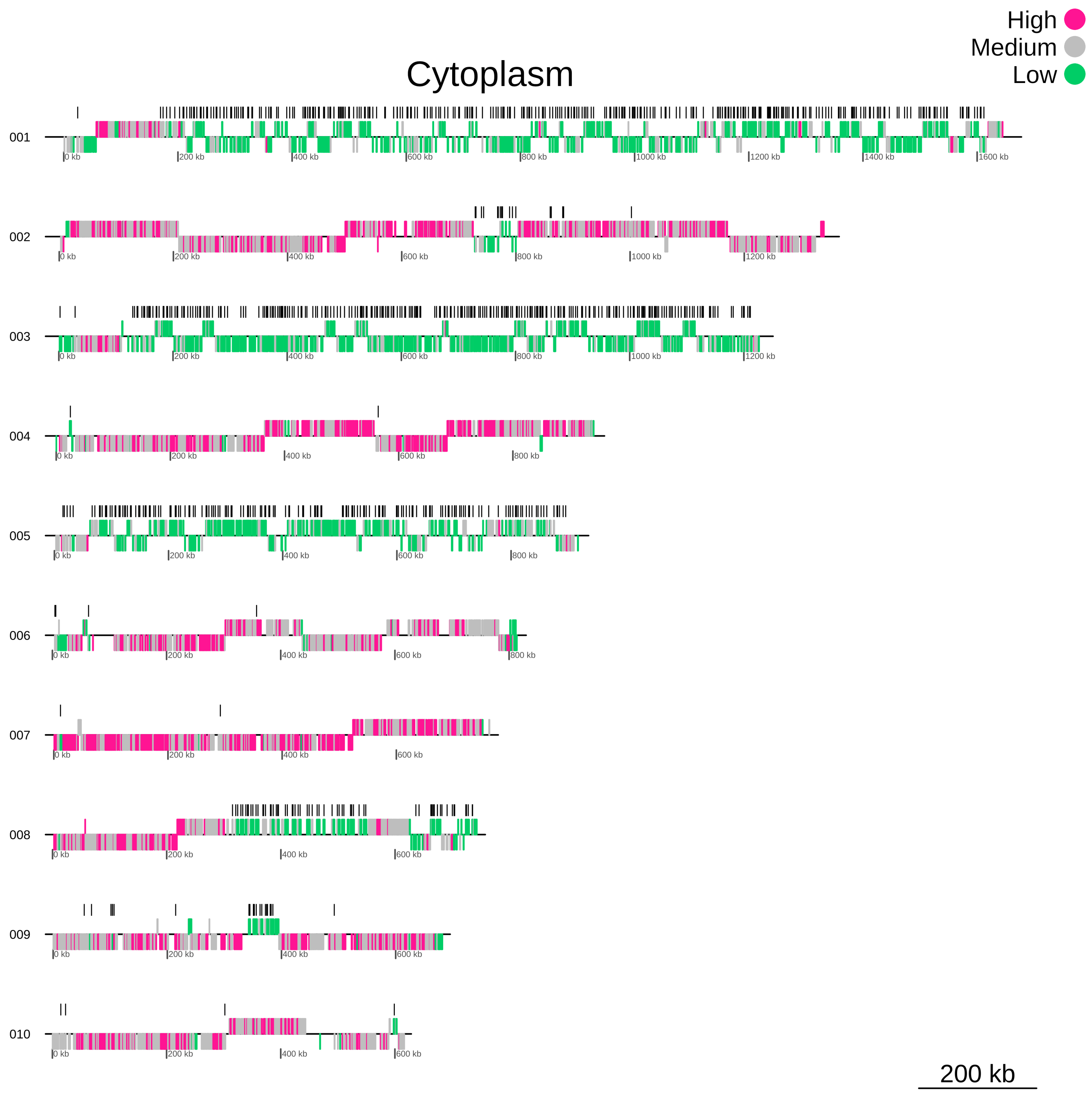

Supplementary Fig. 2: Genomic distribution of genes classified according to the RNA expression level (RNA-seq) in the nucleus and cytoplasm. Disruptive genes (TS, TcMUC and MASP) are indicated in black. Low, medium, and high expressed genes are shown in green, gray, and magenta, respectively.

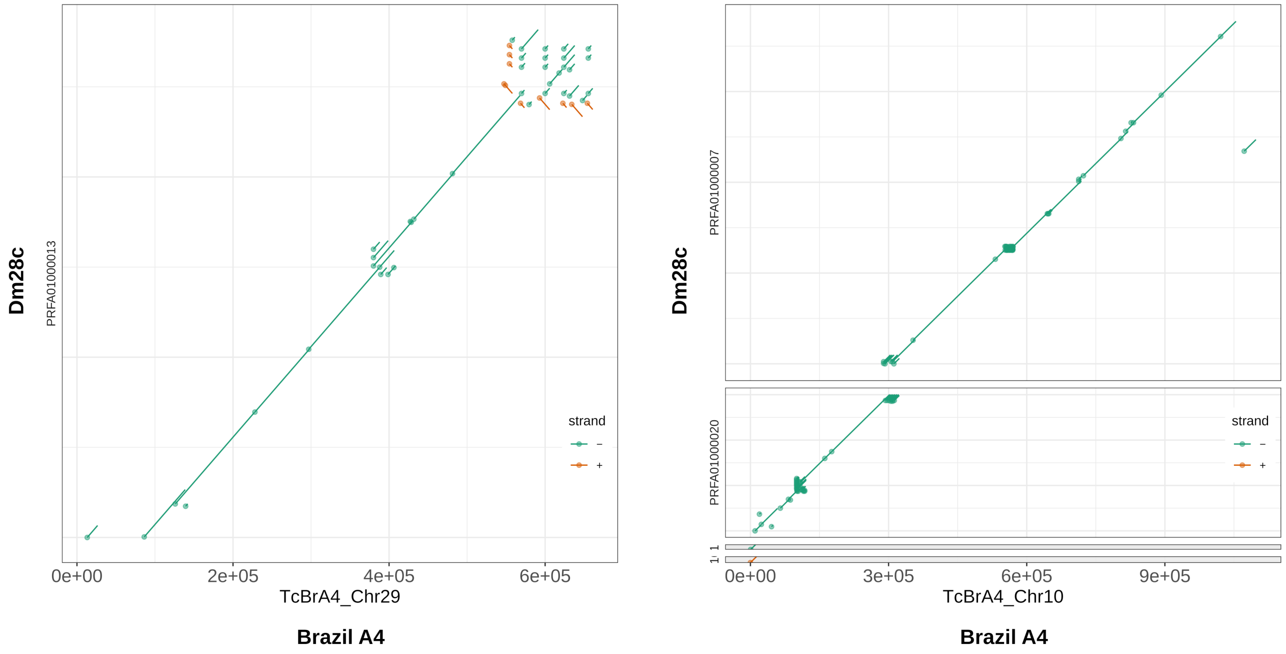

Supplementary Fig. 3: Whole chromosomal alignments. Chromosome 10 from *T. cruzi* Brazil A4 presents synteny with two scaffolds of *T. cruzi* Dm28c genome assembly. The region we studied is present in scaffold PRFA01000007. Chromosome 29 from *T. cruzi* Brazil A4 presents synteny with scaffold PRFA01000013 of *T. cruzi* Dm28c genome assembly.

Supplementary Tables

Supplementary Table 1: Chromatin folding domains. CFD predictions of *T. cruzi* using different algorithms.

Supplementary Table 2: Classification of the *T. cruzi* chromosomes. *T. cruzi* Dm28c and Brazil A4 chromosomes were classified as Core, Disruptive or Mixed according to the genome compartment composition. Chromosomes were classified as core or disruptive if one of these genomic compartments covers more than 80% of the length of the chromosome, while the rest are considered Mixed.

Supplementary Table 3: Differentially expressed genes along the life cycle stages of *T. cruzi*. Result of differential gene expression analysis based on the negative binomial distribution. Differentially expressed genes were identified using DESeq2 R package51 using Wald test prior shrinkage estimation (ashr). p value were corrected for multiple testing using the Benjamini and Hochberg (BH) method. An absolute value of shrunken log2 fold change >1 and an adjusted p value <0.001 (two-sided) was used as the threshold for selecting differentially expressed genes.

Supplementary Table 4: Length of low or zero coverage and transcribed regions on core chromosomes. As the zero cov regions in all transcriptomes are difficult to define in the disruptive compartment because it is a stage-specific expression compartment, The analysis of these regions was carried out on chromosomes defined as core chromosomes (80-100% of chromosome length correspond to core compartment): Chr1, Chr4, Chr7, Chr8, Chr10, Chr14, Chr15, Chr16, Chr17, Chr18, Chr22, Chr23, Chr28.

Supplementary Table 5: Chromatin Conformation Capture 3C primers.

Chromosome	Start	End	Length	Compartment
TcBrA4_Chr1	12000	120000	108000	C
TcBrA4_Chr1	160000	280000	120000	C
TcBrA4_Chr1	335000	585000	250000	M
TcBrA4_Chr1	640000	905000	265000	C
TcBrA4_Chr1	900000	925000	25000	C
TcBrA4_Chr1	920000	1185000	265000	C
TcBrA4_Chr1	1213000	1314000	101000	C
TcBrA4_Chr1	1318000	1419000	101000	C
TcBrA4_Chr1	1460000	1805000	345000	C
TcBrA4_Chr1	1800000	1975000	175000	M
TcBrA4_Chr1	1990000	2100000	110000	C
TcBrA4_Chr1	2100000	2695000	595000	C
TcBrA4_Chr2	150000	345000	195000	C
TcBrA4_Chr2	365000	425000	60000	D
TcBrA4_Chr2	475000	575000	100000	C
TcBrA4_Chr2	625000	710000	85000	M
TcBrA4_Chr2	720000	820000	100000	D
TcBrA4_Chr2	815000	850000	35000	D
TcBrA4_Chr2	845000	885000	40000	D
TcBrA4_Chr2	880000	1015000	135000	D
TcBrA4_Chr2	1010000	1090000	80000	D
TcBrA4_Chr2	1090000	1115000	25000	D
TcBrA4_Chr2	1110000	1215000	105000	D
TcBrA4_Chr2	1215000	1235000	20000	D
TcBrA4_Chr2	1235000	1465000	230000	D
TcBrA4_Chr2	1465000	1520000	55000	D
TcBrA4_Chr2	1540000	1820000	280000	D
TcBrA4_Chr2	1820000	1865000	45000	C
TcBrA4_Chr3	1	140000	139999	D
TcBrA4_Chr3	140000	180000	40000	C
TcBrA4_Chr3	180000	370000	190000	C
TcBrA4_Chr3	385000	410000	25000	C
TcBrA4_Chr3	610000	640000	30000	C
TcBrA4_Chr3	680000	1620000	940000	C
TcBrA4_Chr3	1630000	1685000	55000	C
TcBrA4_Chr3	1713500	1768708	55208	D
TcBrA4_Chr4	55000	165000	110000	C
TcBrA4_Chr4	180000	240000	60000	C
TcBrA4_Chr4	280000	480000	200000	C
TcBrA4_Chr4	505000	725000	220000	C
TcBrA4_Chr4	755000	845000	90000	C
TcBrA4_Chr4	860000	975000	115000	C
TcBrA4_Chr4	1050000	1200000	150000	C
TcBrA4_Chr4	1220000	1330000	110000	C
TcBrA4_Chr4	1345000	1460000	115000	C
TcBrA4_Chr4	1475000	1535000	60000	C
TcBrA4_Chr5	20000	90000	70000	M
TcBrA4_Chr5	102000	228000	126000	C
TcBrA4_Chr5	295000	420000	125000	C
TcBrA4_Chr5	415000	470000	55000	D
TcBrA4_Chr5	465000	595000	130000	M
TcBrA4_Chr5	595000	620000	25000	C
TcBrA4_Chr5	620000	705000	85000	C
TcBrA4_Chr5	710000	765000	55000	C
TcBrA4_Chr5	785000	855000	70000	C
TcBrA4_Chr5	870000	1100000	230000	C
TcBrA4_Chr5	1112500	1328000	215500	C
TcBrA4_Chr5	1338000	1450000	112000	M
TcBrA4_Chr6	310000	400000	90000	C
TcBrA4_Chr6	525000	645000	120000	D
TcBrA4_Chr6	785000	870000	85000	M
TcBrA4_Chr6	885000	970000	85000	C
TcBrA4_Chr6	1170000	1230000	60000	M

TcBrA4_Chr6	1245000	1370000	125000	M
TcBrA4_Chr7	270000	450000	180000	C
TcBrA4_Chr7	465000	655000	190000	C
TcBrA4_Chr7	680000	975000	295000	C
TcBrA4_Chr7	995000	1320000	325000	C
TcBrA4_Chr8	80000	80001	1	C
TcBrA4_Chr8	135000	355000	220000	C
TcBrA4_Chr8	370000	430000	60000	C
TcBrA4_Chr8	500000	570000	70000	C
TcBrA4_Chr8	585000	745000	160000	C
TcBrA4_Chr8	785000	970000	185000	C
TcBrA4_Chr8	1045000	1195000	150000	C
TcBrA4_Chr8	1215000	1336822	121822	C
TcBrA4_Chr9	75000	175000	100000	D
TcBrA4_Chr9	175000	235000	60000	D
TcBrA4_Chr9	235000	355000	120000	C
TcBrA4_Chr9	400000	710000	310000	C
TcBrA4_Chr9	710000	780000	70000	D
TcBrA4_Chr9	795000	900000	105000	D
TcBrA4_Chr9	900000	1020000	120000	D
TcBrA4_Chr9	1020000	1115000	95000	D
TcBrA4_Chr10	120000	290000	170000	C
TcBrA4_Chr10	325000	545000	220000	C
TcBrA4_Chr10	580000	635000	55000	C
TcBrA4_Chr10	660000	700000	40000	C
TcBrA4_Chr10	720000	785000	65000	C
TcBrA4_Chr10	835000	1060000	225000	C
TcBrA4_Chr11	55000	290000	235000	M
TcBrA4_Chr11	310000	440000	130000	D
TcBrA4_Chr11	475000	695000	220000	M
TcBrA4_Chr11	785000	880000	95000	C
TcBrA4_Chr11	900000	950000	50000	D
TcBrA4_Chr11	950000	1005000	55000	D
TcBrA4_Chr12	185000	450000	265000	
TcBrA4_Chr12	475000	610000	135000	
TcBrA4_Chr12	630000	820000	190000	
TcBrA4_Chr13	65000	240000	175000	
TcBrA4_Chr13	255000	310000	55000	
TcBrA4_Chr13	400000	570000	170000	
TcBrA4_Chr13	660000	715000	55000	
TcBrA4_Chr13	735000	855000	120000	
TcBrA4_Chr13	850000	930000	80000	
TcBrA4_Chr14	130000	270000	140000	
TcBrA4_Chr14	290000	435000	145000	
TcBrA4_Chr14	435000	650000	215000	
TcBrA4_Chr14	730000	925000	195000	
TcBrA4_Chr15	1	260000	259999	
TcBrA4_Chr15	280000	350000	70000	
TcBrA4_Chr15	365000	530000	165000	
TcBrA4_Chr15	550000	620000	70000	
TcBrA4_Chr15	645000	760000	115000	
TcBrA4_Chr15	785000	880000	95000	
TcBrA4_Chr16	430000	500000	70000	
TcBrA4_Chr16	520000	780000	260000	
TcBrA4_Chr16	800000	860000	60000	
TcBrA4_Chr17	70000	420000	350000	
TcBrA4_Chr17	415000	485000	70000	
TcBrA4_Chr17	485000	715000	230000	
TcBrA4_Chr17	715000	810000	95000	
TcBrA4_Chr18	120000	190000	70000	
TcBrA4_Chr18	210000	300000	90000	
TcBrA4_Chr18	325000	780000	455000	
TcBrA4_Chr19	125000	265000	140000	
TcBrA4_Chr19	275000	625000	350000	

TcBrA4_Chr19	625000	690000	65000
TcBrA4_Chr19	690000	745000	55000
TcBrA4_Chr20	350000	595000	245000
TcBrA4_Chr20	95000	155000	60000
TcBrA4_Chr20	175000	240000	65000
TcBrA4_Chr20	350000	390000	40000
TcBrA4_Chr20	390000	595000	205000
TcBrA4_Chr20	590000	610000	20000
TcBrA4_Chr20	610000	680000	70000
TcBrA4_Chr22	260000	350000	90000
TcBrA4_Chr22	365000	435000	70000
TcBrA4_Chr22	450000	535000	85000
TcBrA4_Chr22	535000	645000	110000
TcBrA4_Chr22	645000	665000	20000
TcBrA4_Chr22	665000	695000	30000
TcBrA4_Chr23	110000	215000	105000
TcBrA4_Chr23	215000	385000	170000
TcBrA4_Chr23	385000	410000	25000
TcBrA4_Chr23	405000	505000	100000
TcBrA4_Chr23	505000	560000	55000
TcBrA4_Chr23	575000	640000	65000
TcBrA4_Chr23	635000	730000	95000
TcBrA4_Chr24	85000	160000	75000
TcBrA4_Chr24	305000	475000	170000
TcBrA4_Chr24	495000	570000	75000
TcBrA4_Chr24	570000	620000	50000
TcBrA4_Chr24	670000	710000	40000
TcBrA4_Chr27	135000	180000	45000
TcBrA4_Chr27	315000	380000	65000
TcBrA4_Chr27	225000	315000	90000
TcBrA4_Chr27	380000	470000	90000
TcBrA4_Chr27	520000	560000	40000
TcBrA4_Chr27	575000	615000	40000
TcBrA4_Chr28	130000	215000	85000
TcBrA4_Chr28	240000	345000	105000
TcBrA4_Chr28	365000	480000	115000
TcBrA4_Chr28	500000	530000	30000
TcBrA4_Chr28	525000	675000	150000
TcBrA4_Chr29	85000	120000	35000
TcBrA4_Chr29	140000	380000	240000
TcBrA4_Chr29	440000	550000	110000
TcBrA4_Chr31	1	80000	79999
TcBrA4_Chr31	100000	160000	60000
TcBrA4_Chr31	175000	480000	305000
TcBrA4_Chr31	500000	601716	101716
	Average size =	128559,56	129 kb
	Genome covered (%) =	22112244,00	60,26
	CFD core length =	151536,30	152 kb
	CFD disruptive length =	92808,28	93 kb
	CFD mixed length =	140636,36	140 kb

Chromosome	Start	End	Length	Compartment
TcBrA4_Chr1	335000	605000	270000	M
TcBrA4_Chr1	605000	640000	35000	D
TcBrA4_Chr1	640000	1060000	420000	C
TcBrA4_Chr1	1060000	1320000	260000	C
TcBrA4_Chr1	1320000	1455000	135000	C
TcBrA4_Chr2	80000	160000	80000	D
TcBrA4_Chr2	160000	365000	205000	C
TcBrA4_Chr2	365000	425000	60000	D
TcBrA4_Chr2	425000	575000	150000	M
TcBrA4_Chr2	575000	720000	145000	M
TcBrA4_Chr2	720000	850000	130000	D
TcBrA4_Chr2	850000	1140000	290000	D
TcBrA4_Chr2	1140000	1460000	320000	D
TcBrA4_Chr2	1460000	1545000	85000	D
TcBrA4_Chr2	1545000	1665000	120000	D
TcBrA4_Chr2	1665000	1820000	155000	D
TcBrA4_Chr3	145000	180000	35000	C
TcBrA4_Chr3	180000	410000	230000	C
TcBrA4_Chr3	410000	525000	115000	D
TcBrA4_Chr3	525000	610000	85000	D
TcBrA4_Chr3	610000	1625000	1015000	C
TcBrA4_Chr3	1625000	1715000	90000	M
TcBrA4_Chr4	55000	485000	430000	C
TcBrA4_Chr4	485000	750000	265000	C
TcBrA4_Chr4	750000	850000	100000	C
TcBrA4_Chr4	850000	1150000	300000	C
TcBrA4_Chr4	1150000	1460000	310000	C
TcBrA4_Chr5	95000	410000	315000	M
TcBrA4_Chr5	410000	1110000	700000	M
TcBrA4_Chr5	1110000	1320000	210000	C
TcBrA4_Chr6	245000	315000	70000	D
TcBrA4_Chr6	315000	430000	115000	C
TcBrA4_Chr6	430000	485000	55000	D
TcBrA4_Chr6	485000	525000	40000	D
TcBrA4_Chr6	525000	645000	120000	D
TcBrA4_Chr6	645000	780000	135000	D
TcBrA4_Chr6	780000	870000	90000	M
TcBrA4_Chr6	870000	980000	110000	C
TcBrA4_Chr6	980000	1080000	100000	C
TcBrA4_Chr6	1080000	1250000	170000	M
TcBrA4_Chr9	55000	175000	120000	D
TcBrA4_Chr9	175000	235000	60000	D
TcBrA4_Chr9	235000	710000	475000	M
TcBrA4_Chr9	710000	795000	85000	D
TcBrA4_Chr9	795000	900000	105000	D
TcBrA4_Chr9	900000	1020000	120000	D
TcBrA4_Chr10	105000	310000	205000	C
TcBrA4_Chr10	310000	575000	265000	C
TcBrA4_Chr11	175000	430000	255000	M
TcBrA4_Chr11	430000	950000	520000	M
TcBrA4_Chr11	950000	1010000	60000	D
TcBrA4_Chr14	115000	725000	610000	M
TcBrA4_Chr14	725000	925000	200000	C
TcBrA4_Chr15	70000	880000	810000	C
TcBrA4_Chr16	780000	810000	30000	D
TcBrA4_Chr16	810000	875000	65000	C
TcBrA4_Chr18	115000	805000	690000	C
TcBrA4_Chr19	130000	285000	155000	M
TcBrA4_Chr19	285000	625000	340000	C
TcBrA4_Chr19	625000	695000	70000	D
TcBrA4_Chr28	95000	225000	130000	C
TcBrA4_Chr28	225000	490000	265000	C

Chromosome	Start	End	Local minimum of the insulation score
TcBrA4_Chr1	140001	150000	12.881926536560059
TcBrA4_Chr1	300001	310000	3.7687251567840576
TcBrA4_Chr1	480001	490000	2.019509792327881
TcBrA4_Chr1	600001	610000	14.056875228881836
TcBrA4_Chr1	730001	740000	0.4214470088481903
TcBrA4_Chr1	820001	830000	0.6925912499427795
TcBrA4_Chr1	920001	930000	2.6653456687927246
TcBrA4_Chr1	1190001	1200000	16.777610778808594
TcBrA4_Chr1	1320001	1330000	7.572065353393555
TcBrA4_Chr1	1430001	1440000	11.701417922973633
TcBrA4_Chr1	1530001	1540000	0.42909935116767883
TcBrA4_Chr1	1640001	1650000	0.9474783539772034
TcBrA4_Chr1	1720001	1730000	0.6422088146209717
TcBrA4_Chr1	1800001	1810000	1.363781213760376
TcBrA4_Chr1	1980001	1990000	3.3445584774017334
TcBrA4_Chr1	2090001	2100000	4.292863845825195
TcBrA4_Chr1	2200001	2210000	1.2879849672317505
TcBrA4_Chr1	2390001	2400000	1.5441012382507324
TcBrA4_Chr1	2560001	2570000	0.9541520476341248
TcBrA4_Chr2	360001	370000	5.722604274749756
TcBrA4_Chr2	430001	440000	4.391151428222656
TcBrA4_Chr2	600001	610000	4.675435543060303
TcBrA4_Chr2	710001	720000	3.032881498336792
TcBrA4_Chr2	850001	860000	6.89021110534668
TcBrA4_Chr2	980001	990000	2.077282428741455
TcBrA4_Chr2	1090001	1100000	2.7542521953582764
TcBrA4_Chr2	1100001	1110000	2.7542521953582764
TcBrA4_Chr2	1130001	1140000	2.7542521953582764
TcBrA4_Chr2	1200001	1210000	0.9329438209533691
TcBrA4_Chr2	1280001	1290000	1.3757002353668213
TcBrA4_Chr2	1390001	1400000	1.9763784408569336
TcBrA4_Chr2	1530001	1540000	3.779019832611084
TcBrA4_Chr2	1650001	1660000	1.3722541332244873
TcBrA4_Chr2	1780001	1790000	5.839249134063721
TcBrA4_Chr2	1860001	1870000	6.623836040496826
TcBrA4_Chr3	140001	150000	7.498960494995117
TcBrA4_Chr3	520001	530000	5.140378475189209
TcBrA4_Chr3	660001	670000	7.032863616943359
TcBrA4_Chr3	870001	880000	0.41014334559440613
TcBrA4_Chr3	970001	980000	1.1914112567901611
TcBrA4_Chr3	1040001	1050000	0.8159472942352295
TcBrA4_Chr3	1180001	1190000	1.6959326267242432
TcBrA4_Chr3	1280001	1290000	1.0303599834442139
TcBrA4_Chr3	1380001	1390000	2.0904059410095215
TcBrA4_Chr3	1530001	1540000	0.3771129548549652
TcBrA4_Chr3	1620001	1630000	4.434718608856201
TcBrA4_Chr3	1700001	1710000	10.03842830657959
TcBrA4_Chr4	170001	180000	4.5607147216796875
TcBrA4_Chr4	250001	260000	8.257868766784668
TcBrA4_Chr4	350001	360000	0.22267675399780273
TcBrA4_Chr4	480001	490000	5.127054691314697
TcBrA4_Chr4	610001	620000	0.7612230777740479
TcBrA4_Chr4	750001	760000	5.777105331420898
TcBrA4_Chr4	850001	860000	3.9844765663146973
TcBrA4_Chr4	1020001	1030000	8.228283882141113
TcBrA4_Chr4	1200001	1210000	3.4348905086517334
TcBrA4_Chr4	1330001	1340000	4.830540180206299
TcBrA4_Chr4	1460001	1470000	7.930163383483887
TcBrA4_Chr4	1550001	1560000	3.8642969131469727
TcBrA4_Chr5	90001	100000	6.302947521209717
TcBrA4_Chr5	260001	270000	9.635812759399414
TcBrA4_Chr5	440001	450000	1.8038662672042847
TcBrA4_Chr5	600001	610000	2.43546724319458

TcBrA4_Chr5	720001	730000	3.835864305496216
TcBrA4_Chr5	760001	770000	2.334195375442505
TcBrA4_Chr5	780001	790000	2.334195375442505
TcBrA4_Chr5	850001	860000	1.694987177848816
TcBrA4_Chr5	1100001	1110000	2.2973430156707764
TcBrA4_Chr5	1330001	1340000	3.2482495307922363
TcBrA4_Chr6	180001	190000	1.7612390518188477
TcBrA4_Chr6	280001	290000	1.9322983026504517
TcBrA4_Chr6	280001	290000	1.9322983026504517
TcBrA4_Chr6	430001	440000	3.7481069564819336
TcBrA4_Chr6	490001	500000	2.5544023513793945
TcBrA4_Chr6	500001	510000	2.5544023513793945
TcBrA4_Chr6	510001	520000	2.5544023513793945
TcBrA4_Chr6	640001	650000	4.812381267547607
TcBrA4_Chr6	780001	790000	6.976593971252441
TcBrA4_Chr6	980001	990000	8.590333938598633
TcBrA4_Chr6	1090001	1100000	2.0234627723693848
TcBrA4_Chr6	1150001	1160000	1.6673996448516846
TcBrA4_Chr6	1230001	1240000	1.5728273391723633
TcBrA4_Chr7	180001	190000	1.5019687414169312
TcBrA4_Chr7	260001	270000	3.2582623958587646
TcBrA4_Chr7	350001	360000	0.5452326536178589
TcBrA4_Chr7	450001	460000	5.092929363250732
TcBrA4_Chr7	540001	550000	1.1720514297485352
TcBrA4_Chr7	660001	670000	4.939357757568359
TcBrA4_Chr7	820001	830000	0.09284976124763489
TcBrA4_Chr7	980001	990000	1.7175648212432861
TcBrA4_Chr7	1130001	1140000	1.369696855545044
TcBrA4_Chr8	100001	110000	8.500994682312012
TcBrA4_Chr8	240001	250000	0.23281921446323395
TcBrA4_Chr8	370001	380000	1.6213181018829346
TcBrA4_Chr8	570001	580000	2.5212395191192627
TcBrA4_Chr8	760001	770000	6.027675151824951
TcBrA4_Chr8	880001	890000	0.5242547392845154
TcBrA4_Chr8	1030001	1040000	9.07802677154541
TcBrA4_Chr8	1190001	1200000	1.6232458353042603
TcBrA4_Chr9	170001	180000	5.200869560241699
TcBrA4_Chr9	220001	230000	3.4269418716430664
TcBrA4_Chr9	370001	380000	3.2168970108032227
TcBrA4_Chr9	520001	530000	0.3812187910079956
TcBrA4_Chr9	570001	580000	0.6751198768615723
TcBrA4_Chr9	710001	720000	4.930308818817139
TcBrA4_Chr9	780001	790000	6.187654972076416
TcBrA4_Chr9	900001	910000	5.698855400085449
TcBrA4_Chr9	1020001	1030000	7.912715435028076
TcBrA4_Chr10	100001	110000	6.7976508140563965
TcBrA4_Chr10	190001	200000	3.4737675189971924
TcBrA4_Chr10	300001	310000	7.115309715270996
TcBrA4_Chr10	460001	470000	0.3366708755493164
TcBrA4_Chr10	640001	650000	2.5264158248901367
TcBrA4_Chr10	710001	720000	0.9841433167457581
TcBrA4_Chr10	790001	800000	1.9531046152114868
TcBrA4_Chr11	150001	160000	1.8918601274490356
TcBrA4_Chr11	290001	300000	4.364238739013672
TcBrA4_Chr11	440001	450000	2.388209819793701
TcBrA4_Chr11	550001	560000	1.2304356098175049
TcBrA4_Chr11	700001	710000	1.5013371706008911
TcBrA4_Chr11	760001	770000	2.0257420539855957
TcBrA4_Chr12	100001	110000	4.551444053649902
TcBrA4_Chr12	170001	180000	3.584333658218384
TcBrA4_Chr12	310001	320000	1.10141122341156
TcBrA4_Chr12	450001	460000	5.789191722869873
TcBrA4_Chr12	620001	630000	4.122891426086426
TcBrA4_Chr12	740001	750000	0.946941077709198

TcBrA4_Chr12	830001	840000	2.2876908779144287
TcBrA4_Chr13	160001	170000	0.5902708768844604
TcBrA4_Chr13	240001	250000	2.996407985687256
TcBrA4_Chr13	480001	490000	0.9181075096130371
TcBrA4_Chr13	620001	630000	5.003742694854736
TcBrA4_Chr13	720001	730000	6.151154518127441
TcBrA4_Chr13	830001	840000	8.812658309936523
TcBrA4_Chr14	110001	120000	4.30187463760376
TcBrA4_Chr14	280001	290000	4.712911605834961
TcBrA4_Chr14	430001	440000	1.3541899919509888
TcBrA4_Chr14	560001	570000	0.3519779145717621
TcBrA4_Chr14	660001	670000	7.134612083435059
TcBrA4_Chr14	670001	680000	7.134612083435059
TcBrA4_Chr14	670001	680000	7.134612083435059
TcBrA4_Chr15	70001	80000	5.967528343200684
TcBrA4_Chr15	180001	190000	1.2383557558059692
TcBrA4_Chr15	260001	270000	2.8816378116607666
TcBrA4_Chr15	360001	370000	1.4249967336654663
TcBrA4_Chr15	530001	540000	3.3509397506713867
TcBrA4_Chr15	630001	640000	2.8919217586517334
TcBrA4_Chr15	770001	780000	2.090916395187378
TcBrA4_Chr16	170001	180000	1.1242308616638184
TcBrA4_Chr16	260001	270000	0.7694172263145447
TcBrA4_Chr16	410001	420000	6.60719633102417
TcBrA4_Chr16	510001	520000	2.756283760070801
TcBrA4_Chr16	590001	600000	0.5234701037406921
TcBrA4_Chr16	790001	800000	2.7620720863342285
TcBrA4_Chr17	290001	300000	0.8970285058021545
TcBrA4_Chr17	420001	430000	1.8193509578704834
TcBrA4_Chr17	490001	500000	2.551408290863037
TcBrA4_Chr17	700001	710000	7.6679558753967285
TcBrA4_Chr17	810001	820000	4.047159671783447
TcBrA4_Chr18	100001	110000	15.281418800354004
TcBrA4_Chr18	200001	210000	4.582714557647705
TcBrA4_Chr18	310001	320000	5.949787616729736
TcBrA4_Chr18	490001	500000	1.4320905208587646
TcBrA4_Chr18	550001	560000	2.3929238319396973
TcBrA4_Chr18	800001	810000	3.0156283378601074
TcBrA4_Chr19	100001	110000	7.301878929138184
TcBrA4_Chr19	280001	290000	11.348791122436523
TcBrA4_Chr19	470001	480000	0.6430075168609619
TcBrA4_Chr19	620001	630000	3.1669867038726807
TcBrA4_Chr19	750001	760000	4.808713436126709
TcBrA4_Chr20	60001	70000	1.4249746799468994
TcBrA4_Chr20	140001	150000	2.6935222148895264
TcBrA4_Chr20	240001	250000	14.560078620910645
TcBrA4_Chr20	330001	340000	13.004246711730957
TcBrA4_Chr20	610001	620000	2.497251272201538
TcBrA4_Chr20	720001	730000	12.004583358764648
TcBrA4_Chr21	220001	230000	2.006544828414917
TcBrA4_Chr21	270001	280000	0.6779100298881531
TcBrA4_Chr21	340001	350000	1.3274143934249878
TcBrA4_Chr21	460001	470000	2.7137320041656494
TcBrA4_Chr21	550001	560000	2.975006103515625
TcBrA4_Chr21	670001	680000	3.2688069343566895
TcBrA4_Chr22	140001	150000	0.49540266394615173
TcBrA4_Chr22	240001	250000	1.964821696281433
TcBrA4_Chr22	350001	360000	2.3041305541992188
TcBrA4_Chr22	440001	450000	2.604184150695801
TcBrA4_Chr22	530001	540000	2.406968593597412
TcBrA4_Chr22	710001	720000	2.47308349609375
TcBrA4_Chr22	720001	730000	1.496352195739746
TcBrA4_Chr23	100001	110000	2.5465281009674072
TcBrA4_Chr23	210001	220000	8.999552726745605

TcBrA4_Chr23	390001	400000	3.382418155670166
TcBrA4_Chr23	510001	520000	1.3357298374176025
TcBrA4_Chr23	560001	570000	1.674752950668335
TcBrA4_Chr23	620001	630000	1.3886336088180542
TcBrA4_Chr23	630001	640000	1.3886336088180542
TcBrA4_Chr24	160001	170000	6.464430809020996
TcBrA4_Chr24	480001	490000	4.461129665374756
TcBrA4_Chr24	570001	580000	3.991722583770752
TcBrA4_Chr24	650001	660000	6.943575859069824
TcBrA4_Chr25	150001	160000	2.3227078914642334
TcBrA4_Chr25	270001	280000	3.1231212615966797
TcBrA4_Chr25	350001	360000	6.414115905761719
TcBrA4_Chr25	500001	510000	9.001517295837402
TcBrA4_Chr25	590001	600000	3.706737995147705
TcBrA4_Chr26	100001	110000	6.831310272216797
TcBrA4_Chr26	240001	250000	12.662208557128906
TcBrA4_Chr26	330001	340000	0.48047852516174316
TcBrA4_Chr26	400001	410000	5.797889232635498
TcBrA4_Chr26	570001	580000	3.03269100189209
TcBrA4_Chr27	110001	120000	1.9510226249694824
TcBrA4_Chr27	210001	220000	7.142602920532227
TcBrA4_Chr27	320001	330000	3.4266114234924316
TcBrA4_Chr27	370001	380000	3.057560920715332
TcBrA4_Chr27	490001	500000	3.8692336082458496
TcBrA4_Chr27	610001	620000	3.899437189102173
TcBrA4_Chr28	220001	230000	9.173993110656738
TcBrA4_Chr28	340001	350000	5.014415740966797
TcBrA4_Chr28	490001	500000	6.413994789123535
TcBrA4_Chr29	120001	130000	4.360804557800293
TcBrA4_Chr29	230001	240000	1.4334665536880493
TcBrA4_Chr29	400001	410000	3.5368566513061523
TcBrA4_Chr29	570001	580000	3.698395252227783
TcBrA4_Chr30	250001	260000	5.651714324951172
TcBrA4_Chr30	360001	370000	7.116982936859131
TcBrA4_Chr30	500001	510000	3.7966606616973877
TcBrA4_Chr31	80001	90000	0.18757756054401398
TcBrA4_Chr31	170001	180000	5.655488967895508
TcBrA4_Chr31	360001	370000	1.3751540184020996
TcBrA4_Chr31	500001	510000	4.1225128173828125
TcBrA4_Chr32	100001	110000	0.9548206329345703
TcBrA4_Chr32	260001	270000	2.6776721477508545
TcBrA4_Chr32	370001	380000	5.311502933502197
TcBrA4_Chr32	520001	530000	1.94411039352417
TcBrA4_Chr33	120001	130000	3.5935914516448975
TcBrA4_Chr33	330001	340000	2.6565701961517334
TcBrA4_Chr34	80001	90000	0.3140130341053009
TcBrA4_Chr35	160001	170000	2.789161443710327
TcBrA4_Chr36	100001	110000	3.9594321250915527
TcBrA4_Chr38	70001	80000	2.4073736667633057
TcBrA4_Chr39	60001	70000	0.5675839185714722
TcBrA4_Chr40	70001	80000	1.3134182691574097
TcBrA4_Chr41	70001	80000	1.3134182691574097

Chromosome	Chr length (nt)	Core length (nt)	Disruptive length (nt)	Core (%)	Disruptive (%)	Chr classification
TcBrA4_Chr1	2738928	2455197	283731	89,6	10,4	Core
TcBrA4_Chr2	1986034	459662	1526372	23,1	76,9	Mixed
TcBrA4_Chr3	1768708	1380816	387892	78,1	21,9	Mixed
TcBrA4_Chr4	1676910	1546025	130885	92,2	7,8	Core
TcBrA4_Chr5	1492459	1370302	122157	91,8	8,2	Core
TcBrA4_Chr6	1421388	581649	839739	40,9	59,1	Mixed
TcBrA4_Chr7	1369405	1324118	45287	96,7	3,3	Core
TcBrA4_Chr8	1336822	1256979	79843	94,0	6,0	Core
TcBrA4_Chr9	1155514	405350	750164	35,1	64,9	Mixed
TcBrA4_Chr10	1097740	1097740	0	100,0	0,0	Core
TcBrA4_Chr11	1076255	529148	547107	49,2	50,8	Mixed
TcBrA4_Chr12	1041209	219175	822034	21,1	78,9	Mixed
TcBrA4_Chr13	982025	762069	219956	77,6	22,4	Mixed
TcBrA4_Chr14	975858	803853	172005	82,4	17,6	Core
TcBrA4_Chr15	969620	969620	0	100,0	0,0	Core
TcBrA4_Chr16	927191	812500	114691	87,6	12,4	Core
TcBrA4_Chr17	914771	784558	130213	85,8	14,2	Core
TcBrA4_Chr18	909794	905465	4329	99,5	0,5	Core
TcBrA4_Chr19	902532	485778	416754	53,8	46,2	Mixed
TcBrA4_Chr20	846588	468747	377841	55,4	44,6	Mixed
TcBrA4_Chr21	820352	146334	674018	17,8	82,2	Disruptive
TcBrA4_Chr22	815970	682430	133540	83,6	16,4	Core
TcBrA4_Chr23	812063	677468	134595	83,4	16,6	Core
TcBrA4_Chr24	778187	659375	118812	84,7	15,3	Core
TcBrA4_Chr25	742617	84153	658464	11,3	88,7	Disruptive
TcBrA4_Chr26	731747	532750	198997	72,8	27,2	Mixed
TcBrA4_Chr27	716856	552590	164266	77,1	22,9	Mixed
TcBrA4_Chr28	711759	624700	87059	87,8	12,2	Core
TcBrA4_Chr29	660991	420076	240915	63,6	36,4	Mixed
TcBrA4_Chr30	660739	417538	243201	63,2	36,8	Mixed
TcBrA4_Chr31	601716	473419	128297	78,7	21,3	Mixed
TcBrA4_Chr32	590954	333732	257222	56,5	43,5	Mixed
TcBrA4_Chr33	574917	293252	281665	51,0	49,0	Mixed
TcBrA4_Chr34	285003	170555	114448	59,8	40,2	Mixed
TcBrA4_Chr35	243420	0	243420	0,0	100,0	Disruptive
TcBrA4_Chr36	231406	0	231406	0,0	100,0	Disruptive
TcBrA4_Chr37	216495	216495	0	100,0	0,0	Core
TcBrA4_Chr38	166627	0	166627	0,0	100,0	Disruptive
TcBrA4_Chr39	160921	0	160921	0,0	100,0	Disruptive
TcBrA4_Chr40	155078	145068	10010	93,5	6,5	Core
TcBrA4_Chr41	146158	39978	106180	27,4	72,6	Mixed
TcBrA4_Chr42	141754	0	141754	0,0	100,0	Disruptive
TcBrA4_Chr43	141550	112369	29181	79,4	20,6	Mixed

Scaffold	Chr length (nt)	Core length (nt)	Disruptive length (nt)	Core (%)	Disruptive (%)	Sca classification
PRFA01000001	1645565	201455	1444110	12,2	87,8	Disruptive
PRFA01000002	1352947	1280760	72187	94,7	5,3	Core
PRFA01000003	1227947	144460	1083487	11,8	88,2	Disruptive
PRFA01000004	943259	943259	0	100,0	0,0	Core
PRFA01000005	917542	66637	850905	7,3	92,7	Disruptive
PRFA01000006	814295	814295	0	100,0	0,0	Core
PRFA01000007	765515	765515	0	100,0	0,0	Core
PRFA01000008	744599	408815	335784	54,9	45,1	Mixed
PRFA01000009	684680	635132	49548	92,8	7,2	Core
PRFA01000010	619240	619240	0	100,0	0,0	Core
PRFA01000011	599870	271808	328062	45,3	54,7	Mixed
PRFA01000012	595721	500749	94972	84,1	15,9	Core
PRFA01000013	569744	459507	110237	80,7	19,3	Core
PRFA01000014	561960	512514	49446	91,2	8,8	Core
PRFA01000015	561800	68858	492942	12,3	87,7	Disruptive
PRFA01000016	558959	558959	0	100,0	0,0	Core
PRFA01000017	552463	552463	0	100,0	0,0	Core
PRFA01000018	549721	549721	0	100,0	0,0	Core
PRFA01000019	536809	536809	0	100,0	0,0	Core
PRFA01000020	533751	533751	0	100,0	0,0	Core
PRFA01000021	518462	440572	77890	85,0	15,0	Core
PRFA01000022	496439	410704	85735	82,7	17,3	Core
PRFA01000023	492210	492210	0	100,0	0,0	Core
PRFA01000024	489592	392041	97551	80,1	19,9	Core
PRFA01000025	489492	443513	45979	90,6	9,4	Core
PRFA01000026	460657	396639	64018	86,1	13,9	Core
PRFA01000027	458025	256236	201789	55,9	44,1	Mixed
PRFA01000028	455168	431903	23265	94,9	5,1	Core
PRFA01000029	445262	54910	390352	12,3	87,7	Disruptive
PRFA01000030	440675	440675	0	100,0	0,0	Core

GeneID	log2FoldChange	padj	Compartment
C4B63_44g228	4.97683808609184	2.2057888272196e-291	Core
C4B63_2g185	4.10694481005404	4.66528211700573e-226	Core
C4B63_4g189	5.61806805494159	1.31030935872686e-212	Core
C4B63_2g189	4.23514272631205	4.02367730302492e-211	Core
C4B63_16g183	-4.82388488439087	7.98138732970238e-209	Core
C4B63_32g172	-6.15124983824153	1.30988902471078e-202	Disruptive
C4B63_42g60	4.41004093184352	1.28236654855537e-183	Core
C4B63_57g131	4.52553920562234	5.39584055891465e-183	Core
C4B63_44g229	4.29519217035952	1.32702296423289e-172	Core
C4B63_54g69	-3.47961579366788	6.1703309814169e-171	Core
C4B63_13g352	4.09556037107934	1.39637571886734e-166	Core
C4B63_21g343	-4.49716826698989	3.69094806249872e-165	Core
C4B63_255g20	3.04760856054795	4.96368908489312e-161	Core
C4B63_60g203	-5.08638231657299	3.66964210600366e-160	Disruptive
C4B63_271g8	-6.51239234516941	9.59598989073564e-160	Core
C4B63_43g199	-6.37359273345167	1.08939543391025e-155	Core
C4B63_361g13	3.55262927913909	3.54615696219842e-146	Core
C4B63_16g197	-4.08746750554516	1.22408528835677e-145	Disruptive
C4B63_22g743c	4.44474536664537	4.57909867036441e-144	Core
C4B63_11g40	4.42564199926507	4.62134080677293e-142	Core
C4B63_405g4	3.70434391150269	9.63780221105805e-139	Core
C4B63_294g11	3.91943492628056	7.58363763375505e-138	Core
C4B63_101g12	6.56850514431151	3.68625269588421e-136	Core
C4B63_193g13	4.8076622395496	1.63518386052051e-132	Core
C4B63_193g16	4.58174586148636	1.52880391043066e-131	Core
C4B63_101g3	2.57687697427893	1.97452700035279e-131	Core
C4B63_33g179	-5.70649232492187	6.69864258712714e-130	Core
C4B63_260g23	-4.57520535552566	8.12025178299091e-129	Disruptive
C4B63_405g10	3.25433824157292	2.19542865627844e-128	Core
C4B63_102g68	3.32411927331363	1.86821367935601e-127	Core
C4B63_113g41	4.93368838074091	9.52785207523696e-121	Core
C4B63_336g4	2.98932934032157	8.17042710261383e-120	Core
C4B63_12g161	4.76902338155039	5.34456496468993e-118	Core
C4B63_101g6	3.28237126906458	1.85083806777224e-117	Core
C4B63_21g56	3.05847856788346	4.22246946735778e-114	Core
C4B63_92g110	3.83325076022448	3.41112267816241e-112	Core
C4B63_135g24	-3.81809720800302	8.00490673606982e-112	Disruptive
C4B63_68g79	-4.94972940657144	1.9613796375013e-110	Core
C4B63_22g773c	4.55922938806321	4.96605126449062e-109	Core
C4B63_32g43	3.46829320715046	3.75606690557478e-107	Core
C4B63_56g86	2.93851859135084	2.75112830282022e-105	Core
C4B63_61g135	3.22608331409844	3.36385264678545e-105	Core
C4B63_70g116	2.91483428451469	7.42813032617251e-105	Core
C4B63_201g18	-5.7382658740771	3.73968588595246e-104	Core
C4B63_73g11	-3.25286936698531	2.21988751009302e-103	Disruptive
C4B63_351g27c	4.23636865482726	5.13572664837e-103	Core
C4B63_11g29	3.29778657494768	1.11933164858497e-100	Core
C4B63_17g257	2.75220915896024	2.94555479637349e-100	Core
C4B63_8g560	2.72915680151529	1.50037296316618e-99	Core
C4B63_113g42	4.98317524102908	2.22348716499385e-99	Core
C4B63_41g261	-4.04924968328755	5.30015483867775e-97	Disruptive
C4B63_164g14	-4.32803113376679	2.99527059013079e-96	Disruptive
C4B63_44g223	2.7122375483802	1.61808303456726e-95	Core
C4B63_2g317	-5.42322652292873	5.19643207603077e-95	Disruptive
C4B63_40g138	-4.40242148707328	6.64530013127154e-95	Disruptive
C4B63_93g73	2.76520077689704	9.14698808986724e-95	Core
C4B63_207g15	3.45300884063967	1.53155223443926e-94	Core
C4B63_11g67	3.00538833509889	1.32912779677759e-93	Core
C4B63_75g105	-4.57849683028016	2.11118394445681e-92	Disruptive
C4B63_12g175	4.25920740731987	4.53187304292781e-92	Core
C4B63_167g33	-4.10060684268752	6.229054226753e-92	Disruptive
C4B63_75g72	-3.97706875817233	6.64789229736851e-92	Disruptive
C4B63_58g229	-2.67430800476365	5.30993238557902e-91	Disruptive

C4B63_77g40	-4.83092446990198	1.2197197649295e-90	Disruptive
C4B63_51g207	3.17254139292382	4.78979697783252e-90	Core
C4B63_68g91	-4.13037172183647	5.23674207457577e-89	Disruptive
C4B63_9g383	3.91794519652388	1.73077021659359e-88	Core
C4B63_79g110	4.11250393691092	4.13856292890522e-88	Core
C4B63_34g269	3.4515856064882	4.58707779867057e-88	Disruptive
C4B63_158g42	-5.69670843901216	4.67109426018705e-88	Core
C4B63_43g170	2.2658312370935	6.88714732207166e-88	Core
C4B63_14g28	2.68983386662457	1.73607803215908e-87	Core
C4B63_148g10	-4.76466036088204	7.38142741022891e-87	Disruptive
C4B63_207g13	3.5613750874729	9.96487296716975e-87	Core
C4B63_63g35	3.82395109686665	1.70279424015331e-86	Core
C4B63_251g17	2.58880369040926	1.70279424015331e-86	Core
C4B63_203g15	2.56667028646155	3.30674152615196e-86	Core
C4B63_17g205	-4.42894031158985	1.20783835781506e-84	Disruptive
C4B63_7g299	5.11949084774389	1.34379440857556e-84	Core
C4B63_84g32	-4.73307043002488	2.08233003937176e-84	Disruptive
C4B63_119g19	-4.73280362809976	5.33980595571124e-84	Disruptive
C4B63_87g8	4.53555994299568	1.31702691628928e-83	Core
C4B63_34g324	-6.17086431989953	5.00561429998989e-83	Core
C4B63_87g7	2.59945451780667	2.00761977875764e-82	Core
C4B63_33g192	-5.22223612184197	8.9218204047683e-82	Core
C4B63_145g23	-3.98265666793961	1.63891471259018e-79	Disruptive
C4B63_41g263	-4.10507564573137	2.25126621877797e-79	Disruptive
C4B63_46g58	3.4506051038972	2.75549451948188e-79	Core
C4B63_148g15	-5.24671605466944	6.18934748810813e-79	Disruptive
C4B63_86g95	-5.34375973891954	7.13590926048614e-79	Disruptive
C4B63_124g40	-4.92270263968302	2.28179999799861e-78	Disruptive
C4B63_87g138	-3.35928848564825	2.37442178711931e-78	Disruptive
C4B63_144g26	3.62648794296066	2.45571885446658e-78	Core
C4B63_34g275	3.87000880687084	3.15396038089233e-78	Disruptive
C4B63_75g77	-3.7705702401323	1.1622343161786e-77	Disruptive
C4B63_21g165	-3.77333080873318	2.00239015300032e-77	Disruptive
C4B63_119g11	-5.4517687990418	4.67417608290585e-77	Disruptive
C4B63_1g368	-5.60522609768762	1.2670856320085e-75	Disruptive
C4B63_119g31	-4.66565512158	3.89594614084055e-75	Disruptive
C4B63_41g300	-5.38061879700216	1.52213170236638e-74	Disruptive
C4B63_538g1	-5.40412051784159	3.24958144673696e-73	Core
C4B63_34g124	3.53055021847654	6.83983173572551e-73	Core
C4B63_384g22	-4.62070177704841	7.92020013024866e-73	Disruptive
C4B63_12g157	4.14436160266042	8.53058664120747e-73	Core
C4B63_13g117	2.03346208593543	1.49466293510148e-72	Core
C4B63_34g1236c	3.98459660624434	2.78931620284703e-72	Disruptive
C4B63_2g323	-4.98972058214188	2.51183238519805e-71	Disruptive
C4B63_8g182	-4.11733598200719	1.09418850016702e-70	Core
C4B63_35g99	3.17280906922753	1.18579522596706e-70	Core
C4B63_13g280	-4.02914380003032	1.20841951717462e-70	Disruptive
C4B63_72g38	-4.32316864886992	2.68174886766238e-70	Disruptive
C4B63_97g13	3.38584856209971	1.52364462901292e-69	Core
C4B63_387g21	-3.00824342430428	6.46825937271729e-69	Disruptive
C4B63_1g128c	4.06819653527965	6.83090094454999e-69	Core
C4B63_22g28	2.75347452152565	7.97380264376154e-69	Core
C4B63_34g1226c	3.95788964493286	2.23349370223653e-68	Disruptive
C4B63_131g42	-3.49018038034818	3.89649194905224e-68	Disruptive
C4B63_105g53	-4.77030783209821	5.50256387116557e-68	Disruptive
C4B63_2g324	-4.98448519283764	3.71974500694891e-67	Disruptive
C4B63_2g199	3.58937017475328	6.49138249966275e-67	Core
C4B63_34g1237c	3.69754082110062	1.35957887936551e-66	Disruptive
C4B63_18g167	2.8680408896817	2.16532050880758e-66	Core
C4B63_13g292	-4.54682245182833	2.4858953994236e-66	Disruptive
C4B63_153g14	-3.55427462812853	1.46127260849169e-65	Disruptive
C4B63_34g1224c	3.89044959208322	2.24610775436092e-65	Disruptive
C4B63_34g122	-5.99935375275657	2.87904104466503e-65	Core
C4B63_5g433	-4.7926503908424	5.17714662048126e-65	Disruptive

C4B63_21g271	2.87459364699529	6.17685076530224e-65	Core
C4B63_77g60	-2.77908958735707	6.31866594523276e-65	Disruptive
C4B63_361g12	3.98849481971998	6.93092159281615e-65	Core
C4B63_3g1125	3.35481042044503	2.27014233515632e-64	Core
C4B63_41g212	2.14052009722557	2.27014233515632e-64	Core
C4B63_51g234	-3.22861610060638	6.33812000082971e-64	Disruptive
C4B63_73g69	-4.2093708389805	7.96032818956629e-64	Core
C4B63_13g14	-3.97816762128648	2.32647709433828e-63	Disruptive
C4B63_5g493	-4.93264958556785	4.09275144787283e-63	Disruptive
C4B63_46g80	3.75845055029073	7.46923567970576e-63	Core
C4B63_43g92	-4.2553304429839	1.15458112471803e-62	Disruptive
C4B63_29g195	-4.34400549055723	1.26647928764548e-62	Core
C4B63_8g77	-3.58069549725738	1.80306524136718e-62	Disruptive
C4B63_333g14	1.49863258520374	1.80306524136718e-62	Core
C4B63_16g155	2.52801318857365	1.87573146575183e-62	Core
C4B63_204g16	-4.70095077931034	3.10443807649608e-62	Disruptive
C4B63_130g12	-3.0723058655154	5.74275937262275e-62	Core
C4B63_34g1233c	4.23806977111864	8.82411125741547e-62	Disruptive
C4B63_77g53	-3.90411728990906	1.235631733787e-61	Disruptive
C4B63_2g547	3.14094248739344	2.4610206448367e-61	Core
C4B63_34g1223c	4.4378882332804	2.83345654858385e-61	Disruptive
C4B63_84g69	3.32781807131506	5.04876663332481e-61	Core
C4B63_110g16	1.99228845119655	9.00265960049533e-61	Core
C4B63_34g1244c	4.44475021845181	1.09853847227073e-60	Disruptive
C4B63_145g21	-3.30736519678539	1.34457269818697e-60	Disruptive
C4B63_42g262	-4.82689741231222	1.65775271610901e-60	Disruptive
C4B63_34g245	2.5013735906372	2.39711221628137e-60	Disruptive
C4B63_1g788	-5.08476859669934	4.22357573676887e-60	Disruptive
C4B63_67g58	-4.21178803561707	6.51513229506504e-60	Disruptive
C4B63_30g163	2.32482325050896	8.48439620575407e-60	Core
C4B63_27g302	-4.47259293397014	1.15081537401351e-59	Disruptive
C4B63_105g64	-4.06262313791281	1.40923642968242e-59	Disruptive
C4B63_29g211	-4.99478074432512	2.06313615782776e-59	Core
C4B63_16g181	-3.11311090395296	3.08276172591491e-59	Disruptive
C4B63_384g21	-4.76593586066858	3.10061987998922e-59	Disruptive
C4B63_33g156	-4.32492049590296	3.59849343747403e-59	Core
C4B63_22g37	2.75833626508759	6.05912153013734e-59	Core
C4B63_119g5	-4.35765288213949	6.52139034466718e-59	Disruptive
C4B63_69g57	3.29399594772739	8.0048666979061e-59	Core
C4B63_5g131	-5.25831814290304	1.1640487529618e-58	Disruptive
C4B63_32g51	3.90555091029105	1.45392827044233e-58	Core
C4B63_2g765	2.07983647366169	1.96215500854779e-58	Core
C4B63_8g117	-3.12920173629714	4.77635803450028e-58	Core
C4B63_8g123	-2.67639460431603	6.7797516558275e-58	Disruptive
C4B63_2g316	-5.29011453230357	9.27505536415082e-58	Disruptive
C4B63_19g43	2.860477847467	1.0248599210163e-57	Core
C4B63_39g375	-4.47180347154903	1.187279641702e-57	Disruptive
C4B63_43g225	3.91123380753025	1.1939681779569e-57	Core
C4B63_145g11	-3.92040534598278	1.82464653244362e-57	Disruptive
C4B63_23g158	-3.50663923289477	3.05750177562724e-57	Disruptive
C4B63_27g337	-5.56605441936112	5.04573129093798e-57	Disruptive
C4B63_157g40	-2.392894643516	5.82899260685515e-57	Disruptive
C4B63_371g10	-3.51366780003048	1.53764468202692e-56	Disruptive
C4B63_12g170	4.47933047213174	1.96464779028351e-56	Core
C4B63_7g298	5.0612592974653	2.45287182791188e-56	Core
C4B63_63g110	2.73843293461093	5.41983320993473e-56	Core
C4B63_105g66	-5.02975812401373	6.49155670226815e-56	Disruptive
C4B63_21g268	2.89522972788014	1.09640365520028e-55	Core
C4B63_6g537	1.94670664931558	1.11886183774237e-55	Core
C4B63_63g102	2.6646096468823	2.01562594808637e-55	Core
C4B63_155g10	2.84772010425301	3.38466673616357e-55	Disruptive
C4B63_4g334	-4.03432983205062	4.08300533596239e-55	Core
C4B63_258g29	2.75110181734317	6.1132653010398e-55	Core
C4B63_2g661	-4.75729562935732	1.67707296603672e-54	Disruptive

C4B63_1g473	-3.2716357525962	3.94150185799502e-54	Disruptive
C4B63_105g45	-4.30696583233802	5.1543153103001e-54	Disruptive
C4B63_109g41	-5.54382731111243	5.72568359507777e-54	Core
C4B63_145g8	-3.26078262432107	1.77276723291233e-53	Disruptive
C4B63_27g278	-4.8405576106082	1.95378517111478e-53	Disruptive
C4B63_408g15	-4.04116245501527	4.11974651670135e-53	Disruptive
C4B63_232g21	2.86742224707191	6.00551849734705e-53	Disruptive
C4B63_212g8	4.13074878063558	6.21427698173311e-53	Core
C4B63_277g2	2.27418648500235	9.77047645860483e-53	Core
C4B63_45g173	3.43851752356975	6.22976656937271e-52	Core
C4B63_22g32	2.90797341982763	7.64732353022323e-52	Core
C4B63_46g181	-4.38858409706457	1.47393185216406e-51	Disruptive
C4B63_119g10	-5.3363527731611	5.46187098053098e-51	Disruptive
C4B63_22g137	-2.91951455057393	1.03556235022193e-50	Disruptive
C4B63_9g480	2.28609791991783	1.06124907704774e-50	Core
C4B63_111g19	-2.27666449581455	1.41554829386764e-50	Disruptive
C4B63_39g111	-4.98326532547078	1.7852361966429e-50	Disruptive
C4B63_258g21	3.47583857874013	2.45824515648866e-50	Core
C4B63_49g192	2.49884153606631	2.49206150900748e-50	Core
C4B63_5g426	-3.13853249281524	2.81283155547357e-50	Disruptive
C4B63_20g316	2.52781272978393	7.21108317132091e-50	Core
C4B63_38g303	-4.20060259718913	1.6818731299057e-49	Disruptive
C4B63_162g7	-2.97316229616951	1.8522746712486e-49	Disruptive
C4B63_46g62	3.52956015032204	1.91344709108297e-49	Core
C4B63_17g108	-1.90228885681895	2.31699590268018e-49	Core
C4B63_49g210	-3.43656086986079	2.58750434806329e-49	Disruptive
C4B63_161g32	3.6569811439772	3.4562104972467e-49	Core
C4B63_70g50	2.47107899584779	3.55011998487279e-49	Core
C4B63_312g7	4.21461198913138	4.74936891197554e-49	Core
C4B63_39g370	-4.42852034576185	2.45409567370516e-48	Disruptive
C4B63_1g527	-2.4325120009429	3.34947803999244e-48	Disruptive
C4B63_3g454	-3.54615735652278	4.97306523138154e-48	Disruptive
C4B63_166g26	-2.50609821834411	5.4684100600482e-48	Disruptive
C4B63_22g322	2.56419062188478	6.57501729967946e-48	Core
C4B63_22g172	1.18949722342803	1.06922547197354e-47	Core
C4B63_25g320	-2.61287129963439	1.38887325558464e-47	Core
C4B63_24g260	-4.99195730501889	1.51437889147836e-47	Disruptive
C4B63_10g490	1.76901496429567	2.9664395190493e-47	Core
C4B63_35g363	-4.3095654682946	7.40161860074116e-47	Disruptive
C4B63_46g66	2.45617412162518	1.08019561175963e-46	Core
C4B63_54g96	-3.18471993281585	1.37723849114147e-46	Disruptive
C4B63_2g183	3.57230133244786	3.31694260458634e-46	Core
C4B63_58g227	-4.92690070442058	3.38224777704694e-46	Disruptive
C4B63_14g229	-3.59711016389597	3.48850023038698e-46	Disruptive
C4B63_10g296	-1.48452136336734	5.07830851594816e-46	Core
C4B63_16g202	-3.11727814909403	5.5766671782781e-46	Disruptive
C4B63_417g17	-5.12421376330335	6.70949750728603e-46	Disruptive
C4B63_62g3	-4.40336435333151	7.85530894235464e-46	Disruptive
C4B63_22g206	-3.69630560824831	9.35757976226478e-46	Disruptive
C4B63_30g300	-2.08234591492411	1.0464177168841e-45	Disruptive
C4B63_34g248	3.97271622346324	1.16617179201921e-45	Disruptive
C4B63_5g432	-5.03064573785952	1.32163264360975e-45	Disruptive
C4B63_10g266	-4.51116787645505	1.66562505108316e-45	Disruptive
C4B63_159g15	-3.53962180860923	2.31879399702179e-45	Disruptive
C4B63_78g46	2.67219370630857	3.22698004462917e-45	Core
C4B63_6g394	2.84422478189005	3.4349742052062e-45	Core
C4B63_13g5	-4.03386591033091	4.46292519164587e-45	Disruptive
C4B63_46g75	2.21315734495977	5.21902960316483e-45	Core
C4B63_170g1	4.23811863019754	6.32002506693401e-45	Core
C4B63_37g387	-3.41713891033655	9.23077263365226e-45	Disruptive
C4B63_77g67	-2.94949149906013	1.0757982554395e-44	Disruptive
C4B63_24g202	2.33108446362197	1.25293484645072e-44	Core
C4B63_155g17	-3.44105006426624	1.55546268330846e-44	Disruptive
C4B63_43g95	-3.83217818374674	2.28388093185513e-44	Disruptive

C4B63_212g4	4.75024452845148	2.3566336084421e-44	Core
C4B63_37g419	2.09064523100415	4.2525347813982e-44	Core
C4B63_39g364	-4.36186185842289	4.34286777119519e-44	Disruptive
C4B63_28g149	2.82259127062699	1.08774095699391e-43	Core
C4B63_22g131	-3.71136536786342	1.54762144810726e-43	Disruptive
C4B63_34g263	4.1668215783349	1.8936035371295e-43	Disruptive
C4B63_60g164	3.27864399304489	3.08457059319434e-43	Core
C4B63_396g6	2.79637061381668	3.46471293317506e-43	Core
C4B63_153g5	2.52930041763956	3.80240463811555e-43	Core
C4B63_22g738c	3.4337080919445	4.19910360159851e-43	Core
C4B63_109g42	3.68175824134042	5.87149932364525e-43	Core
C4B63_35g105	-1.71951472867878	6.04476078470611e-43	Core
C4B63_246g1	-4.5727776459651	7.77801927695054e-43	Core
C4B63_35g193	-2.35735185259187	8.41256084619018e-43	Disruptive
C4B63_9g443	2.48722224304471	1.01417005794074e-42	Core
C4B63_210g26	3.65429676858472	1.86046778743008e-42	Core
C4B63_70g119	3.12372386964632	2.93300844556079e-42	Core
C4B63_375g1	2.93687696097901	3.93265716514235e-42	Disruptive
C4B63_81g64	2.43002501142555	4.12394476006953e-42	Core
C4B63_231g14	-4.55016942218905	6.27368172027156e-42	Disruptive
C4B63_5g167	-4.43749816274892	7.4316599690659e-42	Disruptive
C4B63_182g13	2.20423696284152	8.53806089457387e-42	Core
C4B63_19g12	-3.35086402757113	1.52489971277379e-41	Core
C4B63_109g67	3.56194682099326	1.86910083337481e-41	Core
C4B63_27g282	-3.4658226697001	2.32653375443961e-41	Disruptive
C4B63_158g35	-4.89561966859421	2.49150917261662e-41	Disruptive
C4B63_1g81	-3.18335448036019	4.77976532211496e-41	Disruptive
C4B63_45g193	3.17210918878512	5.62791430565083e-41	Core
C4B63_37g133	-3.7592563823427	8.09804780151024e-41	Disruptive
C4B63_226g20	-3.55798446887543	8.25999263072444e-41	Disruptive
C4B63_60g179	-2.7200059869171	1.12345641339619e-40	Disruptive
C4B63_12g220	2.16840311669394	1.15397553351832e-40	Core
C4B63_12g232	1.83371875029032	1.57349902231603e-40	Core
C4B63_23g110	-1.77331661975982	1.59790658432717e-40	Core
C4B63_35g334	-3.76687826873689	1.64200609863177e-40	Disruptive
C4B63_16g315	2.18112312610831	1.82413148485316e-40	Core
C4B63_68g74	-3.20051933991178	2.12860886703569e-40	Disruptive
C4B63_9g279	1.62631367927733	2.21235730331273e-40	Core
C4B63_12g162	4.69921608040909	2.23718869482118e-40	Core
C4B63_22g125	-3.24601893961376	2.97813547002823e-40	Disruptive
C4B63_13g310	2.02551031484785	3.46613718271625e-40	Core
C4B63_84g54	2.05886928784848	3.80186735346747e-40	Core
C4B63_48g144	2.14811263808074	4.72872201911012e-40	Core
C4B63_26g261	1.69772154593681	4.7953878408588e-40	Core
C4B63_93g12	-3.48584157496392	6.13338539962836e-40	Disruptive
C4B63_4g462	3.30934316180056	6.86651248396982e-40	Core
C4B63_131g47	-2.74747483661682	6.86651248396982e-40	Disruptive
C4B63_1g130c	3.54446437068881	7.6344014231386e-40	Core
C4B63_31g250	-2.62047812052263	8.70738604118378e-40	Disruptive
C4B63_147g50	-2.84137872967027	1.00019495110983e-39	Disruptive
C4B63_455g9	-2.65183277538486	1.05211341867013e-39	Disruptive
C4B63_110g48	2.79662253774619	1.4217498861474e-39	Core
C4B63_11g131	-3.60805846080009	2.38138139647122e-39	Disruptive
C4B63_88g102	-3.66622242337086	2.60536409973763e-39	Disruptive
C4B63_147g63	-4.03836482809485	3.92141406522263e-39	Disruptive
C4B63_7g438	2.79599825553957	5.59955549266501e-39	Core
C4B63_104g102	3.6895746962619	7.73837268007966e-39	Core
C4B63_52g312c	2.37347803300297	8.42968198969734e-39	Core
C4B63_447g6	-4.50865828132591	9.33626725933933e-39	Disruptive
C4B63_9g382	3.02551195192372	1.15623330855466e-38	Core
C4B63_3g1022	-3.67416275376146	1.26413676504422e-38	Disruptive
C4B63_34g137	-2.61170475917199	1.48348040274957e-38	Disruptive
C4B63_36g86	-3.88090771063115	2.57411464975472e-38	Disruptive
C4B63_105g48	-3.52588178924619	3.3515765329879e-38	Disruptive

C4B63_28g224	2.06755375458315	3.69845402920979e-38	Core
C4B63_24g283	-4.11979900891629	6.83707111201623e-38	Disruptive
C4B63_459g9	2.63182934089896	8.58559851881607e-38	Core
C4B63_12g171	1.75168862350261	1.06959805577981e-37	Core
C4B63_6g435	2.12018302911268	1.25510141846309e-37	Core
C4B63_156g20	-4.25725398179036	1.66385798294611e-37	Disruptive
C4B63_39g154	-4.59445321128536	1.86444981569965e-37	Disruptive
C4B63_43g229	-3.99305085934728	2.17751151381324e-37	Disruptive
C4B63_80g4	2.22962598875972	2.4317115809443e-37	Core
C4B63_371g9	-3.70057050337986	2.90290869472206e-37	Disruptive
C4B63_39g55	-4.62675862711848	3.05211293713614e-37	Disruptive
C4B63_43g175	1.95561363913944	3.4486615673262e-37	Core
C4B63_360g18	-3.34939235948164	3.67907394703888e-37	Disruptive
C4B63_22g135	-3.52032965879937	6.56228035738482e-37	Disruptive
C4B63_69g74	1.85586555220414	1.08705650643438e-36	Core
C4B63_227g12	2.89607338801062	1.42294669443005e-36	Core
C4B63_196g38	-4.86477913810283	1.54330713990927e-36	Disruptive
C4B63_2g712	2.60614030134281	1.60024818864153e-36	Core
C4B63_12g221	2.12251060570094	1.67046980385321e-36	Core
C4B63_21g240	1.97208034930044	1.9825614973898e-36	Core
C4B63_42g193	-1.63964955702546	3.56311409246647e-36	Core
C4B63_3g554	2.56734977984879	4.01532765108459e-36	Core
C4B63_39g365	-4.44517034144768	4.53973913494656e-36	Disruptive
C4B63_94g87	-3.49572352632069	4.81818724497611e-36	Disruptive
C4B63_13g282	-2.52614607716312	4.87680467221677e-36	Disruptive
C4B63_32g281	-2.62269736356332	4.92618801967631e-36	Disruptive
C4B63_30g113	-4.33084730874987	5.4623106694446e-36	Core
C4B63_12g226	2.3896636308819	5.97489569229774e-36	Core
C4B63_47g128	-3.84689379843336	6.10047516975303e-36	Disruptive
C4B63_34g297	3.04791679557774	6.21257056411936e-36	Core
C4B63_28g116	2.45565320966074	8.19885342051354e-36	Core
C4B63_23g10	-3.63716742946664	9.54691756380894e-36	Disruptive
C4B63_194g18	1.91847182661768	1.33409365172895e-35	Core
C4B63_1g748	-3.3038886766244	1.38464303630659e-35	Disruptive
C4B63_281g6	-3.242777757731	1.50854350771855e-35	Core
C4B63_4g490	2.16120614689015	1.52903471122286e-35	Core
C4B63_13g132	2.22794019178179	1.64397993163605e-35	Core
C4B63_47g92	-3.23005655686224	1.79678645952727e-35	Disruptive
C4B63_119g1	-4.0236966183889	1.95879001509047e-35	Disruptive
C4B63_82g89	2.49591946093394	2.06323081685507e-35	Core
C4B63_9g478	-3.41276092713908	2.10909852831417e-35	Disruptive
C4B63_4g269	-2.0506831871462	2.27130791610765e-35	Core
C4B63_34g127	-2.62266262282177	4.63703328404909e-35	Disruptive
C4B63_20g11	-2.06953783154066	6.51353618175515e-35	Core
C4B63_21g180	-3.38722314355811	6.85295843022131e-35	Disruptive
C4B63_387g20	-2.79041699978848	6.97395487304781e-35	Disruptive
C4B63_25g202	2.23072589439165	7.22646687614523e-35	Core
C4B63_63g143	-3.53604985997205	7.22646687614523e-35	Disruptive
C4B63_351g33c	-2.53887698682947	8.05493536678267e-35	Core
C4B63_205g52	-4.05685000143915	9.54325795850743e-35	Disruptive
C4B63_43g230	-4.0918654340305	1.03324917637345e-34	Disruptive
C4B63_558g4	-3.85081522807259	1.17531733500357e-34	Disruptive
C4B63_82g114	-3.29444553392404	1.19262629553374e-34	Disruptive
C4B63_187g13	-2.67475236901947	1.27361449197064e-34	Disruptive
C4B63_154g5	-3.56416336943064	1.5278907541267e-34	Disruptive
C4B63_43g87	-2.67553820610246	1.66930708856378e-34	Disruptive
C4B63_300g22	-5.81573214702183	1.77462681083515e-34	Core
C4B63_52g163	1.8568575165228	2.50647263416055e-34	Core
C4B63_34g284	-2.23793085017106	3.49247793989631e-34	Core
C4B63_28g222	3.56595982402704	4.62587184744498e-34	Core
C4B63_7g81	1.44569611055612	7.97534184918142e-34	Core
C4B63_431g8	3.04402014658162	8.16639357730337e-34	Core
C4B63_24g250	-2.95550579745894	9.13530722496099e-34	Disruptive
C4B63_5g376	-4.35182915753301	1.18451421081535e-33	Disruptive

C4B63_70g139	5.48214124832922	1.71295290210118e-33	Core
C4B63_59g221	-3.62362850422523	1.98853052677308e-33	Disruptive
C4B63_17g1078c	2.12547003825655	2.11698545461139e-33	Core
C4B63_38g180	2.68419617221741	2.37575848912927e-33	Core
C4B63_25g29	2.86589447817482	2.80994612194546e-33	Disruptive
C4B63_1g201	-3.58676317653285	3.14893269724252e-33	Disruptive
C4B63_28g340	-3.15862072145512	3.51055187803454e-33	Disruptive
C4B63_68g103	-3.20489898240053	3.53812984691103e-33	Disruptive
C4B63_49g106	1.57641982777639	5.74110786751399e-33	Core
C4B63_11g45	2.86172661329387	5.95145647099129e-33	Core
C4B63_27g177	1.62884308086108	6.04652629146599e-33	Core
C4B63_1g159	-4.00008082710726	7.67726905552611e-33	Disruptive
C4B63_28g245	-2.81528367823996	7.67726905552611e-33	Core
C4B63_5g754	-3.83802638121533	7.95214135178274e-33	Disruptive
C4B63_197g20	-3.14189821168899	8.25366184996255e-33	Core
C4B63_172g41	-3.09737413841585	8.33306034175189e-33	Disruptive
C4B63_19g238	-3.25002900552298	8.56642500927379e-33	Disruptive
C4B63_212g9	4.56628488571823	1.07006463920141e-32	Core
C4B63_62g98	-3.52746340202356	1.10568334747731e-32	Disruptive
C4B63_2g702	1.45327082318019	1.3296490335262e-32	Core
C4B63_124g15	-2.1857618496638	1.51444371326232e-32	Disruptive
C4B63_32g206	2.90741176265845	1.6158838517482e-32	Core
C4B63_62g195	-3.08358588507739	2.2383760978768e-32	Disruptive
C4B63_5g424	-2.48357288127425	2.25584977526085e-32	Disruptive
C4B63_50g243	-4.48474019896715	2.36510829294313e-32	Core
C4B63_156g18	-3.17976884669117	2.52227321953998e-32	Disruptive
C4B63_31g260	-2.53290992807932	2.57524473414818e-32	Disruptive
C4B63_251g12	2.34048929105913	3.12537733195471e-32	Core
C4B63_12g109	3.07429386151271	3.78874384820004e-32	Core
C4B63_126g15	-3.73997911022657	3.78874384820004e-32	Disruptive
C4B63_81g62	2.8130281184242	3.93437679866225e-32	Core
C4B63_84g67	2.98574826787917	4.80379982485105e-32	Core
C4B63_183g11	2.04841842349156	6.00633919585685e-32	Core
C4B63_143g49	2.04286685053488	7.54017715413031e-32	Core
C4B63_19g692c	1.73445083717832	7.99687103200344e-32	Core
C4B63_77g50	-2.85975816652008	8.98391462271218e-32	Disruptive
C4B63_2g821	1.48672926357858	9.09174990242922e-32	Core
C4B63_60g163	3.2181993947955	1.05076657080003e-31	Core
C4B63_14g241	1.27087694127534	1.17130452857766e-31	Core
C4B63_81g19	2.66011270885769	1.46219179753451e-31	Core
C4B63_45g192	2.53590954699912	1.70459246387916e-31	Core
C4B63_68g70	-1.87661817045912	1.98594468666982e-31	Core
C4B63_8g2646c	1.62561969227046	2.04141412628969e-31	Core
C4B63_84g25	2.64002061332717	2.29926850223045e-31	Core
C4B63_6g592	1.37906934164581	2.50742373559299e-31	Core
C4B63_63g108	1.47249256979042	2.51682318138577e-31	Core
C4B63_5g198	-4.23873543426275	2.84848011358969e-31	Disruptive
C4B63_64g126	-3.39461535559417	2.84848011358969e-31	Disruptive
C4B63_6g273	1.66209422802332	3.23630131028285e-31	Core
C4B63_23g109	-1.73825655754322	3.97127865358538e-31	Core
C4B63_235g1	4.35949597266267	4.13690537405182e-31	Core
C4B63_52g177	1.77082377102232	5.08454699446016e-31	Core
C4B63_209g4	-2.83286445270882	7.21489186059921e-31	Disruptive
C4B63_60g180	-3.0886876159517	9.76728821534382e-31	Disruptive
C4B63_8g175	-3.02815821987617	1.33601412742501e-30	Core
C4B63_38g178	2.74481822373648	1.58315693156047e-30	Core
C4B63_343g14	-2.87933490923034	1.60918866157755e-30	Disruptive
C4B63_2g77	1.35111302203592	1.6189342799952e-30	Core
C4B63_2g1634c	1.89248923420712	1.84278458413134e-30	Core
C4B63_98g34	2.32836076378638	1.86248108897158e-30	Disruptive
C4B63_124g14	-2.43258263024135	2.36329316516074e-30	Disruptive
C4B63_84g68	2.88281933130595	2.49588095030753e-30	Core
C4B63_51g217	-3.11301508928718	2.59997477092586e-30	Disruptive
C4B63_47g131	-3.21105652475875	2.73779969564037e-30	Disruptive

C4B63_5g356	2.55037084057529	3.30860790952467e-30	Disruptive
C4B63_6g396	1.86351059329078	3.89006548941142e-30	Core
C4B63_56g144	-4.61166953027692	3.94095439091412e-30	Disruptive
C4B63_18g220	-1.6274117669331	5.24175024919137e-30	Core
C4B63_9g430	-1.64199481496105	5.38255186284759e-30	Core
C4B63_25g248	1.70078270997558	5.47617209536087e-30	Core
C4B63_60g204	-3.10871076289665	6.53783603331677e-30	Disruptive
C4B63_9g387	2.13334743535121	6.61251533210161e-30	Core
C4B63_45g174	3.95710858754694	6.7214288042209e-30	Core
C4B63_18g39	2.95980219052372	8.02571791309963e-30	Core
C4B63_8g196	2.0619024248808	8.9463835993253e-30	Core
C4B63_12g386	2.07382541175996	1.03438617908025e-29	Core
C4B63_131g40	-1.84543263790553	1.45672972054568e-29	Disruptive
C4B63_182g5	1.98437985864208	1.68994727847681e-29	Core
C4B63_35g143	-2.35812645762776	2.03120238550752e-29	Disruptive
C4B63_24g291	-4.7543537653904	2.552693647012e-29	Disruptive
C4B63_64g80	-3.72077435065287	2.74198219898622e-29	Disruptive
C4B63_4g509	1.65292701953254	2.88424955117199e-29	Core
C4B63_178g41	1.51568701290484	3.35298645371036e-29	Core
C4B63_82g70	2.27980116769172	3.49281503322223e-29	Core
C4B63_129g44	1.23252120188879	3.49281503322223e-29	Core
C4B63_65g27	-3.03126946177417	4.12878545919955e-29	Core
C4B63_37g119	-3.12136139217832	4.5590117952604e-29	Disruptive
C4B63_54g99	-3.15882853258572	6.31846079166871e-29	Disruptive
C4B63_7g72	3.15623638659847	6.75190979911053e-29	Core
C4B63_27g279	-5.46792925110149	6.79031288948076e-29	Disruptive
C4B63_24g306	1.85055615305905	6.97262419777342e-29	Core
C4B63_9g445	2.39995571686878	8.80346203151801e-29	Core
C4B63_8g172	-2.78951244693874	9.92560032811331e-29	Disruptive
C4B63_1g480	-2.99259742248082	1.05960381334138e-28	Disruptive
C4B63_59g102	-2.46427798852263	1.11359994470955e-28	Disruptive
C4B63_26g139	-2.3338666186063	1.1671615374238e-28	Core
C4B63_9g108	-3.53337312486099	1.24972259078211e-28	Disruptive
C4B63_17g284	2.12187520575007	1.61741851624748e-28	Core
C4B63_2g549	2.68640753151059	1.75439131866004e-28	Core
C4B63_35g131	-2.38106145421313	2.10013988514037e-28	Disruptive
C4B63_46g113	-3.71821920709064	2.15186868674642e-28	Disruptive
C4B63_8g163	-2.8487049968073	2.16246679061259e-28	Core
C4B63_30g231	1.74945387717208	2.63921417942975e-28	Core
C4B63_419g2	-2.69995838797399	2.77814859629038e-28	Disruptive
C4B63_1g927	-3.99419134555016	3.4552527718503e-28	Disruptive
C4B63_2g548	3.01972476533963	3.62337803292644e-28	Core
C4B63_106g63	-3.30413646277454	3.63309115344891e-28	Disruptive
C4B63_250g18	1.79592954887177	4.52760607979363e-28	Core
C4B63_31g257	-2.13912489918508	4.87049536008878e-28	Disruptive
C4B63_77g22	1.67495166388576	5.40830030518256e-28	Core
C4B63_174g27	-3.66464588717882	6.44798552330401e-28	Disruptive
C4B63_45g114	-1.30937344792413	8.38491499174797e-28	Core
C4B63_10g157	2.7889360250352	8.95000292461652e-28	Core
C4B63_1g1037	-3.29777902195446	1.04611257852545e-27	Disruptive
C4B63_11g196	-3.06862379845681	1.04611257852545e-27	Disruptive
C4B63_8g280	-3.98460507731017	1.05364455512041e-27	Disruptive
C4B63_14g20	-17.9850822879484	1.10222969284602e-27	Core
C4B63_33g309	-3.64711960001731	1.11180149171907e-27	Disruptive
C4B63_57g113	1.44276781659514	1.17767287453015e-27	Core
C4B63_32g225	1.42636644011795	1.23816979001018e-27	Core
C4B63_51g117	2.06299089242097	1.27757687883962e-27	Core
C4B63_12g227	2.54445758337942	1.3001757903442e-27	Core
C4B63_46g101	2.6084768434677	1.53367003455812e-27	Core
C4B63_58g123	2.3142295466252	1.6043073775983e-27	Core
C4B63_42g61	3.26534922690242	1.87497013428628e-27	Core
C4B63_8g285	-3.9777052385752	1.90447957588964e-27	Disruptive
C4B63_69g26	-1.93747953967295	1.94247291592387e-27	Core
C4B63_258g20	-2.75377093712637	2.27885616863272e-27	Core

C4B63_82g111	-3.04555236905703	2.42050156353437e-27	Disruptive
C4B63_36g128	-3.17838449127092	2.69899275289674e-27	Disruptive
C4B63_333g13	1.69911908429676	2.98962797707736e-27	Core
C4B63_5g740	-3.35819412704318	3.04125015207678e-27	Disruptive
C4B63_6g256	1.32483586899267	3.17254903452372e-27	Core
C4B63_91g93	-3.73508074981649	3.51963306606692e-27	Disruptive
C4B63_25g203	2.25926765759669	4.58656429720841e-27	Core
C4B63_159g4	-2.20279643554103	4.98698076542666e-27	Disruptive
C4B63_294g13	3.00800585210156	5.39246304570275e-27	Core
C4B63_22g123	-2.65116751818466	6.46225911280015e-27	Disruptive
C4B63_361g15	-2.98020024868544	6.68907786853342e-27	Disruptive
C4B63_78g52	-2.3154969581874	7.03652869260453e-27	Core
C4B63_22g78	-2.29668158617548	8.21435170208478e-27	Core
C4B63_19g13	-2.57649553786956	8.22749419022019e-27	Core
C4B63_34g216	4.23906605668497	8.86820589596438e-27	Disruptive
C4B63_375g42c	2.58448951696347	8.97674526965416e-27	Disruptive
C4B63_33g194	-1.87599554046779	1.19926498102267e-26	Disruptive
C4B63_45g184	1.71617094001185	1.28175689701776e-26	Core
C4B63_4g484	1.36595010386081	1.35053534078009e-26	Core
C4B63_277g21	2.38942561452189	1.41273674872917e-26	Core
C4B63_48g159	1.32717713985228	1.57133095168332e-26	Core
C4B63_60g110	-1.95889670366963	1.58369030102645e-26	Disruptive
C4B63_7g117	-1.34707566024205	1.80248245089658e-26	Core
C4B63_5g689	-3.70782356258168	1.80904161574738e-26	Disruptive
C4B63_28g223	4.11054082742005	1.81125427183552e-26	Core
C4B63_100g14	-3.04516669395847	2.05405907810365e-26	Disruptive
C4B63_38g143	2.11004183495288	2.12839564618117e-26	Core
C4B63_51g213	-2.62207548790056	2.20738843604853e-26	Disruptive
C4B63_11g262	1.40916556767573	2.33343331572789e-26	Core
C4B63_43g122	1.92225531245893	2.5116033959446e-26	Core
C4B63_116g59	-2.88020908185644	3.17521887897915e-26	Disruptive
C4B63_35g136	-2.08550071152026	3.67983379063608e-26	Disruptive
C4B63_6g235	2.08817759037627	3.94432194478766e-26	Core
C4B63_27g1015c	1.30628910133757	4.27541515388202e-26	Core
C4B63_1g1100	-3.56882134169722	4.33682953698041e-26	Disruptive
C4B63_21g21	1.31909284604814	4.644210635215e-26	Core
C4B63_183g15	1.50126634195014	5.20651917504839e-26	Core
C4B63_14g39	1.85104229126159	5.73173424290514e-26	Core
C4B63_57g75	1.03664132711619	5.91258597634545e-26	Core
C4B63_251g13	2.66735577769463	6.00683378092672e-26	Core
C4B63_31g233	-2.73106129150207	6.47040714878443e-26	Disruptive
C4B63_23g189	-2.31006025985431	7.73945163775332e-26	Core
C4B63_2g229	1.37924317594682	8.67895006879062e-26	Core
C4B63_8g80	-2.80648914508019	9.28022235991932e-26	Disruptive
C4B63_31g225	1.6852843331708	9.44221266469488e-26	Core
C4B63_3g853	-3.90517760784502	1.02891631508181e-25	Disruptive
C4B63_43g86	-3.07863709468807	1.17229522814945e-25	Disruptive
C4B63_328g10	1.69137505343438	1.62041862039991e-25	Core
C4B63_40g116	-1.72096384053077	1.88387597730335e-25	Core
C4B63_9g377	2.64047987703365	1.98902359237949e-25	Core
C4B63_46g105	2.41396699512057	2.03868604384219e-25	Core
C4B63_30g275	2.9042693917779	2.16116697328352e-25	Core
C4B63_157g44	-3.04485310418841	2.16116697328352e-25	Disruptive
C4B63_7g228	1.86449349209481	2.16971441760793e-25	Core
C4B63_11g213	-2.88536336552203	3.09796038631843e-25	Disruptive
C4B63_12g46	-2.01523331916346	3.12528910640544e-25	Disruptive
C4B63_6g104	1.44153363798301	3.15590192102382e-25	Core
C4B63_246g14	1.6563004822227	3.59594107258475e-25	Core
C4B63_14g2	15.4877530964473	3.82945213087733e-25	Core
C4B63_37g57	-2.27899985109715	3.9065088436088e-25	Disruptive
C4B63_50g232	-4.23237547603566	4.08943599545392e-25	Core
C4B63_150g12	-3.11163547094544	4.14201955575275e-25	Disruptive
C4B63_3g943	-3.47836434231142	4.16186649955741e-25	Disruptive
C4B63_68g121	-2.26382020490982	4.19410873888249e-25	Disruptive

C4B63_251g8	-2.94474230868265	4.41423947950915e-25	Core
C4B63_82g81	2.02965700488519	4.63517419839434e-25	Core
C4B63_419g4	-3.00266493166152	4.72469284069674e-25	Disruptive
C4B63_58g112	1.47992448628304	5.73085143386857e-25	Core
C4B63_1g666	-3.53414771475558	5.81559759815728e-25	Disruptive
C4B63_8g152	-2.62680842147068	5.82910706789908e-25	Disruptive
C4B63_26g354	2.6652664939706	6.55047562105206e-25	Core
C4B63_37g160	-3.5624300997024	7.37069965649175e-25	Disruptive
C4B63_36g364	-2.95063884738844	7.43053683647376e-25	Disruptive
C4B63_7g66	-1.79176939905875	8.53242414013177e-25	Core
C4B63_8g66	-3.69061111114232	1.06446380955061e-24	Core
C4B63_216g7	2.34993446520057	1.0655373318186e-24	Core
C4B63_68g131	-3.02294624690215	1.12880369898955e-24	Disruptive
C4B63_9g454	1.14968671461893	1.14138302540969e-24	Core
C4B63_13g176	-2.29946318255125	1.38409219779156e-24	Disruptive
C4B63_13g11	-3.75547527742251	1.53665975509112e-24	Disruptive
C4B63_5g117	-2.90434889436798	1.58629799288849e-24	Disruptive
C4B63_203g11	1.71069912719579	1.5924628449665e-24	Core
C4B63_68g107	-2.9159385101458	1.71134376050577e-24	Disruptive
C4B63_279g11	2.50189007817903	1.75426662379936e-24	Core
C4B63_1g517	-3.97090693807381	2.3602807133522e-24	Disruptive
C4B63_33g100	-2.20897052966039	3.51836646362504e-24	Disruptive
C4B63_49g227	-3.35147415172456	4.04930315814777e-24	Disruptive
C4B63_47g122	-3.07462014114663	4.29688306936749e-24	Disruptive
C4B63_50g169	-3.25256320643148	4.70548324345801e-24	Disruptive
C4B63_32g39	1.99393497196009	4.96702159199303e-24	Core
C4B63_16g8	1.52255296495141	5.3844179331741e-24	Core
C4B63_34g372	2.78971500428516	7.45224107440556e-24	Core
C4B63_27g301	-3.5292460050244	7.91198851916802e-24	Disruptive
C4B63_28g168	1.61244820548702	8.25599598903248e-24	Core
C4B63_38g39	1.9698135351627	9.73764631135407e-24	Core
C4B63_66g60	-2.57551086553771	1.07155575802741e-23	Core
C4B63_5g155	-2.9913344297843	1.18927752520551e-23	Disruptive
C4B63_3g964	-3.42109400060257	1.46537526827561e-23	Disruptive
C4B63_350g1	2.0964533966127	1.54939503300935e-23	Core
C4B63_34g146	-2.97857122871265	2.05779807986211e-23	Disruptive
C4B63_60g90	-2.14963041609643	2.17143302014946e-23	Disruptive
C4B63_3g355	-2.92952890673958	2.32780712391479e-23	Disruptive
C4B63_107g82	-3.30776733767429	2.39343315911079e-23	Disruptive
C4B63_13g10	-3.37547528486153	2.50063677666404e-23	Disruptive
C4B63_31g238	-2.23367282115422	2.64491188083705e-23	Disruptive
C4B63_112g67	-2.08679443572706	2.65907203700661e-23	Core
C4B63_296g22	-2.74509829109092	3.62042467549224e-23	Disruptive
C4B63_76g17	2.85018130070423	3.65471960706026e-23	Core
C4B63_51g85	1.12448065718751	4.47701742979165e-23	Core
C4B63_16g176	-2.31234955497921	4.83682953103076e-23	Core
C4B63_47g39	1.2555928837933	5.09398487931445e-23	Core
C4B63_54g70	1.99931687797853	5.10859213053749e-23	Core
C4B63_51g222	-3.20966003394129	5.15969400559473e-23	Disruptive
C4B63_3g65	-2.79777468295641	6.14055718989772e-23	Disruptive
C4B63_173g11	-3.1944824974556	6.28691851903482e-23	Disruptive
C4B63_155g14	-3.42605551016373	6.37507384740316e-23	Disruptive
C4B63_1g185	-3.67871199995016	6.81573860960562e-23	Disruptive
C4B63_1g106	-2.63681431680239	7.8500092515298e-23	Disruptive
C4B63_88g114	-2.60754149628895	7.86474337809131e-23	Disruptive
C4B63_212g14	1.33302573826424	8.22189651006979e-23	Core
C4B63_53g163	2.4797290988584	9.34146926134452e-23	Core
C4B63_163g33	2.44239074654152	9.50506751561652e-23	Core
C4B63_5g643	-2.57777100390362	9.58736556552383e-23	Disruptive
C4B63_216g12	-3.86055464137049	9.97217619378488e-23	Core
C4B63_44g231	-1.9939911057032	1.38134672044602e-22	Core
C4B63_1g1402	1.76271147029686	1.44482286097368e-22	Core
C4B63_111g20	-2.43497894141137	1.81243159013453e-22	Disruptive
C4B63_309g8	1.76504989763421	2.06109303941024e-22	Core

C4B63_22g75	-2.43995893660544	2.1902990348191e-22	Core
C4B63_88g112	-3.75887568990687	2.45515415814272e-22	Disruptive
C4B63_13g388	-2.19762744243591	2.65498875299704e-22	Disruptive
C4B63_2g197	2.65322786560329	2.68208300628913e-22	Core
C4B63_42g98	1.54703226020916	2.80798962349831e-22	Core
C4B63_11g230	-3.24074433150553	2.85419619121059e-22	Disruptive
C4B63_5g648	-3.15561356661369	2.88622174233899e-22	Disruptive
C4B63_253g16	1.90229692549589	3.02387964693452e-22	Core
C4B63_7g236	2.26048404361959	3.52981274796188e-22	Core
C4B63_18g287	1.42781932493349	4.01090102426961e-22	Core
C4B63_65g19	-2.46855805730534	4.03355336942873e-22	Disruptive
C4B63_8g86	-2.55881275852063	4.10440361288087e-22	Disruptive
C4B63_104g74	2.27206443878666	4.10440361288087e-22	Core
C4B63_10g538	-2.48261178736781	4.33778395422262e-22	Disruptive
C4B63_5g62	-3.73943225677795	4.6018693989923e-22	Disruptive
C4B63_137g3	1.65696337845537	5.0905292963586e-22	Core
C4B63_83g37	-2.25913504639362	5.32780513027339e-22	Disruptive
C4B63_5g239	-2.84012368383651	5.40772505212986e-22	Disruptive
C4B63_8g141	-2.46417638668763	7.26184286583642e-22	Disruptive
C4B63_44g227	2.47824013310935	7.73563494849367e-22	Core
C4B63_212g5	4.51517905210883	8.05476847073182e-22	Core
C4B63_34g211	3.95356867341949	9.27979348377594e-22	Core
C4B63_54g93	-1.87556771909232	1.00470509636318e-21	Disruptive
C4B63_60g78	1.5932510381699	1.20620837773643e-21	Core
C4B63_27g277	-3.66322463521176	1.2489446026695e-21	Disruptive
C4B63_5g100	-2.64912357182176	1.35262453828689e-21	Disruptive
C4B63_21g10	1.47155344356196	1.39717044398158e-21	Core
C4B63_11g338	-3.88462529288032	1.41134950730324e-21	Disruptive
C4B63_15g253	-2.98255929493169	1.43043547880909e-21	Disruptive
C4B63_51g208	-2.63718559793878	1.48001840877643e-21	Disruptive
C4B63_84g21	1.39610873029642	1.49524040783305e-21	Core
C4B63_53g214	-2.7198137003256	1.5126863292948e-21	Disruptive
C4B63_1g1004	-3.25679459023115	1.67316102681357e-21	Disruptive
C4B63_15g419	-3.29160389255267	1.74373485043819e-21	Disruptive
C4B63_51g126	1.68134304853677	1.80900825156125e-21	Core
C4B63_54g100	-1.96309055925189	1.89697365066072e-21	Disruptive
C4B63_82g75	1.3093692178486	1.90213298839505e-21	Core
C4B63_30g133	1.79861590900306	2.04479350664508e-21	Core
C4B63_2g711	1.65773159932695	2.11141047004035e-21	Core
C4B63_3g760	-3.05196339993447	2.48677452391739e-21	Disruptive
C4B63_89g178c	1.3901335306884	2.52887120171485e-21	Core
C4B63_93g6	-3.33612056451316	2.79646606740462e-21	Disruptive
C4B63_58g38	1.86308650201866	2.80049137403837e-21	Core
C4B63_5g506	-3.42546126754402	2.8190299640043e-21	Disruptive
C4B63_47g44	1.15958022232185	2.97939813067302e-21	Core
C4B63_132g35	-3.27316402159515	3.63680316599608e-21	Disruptive
C4B63_121g78	-3.87497807411458	3.93527559529226e-21	Disruptive
C4B63_20g114	1.60288172094419	4.13889243712273e-21	Core
C4B63_20g219	2.14204094310469	4.13889243712273e-21	Core
C4B63_49g168	3.06485356416803	4.15689535138663e-21	Core
C4B63_37g382	2.38093283180591	4.24390796935367e-21	Disruptive
C4B63_16g302	1.92871882669554	4.30257083214186e-21	Core
C4B63_24g253	-2.20607516573928	4.6725071437162e-21	Disruptive
C4B63_11g198	-2.78228630686871	4.83252841544209e-21	Disruptive
C4B63_106g19	-1.97179837790273	5.29802704056849e-21	Disruptive
C4B63_30g118	1.50348305876823	5.70671932750923e-21	Core
C4B63_14g106	1.73386853543918	6.13621747442075e-21	Core
C4B63_27g230	1.32104174325167	6.13621747442075e-21	Core
C4B63_23g217	1.01192832544554	6.84623201332702e-21	Core
C4B63_27g322	-4.08390903346761	7.15623503038307e-21	Disruptive
C4B63_181g25	2.03382334323909	7.56189494829919e-21	Core
C4B63_4g485	1.35764302781212	7.57538948375838e-21	Core
C4B63_22g96	-1.38699505505182	8.01992109287372e-21	Core
C4B63_4g110	-1.43213398179376	8.20117301998796e-21	Core

C4B63_296g27	-3.06553773080517	8.51953425822378e-21	Disruptive
C4B63_11g371	-3.23383503299307	9.03387614317217e-21	Disruptive
C4B63_225g35	1.31635390423971	9.26525613782232e-21	Core
C4B63_251g19	3.44743438009653	1.01636619922298e-20	Core
C4B63_46g96	2.6040234636868	1.06476277481938e-20	Core
C4B63_18g151	-1.56624209601095	1.09810577326753e-20	Core
C4B63_3g848	-2.41514178690915	1.10102468361386e-20	Disruptive
C4B63_12g268	-2.46580598117152	1.36520138189502e-20	Disruptive
C4B63_8g486	1.84781124702658	1.41551198773321e-20	Core
C4B63_25g330	-1.86928324296115	1.41551198773321e-20	Core
C4B63_79g24	1.46136929723494	1.45460774321253e-20	Core
C4B63_35g191	1.38548638112994	1.48318011985585e-20	Core
C4B63_27g332	-3.17648360184675	1.4961280578898e-20	Disruptive
C4B63_112g36	1.56011690016262	1.51367071977165e-20	Core
C4B63_214g11	-1.50053981760411	1.61793869505838e-20	Core
C4B63_568g1	-2.71221874241729	1.67013863511157e-20	Disruptive
C4B63_60g104	-2.49787479362665	1.88298464719345e-20	Disruptive
C4B63_46g190	2.15687632085275	1.94540412784714e-20	Core
C4B63_35g3	-3.00643087629782	2.1020291546243e-20	Core
C4B63_30g222	1.97100960674584	2.17107599292297e-20	Core
C4B63_127g37	1.80889825446799	2.23982874990167e-20	Core
C4B63_52g64	2.52914751655948	2.38629071267633e-20	Core
C4B63_282g14	1.68572149861816	2.55479036158889e-20	Core
C4B63_6g149	2.00695193329325	2.69873312135013e-20	Core
C4B63_17g268	1.96967879544896	2.78493116644451e-20	Core
C4B63_124g18	-2.77969072657235	2.95827935745334e-20	Disruptive
C4B63_41g154	-2.39742577330945	3.08925920809471e-20	Core
C4B63_54g71	2.02488875418044	3.29697759394345e-20	Core
C4B63_4g172	1.87256819692242	3.6286391125838e-20	Core
C4B63_1g629	-3.92715654789165	3.71519325301431e-20	Disruptive
C4B63_31g255	-2.15056245995401	4.13955651474774e-20	Disruptive
C4B63_139g64	-2.74131104495346	4.16145881356477e-20	Disruptive
C4B63_168g33	1.03634847360207	4.23846830812483e-20	Core
C4B63_111g13	-2.7392118967649	4.2721306044058e-20	Disruptive
C4B63_22g764c	1.58385389099738	4.46770063669924e-20	Core
C4B63_2g76	1.3078039019163	4.53032118933619e-20	Core
C4B63_44g208	-1.69772500253711	4.63537828018027e-20	Core
C4B63_29g63	-2.98766278337801	4.84919177581134e-20	Disruptive
C4B63_25g253	1.57806579292562	5.04620254465467e-20	Core
C4B63_124g47	-3.75289620422482	5.11260357090275e-20	Disruptive
C4B63_26g310	1.6875332289143	5.12122139567632e-20	Core
C4B63_64g140	-2.88011143807117	5.20529300087622e-20	Disruptive
C4B63_1g983	-3.27179263300574	5.29511390546366e-20	Disruptive
C4B63_26g309	1.69803105458967	5.75330540837859e-20	Core
C4B63_1g701	-2.70128416305535	5.81821877157395e-20	Disruptive
C4B63_9g109	-3.49842711676873	5.98502962245412e-20	Disruptive
C4B63_169g32	-3.01493754845956	6.12412453029676e-20	Disruptive
C4B63_75g56	1.77286145394102	6.37583799674948e-20	Core
C4B63_31g230	-2.1929808223924	7.24237169581107e-20	Disruptive
C4B63_237g2	2.21248225991007	7.68384631955711e-20	Core
C4B63_1g25	1.79552993083426	8.02814826847035e-20	Core
C4B63_66g70	-2.26595396766419	8.18373306955441e-20	Disruptive
C4B63_44g218	1.72535637684653	8.2836655089593e-20	Core
C4B63_9g479	-3.58519981114715	9.53711474644172e-20	Disruptive
C4B63_78g59	-2.37584565337407	1.05102892262804e-19	Disruptive
C4B63_18g288	1.3378720545902	1.06305895568552e-19	Core
C4B63_1g872	-3.20421829422889	1.15023709810387e-19	Disruptive
C4B63_44g191	1.63773848645985	1.25817651851128e-19	Core
C4B63_11g146	-2.28482363028166	1.35370878275607e-19	Disruptive
C4B63_1g665	-2.43296847016458	1.36108611910104e-19	Disruptive
C4B63_8g318	-2.65365825615165	1.47990850158732e-19	Core
C4B63_53g223	-3.70480455434406	1.47990850158732e-19	Disruptive
C4B63_82g85	1.65478426361447	1.47990850158732e-19	Core
C4B63_47g90	-2.92576402509155	1.52475758922206e-19	Disruptive

C4B63_51g104	1.9657832174425	1.87565838579819e-19	Core
C4B63_1g806	-3.42285437724523	1.9659858170739e-19	Disruptive
C4B63_5g91	-2.64218219958275	1.97056609717222e-19	Disruptive
C4B63_6g117	-1.70194300114157	1.97056609717222e-19	Core
C4B63_200g44	-2.14107573928826	2.05871029298357e-19	Core
C4B63_88g26	-2.77929236709342	2.08465018039425e-19	Disruptive
C4B63_29g77	-2.94082463205515	2.49008869360458e-19	Disruptive
C4B63_111g12	-1.9167746362109	2.59392160725176e-19	Disruptive
C4B63_6g263	1.73012457096571	2.71904108693957e-19	Core
C4B63_95g19	-1.39067881100504	2.72640343242462e-19	Core
C4B63_72g79	1.26773178258734	2.78223265651412e-19	Core
C4B63_162g31	-2.81083796930265	2.87056675391799e-19	Core
C4B63_37g420	2.08920284552716	2.94931859777104e-19	Core
C4B63_1g537	-3.0843950224728	3.21504461618539e-19	Disruptive
C4B63_459g7	3.0479107629868	3.27063460740414e-19	Core
C4B63_32g138	-3.28719263382331	3.45355417371059e-19	Disruptive
C4B63_259g12	2.26985413119343	3.47473360379814e-19	Core
C4B63_21g5	1.19184580399582	3.51766713224307e-19	Core
C4B63_153g8	1.3152772426678	3.67158643207759e-19	Core
C4B63_3g681	-3.74257979394347	3.78298268668213e-19	Disruptive
C4B63_15g19	2.33871721041222	3.8853283627673e-19	Core
C4B63_28g33	1.29678836692161	4.0124380720487e-19	Core
C4B63_9g96	-3.13217942717663	4.4682667831026e-19	Disruptive
C4B63_7g428	-1.78681509832807	4.54553740991199e-19	Core
C4B63_59g106	-2.66717331785375	4.76213697324855e-19	Disruptive
C4B63_4g333	-1.6353980483297	4.84683203466805e-19	Core
C4B63_69g25	1.22928942709363	4.92697976720557e-19	Core
C4B63_37g100	-1.97503590348089	5.4877936351043e-19	Disruptive
C4B63_295g27	1.20916537416266	5.78634066142093e-19	Core
C4B63_294g12	2.66616858785553	5.92209488862414e-19	Core
C4B63_21g273	1.26719806318329	5.950360944824e-19	Core
C4B63_1g239	-1.90449556480168	6.16395695793623e-19	Disruptive
C4B63_57g38	-2.21863829037098	6.49212416235845e-19	Disruptive
C4B63_27g266	-2.69822442247054	6.84720419980241e-19	Disruptive
C4B63_97g31	1.26542209356176	6.84720419980241e-19	Core
C4B63_4g468	2.30259996153885	7.43536417050144e-19	Core
C4B63_59g110	-3.32682775136841	7.61741071227667e-19	Disruptive
C4B63_9g477	-2.56152404109083	7.72666144237004e-19	Disruptive
C4B63_141g8	-2.08752437844023	8.19963379314157e-19	Disruptive
C4B63_196g41	-2.3389992285293	8.48101805785602e-19	Disruptive
C4B63_43g204	2.06182699000919	8.88219493343051e-19	Core
C4B63_159g21	-3.04397234633444	9.28381179202175e-19	Disruptive
C4B63_15g484	-2.79960234043266	9.8662304798473e-19	Disruptive
C4B63_26g240	1.64743556971777	1.09725461982349e-18	Core
C4B63_2g208c	1.47542130574716	1.13639697343273e-18	Core
C4B63_37g66	-3.02857728973658	1.18832381490972e-18	Disruptive
C4B63_56g153	-2.50344288034057	1.25117596576092e-18	Disruptive
C4B63_9g376	2.75969988412389	1.3451299384908e-18	Core
C4B63_8g316	-2.50411735990889	1.39116951210833e-18	Disruptive
C4B63_277g7	1.92252139309801	1.39116951210833e-18	Core
C4B63_8g282	-2.89445486217815	1.47754284299176e-18	Disruptive
C4B63_3g499	-3.62698192829508	1.59319029053211e-18	Disruptive
C4B63_37g77	-2.79836734929789	1.75012231431536e-18	Disruptive
C4B63_107g30	-2.63193455119723	1.87030381088534e-18	Disruptive
C4B63_8g179	-2.43750521871267	1.92578638692076e-18	Disruptive
C4B63_65g61	-3.59203348344034	2.00250754743267e-18	Disruptive
C4B63_44g146	1.51523664107402	2.01596966068924e-18	Core
C4B63_15g79	-3.45965406321241	2.0586382241142e-18	Disruptive
C4B63_37g151	-3.49628510302059	2.0723989879805e-18	Disruptive
C4B63_1g1319	-2.97940582347747	2.31819462595329e-18	Disruptive
C4B63_23g113	1.14347137122274	2.40238916725598e-18	Core
C4B63_18g23	-1.33570734931665	2.54104216264869e-18	Core
C4B63_4g63	-1.95433845030345	2.55191237597301e-18	Core
C4B63_5g486	-4.04608870617126	2.58121574161556e-18	Disruptive

C4B63_247g14	1.63699111062196	2.6340436393754e-18	Core
C4B63_126g13	-2.63798874709278	2.69320221216898e-18	Disruptive
C4B63_48g143	1.0659947743933	2.81436102148354e-18	Core
C4B63_28g160	1.08506284053504	2.91567862502752e-18	Core
C4B63_73g74	1.57165153607532	2.91567862502752e-18	Core
C4B63_27g292	-3.61871436024922	2.92858337587265e-18	Disruptive
C4B63_31g133	-2.85489558688976	2.92858337587265e-18	Disruptive
C4B63_1g1233	-3.14769550915501	2.9517896944077e-18	Disruptive
C4B63_25g221	1.34017619180608	3.12730252285536e-18	Core
C4B63_132g24	-2.43061403126626	3.31066401004604e-18	Disruptive
C4B63_60g113	-2.69100632780774	3.3926614009144e-18	Disruptive
C4B63_34g364	-1.45426299708937	3.5272739912502e-18	Core
C4B63_126g31	-2.96033126625087	3.59776420580105e-18	Disruptive
C4B63_62g100	-1.86712244793514	3.70262649052442e-18	Disruptive
C4B63_37g188	-3.80905154851954	3.856836396705e-18	Disruptive
C4B63_15g389	-2.69069072071385	4.2568295153335e-18	Disruptive
C4B63_47g91	-2.93225572171278	4.45339197984145e-18	Disruptive
C4B63_31g263	-2.49606294819526	4.47898855130927e-18	Disruptive
C4B63_1g1302	-2.72165894655989	4.4992345919758e-18	Disruptive
C4B63_155g9	-2.23760961138823	4.5783856053431e-18	Disruptive
C4B63_71g5	-4.99448263163296	4.65774344870971e-18	Disruptive
C4B63_1g200	-2.86315718129943	4.73991682930786e-18	Disruptive
C4B63_126g22	-3.0344429783305	4.89505247223222e-18	Disruptive
C4B63_63g86	1.37649322390324	4.93309746719783e-18	Core
C4B63_12g197	1.76748544949743	4.97272670599961e-18	Core
C4B63_427g3	-3.52510470187688	5.47279426196354e-18	Core
C4B63_134g44	-1.93848942260623	5.50161803540017e-18	Disruptive
C4B63_8g133	-2.76734930793375	5.72710319590082e-18	Disruptive
C4B63_63g31	1.03642086908209	5.77080838978963e-18	Core
C4B63_107g46	-3.44685055107016	6.07664055756907e-18	Disruptive
C4B63_2g284	-2.99669289659168	6.09141128797629e-18	Disruptive
C4B63_1g1128	-3.04654810308244	6.25458878185911e-18	Disruptive
C4B63_70g572c	2.07965935214109	6.39744161253578e-18	Core
C4B63_20g162	2.18706441780237	7.38142029351359e-18	Core
C4B63_15g415	-2.73006180423648	7.39087339185941e-18	Disruptive
C4B63_11g484	1.03538697891269	7.43022749875971e-18	Core
C4B63_42g242	1.18699240434192	8.24384164710913e-18	Core
C4B63_38g189	-1.49559029529973	8.72871721384e-18	Disruptive
C4B63_46g10	-2.25658563419909	9.52593937635225e-18	Core
C4B63_64g186	-2.87540900346622	9.68308224952514e-18	Disruptive
C4B63_480g13	-2.27943247032363	9.6918082857088e-18	Core
C4B63_1g780	-2.72475990288871	1.00450767833809e-17	Disruptive
C4B63_3g566	-2.55481663366639	1.00880796161699e-17	Disruptive
C4B63_37g159	-3.86759457702316	1.01513648071343e-17	Disruptive
C4B63_20g10	12.9641308947868	1.04074779458549e-17	Core
C4B63_67g98	-2.67721683712012	1.26841604278142e-17	Disruptive
C4B63_35g347	-3.4003223239663	1.3109982321715e-17	Disruptive
C4B63_2g660	-4.21011268529205	1.33035958103399e-17	Disruptive
C4B63_70g94	2.12981321122056	1.39394864965347e-17	Core
C4B63_25g206	1.92960768351116	1.45997857617853e-17	Core
C4B63_19g112	1.27687970599571	1.47241082513231e-17	Core
C4B63_22g153	-2.33681500788765	1.51200174896379e-17	Disruptive
C4B63_106g49	-2.55668887778558	1.51200174896379e-17	Disruptive
C4B63_132g12	-2.75293609690197	1.51200174896379e-17	Disruptive
C4B63_5g142	-3.12086479604559	1.80868794485477e-17	Disruptive
C4B63_93g25	2.00385389990449	1.80886533553425e-17	Disruptive
C4B63_54g101	-2.14187057814928	1.85401317561014e-17	Disruptive
C4B63_9g474	-2.44181011551627	1.96242040957506e-17	Disruptive
C4B63_14g119	-2.41660602039633	1.96646300800347e-17	Disruptive
C4B63_64g50	-2.33224447418339	1.99152811635526e-17	Disruptive
C4B63_39g336	1.24814300913716	2.23173588424881e-17	Core
C4B63_124g48	-3.41760916641917	2.23173588424881e-17	Disruptive
C4B63_417g23	-4.72052298664238	2.28211289365315e-17	Disruptive
C4B63_169g39	-2.44202096277351	2.38922593172324e-17	Disruptive

C4B63_32g236	1.28222096853519	2.41750071775615e-17	Core
C4B63_33g68	-3.04159712746111	2.41754657106171e-17	Disruptive
C4B63_32g309	-2.18014101565243	2.51718279771439e-17	Disruptive
C4B63_59g129	2.01330926414191	2.51718279771439e-17	Core
C4B63_116g18	1.24529840412263	2.7192221411666e-17	Core
C4B63_18g279	1.30741634433338	2.75765177789204e-17	Core
C4B63_33g163	1.89038087590603	2.83349892745618e-17	Disruptive
C4B63_22g780c	-2.7878948034256	2.99238728660073e-17	Core
C4B63_56g142	-3.25174026902511	3.06487320737107e-17	Disruptive
C4B63_121g75	-2.83738430153854	3.34160635374654e-17	Disruptive
C4B63_46g189	1.5558748291147	3.40603355246762e-17	Core
C4B63_3g237	-3.45443297018174	3.41836852335143e-17	Disruptive
C4B63_3g627	-2.75732121226782	3.91844584018744e-17	Disruptive
C4B63_5g641	-2.91757539039538	4.02844945667537e-17	Disruptive
C4B63_56g34	1.30589455634031	4.09260504605891e-17	Core
C4B63_38g310	-2.33701729818426	4.14714567554958e-17	Disruptive
C4B63_27g265	-3.09335505709633	4.42214834065965e-17	Disruptive
C4B63_34g1240c	4.13857239877494	5.15450166269535e-17	Disruptive
C4B63_41g153	-2.09839404492041	5.49668202206722e-17	Core
C4B63_54g91	-2.2171527310229	5.74923769686415e-17	Disruptive
C4B63_5g398	-2.42770916851892	5.89745270081162e-17	Disruptive
C4B63_51g205	3.32849587225029	6.00924317250627e-17	Core
C4B63_1g1025	-3.27179057791948	6.38777066757763e-17	Disruptive
C4B63_319g13	-2.59082152726064	6.56657339779082e-17	Disruptive
C4B63_90g102	-3.21424416906301	6.68583589445479e-17	Core
C4B63_138g37	1.97730608684165	6.8135259949681e-17	Core
C4B63_8g185	-2.87580498645592	7.52862753859682e-17	Disruptive
C4B63_8g112	-2.19303082898861	8.57606523464616e-17	Disruptive
C4B63_97g14	1.9043765924936	8.61273838252018e-17	Core
C4B63_303g10	1.31605521155932	9.70561308152107e-17	Core
C4B63_10g528	1.24112883608772	1.11630752712086e-16	Core
C4B63_2g829	1.60133872631201	1.2740131074291e-16	Core
C4B63_93g9	-3.68625039069962	1.30344692713802e-16	Disruptive
C4B63_19g183	1.62941562968002	1.36210651338535e-16	Core
C4B63_1g1282	-2.8775836172236	1.43521164514683e-16	Disruptive
C4B63_52g78	-1.4523445843528	1.46183862825835e-16	Core
C4B63_117g59	-2.37576053036696	1.53412890499526e-16	Disruptive
C4B63_43g127	1.06287838064023	1.60296455331827e-16	Core
C4B63_30g131	1.82233052209198	1.6281228998677e-16	Core
C4B63_60g109	-2.33602710812004	1.6740755415893e-16	Disruptive
C4B63_18g152	-1.79461604383166	1.74002989265755e-16	Core
C4B63_62g149	-2.59039579435455	1.75293584077132e-16	Disruptive
C4B63_28g342	-1.9998298764207	1.88866692935555e-16	Disruptive
C4B63_3g791	-2.3479262741974	1.93873367321217e-16	Disruptive
C4B63_7g361	1.58726837362831	2.01275672352729e-16	Core
C4B63_37g368	1.36412239862067	2.09384764023014e-16	Core
C4B63_74g106	-5.15970737311391	2.09384764023014e-16	Disruptive
C4B63_11g382	-3.83272644647545	2.10834692735568e-16	Disruptive
C4B63_48g44	1.29390885516664	2.15852839283548e-16	Core
C4B63_111g11	-1.96173727952539	2.36622263147766e-16	Disruptive
C4B63_39g182	-2.73639183776199	2.39192303844019e-16	Disruptive
C4B63_5g757	-2.27815780849406	2.47100177421199e-16	Disruptive
C4B63_209g8	-1.93476646426274	2.5154318836846e-16	Disruptive
C4B63_63g33	1.50568333360617	2.52970530417343e-16	Core
C4B63_66g157	-3.1516979252778	2.55781121161264e-16	Disruptive
C4B63_9g472	-1.29409767193711	2.68335642649845e-16	Core
C4B63_60g89	-2.1159243057811	2.88477682776941e-16	Disruptive
C4B63_197g29	-2.27482900239956	2.93721207854713e-16	Disruptive
C4B63_42g208	-3.34471603347769	3.01144610397355e-16	Core
C4B63_59g213	-3.09531360551657	3.09068615414828e-16	Disruptive
C4B63_170g16	1.72753731910088	3.11541924165657e-16	Core
C4B63_10g127	1.90807525100226	3.20792608478853e-16	Core
C4B63_1g86	-1.59982242698028	3.43172407789188e-16	Disruptive
C4B63_29g450	-2.42989479143963	3.55287357519764e-16	Disruptive

C4B63_7g356	1.30235765783754	3.79396354101481e-16	Core
C4B63_6g255	1.38747453473839	3.95011151659057e-16	Core
C4B63_3g724	-2.97146462974468	4.19807869302267e-16	Disruptive
C4B63_65g13	-3.25892338874726	4.40614561407058e-16	Core
C4B63_136g24	-1.88866050497218	4.66430729313726e-16	Disruptive
C4B63_66g110	-2.82067033170025	4.68147541766039e-16	Disruptive
C4B63_280g18	1.71370121446231	4.71692012322064e-16	Core
C4B63_25g137	1.21178348118199	5.05526850049852e-16	Core
C4B63_14g128	-2.39664129306068	5.19909406306725e-16	Disruptive
C4B63_4g438	-1.09348703078741	5.33379172032446e-16	Core
C4B63_5g752	-3.63828424068338	5.45654777971652e-16	Disruptive
C4B63_47g125	-2.21129313785119	5.8486879738972e-16	Disruptive
C4B63_25g290	1.59729088368794	5.94947349927678e-16	Core
C4B63_3g774	-2.55317837403042	6.49266234619709e-16	Disruptive
C4B63_46g95	2.69264094757704	6.60912460902351e-16	Core
C4B63_37g383	1.72621165515251	6.77418285681666e-16	Disruptive
C4B63_62g138	-4.63788305419815	6.85788134054007e-16	Disruptive
C4B63_3g864	-2.38284167222362	7.13389456484188e-16	Disruptive
C4B63_100g73	-4.73920320968092	8.19242838392118e-16	Disruptive
C4B63_75g82	-2.07447077418585	8.46492872459318e-16	Disruptive
C4B63_68g77	-1.77537720666323	8.66152423307731e-16	Disruptive
C4B63_4g508	-1.5093523386918	8.76443358874682e-16	Core
C4B63_1g82	-1.98471965420768	8.80764585996051e-16	Disruptive
C4B63_9g395	-1.12714262582938	8.84411317171396e-16	Core
C4B63_74g55	-2.08177547706988	8.96816276756472e-16	Disruptive
C4B63_113g47	1.17391169595613	9.87315347151233e-16	Core
C4B63_36g310	-3.14232475732715	1.03448667905854e-15	Disruptive
C4B63_37g172	-2.24566660804292	1.06152554256981e-15	Disruptive
C4B63_3g1002	-2.80056339382932	1.06416990866487e-15	Disruptive
C4B63_10g527	1.29822574671885	1.07086871615248e-15	Core
C4B63_152g35	-1.93831975561714	1.07263961637389e-15	Disruptive
C4B63_93g11	-3.32850962561072	1.09678138768967e-15	Disruptive
C4B63_84g35	1.02673539716531	1.14408998165859e-15	Core
C4B63_9g373	1.54445017129918	1.1671866101169e-15	Core
C4B63_16g17	1.28541827907711	1.28103167761436e-15	Core
C4B63_23g16	2.02797259386682	1.4135632299389e-15	Core
C4B63_39g377	-3.29049085320201	1.45381327961188e-15	Disruptive
C4B63_196g33	-2.78318665585766	1.50900852008346e-15	Disruptive
C4B63_47g111	1.78961771282005	1.58441661843892e-15	Core
C4B63_32g326	1.58027010419712	1.63331486152893e-15	Core
C4B63_5g125	-3.20013676919735	1.83164345968558e-15	Disruptive
C4B63_219g48	1.37793921702705	1.92032520605784e-15	Core
C4B63_218g26	1.61126439412126	1.97819838201787e-15	Core
C4B63_44g106	1.28991377937791	2.00993950799359e-15	Core
C4B63_255g15	2.01786197695671	2.16863793413184e-15	Core
C4B63_3g844	-3.00801352446726	2.22449928753677e-15	Disruptive
C4B63_396g11	1.99082959449412	2.39136592116076e-15	Core
C4B63_15g379	-2.99177788536255	2.43731746846283e-15	Disruptive
C4B63_11g426	-2.54631132458414	2.54304888922383e-15	Disruptive
C4B63_40g171	-2.34276360595919	2.54304888922383e-15	Disruptive
C4B63_133g28	2.13469250726944	2.74424303985355e-15	Core
C4B63_613g1	-2.41823418898733	2.77022937216683e-15	Disruptive
C4B63_334g25	-2.78377741513005	2.87105030504381e-15	Disruptive
C4B63_72g33	-2.78162727965695	2.97822809407094e-15	Core
C4B63_23g78	1.19638438662602	3.04509313016981e-15	Core
C4B63_25g240	1.6423828995502	3.04878744909625e-15	Core
C4B63_52g63	2.3618999714549	3.06710194235183e-15	Core
C4B63_35g178	-1.65196293444456	3.07776134377093e-15	Core
C4B63_9g100	-2.7247797151151	3.30287441742874e-15	Disruptive
C4B63_39g135	-3.35622702676009	3.31989822764458e-15	Disruptive
C4B63_45g238	1.6810012367083	3.4156252823204e-15	Core
C4B63_87g12	1.19721326780581	3.72855288053596e-15	Core
C4B63_19g185	1.79776533077662	3.75108817081574e-15	Core
C4B63_69g41	-1.93255283241956	3.81309084744273e-15	Disruptive

C4B63_2g205	2.38565900479833	3.88410545406714e-15	Core
C4B63_258g27	12.3191791280511	3.98177479988068e-15	Core
C4B63_91g63	-2.8512113200758	4.17792059521346e-15	Disruptive
C4B63_197g26	-2.21128716866354	4.36997639413335e-15	Disruptive
C4B63_26g50	-2.41710327191013	4.76354986094028e-15	Disruptive
C4B63_54g103	-2.45811992122685	4.79871736708093e-15	Disruptive
C4B63_55g87	1.19700190678928	4.79871736708093e-15	Core
C4B63_3g496	-3.06149091869495	5.33944352707098e-15	Disruptive
C4B63_3g621	-2.50989025583898	5.37703692839683e-15	Disruptive
C4B63_75g91	-1.79301012236282	5.49172016421124e-15	Disruptive
C4B63_116g57	-3.13529590450422	5.59105796365606e-15	Disruptive
C4B63_262g21	1.3907722504404	5.68500937387572e-15	Core
C4B63_1g1045	-2.91630296701661	5.91308817014513e-15	Disruptive
C4B63_111g5	-2.09792868848696	6.36011934221352e-15	Disruptive
C4B63_2g708	1.17348236305468	6.37113743260661e-15	Core
C4B63_150g11	-2.02286624772533	6.56295668489804e-15	Disruptive
C4B63_197g31	-1.89120088717367	6.56448529543076e-15	Disruptive
C4B63_98g33	-2.62810774318079	6.68673523487979e-15	Disruptive
C4B63_8g129	-3.39119781869411	6.70986799951219e-15	Disruptive
C4B63_1g112	-2.36959834302815	6.78837036699285e-15	Disruptive
C4B63_12g225	1.18151969078735	6.9112504022884e-15	Core
C4B63_46g78	-1.76480030034608	6.94880407216752e-15	Core
C4B63_38g169	1.54854248248648	7.34119421995856e-15	Core
C4B63_37g118	-2.88698469006284	8.32630907819373e-15	Disruptive
C4B63_19g723c	1.46941891444648	8.63866797779026e-15	Core
C4B63_29g48	-3.53144111834473	9.02500127141605e-15	Disruptive
C4B63_32g257	-1.22489485124813	9.25112232943862e-15	Core
C4B63_27g306	-2.50519855952187	1.01373377069644e-14	Disruptive
C4B63_2g30	1.33298859120017	1.01979135362381e-14	Core
C4B63_5g77	-2.14827709388216	1.02714149462321e-14	Disruptive
C4B63_25g199	-2.82010313513513	1.08462547864702e-14	Disruptive
C4B63_1g1058	-2.74070133656873	1.090063262477e-14	Disruptive
C4B63_8g186	-2.06697528521971	1.0938956268043e-14	Disruptive
C4B63_138g18	-1.60041659634253	1.11803742999644e-14	Disruptive
C4B63_33g40	-3.05201071188091	1.13341455948425e-14	Disruptive
C4B63_114g19	1.63755521166338	1.13818688202834e-14	Core
C4B63_1g1397	-2.03335918000597	1.17326096009813e-14	Disruptive
C4B63_28g68	1.19984247892014	1.22006938400962e-14	Core
C4B63_167g40	-2.33640980213302	1.25736439416861e-14	Disruptive
C4B63_138g32	-1.36699108419098	1.32363641560151e-14	Core
C4B63_23g1250c	1.50326914002754	1.33726732038842e-14	Core
C4B63_3g847	-2.97832228606329	1.38004766843778e-14	Disruptive
C4B63_27g3	2.13003655616302	1.43919892612902e-14	Core
C4B63_15g12	1.73790515890574	1.47482072684418e-14	Core
C4B63_120g31	-1.44180088712722	1.5613855206386e-14	Disruptive
C4B63_1g1051	-2.45649231306625	1.57843974167663e-14	Disruptive
C4B63_12g385	1.13475293588493	1.59040609265401e-14	Core
C4B63_47g34	2.31710886814363	1.60203791392559e-14	Core
C4B63_68g136	1.45533446991506	1.70609156670866e-14	Core
C4B63_241g16	1.57624617959793	1.7085108381546e-14	Core
C4B63_65g113	-3.55223189329164	1.72749880737013e-14	Disruptive
C4B63_1g1265	-2.83447059648696	1.79739184716697e-14	Disruptive
C4B63_29g356	-2.95869596104016	1.83942197379176e-14	Disruptive
C4B63_107g92	-3.2681744727263	1.91185344295216e-14	Disruptive
C4B63_136g25	-1.95447060677399	1.92926943400759e-14	Disruptive
C4B63_55g287c	1.92953236550867	2.03541921920898e-14	Core
C4B63_22g162	2.01616245777573	2.1202038553001e-14	Core
C4B63_27g321	-2.85446861514063	2.33640416257496e-14	Disruptive
C4B63_23g98	-1.80608791194885	2.33738121901038e-14	Core
C4B63_54g18	1.47024723061995	2.3567657918706e-14	Core
C4B63_336g6	2.37027044603472	2.42719481598693e-14	Core
C4B63_2g3	-1.42855687195895	2.52638834015e-14	Core
C4B63_39g233	-2.39857946420139	2.72353374712939e-14	Disruptive
C4B63_13g325	1.45869861634224	2.85296960600054e-14	Core

C4B63_51g210	-1.98755314586253	2.93726191564457e-14	Disruptive
C4B63_50g193	-2.59428031143188	2.98477448129086e-14	Disruptive
C4B63_10g112	1.48877160097299	3.04726479376968e-14	Core
C4B63_23g130	-1.17931455380276	3.09095044113612e-14	Core
C4B63_94g80	-2.73489991763697	3.12126335216111e-14	Disruptive
C4B63_7g357	1.54344842748153	3.32832905736655e-14	Core
C4B63_66g145	-3.25589658831717	3.3993751791225e-14	Disruptive
C4B63_275g6	-1.21852066886156	3.42267516884114e-14	Core
C4B63_38g94	2.00761197630112	3.73714636620118e-14	Core
C4B63_36g183	-1.87717857588664	3.77480609611391e-14	Disruptive
C4B63_257g7	1.68904343321251	3.85355154956576e-14	Core
C4B63_11g177	-3.043234265266	3.85704395822951e-14	Disruptive
C4B63_350g4	2.75057559609	4.06895184794102e-14	Core
C4B63_219g47	1.35524906418723	4.07792021709159e-14	Core
C4B63_8g121	-2.59486727350579	4.09611554797608e-14	Disruptive
C4B63_225g26	1.23459941955878	4.10896731621188e-14	Core
C4B63_22g83	1.7362164462953	4.52081022273801e-14	Core
C4B63_49g109	1.55549338582034	4.69735735164238e-14	Core
C4B63_343g13	-2.73515252060586	4.9307764920969e-14	Disruptive
C4B63_51g81	1.23675029725282	4.99273288683484e-14	Core
C4B63_63g50	1.33824736772489	5.5589781880181e-14	Core
C4B63_65g60	-3.5171246949997	5.67549014438455e-14	Disruptive
C4B63_155g21	-2.77601854309977	5.78239113135976e-14	Disruptive
C4B63_1g1095	-2.77511272248329	5.79091657010638e-14	Disruptive
C4B63_15g224	-2.337457186578	6.2736521480137e-14	Disruptive
C4B63_2g744	-1.09593399274077	6.74359187746482e-14	Core
C4B63_29g13	-3.43132784442763	6.81345478257443e-14	Disruptive
C4B63_12g405	1.52414551535848	6.93392045699184e-14	Core
C4B63_51g225	-2.55324497631675	7.00990426424539e-14	Disruptive
C4B63_23g1438c	1.89265278173567	7.35688510284215e-14	Core
C4B63_43g134	1.6210180429554	7.58158998215068e-14	Core
C4B63_295g26	1.11456944092439	7.59897061697097e-14	Core
C4B63_14g140	-2.16682489735441	8.27398195943459e-14	Disruptive
C4B63_82g94	-1.54271746442798	8.40059756386268e-14	Core
C4B63_64g142	-1.78019835436058	8.89451586433653e-14	Disruptive
C4B63_115g9	-2.94228530395832	8.89451586433653e-14	Disruptive
C4B63_38g62	-1.3394453495787	8.99349272959261e-14	Core
C4B63_59g131	1.08315396659572	9.03497548161476e-14	Core
C4B63_1g1261	-2.66995758575901	9.08695494952024e-14	Core
C4B63_59g114	1.05259113344205	9.12113707185258e-14	Core
C4B63_12g266	-2.23344069336562	9.38119708099853e-14	Disruptive
C4B63_15g269	-2.31558160725944	9.65205877976674e-14	Disruptive
C4B63_81g59	1.31030262235231	1.038014808831e-13	Core
C4B63_5g221	-4.28365788095817	1.0763981785789e-13	Disruptive
C4B63_138g21	1.04713762082692	1.0792189026046e-13	Core
C4B63_38g61	-1.22758473643164	1.10347658691342e-13	Core
C4B63_399g10	-1.84852780396249	1.11895546864847e-13	Disruptive
C4B63_1g495	-2.57721706500265	1.18155180974262e-13	Disruptive
C4B63_71g114	-4.05710477235885	1.22380421501908e-13	Disruptive
C4B63_3g1037	-1.99118915280638	1.31399274594232e-13	Disruptive
C4B63_53g181	-2.75792959933923	1.38078703500969e-13	Disruptive
C4B63_76g18	-2.08470331399382	1.38078703500969e-13	Core
C4B63_3g1033	-2.6464924898086	1.39927556944023e-13	Disruptive
C4B63_29g283	-2.40559441426361	1.40320694944654e-13	Disruptive
C4B63_23g241	1.21032197590101	1.40592851499872e-13	Core
C4B63_44g163	-1.73565418355495	1.40628790014329e-13	Core
C4B63_34g140	-2.59697812411571	1.4795790601109e-13	Disruptive
C4B63_16g142	-2.26488393522602	1.48235440738408e-13	Disruptive
C4B63_6g577	1.06981070805343	1.48601515288572e-13	Core
C4B63_117g67	-3.01058881592203	1.52355283312093e-13	Disruptive
C4B63_3g300	-3.19584458849892	1.55591924427371e-13	Disruptive
C4B63_12g20	1.47987228508241	1.611479483831e-13	Core
C4B63_139g14	-2.61213173804954	1.62167044374293e-13	Disruptive
C4B63_142g5	1.67148601885794	1.6410796830778e-13	Core

C4B63_37g134	-4.35814271709301	1.65663299811598e-13	Disruptive
C4B63_45g129	1.89862072383086	1.67337166942278e-13	Core
C4B63_36g149	-3.15660739214355	1.681552728937e-13	Disruptive
C4B63_1g671	-2.71573271785951	1.69681026482846e-13	Disruptive
C4B63_32g226	-2.30582466409181	1.78747558136346e-13	Core
C4B63_8g101	-2.38847316911196	1.79268822050144e-13	Disruptive
C4B63_66g170	-2.36436231813446	1.81542905094992e-13	Disruptive
C4B63_5g215	-2.10602443457927	1.83962140099024e-13	Disruptive
C4B63_2g153	-1.37926075789353	1.87007663719345e-13	Core
C4B63_431g7	1.52270008505116	2.02693871299881e-13	Core
C4B63_109g47	-2.04044488647828	2.07713125665577e-13	Disruptive
C4B63_2g4104c	2.74520484924318	2.11830696106399e-13	Core
C4B63_36g100	-1.93068778017355	2.13764735392533e-13	Disruptive
C4B63_323g5	-2.61937785058493	2.15553307864009e-13	Disruptive
C4B63_53g174	-1.82090431514045	2.15658878639747e-13	Core
C4B63_19g171	1.07726168276473	2.17147517331248e-13	Core
C4B63_128g59	1.96239609381635	2.17962147240552e-13	Core
C4B63_122g1	1.91506210183619	2.19802708269898e-13	Core
C4B63_3g870	-2.68069501770548	2.20930578665004e-13	Disruptive
C4B63_11g423	-2.41827424333515	2.23207788427866e-13	Disruptive
C4B63_21g6	1.55284077398869	2.56402635141251e-13	Core
C4B63_17g115	-1.20175881092683	2.57975858818719e-13	Core
C4B63_144g5	-2.43447450746555	2.59632730150686e-13	Disruptive
C4B63_29g21	-3.15463560978268	2.60023546329672e-13	Disruptive
C4B63_29g134	-2.31127482274897	2.65983434364973e-13	Disruptive
C4B63_15g33	-3.03128156649014	2.74920419390946e-13	Disruptive
C4B63_24g263	1.4921232506316	2.7713664065531e-13	Core
C4B63_67g107	-3.75070513927784	2.8071787489524e-13	Disruptive
C4B63_9g372	1.90283576192508	2.99019287123591e-13	Core
C4B63_54g84	-2.24204550791367	3.00238889965853e-13	Disruptive
C4B63_6g2366c	1.14445840378579	3.00340105626354e-13	Core
C4B63_5g39	-2.71862023278364	3.35993734208541e-13	Disruptive
C4B63_30g261	1.54423197927564	3.79522071450475e-13	Core
C4B63_120g23	-3.29426910419581	3.92369812238788e-13	Disruptive
C4B63_219g34	2.22077285190266	4.06445635709548e-13	Core
C4B63_33g273	-2.24300844691723	4.0827452359934e-13	Disruptive
C4B63_4g423	-1.40313249352063	4.12509329625656e-13	Core
C4B63_1g1145	-2.71288326017452	4.27205704805094e-13	Disruptive
C4B63_156g11	1.65383771853609	4.42702633321028e-13	Core
C4B63_62g157	-3.55416442147579	4.81521436715734e-13	Disruptive
C4B63_95g6	-1.05553575813876	4.88017826656159e-13	Disruptive
C4B63_31g94	-1.82261649484567	5.04768162121491e-13	Core
C4B63_13g25	-1.97153413546274	5.07245073227098e-13	Disruptive
C4B63_5g698	-2.50866768644294	5.38082314021532e-13	Disruptive
C4B63_170g23	-1.7396933723129	5.38666816267341e-13	Core
C4B63_50g132	-2.76196410342646	5.39794018024077e-13	Disruptive
C4B63_33g64	-2.52787886817124	5.46762568504571e-13	Disruptive
C4B63_29g34	-2.58597493639914	5.48377753978127e-13	Disruptive
C4B63_1g1002	-2.31075995827747	5.6083650733806e-13	Disruptive
C4B63_2g1604c	1.31109651098264	5.69295610899355e-13	Core
C4B63_23g132	-1.53755785357167	5.81325140698444e-13	Core
C4B63_10g137	1.40329802084626	5.81522231298615e-13	Core
C4B63_20g208	1.41332687144714	5.81545383088407e-13	Core
C4B63_62g10	-1.91724534930259	5.97324666730139e-13	Core
C4B63_29g448	-2.01192156711111	6.03271277509329e-13	Disruptive
C4B63_106g40	1.60884284479756	6.15923960809853e-13	Core
C4B63_4g341	-1.03910132097131	6.57528918261397e-13	Core
C4B63_181g4	1.59004268234261	6.58816175823932e-13	Core
C4B63_3g455	-2.19104514340814	6.72368905538024e-13	Core
C4B63_3g207	-2.56851220437504	6.77761183653787e-13	Disruptive
C4B63_1g809	-2.90412004204547	7.51804173034502e-13	Disruptive
C4B63_109g54	-1.98714364099168	7.74954266611166e-13	Disruptive
C4B63_147g68	1.60327165968018	7.76010097744918e-13	Core
C4B63_12g330	1.02708372460484	8.08551714901243e-13	Core

C4B63_46g74	1.03716720789622	8.32393322714909e-13	Core
C4B63_88g23	-3.06340594128324	8.42616285783207e-13	Disruptive
C4B63_350g3	2.06524204663435	9.40801738326137e-13	Core
C4B63_116g4	1.23911733476977	9.43158561862663e-13	Core
C4B63_8g93	-2.24472867049126	9.72401973639802e-13	Disruptive
C4B63_34g285	1.87290527143932	1.01694303477607e-12	Core
C4B63_17g118	-1.38008542765127	1.03192500783402e-12	Core
C4B63_28g161	1.27419686226638	1.06318490018757e-12	Core
C4B63_73g70	1.37240404972132	1.06318490018757e-12	Core
C4B63_28g32	-1.11260622899497	1.06322063551106e-12	Core
C4B63_37g169	-2.93674777693388	1.1577544488892e-12	Disruptive
C4B63_106g53	-2.29751440222102	1.16573259779094e-12	Disruptive
C4B63_50g127	-2.8013096078076	1.21228455749623e-12	Disruptive
C4B63_37g98	-3.21760983308516	1.21789174973415e-12	Disruptive
C4B63_32g229	-2.0506258254811	1.23542706905217e-12	Core
C4B63_59g224	-2.23544348228894	1.25473050615927e-12	Disruptive
C4B63_5g184	-2.7123812912522	1.30422783357287e-12	Disruptive
C4B63_5g121	-3.1225646202221	1.33557228503901e-12	Disruptive
C4B63_1g94	-1.63489465403215	1.34025792425061e-12	Disruptive
C4B63_15g86	-2.9738494462783	1.40798042663986e-12	Disruptive
C4B63_18g19	1.56844340032813	1.42834668141235e-12	Core
C4B63_1g1342	-2.62405454816809	1.47519958076573e-12	Disruptive
C4B63_71g171	-2.69702066689987	1.50273193095764e-12	Disruptive
C4B63_319g10	-1.97708823936045	1.50428750863309e-12	Disruptive
C4B63_95g50	-1.20524798359066	1.55547758844597e-12	Core
C4B63_13g136	1.15387598932719	1.6267864203284e-12	Core
C4B63_23g18	1.42854496103687	1.6374711417362e-12	Core
C4B63_147g55	-2.22431009236169	1.64990328763937e-12	Disruptive
C4B63_10g293	-1.67599422791938	1.65689258242977e-12	Core
C4B63_13g287	-2.31099717230798	1.70334443995997e-12	Disruptive
C4B63_58g47	1.2613283554354	1.70334443995997e-12	Core
C4B63_27g82	-3.43877284796713	1.70376358270079e-12	Disruptive
C4B63_168g17	1.26630912765178	1.73274322379817e-12	Core
C4B63_2g306	1.35599381275543	1.74983502300312e-12	Core
C4B63_1g1315	-2.4841211310954	1.7568500923511e-12	Disruptive
C4B63_5g135	-2.7021222202166	1.7568500923511e-12	Disruptive
C4B63_303g6	1.38061382275178	1.83193156388817e-12	Core
C4B63_8g100	-2.28927253518028	1.9417821320454e-12	Disruptive
C4B63_5g308	-2.84317863301594	1.94241497034518e-12	Disruptive
C4B63_34g1506c	2.44937876160849	1.97711664634251e-12	Disruptive
C4B63_34g239	3.86873329325085	2.0024311750993e-12	Disruptive
C4B63_187g24	-1.83240085743883	2.0024311750993e-12	Disruptive
C4B63_152g41	1.57249891365843	2.04571358703112e-12	Core
C4B63_201g1	-3.66625027898009	2.04571903967301e-12	Disruptive
C4B63_36g231	-4.1521130433351	2.05632094769023e-12	Disruptive
C4B63_2g725	-1.21892808750426	2.0598577743865e-12	Core
C4B63_157g30	1.66689315561641	2.16785639813439e-12	Core
C4B63_1g1110	-2.61607652060758	2.18512060939278e-12	Disruptive
C4B63_4g335	1.24675768945591	2.23806650291992e-12	Core
C4B63_23g34	1.19572862225976	2.2945715847954e-12	Core
C4B63_27g327	-2.6083773636693	2.35355159520692e-12	Disruptive
C4B63_1g1102	-2.36643857223361	2.50524122973195e-12	Disruptive
C4B63_19g218	1.42333476618531	2.52979937710773e-12	Core
C4B63_23g21	1.74264691671984	2.65080597662725e-12	Core
C4B63_79g2	-2.03248834178394	2.72225382341962e-12	Core
C4B63_56g101	1.70991818295485	2.89252114736947e-12	Core
C4B63_57g42	-3.17538142771061	2.90910213760842e-12	Disruptive
C4B63_77g10	1.36929208694128	2.92391837132006e-12	Core
C4B63_197g25	-1.81753267347733	3.16129209743437e-12	Disruptive
C4B63_19g184	1.48342332002958	3.25888094472054e-12	Core
C4B63_64g79	-2.52357352494207	3.41365458301352e-12	Disruptive
C4B63_66g18	-2.69125451866208	3.45697833348321e-12	Disruptive
C4B63_118g25	-3.3988676672188	3.45697833348321e-12	Disruptive
C4B63_30g187	1.53032009258762	3.46760382222836e-12	Core

C4B63_23g75	1.45576142909732	3.54566210302586e-12	Core
C4B63_31g226	1.48233957272648	3.61236445524806e-12	Core
C4B63_104g101	3.11147923685871	3.74805131240217e-12	Core
C4B63_163g21	1.7050464479257	3.80432383711447e-12	Core
C4B63_105g68	1.39299385699847	3.92833532807558e-12	Core
C4B63_49g107	-1.08532111329674	4.18221975596282e-12	Core
C4B63_170g15	1.16238355254668	4.24345835366706e-12	Core
C4B63_32g40	1.72571080385185	4.27408318925369e-12	Core
C4B63_15g133	-2.00346238134502	4.27681659996391e-12	Disruptive
C4B63_3g1066	-3.20811116911669	4.33383711609727e-12	Disruptive
C4B63_266g13	1.57962891546306	4.53205084598775e-12	Core
C4B63_13g193	1.39857483215045	4.63231133760844e-12	Core
C4B63_62g175	-2.66115732657497	4.71659724365319e-12	Disruptive
C4B63_122g20	2.01786109030214	4.87379475340552e-12	Core
C4B63_8g168	-2.51318257554385	4.87524238474935e-12	Disruptive
C4B63_294g8	2.20821395208429	5.37283721997524e-12	Core
C4B63_126g3	-1.81752102071226	5.44851530334463e-12	Disruptive
C4B63_1g1263	-2.61807046695931	5.86560653906479e-12	Disruptive
C4B63_235g14	-1.95316928033342	5.86560653906479e-12	Core
C4B63_29g59	-2.87783096247826	6.02549290447911e-12	Disruptive
C4B63_24g313	1.45534929271389	6.0353463992076e-12	Core
C4B63_48g133	-1.5117322411158	6.0353463992076e-12	Core
C4B63_5g787	-1.21383428537324	6.43433483545045e-12	Disruptive
C4B63_386g12	-2.58063918312821	6.70352704617042e-12	Disruptive
C4B63_66g146	-2.49293586933864	6.71496526655869e-12	Disruptive
C4B63_5g256	-2.97306002132443	6.75473986175757e-12	Disruptive
C4B63_20g324	-1.65572795223723	7.19793902087667e-12	Core
C4B63_26g126	1.16201136846264	7.27382263068789e-12	Core
C4B63_174g17	-2.38338184070537	7.50846310449508e-12	Disruptive
C4B63_7g224	1.30704217229387	7.5137783977794e-12	Core
C4B63_61g146	-1.36446494671749	7.6785528738196e-12	Core
C4B63_7g443	1.23721683095199	7.73759174009015e-12	Core
C4B63_132g15	-2.68532228231666	7.78914350388363e-12	Disruptive
C4B63_15g223	-2.76013700096227	7.80816387729981e-12	Disruptive
C4B63_156g14	-1.94055833667854	7.82866374109444e-12	Core
C4B63_37g205	-4.28548151985665	7.9111647914101e-12	Disruptive
C4B63_1g406	-2.99827327961749	7.95827333910621e-12	Disruptive
C4B63_140g6	1.19467939142073	8.44586106426571e-12	Core
C4B63_358g15	2.32671800828097	8.60730372377284e-12	Core
C4B63_142g14	1.40685371609951	9.07992198879552e-12	Core
C4B63_386g23	-2.04202575608629	9.10350551315856e-12	Disruptive
C4B63_29g366	-2.67790104685074	9.20315596315105e-12	Disruptive
C4B63_51g221	-2.9096459258587	9.34401247185649e-12	Disruptive
C4B63_75g84	-1.55761125292421	9.39859334373578e-12	Disruptive
C4B63_28g225	3.26375618016914	9.54426172282921e-12	Core
C4B63_63g40	-1.74120679156071	9.87551110501163e-12	Core
C4B63_138g2	1.8599375263544	1.02736762548039e-11	Core
C4B63_34g307	1.19800968329372	1.09518367854922e-11	Core
C4B63_31g89	-1.5910576069855	1.12959042932273e-11	Core
C4B63_45g130	1.90473088435664	1.16018802503229e-11	Core
C4B63_3g575	-2.65173669709516	1.17000455070729e-11	Disruptive
C4B63_21g249	-1.44575989078931	1.17292565655872e-11	Core
C4B63_87g42	1.56198729424806	1.18243310668025e-11	Core
C4B63_8g2697c	1.54473134004437	1.30710031773048e-11	Core
C4B63_10g530	1.68188823022507	1.30810104628128e-11	Core
C4B63_180g11	-1.49612886646303	1.30810104628128e-11	Disruptive
C4B63_17g102	1.73897755431899	1.3338154225902e-11	Core
C4B63_275g7	-1.45214181176956	1.39418054110407e-11	Core
C4B63_62g182	-2.61648024360746	1.39951311624966e-11	Disruptive
C4B63_15g156	-2.2997068124992	1.45708757406339e-11	Disruptive
C4B63_330g9	1.28882766644237	1.48966832677368e-11	Core
C4B63_8g259	-3.10378546485173	1.58404652077658e-11	Disruptive
C4B63_408g13	-10.8792753044584	1.60084666961526e-11	Disruptive
C4B63_3g560	-2.64838695976116	1.62580691773156e-11	Disruptive

C4B63_97g37	1.52858268318543	1.69114722879528e-11	Core
C4B63_1g1324	-2.83561493255462	1.73394565454524e-11	Disruptive
C4B63_57g83	1.07404968478694	1.74072981671677e-11	Core
C4B63_65g124	-2.15038516096482	1.75686024006973e-11	Disruptive
C4B63_35g148	-1.51224462768279	1.8040261289199e-11	Disruptive
C4B63_7g118	-1.38032470098639	1.89043794046277e-11	Core
C4B63_5g34	-2.0216388901853	1.91617034979538e-11	Disruptive
C4B63_60g149	1.0019539970468	1.93946716831254e-11	Core
C4B63_43g3	1.04596072673643	2.0232617891779e-11	Core
C4B63_37g189	-1.97290463586911	2.07089484164446e-11	Disruptive
C4B63_25g200	-2.83578923082539	2.07552785846328e-11	Disruptive
C4B63_5g367	-2.08128529205832	2.12753445662798e-11	Disruptive
C4B63_112g23	1.42285691442243	2.12875412870775e-11	Core
C4B63_90g81	-2.59425307065515	2.22009112324762e-11	Disruptive
C4B63_26g274	1.0838617133275	2.2509809460583e-11	Core
C4B63_15g116	-2.76984838581307	2.27577034450681e-11	Disruptive
C4B63_121g35	-2.27701696965114	2.29359147981393e-11	Disruptive
C4B63_101g68	-2.59819939981058	2.34261711309618e-11	Disruptive
C4B63_145g29	-1.00940209742976	2.46140784518853e-11	Core
C4B63_14g110	1.06253359572698	2.46754299748803e-11	Core
C4B63_104g99	2.92509019687287	2.47432887420593e-11	Core
C4B63_87g29	2.00017538839687	2.56985595925087e-11	Core
C4B63_33g43	-2.84059206647668	2.57213306954861e-11	Disruptive
C4B63_39g312	1.81643470264117	2.60865541348628e-11	Core
C4B63_1g839	-2.39946746856684	2.61630206686437e-11	Disruptive
C4B63_7g116	-1.22318962582186	2.63303251652809e-11	Core
C4B63_2g362	1.11505807320191	2.68230941832655e-11	Core
C4B63_50g98	-2.45685604279708	2.89740406939307e-11	Disruptive
C4B63_34g331	2.21555448028361	2.90137599717572e-11	Disruptive
C4B63_3g380	-3.31436140626713	3.08535357195865e-11	Disruptive
C4B63_32g209	1.95598127957497	3.11142955203212e-11	Core
C4B63_25g331	1.97306696602955	3.18717795007502e-11	Disruptive
C4B63_55g177	1.19535057722848	3.38657832049606e-11	Core
C4B63_44g215	-1.03847885086432	3.3984501306386e-11	Core
C4B63_14g113	-1.47233966582613	3.4249334546803e-11	Core
C4B63_52g143	1.21602201652986	3.47565492490773e-11	Core
C4B63_5g90	-2.79055595713369	3.52127754939373e-11	Disruptive
C4B63_72g84	-2.01412516672357	3.62161481243974e-11	Core
C4B63_60g159	1.64446701965039	3.76674510234265e-11	Core
C4B63_1g813	-2.55109158316619	3.85371205116786e-11	Disruptive
C4B63_1g491	-2.93875869941425	3.93485003033182e-11	Disruptive
C4B63_37g143	-2.22376042345347	4.20893127227554e-11	Disruptive
C4B63_96g90	-2.49962727800908	4.25344614914142e-11	Disruptive
C4B63_12g334	1.49300176803331	4.46638336887482e-11	Core
C4B63_25g186	1.54709548223343	4.52618219346103e-11	Core
C4B63_64g195	-3.46371078961196	4.56081156775888e-11	Disruptive
C4B63_42g180	1.41802903600304	4.99991138379432e-11	Core
C4B63_249g16	1.92169834861734	5.21619557004555e-11	Core
C4B63_167g34	1.19055434224528	5.22588218699127e-11	Core
C4B63_1g1134	-2.73876643678539	5.26461116997126e-11	Disruptive
C4B63_27g167	-1.80031982823479	5.32710268598498e-11	Core
C4B63_52g80	1.54298353595534	5.32710268598498e-11	Core
C4B63_64g55	-2.16080212547467	5.39772084733814e-11	Disruptive
C4B63_19g220	1.63393237070138	5.41853792052413e-11	Core
C4B63_110g6	1.68434764811591	5.6123787868885e-11	Core
C4B63_11g147	-2.45281949792633	5.6251883176244e-11	Disruptive
C4B63_142g27	1.26774443764876	5.75211343903922e-11	Core
C4B63_79g92	1.86550608231502	5.77533724063969e-11	Core
C4B63_21g40	-1.03251256966553	5.87340393287897e-11	Core
C4B63_26g271	1.24802101629602	5.96559180717646e-11	Core
C4B63_1g607	-2.33172460828183	5.98484121039719e-11	Disruptive
C4B63_38g259	2.31592605645105	5.98748468666002e-11	Core
C4B63_23g274	-1.02863778627946	6.23105065059793e-11	Core
C4B63_39g137	-2.19055057165153	6.29446308582362e-11	Disruptive

C4B63_11g220	1.23256233104955	6.31136791132421e-11	Core
C4B63_309g17	-2.23799440313885	6.36126615864168e-11	Disruptive
C4B63_23g17	2.12194165685464	6.38875749321206e-11	Core
C4B63_1g558	-1.49276484410377	6.5596661123882e-11	Core
C4B63_68g98	-1.9950934513247	6.57333105962187e-11	Disruptive
C4B63_9g525	1.31396295688378	6.62372345658041e-11	Core
C4B63_86g61	-2.2218520796818	6.83346665715728e-11	Disruptive
C4B63_9g511	1.3548476123618	6.99654375539873e-11	Core
C4B63_36g363	-2.89954610066111	7.11122969618167e-11	Disruptive
C4B63_310g9	-2.09410359489484	7.22632820465799e-11	Disruptive
C4B63_2g633	-2.67275013061548	7.66265053768066e-11	Disruptive
C4B63_63g44c	1.34207451092992	7.78192452539278e-11	Core
C4B63_3g580	-1.88333901571809	7.86303980428973e-11	Disruptive
C4B63_78g31	1.09221674102587	8.03444746551678e-11	Core
C4B63_19g219	1.74838647012858	8.0493153873553e-11	Core
C4B63_1g676	-2.6848811443771	8.20103940311521e-11	Disruptive
C4B63_27g56	-2.70485609421259	8.225495729582e-11	Disruptive
C4B63_11g57	1.04738296219539	8.5916080675064e-11	Core
C4B63_5g210	-2.07915781790136	8.59831523095042e-11	Disruptive
C4B63_11g305c	1.00576173952381	8.61411710626182e-11	Core
C4B63_1g526	-2.80433359578785	8.71555121654515e-11	Disruptive
C4B63_74g44	-2.75446392794232	9.18918212772354e-11	Disruptive
C4B63_62g194	-2.49681349063779	9.20704849372766e-11	Disruptive
C4B63_60g82	-1.98179214079823	9.40666475793375e-11	Disruptive
C4B63_64g132	-3.41176916868818	9.63355757770141e-11	Disruptive
C4B63_3g743	-3.94956023531914	9.89991233528482e-11	Disruptive
C4B63_44g158	-1.85833919942557	1.06101881060777e-10	Core
C4B63_14g126	-2.18166034821854	1.07668465061505e-10	Disruptive
C4B63_44g132	1.06749223747621	1.08355897380481e-10	Core
C4B63_135g35	-1.9900404338784	1.1209966810235e-10	Disruptive
C4B63_94g53	1.50618145567354	1.15903799083286e-10	Core
C4B63_41g123	-1.45781597175021	1.18310825431842e-10	Core
C4B63_1g968	-2.08975659230627	1.3511896266026e-10	Disruptive
C4B63_30g140	1.30335298510183	1.35607165981922e-10	Core
C4B63_29g16	-2.58110383028618	1.36090678987299e-10	Disruptive
C4B63_63g52	1.06293339746576	1.3918143381298e-10	Core
C4B63_23g238	1.07250368899827	1.41749424339516e-10	Core
C4B63_31g228	1.5431757460888	1.43848853567347e-10	Core
C4B63_49g116	1.32875142248436	1.45946753536225e-10	Core
C4B63_77g15	1.21598320211305	1.46189803055075e-10	Core
C4B63_3g574	-1.96338200294613	1.4800888846001e-10	Disruptive
C4B63_37g109	-2.46622405837353	1.50480349972932e-10	Disruptive
C4B63_29g223	1.58885733246847	1.53649061803607e-10	Disruptive
C4B63_40g176	-1.67830075541329	1.53676872251543e-10	Disruptive
C4B63_76g16	1.34454234071938	1.54233356470801e-10	Core
C4B63_34g333	2.47551205705721	1.5790902357391e-10	Disruptive
C4B63_244g5	-1.31580406997109	1.60104580588027e-10	Core
C4B63_50g151	-2.4029641778928	1.60209112798327e-10	Disruptive
C4B63_341g15	-2.35321781612645	1.61068809191678e-10	Core
C4B63_22g175	1.1897949308537	1.62922562571673e-10	Core
C4B63_3g769	-2.18056686064932	1.64350649319136e-10	Disruptive
C4B63_169g46	-3.24445736146265	1.6563391778348e-10	Disruptive
C4B63_17g85	1.71455526795333	1.68687560826996e-10	Core
C4B63_35g9	-2.50204885925272	1.6952567651988e-10	Core
C4B63_62g165	-3.10689981675292	1.73602725337389e-10	Disruptive
C4B63_39g88	-2.23725355382306	1.73703272246057e-10	Disruptive
C4B63_2g693	1.47520544504921	1.74941755327641e-10	Core
C4B63_47g160	-1.74561573531712	1.8622303606766e-10	Disruptive
C4B63_1g300	-1.57873385176834	1.86585850309583e-10	Disruptive
C4B63_319g9	-1.49270051115266	1.90654957847102e-10	Disruptive
C4B63_37g215	-4.01675976435485	1.93049231182761e-10	Disruptive
C4B63_96g56	-2.99360892937234	2.12064406294803e-10	Disruptive
C4B63_14g80	-1.03831417603662	2.13539564299819e-10	Core
C4B63_8g300	1.21864948814228	2.18354223091657e-10	Core

C4B63_65g149	-1.97099343777796	2.26769186782337e-10	Disruptive
C4B63_37g121	-1.80972201972602	2.32216468878364e-10	Disruptive
C4B63_120g71	1.43151245777948	2.34901967219917e-10	Core
C4B63_15g317	-2.71297659759031	2.44514381333918e-10	Disruptive
C4B63_32g50	1.30301585620675	2.5146353131457e-10	Core
C4B63_305g8	-1.74291991863177	2.54824462528103e-10	Disruptive
C4B63_11g25	-1.27650542888242	2.72486745741306e-10	Core
C4B63_39g65	-2.72403771089418	2.72877059794212e-10	Disruptive
C4B63_8g487	1.12807861910598	2.75198801527713e-10	Core
C4B63_211g37	2.05199318682783	2.78131839846532e-10	Core
C4B63_4g242	-1.28316667365748	2.79355653655606e-10	Core
C4B63_132g13	-2.67458489664511	2.83582808434651e-10	Disruptive
C4B63_9g273	1.57488601432083	2.8409332569412e-10	Core
C4B63_96g81	-2.5379162108178	2.86668565861212e-10	Disruptive
C4B63_36g204	-2.07311972894107	2.87994011008481e-10	Disruptive
C4B63_3g1036	-2.554976519058	2.95864900319157e-10	Disruptive
C4B63_3g1092	1.70235500934023	2.98073033787964e-10	Core
C4B63_106g72	-1.84460980257716	2.98642905264005e-10	Disruptive
C4B63_31g256	-2.11872527472296	3.09350995693143e-10	Disruptive
C4B63_1g957	-2.51674400377925	3.11679267434591e-10	Disruptive
C4B63_20g204	1.30031591318843	3.20332138656206e-10	Core
C4B63_72g28	-1.69927375102176	3.29978193813246e-10	Disruptive
C4B63_37g114	-2.26197062689938	3.38126771592697e-10	Disruptive
C4B63_44g188	-1.8802288066336	3.46065528409369e-10	Core
C4B63_2g783	1.29007940916414	3.47699520964629e-10	Core
C4B63_18g811c	1.17017094202014	3.51041646202548e-10	Core
C4B63_3g1046	-2.55552850942069	3.52029372978667e-10	Disruptive
C4B63_5g692	-2.25867853661166	3.65153078072859e-10	Disruptive
C4B63_3g73	-3.38409866660612	3.66304585819219e-10	Disruptive
C4B63_15g308	-2.3437772143677	3.70747679189989e-10	Disruptive
C4B63_17g208	-10.2540238252624	3.71445628279246e-10	Disruptive
C4B63_30g290	-1.53232989102946	3.73486297701208e-10	Core
C4B63_2g318	1.51959561414429	3.80419241608288e-10	Core
C4B63_3g779	-2.35243012589455	4.14363521337838e-10	Disruptive
C4B63_1g1244	-2.72441302945606	4.35819184825017e-10	Disruptive
C4B63_37g106	-2.06908384222758	4.35819184825017e-10	Disruptive
C4B63_123g31	-1.58891712235766	4.35819184825017e-10	Core
C4B63_67g29	-2.28775948542925	4.42830834020204e-10	Disruptive
C4B63_29g133	-1.8271669586239	4.43477056227949e-10	Disruptive
C4B63_29g56	-1.9556364015832	4.47472034836419e-10	Disruptive
C4B63_8g524	-1.36178463128918	4.51072083078009e-10	Core
C4B63_42g47	1.45173675912944	4.53728075462336e-10	Core
C4B63_223g15	-2.93590248064447	4.53728075462336e-10	Disruptive
C4B63_44g216	-1.36125927530716	4.74781231277823e-10	Core
C4B63_21g90	1.10769412126311	4.74791130810824e-10	Core
C4B63_43g160	1.20023402721705	4.82573087649463e-10	Core
C4B63_99g97	-1.88306076041232	5.03129185329554e-10	Disruptive
C4B63_142g26	1.51781561455472	5.0634001680753e-10	Core
C4B63_17g270	1.04158250927775	5.1212911326357e-10	Core
C4B63_60g170	-1.11248693492292	5.23492455407785e-10	Core
C4B63_2g33	1.14333687227105	5.27288629337271e-10	Core
C4B63_8g82	-1.88599031674218	5.39900205476765e-10	Disruptive
C4B63_21g239c	1.2277689643225	5.73056184148887e-10	Core
C4B63_7g340	-1.66318006358268	5.80480058159215e-10	Core
C4B63_48g3	1.26338741442728	5.86769856677967e-10	Core
C4B63_88g101	-2.37223709487847	5.88458687131147e-10	Disruptive
C4B63_41g152	-2.11549569188285	5.94433477068969e-10	Core
C4B63_3g1044	-2.06674500144291	6.01035573110096e-10	Disruptive
C4B63_5g130	-2.60339718879533	6.11057410972707e-10	Disruptive
C4B63_30g161	-1.29664936071036	6.12137329406848e-10	Core
C4B63_47g165	1.16319025596311	6.39414396641364e-10	Disruptive
C4B63_110g7	1.34238682588075	6.40870089247102e-10	Core
C4B63_42g42	1.21083640053521	6.58573359821617e-10	Core
C4B63_244g7	1.79401494228556	7.05360433313942e-10	Disruptive

C4B63_67g69	-3.08361265560492	7.09560487452995e-10	Disruptive
C4B63_43g180	1.02609490143451	7.28657342459852e-10	Core
C4B63_5g349	-2.06192196611881	7.38436171708104e-10	Disruptive
C4B63_3g865	-2.05450518738501	7.46862501562861e-10	Disruptive
C4B63_222g5	-2.31194266795862	7.54226889871883e-10	Disruptive
C4B63_28g159	1.05476150783074	7.95420837642435e-10	Core
C4B63_238g17	1.61246234315573	8.01670000898312e-10	Core
C4B63_6g145	1.12476242252498	8.12887383374092e-10	Core
C4B63_403g4	1.32818667437566	8.14193867155791e-10	Core
C4B63_15g369	-2.80556188820855	8.14227553807419e-10	Disruptive
C4B63_7g93	1.43821543783652	8.29186845617927e-10	Core
C4B63_19g98	1.67146450526983	8.39647628539951e-10	Core
C4B63_76g31	1.00501059692974	8.60450755455775e-10	Core
C4B63_61g127	1.65003859300655	9.05593252119631e-10	Core
C4B63_15g455	-2.27032720263694	9.28043735488072e-10	Disruptive
C4B63_37g89	-1.98390523418789	9.29755801739146e-10	Disruptive
C4B63_280g17	1.09119489291985	9.38107539135543e-10	Core
C4B63_5g731	-3.02078418119095	9.41277255755168e-10	Disruptive
C4B63_41g217	1.68347514850723	9.49601139365659e-10	Core
C4B63_59g45	-2.35880776655959	9.53132866293801e-10	Disruptive
C4B63_40g189	1.01481629425486	9.99689114620759e-10	Core
C4B63_8g251	-3.76664298622982	1.03380833251796e-09	Disruptive
C4B63_14g138	-2.14430406563804	1.05069591006885e-09	Disruptive
C4B63_91g67	-3.14447168205948	1.05269077041028e-09	Disruptive
C4B63_4g422	-1.25237014867033	1.05676353044706e-09	Core
C4B63_131g50	-2.2235117319832	1.09105261140786e-09	Disruptive
C4B63_50g230	-2.29807407889868	1.11376061164261e-09	Disruptive
C4B63_320g3	-2.951185837387	1.13364247715244e-09	Disruptive
C4B63_334g34	-3.23355784552796	1.13771200929799e-09	Disruptive
C4B63_11g281	-2.6281994758051	1.15686910214426e-09	Disruptive
C4B63_2g320	1.04164755735044	1.15772391062702e-09	Core
C4B63_241g24	1.10681665772707	1.16175798449248e-09	Core
C4B63_186g23	2.2867074723271	1.1865926325409e-09	Core
C4B63_16g120	1.17943029299688	1.20768736021947e-09	Core
C4B63_37g122	-2.28767313358833	1.23156220955887e-09	Disruptive
C4B63_8g2652c	2.51646948330967	1.24454118006311e-09	Core
C4B63_64g141	-1.66676999241883	1.27204358376225e-09	Disruptive
C4B63_40g177	-3.9067934528647	1.36306084459295e-09	Disruptive
C4B63_125g60	-3.19370808784192	1.38985034899473e-09	Disruptive
C4B63_71g188	-2.25953190799237	1.40341310500753e-09	Disruptive
C4B63_23g4	2.16470072578829	1.40598083293045e-09	Core
C4B63_1g1084	-1.86523316403884	1.40919715662668e-09	Disruptive
C4B63_51g112	1.21935748008162	1.43784444738829e-09	Core
C4B63_5g699	-2.81097921307486	1.44242950809818e-09	Disruptive
C4B63_128g301c	1.89892594418186	1.46615570688906e-09	Core
C4B63_22g103	1.03236515625524	1.50593701027594e-09	Core
C4B63_91g39	1.28195911214592	1.51517227602273e-09	Core
C4B63_1g1238	-2.0485436908271	1.53489824139771e-09	Disruptive
C4B63_1g929	-2.91273826014932	1.56103097190200e-09	Disruptive
C4B63_5g225	-5.02333024750218	1.59150582114016e-09	Disruptive
C4B63_37g68	-1.72652400371225	1.59699634017899e-09	Disruptive
C4B63_8g92	-2.00013440205153	1.62649055106633e-09	Disruptive
C4B63_96g86	-2.33139376281739	1.63912870644495e-09	Disruptive
C4B63_387g12	-1.25888118173311	1.70185033155646e-09	Disruptive
C4B63_44g232	1.56833244367641	1.71819403149427e-09	Core
C4B63_201g2	-3.75488136621008	1.74552989883843e-09	Disruptive
C4B63_15g306	-3.06397046107713	1.75551315984748e-09	Disruptive
C4B63_36g230	-2.31438202593529	1.76437594414476e-09	Disruptive
C4B63_45g139	1.86000611199107	1.80759579104483e-09	Core
C4B63_102g62	1.46829983959613	1.8333569661897e-09	Core
C4B63_159g17	1.25130969075509	1.84969266707766e-09	Core
C4B63_5g388	-2.18637862224334	1.88725630785116e-09	Disruptive
C4B63_26g358	-1.52226025391425	1.9061613691404e-09	Disruptive
C4B63_89g67	1.40595263010039	1.92510079927515e-09	Core

C4B63_70g78	-1.19934541892864	1.94564236461297e-09	Core
C4B63_3g766	-2.12744407613455	1.96621766381014e-09	Disruptive
C4B63_27g296	-2.39524153613727	1.97935312527244e-09	Disruptive
C4B63_3g80	-2.66118477192887	1.99905992372161e-09	Disruptive
C4B63_29g330	-2.13611717146382	1.99905992372161e-09	Disruptive
C4B63_93g39	-1.52245614597591	2.0343577602254e-09	Core
C4B63_1g44	-1.40156014841814	2.13585083932917e-09	Core
C4B63_39g157	-2.28404187354135	2.14747609309824e-09	Disruptive
C4B63_8g235	-2.29379501986694	2.16637503250566e-09	Disruptive
C4B63_37g415	-3.59228449341175	2.1773423236143e-09	Disruptive
C4B63_61g147	1.53259243588866	2.19123940330482e-09	Core
C4B63_66g56	-2.52809316992004	2.210806354188e-09	Disruptive
C4B63_3g932	-2.53097482266928	2.23818740671354e-09	Disruptive
C4B63_244g6	1.41269374423233	2.24996370641038e-09	Disruptive
C4B63_1g225	-2.4466535929978	2.28087756011244e-09	Disruptive
C4B63_34g161	1.93324430763075	2.28087756011244e-09	Disruptive
C4B63_3g448	-2.38253525344207	2.33109796910935e-09	Disruptive
C4B63_124g13	-2.29416759184046	2.37970962618176e-09	Disruptive
C4B63_8g539	1.19350929585118	2.38334029611625e-09	Core
C4B63_29g4	1.17681528647991	2.38898067158573e-09	Core
C4B63_1g1078	-2.32754193555244	2.39431675764393e-09	Disruptive
C4B63_1g562	-2.05252757866391	2.41685150557307e-09	Disruptive
C4B63_196g44	-1.89940673209431	2.45080563736197e-09	Disruptive
C4B63_13g111	-1.0333610680352	2.5359131460034e-09	Core
C4B63_41g231	-1.16618868190605	2.54810068211096e-09	Core
C4B63_3g567	-2.21019920393007	2.57451526190349e-09	Disruptive
C4B63_314g13	1.49170777682463	2.64270825380907e-09	Core
C4B63_52g138	1.8482283422762	2.6617228667465e-09	Core
C4B63_1g1198	-2.90937115367389	2.691451464364e-09	Disruptive
C4B63_139g13	-3.15130161314401	2.691451464364e-09	Disruptive
C4B63_53g59	-2.69007866747249	2.72678844106588e-09	Disruptive
C4B63_32g53	1.84261888392026	2.78851692739385e-09	Core
C4B63_23g270	1.09379031327435	2.84783937983587e-09	Core
C4B63_33g148	-1.68281075333251	2.90992561865674e-09	Disruptive
C4B63_13g272	1.2138629988128	2.96382693029544e-09	Core
C4B63_121g27	-2.03653023256616	3.03285183915512e-09	Disruptive
C4B63_45g128	1.74375455056247	3.14401877312642e-09	Core
C4B63_5g274	-2.4983190250338	3.1555775903512e-09	Disruptive
C4B63_5g151	-2.31173109282733	3.18026346548169e-09	Disruptive
C4B63_3g1071	-3.61933966632814	3.18420329194077e-09	Disruptive
C4B63_1g1320	-2.78760525781823	3.24244893393481e-09	Disruptive
C4B63_10g529	1.11168701696312	3.24298933168498e-09	Core
C4B63_12g345	1.01054173146037	3.29295011801956e-09	Core
C4B63_108g11	1.17084246312402	3.32941160948579e-09	Core
C4B63_125g16	-1.76217420065783	3.32941160948579e-09	Disruptive
C4B63_10g445	1.47458720699589	3.35530405553327e-09	Core
C4B63_4g526	-1.22722642440622	3.37633614529888e-09	Core
C4B63_41g120	-1.88885175693337	3.38679168802319e-09	Core
C4B63_40g178	-1.80332817153439	3.52917849022214e-09	Disruptive
C4B63_33g5	1.69305848618797	3.53811074660673e-09	Core
C4B63_246g15	1.74230763177437	3.53811074660673e-09	Core
C4B63_66g144	-2.79372061729826	3.63666663588837e-09	Disruptive
C4B63_219g46	1.2700988136104	3.82123921712022e-09	Core
C4B63_3g957	-2.3519453367757	3.82737502031489e-09	Disruptive
C4B63_24g203	1.84963447815403	3.82804997152431e-09	Core
C4B63_13g22	-9.67854978953524	3.90916746583221e-09	Disruptive
C4B63_77g28	-1.739881245215	3.98911516805644e-09	Core
C4B63_5g251	-3.48403836842756	4.04312379575188e-09	Disruptive
C4B63_3g806	-2.04047275342302	4.21435665941183e-09	Disruptive
C4B63_50g145	-2.48933700174305	4.30171947692531e-09	Disruptive
C4B63_38g297	2.02920281551705	4.3364616175954e-09	Core
C4B63_175g9	-1.16313672126688	4.43732563341473e-09	Core
C4B63_6g2482c	1.37002575834636	4.45919765857752e-09	Core
C4B63_20g1412c	1.14321926134162	4.45919765857752e-09	Core

C4B63_64g133	-1.63036934187643	4.49014896721011e-09	Disruptive
C4B63_5g317	-2.34666330949178	4.51173199624316e-09	Disruptive
C4B63_126g4	-2.51685478825221	4.59988200615237e-09	Disruptive
C4B63_16g290	1.15897039785772	4.68378921103165e-09	Core
C4B63_144g10	-1.94282304577464	4.81779246604247e-09	Disruptive
C4B63_172g38	-1.90697978561462	4.97596211486314e-09	Disruptive
C4B63_6g113	1.05803531654031	5.06887532217899e-09	Core
C4B63_108g18	-1.47170342329646	5.26231664289383e-09	Disruptive
C4B63_56g41	2.06613797118057	5.30938248370949e-09	Core
C4B63_168g15	1.09050302571061	5.33677178450299e-09	Core
C4B63_23g237	-1.42266277065145	5.33770557013785e-09	Core
C4B63_35g375	1.07351230383078	5.55077852792511e-09	Core
C4B63_9g494	1.15987441182745	5.55253810629152e-09	Core
C4B63_33g61	-2.81241520318581	5.62607360749921e-09	Disruptive
C4B63_39g324	1.25660008439908	5.62718707350392e-09	Core
C4B63_27g261	-2.7761883907986	5.66609158060206e-09	Disruptive
C4B63_18g239	1.03349350495715	5.79134967456369e-09	Core
C4B63_193g17	1.47769360309345	5.79134967456369e-09	Core
C4B63_3g1128	-1.67948412564807	5.80837480470422e-09	Core
C4B63_86g44	-2.32863310873005	5.81007622259504e-09	Disruptive
C4B63_3g595	-2.43059228170504	5.81292005938293e-09	Disruptive
C4B63_66g51	-2.56575818705226	6.15125674888674e-09	Disruptive
C4B63_24g308	1.3063599448801	6.17925470071763e-09	Core
C4B63_10g523	1.03651032160871	6.21171242326281e-09	Core
C4B63_15g50	-2.17104956431339	6.29156452934344e-09	Disruptive
C4B63_89g47	1.29268684209496	6.50197747865823e-09	Core
C4B63_42g103	1.42876529352115	6.55686377877253e-09	Core
C4B63_1g382	-2.2749025683288	6.58279174467755e-09	Disruptive
C4B63_1g38	-1.84341469600854	6.69736489340553e-09	Core
C4B63_16g168	1.18580797242472	6.81476924566667e-09	Core
C4B63_4g171	1.23666971590222	7.31982702416564e-09	Core
C4B63_78g33	1.01561265538144	7.31982702416564e-09	Core
C4B63_71g189	-2.89814078097441	7.35433048091325e-09	Disruptive
C4B63_138g13	-1.33668296748153	7.40212197181981e-09	Disruptive
C4B63_12g362	-1.88957567354518	7.61417205805179e-09	Core
C4B63_118g10	-1.56212754178505	7.61417205805179e-09	Disruptive
C4B63_246g4	1.22138542049946	7.63401245813125e-09	Core
C4B63_8g253	-2.8978564315113	8.11525539132757e-09	Disruptive
C4B63_3g614	-2.28969722266926	8.21140337114173e-09	Disruptive
C4B63_180g9	-1.12434830128326	8.28294621465416e-09	Disruptive
C4B63_101g64	-2.13889160466058	8.33933733567832e-09	Disruptive
C4B63_100g59	-1.79445710645501	8.911794799841e-09	Disruptive
C4B63_24g169	-1.69421677679737	9.06929260759667e-09	Core
C4B63_52g79	-1.42202331820335	9.12798355314265e-09	Core
C4B63_15g353	-2.21158819244122	9.2197749739132e-09	Disruptive
C4B63_6g612	-2.94595234632185	9.27049380043922e-09	Disruptive
C4B63_16g6	-1.1196001058926	9.28839672109688e-09	Core
C4B63_8g240	-2.39455084117922	9.49546573705006e-09	Disruptive
C4B63_45g240	-1.55901742162167	9.53677428897512e-09	Core
C4B63_169g35	-1.96370267915291	9.58904135085617e-09	Disruptive
C4B63_16g78	-1.91509942189972	9.63345265525032e-09	Disruptive
C4B63_10g454	1.22822395025439	9.64816775127658e-09	Core
C4B63_68g97	-1.94431126342756	9.9183938705066e-09	Disruptive
C4B63_11g86	1.04868665037871	1.0352765724993e-08	Core
C4B63_76g52	1.1101628249684	1.05642318860112e-08	Core
C4B63_27g291	-2.50176248863658	1.0631990284282e-08	Disruptive
C4B63_3g911	-1.92166585859976	1.06435748037688e-08	Disruptive
C4B63_5g375	-1.85023447247077	1.06598048680383e-08	Disruptive
C4B63_116g34	-1.85156913657224	1.12049240286534e-08	Disruptive
C4B63_330g5	1.59121145894443	1.13571449066272e-08	Core
C4B63_235g16	-1.61564933016753	1.14693567323653e-08	Core
C4B63_11g124	-2.22619005336973	1.17355477856161e-08	Disruptive
C4B63_5g393	-2.0836829342996	1.18606564187567e-08	Disruptive
C4B63_71g132	-2.92784518045255	1.1877276146502e-08	Disruptive

C4B63_3g919	-2.39838993729626	1.22111190864304e-08	Core
C4B63_1g1255	-2.12453320121112	1.22128093639247e-08	Disruptive
C4B63_41g208	1.0654667842123	1.22456901587111e-08	Core
C4B63_29g84	-3.00652324824196	1.22471998713207e-08	Disruptive
C4B63_299g9	-1.94331983878171	1.22471998713207e-08	Disruptive
C4B63_24g287	-2.27198103009604	1.23205736674345e-08	Disruptive
C4B63_19g103	1.17388963592166	1.27675534345488e-08	Core
C4B63_105g51	-1.70796644810453	1.27675534345488e-08	Disruptive
C4B63_19g221	1.62393803403438	1.28450221205067e-08	Core
C4B63_10g459	1.11788152061069	1.29517036870599e-08	Core
C4B63_1g1079	-2.70831368631099	1.29531957581e-08	Disruptive
C4B63_142g4	2.09711581013922	1.30365411966669e-08	Core
C4B63_8g2705c	1.74733708446384	1.30786523224233e-08	Core
C4B63_121g86	-2.55003725048588	1.3111735811955e-08	Disruptive
C4B63_198g21	1.06179897614658	1.35351414178949e-08	Core
C4B63_11g316	-2.44619780752244	1.36559137371055e-08	Disruptive
C4B63_427g5	1.21613710426675	1.36767056179195e-08	Core
C4B63_544g7	1.31034205382668	1.39554170123181e-08	Core
C4B63_3g686	-3.01094157637496	1.39822340328274e-08	Disruptive
C4B63_313g5	-1.3699621799379	1.40373374651366e-08	Core
C4B63_1g1311	-2.0148070056626	1.42359836463611e-08	Disruptive
C4B63_39g98	-1.99822066207479	1.42549971322872e-08	Disruptive
C4B63_251g5	-1.8796127255195	1.42988038541817e-08	Core
C4B63_44g162	-1.68262660045563	1.43026994920795e-08	Core
C4B63_8g75	-1.90128963102495	1.45010359752727e-08	Disruptive
C4B63_5g156	-2.37823514385932	1.45609995804657e-08	Disruptive
C4B63_29g362	-2.09073608242449	1.47141993023896e-08	Disruptive
C4B63_5g371	-2.5245068235565	1.47941728317518e-08	Disruptive
C4B63_1g1316	-3.36933934437787	1.48057992506602e-08	Disruptive
C4B63_277g17	2.78460692579091	1.50015355825993e-08	Core
C4B63_139g23	-2.31389368250598	1.50675621201603e-08	Disruptive
C4B63_38g192	-1.20462904632734	1.51348321771918e-08	Disruptive
C4B63_28g226	-2.24082205348209	1.5159724841798e-08	Core
C4B63_60g133	1.49211135502373	1.52566741883668e-08	Core
C4B63_15g17	1.37383608818059	1.55738399463904e-08	Core
C4B63_57g129	1.23549299586236	1.55860021207102e-08	Core
C4B63_16g291	1.17232225511046	1.56551872850295e-08	Core
C4B63_34g379	-1.4269746089421	1.56618916212682e-08	Disruptive
C4B63_1g582	-1.89074573932548	1.56805942721851e-08	Disruptive
C4B63_112g25	1.07701174833361	1.58331966748368e-08	Core
C4B63_39g144	-2.68768035758723	1.59466335212366e-08	Disruptive
C4B63_40g179	-1.44308773039035	1.61128515276738e-08	Disruptive
C4B63_53g146	1.42677163960701	1.61128515276738e-08	Core
C4B63_99g68	-2.5603520649917	1.63146465487145e-08	Disruptive
C4B63_286g15	1.84553021745414	1.66436996801558e-08	Core
C4B63_29g69	-2.4969148775396	1.6661836439482e-08	Disruptive
C4B63_331g5	2.00666991325413	1.66632276761268e-08	Core
C4B63_23g147	1.03394376632858	1.70514282703786e-08	Core
C4B63_2g437	1.22525271870009	1.72481175523862e-08	Core
C4B63_39g128	-1.92184293643354	1.7290278465432e-08	Disruptive
C4B63_39g110	-1.93809233043185	1.73873887644796e-08	Disruptive
C4B63_2g409	1.04750180028659	1.74841765321347e-08	Core
C4B63_57g39	-2.1804898524544	1.75009908199901e-08	Disruptive
C4B63_60g198	1.4182442447503	1.76479582547805e-08	Core
C4B63_16g94	1.01989879314078	1.77513761117352e-08	Core
C4B63_15g356	1.11461845582774	1.79059999308849e-08	Core
C4B63_72g22	1.25671066510508	1.79059999308849e-08	Core
C4B63_224g19	-1.71348160785813	1.79473039814115e-08	Core
C4B63_6g284	1.56085735875348	1.80586609436744e-08	Core
C4B63_154g15	-2.11484239785932	1.8197106226635e-08	Disruptive
C4B63_188g152c	1.85137625489551	1.82074988361768e-08	Core
C4B63_5g227	-2.84170034299919	1.85805711462598e-08	Disruptive
C4B63_10g453	1.27491916475845	1.87206172414775e-08	Core
C4B63_16g182	-1.69704185598055	1.94384737425008e-08	Disruptive

C4B63_29g141	-2.00195512200098	1.96012398625144e-08	Disruptive
C4B63_15g158	-2.60410306484154	1.97164979129942e-08	Disruptive
C4B63_1g997	-2.00820536435091	1.98963585362391e-08	Disruptive
C4B63_1g732	-2.39202186895	2.09491930447781e-08	Disruptive
C4B63_153g40	1.2982380992264	2.10540935241321e-08	Core
C4B63_58g70	1.49289037544659	2.12307061930903e-08	Core
C4B63_5g298	-2.22404228888274	2.161144353362e-08	Disruptive
C4B63_14g129	-2.2453933971732	2.17785255345473e-08	Disruptive
C4B63_133g46	1.81721067046841	2.20372614953208e-08	Core
C4B63_11g116	-2.11794001429593	2.23849514993738e-08	Disruptive
C4B63_109g88	2.40396477961508	2.28489189683583e-08	Disruptive
C4B63_33g272	-2.08898106967631	2.37097065993265e-08	Disruptive
C4B63_101g65	-2.61554432600522	2.38559989618464e-08	Disruptive
C4B63_29g290	-2.63636697071224	2.42883620748267e-08	Disruptive
C4B63_36g170	-1.07191012971527	2.44228397479431e-08	Disruptive
C4B63_165g11	-1.71414686773055	2.44439152175136e-08	Disruptive
C4B63_64g57	-2.49486203164509	2.45077023637838e-08	Disruptive
C4B63_123g77	-4.21397722092123	2.51193381356116e-08	Disruptive
C4B63_1g43	-1.65222022849989	2.5162293910002e-08	Core
C4B63_18g171	1.05773541449041	2.54344349140743e-08	Core
C4B63_1g341	-1.63916041042158	2.61101443742746e-08	Disruptive
C4B63_33g47	-2.12987546037671	2.61692890487672e-08	Disruptive
C4B63_23g1324c	1.53877903879279	2.63145316724001e-08	Core
C4B63_6g155	-1.55689829858415	2.64930503557798e-08	Core
C4B63_45g99	1.18606137407743	2.71415640105436e-08	Core
C4B63_3g1132	-2.06831460670132	2.7217188056242e-08	Disruptive
C4B63_84g22	1.19545760909157	2.73406409933083e-08	Core
C4B63_6g265	1.34397187465948	2.73604462724053e-08	Core
C4B63_6g148	1.46551629989815	2.75518597844441e-08	Core
C4B63_522nc5	-2.14397951424491	2.7647381500278e-08	Core
C4B63_34g329	2.01368682567187	2.81713611557368e-08	Disruptive
C4B63_5g776	1.05455441132373	2.96260706138154e-08	Core
C4B63_29g300	-2.62900468914798	2.98256357171817e-08	Disruptive
C4B63_6g220	1.07457061503005	2.98393188849627e-08	Core
C4B63_104g100	2.40447831415213	3.10391111377456e-08	Core
C4B63_27g300	-1.96380620821992	3.44652733485838e-08	Disruptive
C4B63_11g343	-1.67619026431121	3.49742217991882e-08	Disruptive
C4B63_20g343	1.68770730148863	3.55384841414105e-08	Core
C4B63_37g97	-2.79156224196847	3.59045329972901e-08	Disruptive
C4B63_36g184	-2.24146792083603	3.59855312128906e-08	Disruptive
C4B63_36g369	-1.77521895759768	3.76246926209383e-08	Disruptive
C4B63_1g246	-2.11356296092068	3.80943495920887e-08	Disruptive
C4B63_65g69	-3.4820382564687	3.81755826893255e-08	Disruptive
C4B63_403g6	1.04432298894026	3.83698685881202e-08	Core
C4B63_15g268	-1.88939308277492	3.94264499938063e-08	Disruptive
C4B63_193g14	1.22245734739392	3.99779189788681e-08	Core
C4B63_42g67	1.3456190669442	4.02407599856282e-08	Core
C4B63_97g41	1.45845546418669	4.1794821523103e-08	Core
C4B63_164g3	-1.71902145762229	4.23702676521545e-08	Disruptive
C4B63_206g14	1.27169611151467	4.28973236479385e-08	Core
C4B63_122g39	-4.12678625294086	4.34922752570217e-08	Disruptive
C4B63_79g97	1.61509102373307	4.46747958079771e-08	Core
C4B63_253g14	1.02864598480149	4.58996267314774e-08	Core
C4B63_80g54	1.1827945017683	4.6590538686671e-08	Core
C4B63_12g6	1.24943879118412	4.7519240828815e-08	Core
C4B63_5g372	-2.18889992322402	4.76584642970767e-08	Disruptive
C4B63_26g55	-1.74673837533586	4.793901822729e-08	Disruptive
C4B63_50g188	-2.59436734322594	4.8170020602777e-08	Disruptive
C4B63_24g344	-1.37152269287134	4.87518876214615e-08	Disruptive
C4B63_39g106	-2.50473507023704	4.87518876214615e-08	Disruptive
C4B63_27g253	-1.82011763403354	4.93996438858209e-08	Disruptive
C4B63_15g503	-2.50474018857588	5.01780742534843e-08	Disruptive
C4B63_66g167	-2.49531970695678	5.09691759950334e-08	Disruptive
C4B63_31g50	1.0968194184771	5.18811909379472e-08	Core

C4B63_62g23	-2.45104660468711	5.2586599477782e-08	Disruptive
C4B63_121g1	-2.59874729078602	5.42321340962975e-08	Disruptive
C4B63_29g139	-3.00830547038099	5.53138719764248e-08	Disruptive
C4B63_44g123	1.07899670171878	5.59854279021969e-08	Core
C4B63_151g38	1.86202526860235	5.60685799370951e-08	Disruptive
C4B63_228g39	-1.59161787515409	5.63117946822722e-08	Core
C4B63_18g283	1.45603379868098	5.69946525006793e-08	Core
C4B63_44g181	-1.45860333341631	5.70270947430085e-08	Core
C4B63_11g336	-2.09753611028424	5.73056265759468e-08	Disruptive
C4B63_3g814	-2.48659221219838	5.98737096942367e-08	Disruptive
C4B63_238g16	1.59210504963023	6.07534119358424e-08	Core
C4B63_39g228	-1.91173688799442	6.28761339309176e-08	Disruptive
C4B63_12g163	1.5136847038348	6.50230304637498e-08	Core
C4B63_3g391	-2.01897808284513	6.51453243422119e-08	Disruptive
C4B63_1g779	-2.16427453562581	6.58472571503368e-08	Disruptive
C4B63_45g248	-1.43625477365456	6.5977314063629e-08	Core
C4B63_3g888	-1.56074575268914	6.68873208245761e-08	Disruptive
C4B63_31g264	1.41398083524865	6.87978988951943e-08	Core
C4B63_54g23	1.24587550679858	7.25384724079801e-08	Core
C4B63_66g71	-1.99204480254526	7.26204790657743e-08	Disruptive
C4B63_15g332	-2.13407006786755	7.26959713043554e-08	Disruptive
C4B63_90g60	-1.41002487106681	7.54535942841966e-08	Core
C4B63_26g311	1.8015076851475	7.64435077654422e-08	Core
C4B63_26g263	1.09040032517323	7.84332830480828e-08	Core
C4B63_277g3	1.67009231369051	8.03464214501403e-08	Core
C4B63_72g6	-1.2138877262239	8.06689544348025e-08	Disruptive
C4B63_30g162	2.46869550509788	8.1963635419996e-08	Core
C4B63_1g351	-1.95409935960635	8.32345674740065e-08	Disruptive
C4B63_1g661	-2.38186022460101	8.35783771529971e-08	Disruptive
C4B63_6g611	-1.79029516771633	8.57630212693753e-08	Disruptive
C4B63_70g127	-1.45364424483093	8.60036561713466e-08	Core
C4B63_10g455	1.20095554991227	8.60561943521423e-08	Core
C4B63_109g58	-2.11867172857708	8.67314401172044e-08	Disruptive
C4B63_71g117	-2.45003505704119	8.68830094082945e-08	Disruptive
C4B63_242g10	1.58628149985201	8.69543569386483e-08	Core
C4B63_235g10	-1.12010512335247	8.75980806054423e-08	Core
C4B63_134g59	-1.30591186913053	8.95226139312554e-08	Disruptive
C4B63_46g107	1.37994278021741	8.99045230399478e-08	Core
C4B63_28g106	-1.80275404863714	9.06137838713364e-08	Core
C4B63_13g1339c	1.76355743223022	9.23615133043103e-08	Core
C4B63_3g819	1.02763214966227	9.51381933781302e-08	Core
C4B63_36g55	-2.47732776543741	9.53162072926332e-08	Disruptive
C4B63_143g20	-1.2224351934717	9.54958067249313e-08	Core
C4B63_60g160	1.4252162960807	9.56891454990205e-08	Core
C4B63_422g11	1.96156611104107	9.57604544673239e-08	Core
C4B63_106g1	2.30510206382478	9.6211936210973e-08	Core
C4B63_3g447	-2.9201965288213	9.72909885089003e-08	Disruptive
C4B63_26g272	1.39690292058007	9.81588132255804e-08	Core
C4B63_183g32	2.31031471834795	9.90115032517277e-08	Core
C4B63_53g60	-2.73166735820065	1.00164917525977e-07	Disruptive
C4B63_2g449	-1.01628444249729	1.00502887243201e-07	Core
C4B63_50g238	-2.62533977339015	1.01422889508212e-07	Disruptive
C4B63_25g968c	1.17465866334039	1.02281336025046e-07	Core
C4B63_20g314	1.24182157048347	1.05166488706929e-07	Core
C4B63_50g101	-2.27096000423554	1.07244401439281e-07	Disruptive
C4B63_36g177	-2.40363718557071	1.13539805672648e-07	Disruptive
C4B63_20g344	-1.5546503605489	1.16886512948457e-07	Core
C4B63_13g978c	1.01157009214155	1.17985835801377e-07	Core
C4B63_7g410	1.42762279884132	1.18066500177987e-07	Core
C4B63_88g97	-1.99370069231399	1.18549203582663e-07	Disruptive
C4B63_62g159	-2.07815913127874	1.18739962859241e-07	Disruptive
C4B63_31g90	1.2606434382025	1.22214608141887e-07	Core
C4B63_144g17	-1.64735361067977	1.24169922893165e-07	Disruptive
C4B63_277g11	1.55430197131844	1.25066161518816e-07	Core

C4B63_326g24	-1.7400303435867	1.26195866924633e-07	Core
C4B63_96g41	-2.61778257064242	1.26825708507789e-07	Disruptive
C4B63_1g544	-1.91445753602216	1.28275144358475e-07	Disruptive
C4B63_51g226	-2.18807329976387	1.28449288212583e-07	Disruptive
C4B63_17g324	-1.85803809175576	1.29607429553906e-07	Core
C4B63_1g1237	-2.66086037844842	1.30439416671179e-07	Disruptive
C4B63_62g102	-1.81726163403646	1.32295263318518e-07	Disruptive
C4B63_1g691	-1.09311910741303	1.33977430498032e-07	Disruptive
C4B63_39g160	-2.3258559635986	1.36590315345851e-07	Disruptive
C4B63_15g485	-3.30416453148413	1.36650284013748e-07	Disruptive
C4B63_3g858	-2.83060620577846	1.38175702245671e-07	Disruptive
C4B63_100g2	1.29888669011356	1.42672926288994e-07	Core
C4B63_59g52	-3.45277433089841	1.42682749060782e-07	Disruptive
C4B63_35g109	1.29818118626437	1.43397099651595e-07	Core
C4B63_1g388	-2.16574744354682	1.47880223108357e-07	Disruptive
C4B63_5g780	-1.24231774046759	1.5357457294402e-07	Disruptive
C4B63_27g54	-2.54159704423948	1.55853662728771e-07	Disruptive
C4B63_275g9	-1.27704348056295	1.59005345642063e-07	Core
C4B63_1g1094	-3.16578694426902	1.60103645709267e-07	Disruptive
C4B63_18g176	1.09529720663922	1.60648873185755e-07	Core
C4B63_8g464	-1.08042661194993	1.65127585806906e-07	Core
C4B63_54g87	-2.8355790436538	1.67195262879713e-07	Disruptive
C4B63_26g42	-2.85057308345752	1.67580691786307e-07	Disruptive
C4B63_336g2	2.60496862462643	1.68125636207783e-07	Core
C4B63_1g756	-1.44056103770304	1.70431376240143e-07	Core
C4B63_306g9	2.64376986003636	1.7185522591908e-07	Disruptive
C4B63_600g1	-1.14751144104455	1.73874959046477e-07	Core
C4B63_24g215	1.22491563626081	1.75259827576986e-07	Core
C4B63_32g1	-1.07907962016627	1.79027888325681e-07	Disruptive
C4B63_28g292c	1.32171365703287	1.79119411741994e-07	Core
C4B63_161g33	1.34348066265233	1.79226793005242e-07	Core
C4B63_29g287	-2.83279594914337	1.81850169721232e-07	Disruptive
C4B63_16g317	1.69213491512073	1.85931203503782e-07	Core
C4B63_108g19	-1.42789311364427	1.87278250138672e-07	Disruptive
C4B63_128g24	1.1939777306832	1.88543332701135e-07	Core
C4B63_5g29	-1.36506768666569	1.91226428532535e-07	Core
C4B63_150g18	-2.0797504371788	1.91346258388098e-07	Disruptive
C4B63_47g72	-1.50813823332305	1.95207309662536e-07	Core
C4B63_30g255	-2.618420837662	1.97611799323821e-07	Core
C4B63_36g114	-2.19498956871275	1.98091560218138e-07	Disruptive
C4B63_118g24	-2.28230811082815	1.98164438488681e-07	Disruptive
C4B63_145g28	1.40038847448118	2.00211803807588e-07	Core
C4B63_1g1146	-2.35205614128039	2.03150443704272e-07	Disruptive
C4B63_80g21	1.04064825408046	2.05400974067492e-07	Core
C4B63_19g227	1.21041150711629	2.11484785643195e-07	Core
C4B63_71g178	-2.48662627741426	2.13332023946785e-07	Disruptive
C4B63_78g5	-26.5203522519945	2.17861712604861e-07	Core
C4B63_5g495	-2.42357253321886	2.20562378623324e-07	Disruptive
C4B63_183g20	1.77082704298109	2.2231025570315e-07	Core
C4B63_26g1224c	-2.10172700665202	2.28017038081294e-07	Core
C4B63_28g56	1.14374506593665	2.34278137761041e-07	Core
C4B63_7g103	1.17763082955619	2.34722032295042e-07	Core
C4B63_74g14	-2.30146669039654	2.34722032295042e-07	Disruptive
C4B63_156g4	-2.0362379740306	2.37641958014914e-07	Disruptive
C4B63_33g38	-2.52962262594276	2.42873069521147e-07	Disruptive
C4B63_32g1409c	-1.11672390544935	2.4360633799686e-07	Core
C4B63_69g34	-2.15813639341492	2.44389931358893e-07	Disruptive
C4B63_112g34	-3.83754186267324	2.46787903268529e-07	Core
C4B63_1g702	-1.47558434638836	2.54826524743896e-07	Disruptive
C4B63_19g229	1.36012864885405	2.56237268046686e-07	Core
C4B63_41g203	1.65506418100003	2.56237268046686e-07	Core
C4B63_51g842c	-1.06041456879417	2.56237268046686e-07	Core
C4B63_91g36	1.05425089455319	2.65905013693534e-07	Core
C4B63_99g112	1.54005170819768	2.71954450121256e-07	Core

C4B63_39g104	-2.20706693144396	2.76105282677955e-07	Disruptive
C4B63_29g280	-2.12689582539153	2.81225260321122e-07	Disruptive
C4B63_71g126	-2.81526325805485	2.84207895960108e-07	Disruptive
C4B63_15g313	-2.94535109265735	2.84495794585312e-07	Disruptive
C4B63_85g54	1.28263385792784	2.86289507723796e-07	Core
C4B63_19g222	1.76930027309621	2.885116708199e-07	Core
C4B63_59g231	-2.69516362173983	2.885116708199e-07	Disruptive
C4B63_11g375	-2.20893008686434	2.90918314692085e-07	Disruptive
C4B63_7g343	-1.29988637354509	2.92979521117279e-07	Core
C4B63_23g6	-1.72783471772032	2.97225442287905e-07	Core
C4B63_3g790	-2.13258880721425	3.01611173861577e-07	Disruptive
C4B63_5g642	-3.08644747210114	3.06465744561743e-07	Disruptive
C4B63_1g849	-2.82196635382572	3.10931222100967e-07	Disruptive
C4B63_3g833	-2.54828497932845	3.14016962615334e-07	Disruptive
C4B63_301g4	2.207838636289	3.23598262932145e-07	Core
C4B63_1g564	-2.00566616125506	3.35604579978874e-07	Disruptive
C4B63_18g331	1.63008694305745	3.51612428640238e-07	Core
C4B63_402g15	-2.23789824928572	3.52500730563132e-07	Core
C4B63_9g209	1.19885858312626	3.66456088255117e-07	Core
C4B63_387g16	-1.09539749636958	3.69647580869607e-07	Disruptive
C4B63_33g146	1.29585881961482	3.70146649005628e-07	Core
C4B63_151g43	-1.89907174229033	3.72172090707711e-07	Disruptive
C4B63_25g236	1.14498858169958	3.77344017355854e-07	Core
C4B63_20g339	-1.56787045113885	3.80886420450402e-07	Core
C4B63_14g231	-1.49920885743614	3.8771772723709e-07	Disruptive
C4B63_11g232	-2.0203752690681	3.92290394119839e-07	Disruptive
C4B63_39g69	-1.78104016817945	3.93426543965035e-07	Disruptive
C4B63_11g376	-1.73168634599171	3.95605547219957e-07	Disruptive
C4B63_26g267	1.22076499107202	4.06000445961673e-07	Core
C4B63_148g22	-1.07353501801831	4.22644983981367e-07	Core
C4B63_65g68	-4.4903118723447	4.33148792869096e-07	Disruptive
C4B63_69g40	-1.97300916848646	4.35297516553727e-07	Disruptive
C4B63_240g12	-1.32205400305422	4.40989907314375e-07	Disruptive
C4B63_67g75	-2.6803415431337	4.44293302543906e-07	Disruptive
C4B63_208g12	-1.70123082047015	4.67608420911367e-07	Core
C4B63_37g216	-6.40208990695446	4.72343306504575e-07	Disruptive
C4B63_5g373	-2.66074832501884	4.74359035877085e-07	Disruptive
C4B63_322g9	-1.03396375936754	4.74794498873499e-07	Core
C4B63_107g28	-2.75079389076815	4.75701876698526e-07	Disruptive
C4B63_36g111	-2.2462468333708	4.85868630661495e-07	Disruptive
C4B63_67g78	-2.8564498055624	4.94227875412625e-07	Disruptive
C4B63_157g36	1.10543717887924	5.00739729755845e-07	Disruptive
C4B63_65g14	-2.18881484667113	5.00954137091036e-07	Core
C4B63_2g26	1.0649565402392	5.06774776651927e-07	Core
C4B63_24g1258c	1.62021285816407	5.22737967334351e-07	Core
C4B63_85g60	1.17641427708964	5.24143985564252e-07	Core
C4B63_54g179	1.56742997570199	5.25992426694176e-07	Core
C4B63_40g98	1.06915332348894	5.26983550216304e-07	Core
C4B63_14g5	-1.30599883288641	5.5824123540211e-07	Core
C4B63_47g102	1.56345953844947	5.58535670419995e-07	Core
C4B63_9g333	1.34805215563274	5.60399865396887e-07	Core
C4B63_13g16	9.46481689967559	5.61963642289651e-07	Disruptive
C4B63_15g26	-2.33471406499696	5.89871781280154e-07	Disruptive
C4B63_30g254	-2.60855168816539	5.91538823005833e-07	Core
C4B63_36g66	-1.91561556079619	5.93854961186281e-07	Disruptive
C4B63_141g33	-2.21586141941813	5.94544514375554e-07	Disruptive
C4B63_2g648	1.08079656819527	5.94637394231433e-07	Core
C4B63_1g556	-2.00996334610894	5.9685660397903e-07	Disruptive
C4B63_3g815	-2.38625402437129	6.11658391380939e-07	Disruptive
C4B63_15g255	-2.01245663653744	6.28786252770697e-07	Disruptive
C4B63_67g24	-2.34995979722724	6.44037088989713e-07	Disruptive
C4B63_1g1346	-2.3240826599382	6.56575655720784e-07	Disruptive
C4B63_3g937	-1.9912938888277	6.58173085176035e-07	Disruptive
C4B63_5g72	-1.85566884782393	6.58392228512221e-07	Disruptive

C4B63_392g24	2.02053648205791	6.59285489711556e-07	Core
C4B63_28g334	-2.60109403801515	6.66476716396998e-07	Core
C4B63_240g13	-1.6555459390205	6.68663258758622e-07	Disruptive
C4B63_74g16	-3.8027508516922	6.69826401939309e-07	Disruptive
C4B63_54g96c	1.56120784847535	6.8452288483063e-07	Core
C4B63_9g476	-2.80755180355309	6.86361334504268e-07	Disruptive
C4B63_156g6	-3.79164794708483	6.87513091441043e-07	Disruptive
C4B63_528g4	-8.33009920276578	6.8826012840362e-07	Core
C4B63_6g285	-1.32835341836858	6.90818590980695e-07	Core
C4B63_121g88	-2.29907941409444	7.09923011960041e-07	Disruptive
C4B63_19g228	1.17290261062836	7.19395064994041e-07	Core
C4B63_71g158	-2.48333686663439	7.20377642035521e-07	Disruptive
C4B63_74g137	-1.76742460611826	7.22400569888341e-07	Disruptive
C4B63_5g631	-2.69970173913915	7.42390398120001e-07	Disruptive
C4B63_212g2	1.23748274688877	7.70493062930545e-07	Core
C4B63_258g17	-1.47515984505459	7.72841985103511e-07	Core
C4B63_43g74	1.04404037138322	7.75706894462676e-07	Core
C4B63_39g180	-2.42334363003128	7.76678186690065e-07	Disruptive
C4B63_7g145	1.37013656015413	7.80918613927307e-07	Core
C4B63_67g68	-3.98855055221241	7.82693504213538e-07	Disruptive
C4B63_15g122	-2.20221189953524	8.01132595975075e-07	Disruptive
C4B63_5g305	-2.05428195573861	8.03424746975404e-07	Core
C4B63_39g138	-2.0550033500407	8.08625260710435e-07	Core
C4B63_35g153	-2.0687152050084	8.4822305728569e-07	Disruptive
C4B63_13g205	1.06644509899123	8.59914293021509e-07	Core
C4B63_215g13	-2.86795822260548	8.66857057861462e-07	Disruptive
C4B63_29g286	-2.67955471133318	8.8807734010227e-07	Disruptive
C4B63_95g2	-2.62106823968785	8.93006046747361e-07	Disruptive
C4B63_67g210	-2.2612235277549	8.94741850438217e-07	Disruptive
C4B63_125g57	-2.1986177439399	9.10628920197361e-07	Disruptive
C4B63_402g10	1.09748894163719	9.16854487877432e-07	Core
C4B63_50g170	-1.94697121346206	9.31208812646543e-07	Disruptive
C4B63_1g1285	-2.17316701724453	9.46308667026138e-07	Disruptive
C4B63_7g154	-1.24456655956776	9.47588862853836e-07	Core
C4B63_64g68	-1.35890919863777	9.5639107158009e-07	Disruptive
C4B63_20g747c	1.69335716441168	9.57221604920327e-07	Core
C4B63_71g130	-2.2049228129705	9.60211570924261e-07	Disruptive
C4B63_3g843	-2.09700737847873	9.61827593351306e-07	Disruptive
C4B63_44g214	-1.3796645364688	9.64292146290652e-07	Core
C4B63_106g50	-2.13439863151728	9.90239570417374e-07	Disruptive
C4B63_60g77	1.62722135551061	1.02367886421343e-06	Core
C4B63_50g142	-2.23156121587568	1.03104858642507e-06	Disruptive
C4B63_294g10	4.07834644195512	1.08303602937293e-06	Core
C4B63_202g13	1.43021632457588	1.0943391812418e-06	Core
C4B63_27g269	-2.16140520628004	1.12062315802059e-06	Disruptive
C4B63_3g956	-2.00671420242876	1.13506511135419e-06	Disruptive
C4B63_3g863	-2.42692454392996	1.146427523562e-06	Disruptive
C4B63_205g30	-1.40222040130284	1.16105558204843e-06	Core
C4B63_14g60	1.2224667916943	1.16990008505868e-06	Core
C4B63_1g848	-2.02755709349533	1.19450195091153e-06	Disruptive
C4B63_48g25	-1.05240656533173	1.19896831050171e-06	Core
C4B63_169g40	-1.69590155981314	1.2100149391453e-06	Disruptive
C4B63_165g10	-1.75951294443833	1.23466173534125e-06	Disruptive
C4B63_35g123	1.38342478581285	1.2408576496574e-06	Core
C4B63_15g34	-1.94905865365458	1.24615513199331e-06	Disruptive
C4B63_12g182	1.19765914873041	1.26401488197888e-06	Core
C4B63_41g164	-1.78421175799702	1.28363699622426e-06	Core
C4B63_58g37	1.17830825484571	1.28547329213723e-06	Core
C4B63_13g12	-1.62104208725038	1.28592650235301e-06	Disruptive
C4B63_5g43	-1.63957578002767	1.29842647889598e-06	Disruptive
C4B63_96g40	-2.48317569555402	1.29965857891155e-06	Disruptive
C4B63_49g112	1.19849686905456	1.33991260926056e-06	Core
C4B63_39g231	-2.37104438583665	1.35172651183408e-06	Disruptive
C4B63_326g20	-2.45712349799234	1.39843650330115e-06	Core

C4B63_65g44	-4.78753108913101	1.45823138344618e-06	Disruptive
C4B63_38g147	1.36446660730343	1.47026769337259e-06	Core
C4B63_87g112	-1.78177269273537	1.49485845224314e-06	Core
C4B63_40g173	9.01805818149184	1.4974624250551e-06	Disruptive
C4B63_117g53	1.28096704444775	1.50332933644868e-06	Core
C4B63_11g251	-2.05900173968445	1.50565356524333e-06	Disruptive
C4B63_157g28	-1.09838644641696	1.5854888060285e-06	Core
C4B63_156g3	-2.68998276301467	1.59075802621205e-06	Disruptive
C4B63_13g190	1.03695005599434	1.60673863353301e-06	Core
C4B63_1g555	-2.93752834087629	1.61516660196872e-06	Disruptive
C4B63_28g240	-1.39450405373753	1.62726968713085e-06	Core
C4B63_92g67	1.16545097269631	1.63498626635358e-06	Core
C4B63_115g73	-1.72871901590946	1.63498626635358e-06	Disruptive
C4B63_50g227	-2.03929702985669	1.65308868607795e-06	Disruptive
C4B63_74g138	-1.96087290777325	1.66622875743069e-06	Disruptive
C4B63_14g62	1.35800778368194	1.68148669374252e-06	Core
C4B63_33g164	2.0315071144203	1.69391163176983e-06	Disruptive
C4B63_188g42	-1.054381104187	1.69694136601586e-06	Core
C4B63_30g219	1.32471449383734	1.70003690879138e-06	Core
C4B63_9g516	-1.98376705694219	1.70208399978866e-06	Core
C4B63_45g175	3.4730883133303	1.73673605283471e-06	Core
C4B63_8g143	-1.2165454596191	1.74153010107502e-06	Disruptive
C4B63_121g24	-2.24609077889772	1.74550085462165e-06	Disruptive
C4B63_4g283	-8.58501731378195	1.74887793711047e-06	Core
C4B63_74g53	-2.84885881078988	1.79271960282223e-06	Disruptive
C4B63_44g151	-1.68254219369813	1.86197266305399e-06	Core
C4B63_17g109	-1.21656101734128	1.87707316191984e-06	Core
C4B63_3g755	-2.56020626483987	1.89546434014029e-06	Disruptive
C4B63_33g167	1.22507714792091	1.89584594101359e-06	Disruptive
C4B63_121g43	-2.61104201347332	1.90446563747676e-06	Disruptive
C4B63_3g298	1.52584472433033	1.9372773094771e-06	Disruptive
C4B63_1g264	-1.22803171891848	1.95756515756748e-06	Disruptive
C4B63_138g113c	1.11654165301712	1.98878677969081e-06	Core
C4B63_30g291	1.37073217826057	1.98992978059782e-06	Core
C4B63_79g20	1.31560477916451	2.03873856918588e-06	Core
C4B63_263g4	1.08812670730177	2.07509277064642e-06	Core
C4B63_50g210	-2.03906562991015	2.08424031731541e-06	Disruptive
C4B63_299g1	-1.60930393940255	2.08424031731541e-06	Disruptive
C4B63_516g1	-3.72379789271621	2.10139909388941e-06	Core
C4B63_339g2	1.28762659760862	2.11444514005485e-06	Core
C4B63_56g98	1.17535042619836	2.16372834329823e-06	Core
C4B63_86g75	1.05946946736857	2.16673751149869e-06	Core
C4B63_33g313	-2.25389843055752	2.19781421563552e-06	Disruptive
C4B63_107g77	-1.93162876353285	2.19781421563552e-06	Disruptive
C4B63_36g52	-1.82175710137592	2.21310369206707e-06	Disruptive
C4B63_4g347	1.18313797997191	2.2296941441726e-06	Core
C4B63_29g91	-1.98956271784711	2.26957011959133e-06	Disruptive
C4B63_99g85	-8.60451914021496	2.3157532108096e-06	Disruptive
C4B63_4g252	1.22609518390551	2.32483428230546e-06	Core
C4B63_260g27	-1.11410415797757	2.33036741014947e-06	Disruptive
C4B63_189g23	-1.37762212737169	2.34682952877206e-06	Disruptive
C4B63_5g44	-1.02808472328416	2.49961981453229e-06	Disruptive
C4B63_1g901	-2.33513169300983	2.51020959967729e-06	Disruptive
C4B63_28g407c	1.03876198226277	2.5366775761746e-06	Core
C4B63_2g773	1.64040983349686	2.56811299447248e-06	Core
C4B63_18g61	1.03134490440009	2.60665009600623e-06	Core
C4B63_202g28	1.55187101154978	2.63131017091062e-06	Core
C4B63_10g312	-1.08533260429344	2.67522644289604e-06	Core
C4B63_28g142	-1.10887567469054	2.68753442996983e-06	Core
C4B63_103g68	-2.10511814246374	2.73734386662316e-06	Disruptive
C4B63_11g20	1.18583586961205	2.76351869592381e-06	Core
C4B63_1g585	-1.89865879246917	2.81009949480717e-06	Disruptive
C4B63_21g281	1.18422100970339	2.87528753670345e-06	Core
C4B63_65g88	-3.43208783347407	2.89331805995552e-06	Disruptive

C4B63_369g4	-2.41803969839317	2.97289271559324e-06	Core
C4B63_36g30	-1.60525830231637	3.01583095845271e-06	Core
C4B63_42g164	2.58403079540817	3.02562613869837e-06	Core
C4B63_70g68	1.0076835492024	3.05984256522659e-06	Core
C4B63_25g315	-1.0237822309398	3.16284962226284e-06	Core
C4B63_208g39	-1.66379908127454	3.16284962226284e-06	Core
C4B63_10g535	-2.59700441083929	3.27852086815327e-06	Disruptive
C4B63_251g4	-1.12295591989368	3.27987764787297e-06	Core
C4B63_36g69	-3.36075625178004	3.32503849062214e-06	Disruptive
C4B63_272g24	1.07663697961641	3.33261404461456e-06	Core
C4B63_259g135c	1.43430516705726	3.35710734342621e-06	Core
C4B63_5g538	-2.0804588082036	3.3616611007677e-06	Disruptive
C4B63_23g195	1.19157755530257	3.37208621197535e-06	Core
C4B63_3g494	-2.3857860922736	3.37665515305049e-06	Disruptive
C4B63_1g508	-2.20752294439908	3.46210556423706e-06	Disruptive
C4B63_8g134	-1.87627869531051	3.49909657068595e-06	Disruptive
C4B63_59g208	-2.96863458053718	3.50820554781866e-06	Disruptive
C4B63_106g71	-2.19743037397987	3.51548123367698e-06	Disruptive
C4B63_39g238	-1.79348413502008	3.52614225388396e-06	Disruptive
C4B63_74g50	-8.46084312406718	3.53632100231999e-06	Disruptive
C4B63_22g142	-1.8136232086433	3.56666389978652e-06	Disruptive
C4B63_5g326	-1.97063465526175	3.58703976792079e-06	Disruptive
C4B63_5g28	-2.01546244168763	3.59049204521209e-06	Disruptive
C4B63_3g383	-1.67252267696832	3.70836681786255e-06	Disruptive
C4B63_1g514	-2.15777276542269	3.71028396822684e-06	Disruptive
C4B63_60g116	1.29363558641395	3.7777292695273e-06	Core
C4B63_103g44	1.26652400997548	3.87101438401038e-06	Core
C4B63_1g281	-1.61273048325804	3.9431143577951e-06	Disruptive
C4B63_8g181	-1.70206626839883	3.96228046494508e-06	Disruptive
C4B63_56g38	1.47455578024538	3.97789800554262e-06	Core
C4B63_85g48	1.14481999398917	4.09384282224461e-06	Core
C4B63_5g262	1.65332628016334	4.09880855773857e-06	Core
C4B63_72g4	-1.12334234180114	4.12171068300114e-06	Disruptive
C4B63_1g964	-2.23181761668239	4.18224168586616e-06	Disruptive
C4B63_128g40	1.01785355250523	4.20149534900264e-06	Core
C4B63_275g10	-1.25393497851007	4.2030893550004e-06	Core
C4B63_286g89c	1.02774369464642	4.20434627848325e-06	Core
C4B63_67g92	-2.39732290053164	4.2370151017495e-06	Disruptive
C4B63_50g199	1.47719323019097	4.47082219984053e-06	Core
C4B63_29g108	-2.17078847192344	4.49868105845024e-06	Disruptive
C4B63_16g136	2.31414633832122	4.63427249384196e-06	Core
C4B63_189g41	-1.62974042770577	4.64609332079875e-06	Disruptive
C4B63_1g1166	-2.13584813189766	4.65941846931824e-06	Disruptive
C4B63_33g316	-2.50473477409148	4.78284590490511e-06	Disruptive
C4B63_29g292	-1.23139276463471	4.78341932721347e-06	Disruptive
C4B63_178g44	1.17795931440974	4.78341932721347e-06	Core
C4B63_71g157	-2.44851236605111	4.87034303911774e-06	Disruptive
C4B63_1g1219	-1.75331780314355	4.97675451437451e-06	Disruptive
C4B63_3g999	-2.22307502852542	4.98895464124546e-06	Disruptive
C4B63_1g1090	-2.02192165745249	5.06924323974094e-06	Disruptive
C4B63_25g1482c	1.28088625461604	5.09617588723356e-06	Core
C4B63_228g47	1.85716342348011	5.09617588723356e-06	Core
C4B63_11g145	-2.01565095819473	5.15583633415327e-06	Disruptive
C4B63_32g32	1.23067327592751	5.20275717683295e-06	Core
C4B63_35g155	1.11087982861868	5.23025259484586e-06	Core
C4B63_34g1235c	4.06491656352768	5.25513582851064e-06	Disruptive
C4B63_111g16	-2.88945641040797	5.25797722534863e-06	Disruptive
C4B63_50g220	-1.88633023918528	5.45004094445501e-06	Disruptive
C4B63_27g204	1.22671084330357	5.53387219117784e-06	Core
C4B63_21g258	1.09401335286269	5.54129721437876e-06	Core
C4B63_15g385	1.10606559433874	5.65166187112526e-06	Core
C4B63_123g73	-5.07648372514305	5.86947904454831e-06	Disruptive
C4B63_1g199	-2.24666380193738	5.94343789948122e-06	Disruptive
C4B63_223g4	-2.44738669877556	5.94352547604084e-06	Disruptive

C4B63_1g591	-2.42538684137532	6.21150554913529e-06	Disruptive
C4B63_218g34	-8.36790541264954	6.2315049205776e-06	Core
C4B63_85g319c	1.33584944286125	6.27362648800215e-06	Core
C4B63_233g30	-1.14242057307699	6.36378734059727e-06	Core
C4B63_4g76	1.01238889352818	6.44186421848889e-06	Core
C4B63_1g1200	-2.72952119719191	6.74022667064073e-06	Disruptive
C4B63_82g113	-2.71077395513704	6.85607969324412e-06	Disruptive
C4B63_296g16	-2.45991979451277	6.86891509291156e-06	Disruptive
C4B63_25g269	-1.30093434409693	6.9021798646024e-06	Disruptive
C4B63_50g212	-2.17206505847546	6.94277975703044e-06	Core
C4B63_3g737	-3.13829576459355	7.01613533575666e-06	Disruptive
C4B63_29g85	-2.10539234323548	7.085759953132e-06	Disruptive
C4B63_57g108	-1.04191855238441	7.09471388131248e-06	Core
C4B63_2g412	1.51563345928272	7.24826813721799e-06	Core
C4B63_59g101	-2.49415012537265	7.43568826940208e-06	Disruptive
C4B63_83g92	1.35910797552892	7.48869842740603e-06	Core
C4B63_68g71	-1.28044990161962	7.50764442460128e-06	Core
C4B63_2g453	1.07648650159134	7.66815981706909e-06	Core
C4B63_42g214	-1.39866433935854	7.68012488876033e-06	Core
C4B63_21g182	-1.4784466541434	7.86297625174821e-06	Disruptive
C4B63_3g646	-1.65215868438648	7.97850556412077e-06	Disruptive
C4B63_60g80	1.244190647653	8.17582453234447e-06	Core
C4B63_1g1161	-1.86852850028924	8.48073048114678e-06	Disruptive
C4B63_22g143	-1.36851769846922	8.58904606375656e-06	Disruptive
C4B63_15g331	-1.99641379884637	8.73302672133336e-06	Disruptive
C4B63_30g265	-1.90549289145156	8.86724331239845e-06	Core
C4B63_461g12	-2.88107092042948	8.96264270860928e-06	Disruptive
C4B63_299g15	-2.04116869077544	9.33460252137152e-06	Disruptive
C4B63_30g150	1.0066718079035	9.336126073771e-06	Core
C4B63_5g374	-1.72031014275475	9.37822291981714e-06	Disruptive
C4B63_32g264	-1.25131733872524	9.37822291981714e-06	Core
C4B63_121g77	-1.52836036494017	9.39075114672367e-06	Disruptive
C4B63_241g12	1.50649188882078	9.39075114672367e-06	Core
C4B63_15g493	-2.56721831103204	9.85131337606652e-06	Disruptive
C4B63_232g12	22.9178109976085	9.89359456746691e-06	Disruptive
C4B63_12g158	1.76531051660775	1.00144214243992e-05	Core
C4B63_8g97	-1.23895162517242	1.0136621577314e-05	Disruptive
C4B63_16g295	-1.307293548671	1.02315516330637e-05	Core
C4B63_40g17	-1.17016465546472	1.02471124403294e-05	Core
C4B63_485g5	1.79743866908018	1.02528166825122e-05	Core
C4B63_81g70	7.984145545043	1.04745792621685e-05	Core
C4B63_27g331	-2.18408773895253	1.04854227978486e-05	Disruptive
C4B63_106g41	1.41947440471266	1.04945821299213e-05	Core
C4B63_67g177	-1.98379768493219	1.05778810566187e-05	Disruptive
C4B63_8g252	-2.47888451356784	1.06513348075455e-05	Disruptive
C4B63_35g135	-1.40710897072652	1.06580074675927e-05	Disruptive
C4B63_13g200	1.1143045057324	1.07138522232584e-05	Core
C4B63_103g48	1.82699871779787	1.07160772588769e-05	Disruptive
C4B63_32g186	-1.89733733420084	1.07346373606485e-05	Disruptive
C4B63_38g191	-1.0410521639304	1.07841123556099e-05	Disruptive
C4B63_89g66	1.01960319214845	1.10472211637923e-05	Core
C4B63_20g325	-1.38736129701974	1.10751110930461e-05	Core
C4B63_91g74	-1.9571941540399	1.10888630794888e-05	Disruptive
C4B63_92g16	-1.64200698307366	1.13774113181671e-05	Core
C4B63_276g4	-1.32996648990336	1.15677723762846e-05	Disruptive
C4B63_3g620	-2.20994976469558	1.17282027566743e-05	Disruptive
C4B63_27g33	-2.84542847076011	1.17593559727095e-05	Disruptive
C4B63_132g19	-1.93271948903564	1.17682998742158e-05	Disruptive
C4B63_336g5	2.02642344076551	1.18283097655873e-05	Core
C4B63_50g180	-1.84008058000768	1.18343928417295e-05	Disruptive
C4B63_12g243	1.09695950263173	1.190541025718e-05	Core
C4B63_1g715	1.07261660988858	1.20893075822652e-05	Core
C4B63_1g604	-2.37090985344705	1.22173249113296e-05	Disruptive
C4B63_39g146	-2.13144285609498	1.24067132294988e-05	Disruptive

C4B63_18g322	1.07569451673999	1.25449396407803e-05	Core
C4B63_29g107	-1.64167206173112	1.25449396407803e-05	Disruptive
C4B63_98g38	-1.30096065742518	1.29485271087645e-05	Disruptive
C4B63_189g34	1.31773252403137	1.31430890830463e-05	Core
C4B63_56g154	-2.02856245901148	1.32030237758428e-05	Disruptive
C4B63_5g96	-2.24172220074546	1.32413001761496e-05	Disruptive
C4B63_15g106	-2.59441654285243	1.32413001761496e-05	Disruptive
C4B63_289g2	-8.22750074104772	1.3279672055952e-05	Core
C4B63_46g185	-1.28933740425914	1.33076091223554e-05	Disruptive
C4B63_66g54	-2.0327842591956	1.33443761475478e-05	Disruptive
C4B63_25g238	1.22070379402235	1.37205005556547e-05	Core
C4B63_23g1229c	1.0892681045898	1.39011648872119e-05	Core
C4B63_11g425	-1.78861071861872	1.42030307143917e-05	Disruptive
C4B63_122g44	-2.64789075770424	1.42218633984508e-05	Disruptive
C4B63_86g90	-1.87676502627618	1.44421532595201e-05	Disruptive
C4B63_4g550	1.07467271746925	1.45513953860077e-05	Core
C4B63_134g39	-1.25903091816309	1.47048640578031e-05	Disruptive
C4B63_392g23	2.08067152303012	1.49878003911181e-05	Core
C4B63_12g34	-2.66980947626683	1.50904708578093e-05	Core
C4B63_113g65	1.50230823877856	1.51108503229554e-05	Core
C4B63_62g19	-2.74540448480628	1.51519794677866e-05	Disruptive
C4B63_121g82	-1.56733416353356	1.51519794677866e-05	Disruptive
C4B63_403g3	1.34188729903888	1.53140555944426e-05	Core
C4B63_536g5	-1.07106038484834	1.54271687636801e-05	Disruptive
C4B63_50g242	-2.15368898699636	1.56409679123559e-05	Disruptive
C4B63_39g277	-2.47959237065007	1.58665440179514e-05	Disruptive
C4B63_333g9	1.52137013672404	1.58911239373478e-05	Disruptive
C4B63_33g67	-1.63286981944235	1.59759237676481e-05	Disruptive
C4B63_3g1076	-2.6123744258743	1.64540169624637e-05	Disruptive
C4B63_34g243	3.57427603155099	1.66721018618834e-05	Disruptive
C4B63_165g17	1.40421567529796	1.67537778366632e-05	Core
C4B63_14g63	1.5657022607588	1.69204975389613e-05	Core
C4B63_122g41	-2.89232758454244	1.69924793456247e-05	Disruptive
C4B63_6g525	-1.28413935841775	1.70790527505658e-05	Core
C4B63_4g446	-1.43345016145155	1.716150523146e-05	Core
C4B63_20g635c	1.3816443495795	1.82275303970871e-05	Core
C4B63_3g618	-1.63584723999423	1.82782093521029e-05	Disruptive
C4B63_53g177	-1.89169618473384	1.83993364231812e-05	Disruptive
C4B63_292g19	-1.21655391593263	1.8446931289945e-05	Core
C4B63_64g78	-2.44438338379317	1.87649188859134e-05	Disruptive
C4B63_1g1096	-3.07774248042769	1.88777720248668e-05	Disruptive
C4B63_4g275	1.26008932217539	1.89772942892058e-05	Core
C4B63_1g1101	-1.99411890700604	1.90250825402193e-05	Disruptive
C4B63_88g29	-1.61863818452843	1.92618207361113e-05	Disruptive
C4B63_213g8	-2.61152563539955	2.02737008182552e-05	Disruptive
C4B63_12g45	-2.1727653310407	2.07464949617799e-05	Core
C4B63_96g27	-1.44893389059689	2.08709217957274e-05	Disruptive
C4B63_68g111	-1.47588783365346	2.1173777287854e-05	Disruptive
C4B63_15g400	-1.6637287107506	2.1306394426882e-05	Core
C4B63_59g105	-1.73680212943676	2.1638159415351e-05	Disruptive
C4B63_11g496	1.08235228469496	2.22541970806306e-05	Core
C4B63_1g1170	-2.61668292717193	2.27479374166345e-05	Disruptive
C4B63_89g69	1.33480098755226	2.3518787466528e-05	Core
C4B63_115g68	-1.0784714795564	2.35724068428733e-05	Disruptive
C4B63_109g65	-1.33543580178842	2.37078425941718e-05	Disruptive
C4B63_32g139	-1.64106522700012	2.40645065012369e-05	Disruptive
C4B63_66g52	-2.04810730714069	2.43588859674733e-05	Disruptive
C4B63_29g372	-1.3612260289415	2.43820293333103e-05	Disruptive
C4B63_2g41	1.40152971355326	2.45002176712217e-05	Core
C4B63_5g218	-3.37064601237536	2.49858376923489e-05	Disruptive
C4B63_16g7	-1.30470310468644	2.50636563467222e-05	Core
C4B63_44g152	-1.77627019157142	2.619937605588e-05	Core
C4B63_71g131	-2.45221663637055	2.64103584224809e-05	Disruptive
C4B63_87g82	-1.45863457443626	2.66377616236989e-05	Core

C4B63_67g106	-3.83066124231697	2.67823173625976e-05	Disruptive
C4B63_3g1003	-1.82357148051692	2.69698892223618e-05	Disruptive
C4B63_33g293	-1.24690487709283	2.70598365376432e-05	Disruptive
C4B63_1g794	-1.45256930847856	2.7073747856179e-05	Disruptive
C4B63_8g73	-1.62451014938275	2.75123164530799e-05	Disruptive
C4B63_37g197	-2.16931608753647	2.76589052155143e-05	Disruptive
C4B63_15g397	-1.79179940814963	2.78237304302492e-05	Disruptive
C4B63_153g21	-2.53206942033986	2.81519029383379e-05	Core
C4B63_67g188	-2.80474885684099	2.82112455843737e-05	Disruptive
C4B63_87g89	-1.26914678041815	2.85302398076808e-05	Core
C4B63_33g372	-1.6172944368033	2.86357813354462e-05	Disruptive
C4B63_121g45	-3.01327762579147	2.89017425656563e-05	Disruptive
C4B63_91g75	-1.8744157509984	2.89212823978469e-05	Disruptive
C4B63_71g133	-2.04075356022007	2.92378118835612e-05	Disruptive
C4B63_100g90	-1.95264650346277	2.94951957424166e-05	Disruptive
C4B63_104g94	-1.10753688733978	2.95972428718274e-05	Core
C4B63_330g10	1.34021275669679	2.98246679599967e-05	Core
C4B63_338g3	-2.78740008571161	3.04926360206668e-05	Disruptive
C4B63_19g821c	1.17259214340371	3.07403687359014e-05	Core
C4B63_112g71	2.13771723377926	3.07469068706796e-05	Core
C4B63_90g107	-2.56863338246406	3.09231289226942e-05	Disruptive
C4B63_1g550	-1.93755006771166	3.10501497087548e-05	Disruptive
C4B63_60g197	1.24063813633741	3.12720833740415e-05	Core
C4B63_1g1314	-2.23334296034069	3.18226517506964e-05	Disruptive
C4B63_308g5	-1.11635747701035	3.20232126537939e-05	Core
C4B63_64g125	-1.75643249402511	3.20310547832083e-05	Disruptive
C4B63_266g12	-2.00842854344381	3.20310547832083e-05	Core
C4B63_80g53	1.2674533017468	3.20585404434114e-05	Core
C4B63_300g102c	2.34289765816214	3.21646619979153e-05	Disruptive
C4B63_36g375	-3.04607310826667	3.22674077222014e-05	Disruptive
C4B63_251g11	1.67206059115596	3.22994846001156e-05	Core
C4B63_62g164	-3.30629250868605	3.24491271852905e-05	Disruptive
C4B63_5g590	-1.4837498890158	3.25891763282993e-05	Disruptive
C4B63_1g959	-2.31169414272724	3.31375234282619e-05	Disruptive
C4B63_19g230	1.06484519293362	3.41810189794669e-05	Core
C4B63_4g267	1.30888213370593	3.47017028040606e-05	Core
C4B63_80g67	1.12975971346481	3.493553892788e-05	Core
C4B63_8g197	1.06131196162939	3.50759713986229e-05	Core
C4B63_38g72	1.38783932078653	3.5412303946708e-05	Core
C4B63_53g70	-3.13135755813048	3.57098648500372e-05	Disruptive
C4B63_39g143	-2.03868927515916	3.58830780750596e-05	Disruptive
C4B63_314g18	-1.23257373874321	3.66750250799694e-05	Core
C4B63_26g280	1.02843854597764	3.68465477363625e-05	Core
C4B63_48g81c	1.10239983016374	3.68465477363625e-05	Core
C4B63_31g217	1.00421805684236	3.69327306515653e-05	Core
C4B63_9g335	-1.40603419559643	3.73125439964394e-05	Core
C4B63_104g91	-3.0742725623779	3.83292560594863e-05	Core
C4B63_137g17	-1.33796308710987	3.84412142990151e-05	Core
C4B63_48g693c	1.4391372050716	3.90921567818562e-05	Core
C4B63_124g46	-1.15375047966199	3.9474618578561e-05	Disruptive
C4B63_8g96	-1.44798636711399	3.97988447529044e-05	Disruptive
C4B63_15g396	-2.04453714852529	3.98252799814432e-05	Disruptive
C4B63_1g367	-1.73521933168618	3.99782995180127e-05	Disruptive
C4B63_115g12	-1.773045246232	3.99863865688843e-05	Disruptive
C4B63_53g57	-2.89712595702701	4.07453498018969e-05	Disruptive
C4B63_70g547c	1.00256419474474	4.15682343213191e-05	Core
C4B63_258g26	1.92460485465617	4.21071624431412e-05	Core
C4B63_5g74	-1.88615091743365	4.2620857283799e-05	Disruptive
C4B63_1g1253	-2.70001306986949	4.29781168476212e-05	Disruptive
C4B63_3g403	-1.32642648563261	4.35483590430881e-05	Disruptive
C4B63_5g226	-2.77599874898396	4.38133893189775e-05	Disruptive
C4B63_12g35	-1.20420206766722	4.39273695421643e-05	Disruptive
C4B63_29g288	-2.00253695348081	4.41153638929188e-05	Disruptive
C4B63_48g15	1.29766510402975	4.44245254696643e-05	Core

C4B63_149g61	1.13029816730853	4.47561975037929e-05	Core
C4B63_224g17	-1.46431712219788	4.49632555152959e-05	Core
C4B63_195g15	-1.57545309110239	4.50539043971296e-05	Disruptive
C4B63_281g7	-1.65469434342887	4.54173876154352e-05	Core
C4B63_5g134	-2.80584676362439	4.60058058612527e-05	Disruptive
C4B63_43g212	1.02066447653347	4.66406970143386e-05	Core
C4B63_33g282	-1.84382009718108	4.67286235628422e-05	Disruptive
C4B63_15g399	-2.07115980353449	4.71713265007321e-05	Disruptive
C4B63_173g38	-7.84496074395155	4.74850263742744e-05	Disruptive
C4B63_300g106c	1.9964184499612	4.7706806804415e-05	Disruptive
C4B63_65g41	-1.71527282149366	4.84096806962978e-05	Disruptive
C4B63_136g6	-1.84094817332824	4.84758197864434e-05	Disruptive
C4B63_265g1	-2.11955778085417	4.90172597020399e-05	Core
C4B63_75g100	1.04500159744248	4.92232985217558e-05	Core
C4B63_27g124	1.07088918711708	4.94653580690613e-05	Disruptive
C4B63_544g5	1.03578420069539	4.96107144286299e-05	Core
C4B63_66g17	-1.93537584068532	4.9643150681195e-05	Disruptive
C4B63_73g22	-1.11178821120773	5.04039821023679e-05	Core
C4B63_15g218	-1.52644623786268	5.10895115854384e-05	Disruptive
C4B63_19g191	1.01063069603768	5.16853191453928e-05	Core
C4B63_36g21	-1.78849657471514	5.19881023636897e-05	Disruptive
C4B63_8g243	-2.79844724752109	5.27142273040402e-05	Disruptive
C4B63_111g8	-2.20071646738909	5.41891914419006e-05	Disruptive
C4B63_39g109	-2.18069658712826	5.42079564249532e-05	Disruptive
C4B63_66g67	-1.98500813027129	5.43510077603389e-05	Disruptive
C4B63_13g331	-1.02141358303245	5.44632008707967e-05	Core
C4B63_95g69	-1.2335781765988	5.48034339798751e-05	Disruptive
C4B63_36g121	-3.72534774767103	5.48325371250482e-05	Disruptive
C4B63_60g151	1.24710190321931	5.53243892920642e-05	Core
C4B63_33g269	-2.12174543782581	5.61531129527651e-05	Disruptive
C4B63_8g106	-1.83406702312113	5.62484409747262e-05	Disruptive
C4B63_15g321	-2.14920394649531	5.97650878486403e-05	Disruptive
C4B63_36g56	-3.00776538094931	6.00063403945452e-05	Disruptive
C4B63_35g179	1.14515360257498	6.00410894135703e-05	Core
C4B63_3g1059	-1.49595283068367	6.04325407343144e-05	Disruptive
C4B63_71g164	-3.0800656649821	6.11403456446482e-05	Disruptive
C4B63_121g70	-1.86687449432982	6.12486715729952e-05	Disruptive
C4B63_67g209	-2.0385606048305	6.37155994500596e-05	Disruptive
C4B63_6g267	1.29439487882379	6.42564496228593e-05	Core
C4B63_29g174	1.12953364564732	6.57146717133686e-05	Core
C4B63_37g406	-1.19073915368229	6.60303758597703e-05	Core
C4B63_206g7	1.28062919289022	6.77843223245547e-05	Core
C4B63_6g2556c	1.035994297154	6.80467937605075e-05	Core
C4B63_17g97	1.04338436961207	6.84510152081431e-05	Core
C4B63_67g183	-2.35128268562684	6.8474215663571e-05	Disruptive
C4B63_36g68	-2.34259397434788	6.9091319420229e-05	Disruptive
C4B63_53g23	-3.36642022573768	6.92957697418648e-05	Core
C4B63_65g75	-1.7700423936169	6.98316620577106e-05	Disruptive
C4B63_2g291	1.09084510254599	7.00569855235249e-05	Core
C4B63_8g108	-1.61108075383947	7.17378437766229e-05	Disruptive
C4B63_37g80	-1.8975710080167	7.208354762397e-05	Disruptive
C4B63_8g173	-1.34567556330558	7.24151752467997e-05	Disruptive
C4B63_90g101	-1.46994810454413	7.27210455812169e-05	Disruptive
C4B63_4g175	-1.23919361633455	7.40982242111503e-05	Core
C4B63_102g56	1.10643105627451	7.5927365633927e-05	Core
C4B63_11g155	-2.21413929725642	7.74215531198422e-05	Disruptive
C4B63_544g6	1.77506541289249	7.74599215501192e-05	Core
C4B63_247g17	1.12576471589582	7.77074110990221e-05	Core
C4B63_22g138	-1.17539081353889	7.8857113781374e-05	Disruptive
C4B63_33g37	-2.03684039266808	7.89024683221333e-05	Disruptive
C4B63_89g68	1.41359528102072	8.09452799812975e-05	Core
C4B63_132g20	-3.52794488473097	8.09452799812975e-05	Disruptive
C4B63_19g225	1.26410908509757	8.15526535058523e-05	Core
C4B63_251g7	-2.08949654790793	8.21207929317566e-05	Core

C4B63_5g297	-1.76555214864543	8.21363725301559e-05	Disruptive
C4B63_11g151	-1.40810921788552	8.64290977766311e-05	Disruptive
C4B63_1g532	-2.01978532357076	8.67756216755431e-05	Disruptive
C4B63_41g116	-2.03918369411825	8.90242452703225e-05	Core
C4B63_73g75	1.58308941247616	9.1949164365227e-05	Core
C4B63_101g66	-2.50779188530704	9.25100285194226e-05	Disruptive
C4B63_11g370	-2.20293740598175	9.56684062071068e-05	Disruptive
C4B63_14g81	-1.04895588830904	9.68356652685872e-05	Core
C4B63_89g20	1.43017798324272	9.68356652685872e-05	Disruptive
C4B63_128g56	-1.5059873284812	9.74912651137354e-05	Core
C4B63_125g66	-2.46756374560832	0.000100053484554539	Disruptive
C4B63_3g450	1.43061036513314	0.000100627741360059	Disruptive
C4B63_3g1118	1.03120689661788	0.000101003603153553	Core
C4B63_178g47	1.48326442511112	0.000102597057924618	Core
C4B63_1g400	-1.97830982734166	0.000104237378689631	Disruptive
C4B63_1g912	-1.92384854116253	0.000104358832165513	Disruptive
C4B63_196g32	-1.24931429911663	0.000107102554247587	Disruptive
C4B63_50g239	-2.05351866967827	0.00011000556972018	Disruptive
C4B63_88g95	-3.19992463307089	0.000112564031996667	Disruptive
C4B63_1g1307	-1.35984222852488	0.000114477514461463	Disruptive
C4B63_95g68	-1.23976995476849	0.000115991774754814	Disruptive
C4B63_15g337	-1.64357337028853	0.000116784944049368	Disruptive
C4B63_39g147	-1.74861681317964	0.000119760591967742	Disruptive
C4B63_2g181	1.26373466367983	0.000120048632133626	Core
C4B63_146g45	-2.17564140123414	0.000120652676449051	Disruptive
C4B63_36g93	-1.89844997893741	0.000123225791235666	Disruptive
C4B63_5g38	-2.51751314725529	0.00012471744748923	Disruptive
C4B63_116g2	1.47000507081701	0.00012516987225058	Core
C4B63_3g915	-1.65795688934163	0.000126143430352256	Disruptive
C4B63_74g54	-1.42613094561249	0.000126188147236485	Disruptive
C4B63_68g69	-1.27372786511969	0.000126493562257365	Core
C4B63_5g313	-1.74910462256691	0.000128528661715722	Disruptive
C4B63_183g37	1.53199077035932	0.000128903802001623	Core
C4B63_53g143	1.51242054705688	0.000130541167663395	Core
C4B63_115g23	-1.74026488707743	0.000130800203185776	Disruptive
C4B63_9g298	-1.06800327214371	0.00013087781883887	Core
C4B63_103g72	-2.014245154441	0.000132424255564407	Disruptive
C4B63_67g182	-4.4071693803239	0.000132584682124328	Disruptive
C4B63_53g213	-1.24266744453526	0.000132977440873981	Disruptive
C4B63_126g18	-1.96740706020328	0.000134462181733709	Disruptive
C4B63_32g187	-2.07692858257589	0.000135478449966102	Disruptive
C4B63_5g61	-2.55259894596681	0.000135655003461618	Disruptive
C4B63_2g298	-1.08935386366569	0.000136170827233597	Core
C4B63_12g291	1.51511403751824	0.00013657765575075	Core
C4B63_41g129	-1.069710997887	0.000141317804279314	Core
C4B63_5g149	-2.53291497966503	0.000141803153084565	Disruptive
C4B63_37g83	-2.34307447865207	0.000141803153084565	Disruptive
C4B63_66g94	-2.60160688509393	0.000144376510669641	Disruptive
C4B63_181g11	1.49279000796747	0.000144397875136504	Core
C4B63_131g57	-4.06422978217062	0.00014669581205804	Disruptive
C4B63_507g5	-3.24466426875247	0.000148332872318275	Disruptive
C4B63_1g986	-2.07819892749027	0.000150110671360992	Disruptive
C4B63_330g8	1.47465548432096	0.000150130625548024	Core
C4B63_74g101	-4.13137467053885	0.000155755590132346	Core
C4B63_1g946	1.78025672844012	0.000155837882133849	Core
C4B63_31g218	1.07727205330868	0.000156832906414645	Core
C4B63_72g105	1.05985055516183	0.000161075273579518	Core
C4B63_21g280	1.3298212326996	0.00016162669379951	Core
C4B63_95g64	-1.28592482856475	0.000162821400125188	Core
C4B63_39g276	-1.76037026847327	0.000165921143118779	Disruptive
C4B63_1g602	-2.07771646323842	0.000167090634683851	Disruptive
C4B63_48g29	1.4376144811657	0.000168451239684876	Core
C4B63_32g177	-1.77581504701478	0.000170208328668155	Disruptive
C4B63_151g47	1.00600249039051	0.000172550670211661	Disruptive

C4B63_84g174c	1.3186153537331	0.000177572361493765	Core
C4B63_104g96	-1.38112329754181	0.000179392607547362	Core
C4B63_29g184	1.07912336882402	0.000182427187190601	Core
C4B63_121g66	-1.91959121517373	0.00018432973183827	Disruptive
C4B63_3g733	-1.58632255549091	0.000184466845246572	Disruptive
C4B63_71g192	-1.46827099197171	0.000185561749082794	Disruptive
C4B63_39g275	-1.74621680130994	0.00019059714432729	Disruptive
C4B63_30g116	1.0272410422627	0.000192796121072038	Core
C4B63_9g93	-1.94623554667806	0.000193160916466773	Disruptive
C4B63_3g801	-2.72625227852992	0.000194061231131348	Disruptive
C4B63_3g991	1.2211585978969	0.000194133560063786	Core
C4B63_33g52	-1.9175089411858	0.000194818791826396	Disruptive
C4B63_67g14	-2.29260759769838	0.000195084587120756	Disruptive
C4B63_39g153	-2.0404257641627	0.000196270980292202	Disruptive
C4B63_104g103	1.11444764517135	0.000196520626846817	Core
C4B63_3g105	-1.74249945144342	0.000198095582032304	Disruptive
C4B63_60g146	1.07219059558828	0.000201967485103847	Core
C4B63_15g494	-1.57197406219678	0.000205408159573314	Disruptive
C4B63_174g7	-1.78887573863463	0.000206889194146951	Disruptive
C4B63_88g106	-1.52131619380581	0.000212371441486897	Disruptive
C4B63_60g132	1.12538674738896	0.000214259483246133	Core
C4B63_37g180	-2.62650152519104	0.000214775435142533	Disruptive
C4B63_157g168c	1.46570835709375	0.000214884362336347	Core
C4B63_36g116	-3.65743925770654	0.000215319432983048	Disruptive
C4B63_12g357	-1.69570056434774	0.000216898720758135	Disruptive
C4B63_202g24	2.24800138947111	0.000219362231942312	Core
C4B63_64g194	-1.65665557177354	0.000221429202820182	Disruptive
C4B63_31g241	-1.0104886048773	0.000221846295201899	Disruptive
C4B63_107g104	-1.96521582608292	0.000222104100236472	Disruptive
C4B63_15g492	-3.4374538788245	0.00022649129809633	Disruptive
C4B63_4g545	1.35598019313024	0.000228318845738328	Core
C4B63_50g93	-2.22201751647997	0.000228937020491621	Disruptive
C4B63_65g125	-2.20977304546152	0.000230883386351584	Disruptive
C4B63_3g923	-1.58715671573183	0.000239788017371587	Disruptive
C4B63_37g86	-2.02437785330516	0.000242890309407643	Disruptive
C4B63_2g643	-2.41328962565306	0.000243049252544179	Disruptive
C4B63_53g74	-4.69144352600109	0.000246993422984904	Disruptive
C4B63_5g380	-1.72062612009718	0.000250585013377931	Disruptive
C4B63_258g18	-2.65962306197354	0.000252297914817603	Core
C4B63_65g70	-2.57998527752581	0.000252423153702891	Disruptive
C4B63_293g6	-1.85506098319929	0.000257089499446049	Core
C4B63_28g343	-1.98491277520989	0.000261270515684352	Disruptive
C4B63_146g28	2.20465957889772	0.000263307957763778	Core
C4B63_2g280	-1.30523625487388	0.000267389912687466	Disruptive
C4B63_67g76	-2.16478286159064	0.000268923139237469	Disruptive
C4B63_90g3	-2.35217510187012	0.000274014439731066	Disruptive
C4B63_74g129	-1.82246412993681	0.00027602817046293	Disruptive
C4B63_37g206	-2.10041030356099	0.000280945567872348	Disruptive
C4B63_62g85	1.60570655967143	0.000281020946769995	Core
C4B63_41g121	-1.46924918761359	0.000282382008395536	Core
C4B63_1g1097	-1.56401502440719	0.000283017218218471	Disruptive
C4B63_29g37	-1.54233022018357	0.000295599872470178	Disruptive
C4B63_163g24	-1.52348863545858	0.000298413088742671	Core
C4B63_62g22	-1.99863823336331	0.00030229064830474	Disruptive
C4B63_44g150	-1.55727662036466	0.000306794952405199	Core
C4B63_51g82	1.11566655208471	0.000306843658632752	Core
C4B63_98g22	1.09129956554791	0.000312186702379511	Disruptive
C4B63_74g135	-1.74505810371058	0.000313982176778005	Disruptive
C4B63_1g786	-2.22929080193879	0.000317376569633645	Disruptive
C4B63_27g127	1.08340575462457	0.000321976768121412	Core
C4B63_37g179	-2.97390143614847	0.000327687911142342	Disruptive
C4B63_1g230	-2.05995551436441	0.000329232995653315	Disruptive
C4B63_34g136	-1.09783510086453	0.000331369197267441	Disruptive
C4B63_129g31	1.49089376227843	0.000336765299174428	Disruptive

C4B63_5g123	-1.61495529646789	0.000343721726176503	Disruptive
C4B63_100g63	1.06974241390409	0.000347394397166247	Core
C4B63_1g1030	1.211330678041	0.000348722993731675	Disruptive
C4B63_64g146	-2.09725711478815	0.000349166531164536	Disruptive
C4B63_13g367	-1.64967245433694	0.000352772477501162	Disruptive
C4B63_8g258	-3.36292861910042	0.000359823386891637	Disruptive
C4B63_135g34	-2.0145136799367	0.000365667709216465	Disruptive
C4B63_228g31	-1.49661507108829	0.000368108363083346	Core
C4B63_87g27	-1.10090680391537	0.000371453288759892	Core
C4B63_27g320	-2.38022431716739	0.000371865413523608	Disruptive
C4B63_136g12	-1.07024525769379	0.000373895792066795	Disruptive
C4B63_76g23	1.54385827149186	0.000384704108956186	Core
C4B63_71g187	-2.41799824942247	0.000387733742128033	Disruptive
C4B63_69g49	-1.03115689849032	0.000390974806358758	Disruptive
C4B63_1g533	-2.77887869211965	0.000394117006327575	Disruptive
C4B63_103g75	-1.86808139555545	0.000402138463119574	Disruptive
C4B63_193g18	1.16620703276248	0.000403819737343606	Core
C4B63_208g38	1.45232480583946	0.000405057657381484	Core
C4B63_29g371	-2.14349766449639	0.000417381221348134	Disruptive
C4B63_37g396	1.19559756929865	0.000418153876453049	Core
C4B63_53g75	-1.53085568797288	0.000418264575509522	Disruptive
C4B63_241g23	3.97138391542658	0.000418453242299728	Disruptive
C4B63_27g290	-1.77669895184398	0.000427784387957376	Disruptive
C4B63_103g83	-1.71492059971032	0.000434641831575157	Disruptive
C4B63_62g105	-1.48434404788918	0.00044378847125223	Disruptive
C4B63_13g366	-2.22381129322812	0.000454293463587781	Disruptive
C4B63_137g43	-1.14391077219702	0.000454603680225829	Core
C4B63_358g11	-1.55712196124668	0.000466801566754864	Core
C4B63_143g3	-1.05322786245095	0.000470178820775374	Core
C4B63_39g159	-1.77850420526206	0.000471210874093998	Disruptive
C4B63_278g8	1.05747682384594	0.000475984525745994	Core
C4B63_3g564	-1.53137313275685	0.00047642174314848	Disruptive
C4B63_8g167	-1.73963044204566	0.000499903351297712	Disruptive
C4B63_12g32	-1.13154432246969	0.000501121166265305	Disruptive
C4B63_29g339	1.19926317727458	0.000501544839751673	Core
C4B63_53g67	-1.38457318593355	0.000508153339358169	Disruptive
C4B63_193g20	1.23374370449638	0.000517118414414537	Core
C4B63_15g260	-1.68183945700591	0.000531547355360189	Disruptive
C4B63_235g2	2.16091676731369	0.000533627735043513	Core
C4B63_4g3081c	1.21266507837418	0.000536246563173443	Core
C4B63_53g178	-2.35938851906492	0.000545845218021609	Disruptive
C4B63_71g97	-1.39867908824484	0.000552507158404802	Disruptive
C4B63_29g428	-1.26976652152979	0.000561027871452026	Disruptive
C4B63_66g109	1.54386595143213	0.000580723044304276	Core
C4B63_49g58	-1.72809745077306	0.000584042194094275	Core
C4B63_39g175	-1.74390266993045	0.000592578598572421	Disruptive
C4B63_5g281	-1.96942279375801	0.000594880012124328	Disruptive
C4B63_108g21	-1.23501731685221	0.000595069445874229	Disruptive
C4B63_13g1646c	1.9272245072339	0.000600880870998458	Core
C4B63_93g44	1.00632086484903	0.00060226028760195	Core
C4B63_15g31	-1.92833468601559	0.000606022827904435	Disruptive
C4B63_59g230	-1.90826100545292	0.000623113453401079	Disruptive
C4B63_3g204	-1.89911580211451	0.000628692805596816	Disruptive
C4B63_71g177	-2.0037076059638	0.000633447141208358	Disruptive
C4B63_1g819	-1.12022087311272	0.000641113913625666	Disruptive
C4B63_183g27	2.54738204166478	0.00064862922561878	Core
C4B63_22g319	1.12732614905862	0.000658506379146903	Core
C4B63_17g327	1.28001219948512	0.00066277353899719	Core
C4B63_29g135	-1.35762767877346	0.000671919006289476	Disruptive
C4B63_60g87	-1.66918032990371	0.000673237651516431	Disruptive
C4B63_99g57	1.09026688968674	0.000677680687689556	Core
C4B63_33g27	-1.75288488733384	0.000683722487332835	Disruptive
C4B63_17g237	-1.07620180880817	0.000684533474275527	Core
C4B63_57g66	1.09561199022203	0.000694869241569185	Core

C4B63_37g395	1.25229411108499	0.000695240079557623	Core
C4B63_41g117	-1.88286973979414	0.000695695877316216	Core
C4B63_34g1428c	2.1827246427361	0.000699446939808531	Disruptive
C4B63_1g516	-3.33058221841205	0.000700168669764359	Disruptive
C4B63_61g21	1.120474493758	0.000702950091460058	Core
C4B63_39g61	-1.5994897203231	0.000705920581960983	Disruptive
C4B63_209g15	1.01267991283003	0.000707956959027742	Core
C4B63_162g35	-1.26065630102992	0.000708085699345132	Core
C4B63_67g23	-1.92101076101166	0.000708561683136089	Disruptive
C4B63_93g8	-3.84615148551832	0.000716590478091267	Disruptive
C4B63_63g4	1.5665001950074	0.000723588851556274	Core
C4B63_62g181	-2.63580065010231	0.000727342403288425	Disruptive
C4B63_103g69	-2.62819371833049	0.000747955781067933	Disruptive
C4B63_37g156	-1.45873415718996	0.000748896480935969	Disruptive
C4B63_17g134	-1.35576496973532	0.000750382523697698	Core
C4B63_3g899	-1.43616673971251	0.000761693308795496	Disruptive
C4B63_29g327	-1.43473502677731	0.000766093753455545	Disruptive
C4B63_258g25	1.5791020310018	0.000774687599212235	Core
C4B63_94g89	-1.60077676976442	0.000778080879122146	Disruptive
C4B63_158g47	-1.33656497604914	0.000779069724906307	Disruptive
C4B63_1g497	-2.38020710089589	0.00079037058169607	Disruptive
C4B63_66g156	-1.67666955025841	0.000801541222346209	Disruptive
C4B63_1g1283	-1.38828701783482	0.000813386387185893	Disruptive
C4B63_1g833	-1.69779734835202	0.000833667385268304	Disruptive
C4B63_20g353	-1.1242961251014	0.000834451106025582	Core
C4B63_258g22	-1.47677923112979	0.000840027224454294	Core
C4B63_169g33	-1.41396014159197	0.000847595710330833	Disruptive
C4B63_67g22	-2.16275546256863	0.000851664462174439	Disruptive
C4B63_55g201	-1.32297608199248	0.000858004972584777	Disruptive
C4B63_174g6	-1.7139978120763	0.000858004972584777	Disruptive
C4B63_13g133	-1.06894154493784	0.000866426056908339	Core
C4B63_44g243	1.21529407876466	0.000867626327987645	Core
C4B63_1g196	-1.36850571440568	0.000869937800844214	Disruptive
C4B63_125g62	-1.26623562682687	0.000875544004356651	Disruptive
C4B63_88g72	-2.1666098514358	0.000878290545620149	Disruptive
C4B63_29g60	-1.68105950219002	0.000893312450762368	Disruptive
C4B63_146g37	-2.46222841468836	0.000897327340613207	Disruptive
C4B63_1g594	-2.49348371856291	0.000900946512795964	Disruptive
C4B63_9g97	-1.88835448355562	0.000917432552585328	Disruptive
C4B63_41g274	-1.08144854509931	0.000959904934249868	Core
C4B63_20g161	1.00385010141714	0.000963371055306113	Core
C4B63_37g102	-1.81945984090042	0.000990000772246155	Disruptive
C4B63_33g368	-1.68838093490585	0.000990044826582955	Disruptive

GeneID	log2FoldChange	padj	Compartment
C4B63_44g228	4.39780676240455	3.65017935571142e-224	Core
C4B63_4g189	5.11455480068836	9.27004446229479e-174	Core
C4B63_2g185	3.46771933297992	3.71166965141883e-158	Core
C4B63_16g183	-4.72474144842212	1.40772076573635e-150	Core
C4B63_92g110	4.49336868092178	7.52124753575511e-150	Core
C4B63_2g189	3.55318681473989	3.8711943344528e-147	Core
C4B63_57g131	4.06645042956357	1.70470087385029e-146	Core
C4B63_32g172	-5.59852272313013	1.06434630225478e-145	Disruptive
C4B63_13g352	3.78338694828495	8.42519017140943e-140	Core
C4B63_42g60	3.99800337663977	1.46714086795298e-138	Core
C4B63_255g20	2.88421289566167	4.58289364204502e-137	Core
C4B63_11g40	4.31562666342163	3.58237324382589e-133	Core
C4B63_60g203	-5.00141687355655	8.12521152196492e-130	Disruptive
C4B63_44g229	3.75102379947217	1.30541617353329e-129	Core
C4B63_101g12	6.24470969761854	2.04629984660088e-122	Core
C4B63_22g743c	4.18742335503815	9.32668410711444e-122	Core
C4B63_294g11	3.74758261527522	6.22709713778503e-121	Core
C4B63_271g8	-6.174423656345	2.12676183753928e-118	Core
C4B63_193g13	4.53455762823773	7.04459310322354e-115	Core
C4B63_193g16	4.30090783527678	1.09476276983833e-112	Core
C4B63_405g10	3.0549157701906	2.95402279059016e-109	Core
C4B63_43g199	-5.72434737316361	3.6164614785401e-107	Core
C4B63_405g4	3.28457905874017	1.17085511552039e-104	Core
C4B63_33g179	-5.7784468347641	7.22790538852421e-104	Core
C4B63_102g68	3.03950234036034	8.32798934548496e-104	Core
C4B63_54g69	-2.9701912469527	1.21569967111565e-103	Core
C4B63_113g41	4.59693611869778	9.85010876139835e-103	Core
C4B63_16g197	-3.94867214870806	9.88325759577784e-102	Disruptive
C4B63_260g23	-4.46987392529779	1.78615871508952e-101	Disruptive
C4B63_21g343	-4.30447897268749	1.79065201359642e-101	Core
C4B63_12g161	4.50626287291739	1.0669072841224e-100	Core
C4B63_361g13	2.97724085162968	2.65548062272972e-97	Core
C4B63_12g175	4.34750283355184	2.10438647570122e-95	Core
C4B63_21g56	2.86297246388514	8.11180656059028e-95	Core
C4B63_44g223	2.76093292905676	2.1747215407377e-94	Core
C4B63_68g79	-5.0092912355994	6.11464660742695e-94	Core
C4B63_11g67	3.06447089226719	6.50125739494821e-94	Core
C4B63_73g11	-3.30807932926846	2.9123844313979e-91	Disruptive
C4B63_351g27c	4.08423098286249	7.73870321787325e-91	Core
C4B63_251g17	2.80406061073425	2.12369534539811e-90	Core
C4B63_203g15	2.67191287194204	1.15999181177821e-89	Core
C4B63_22g773c	4.25742182984535	1.96518570544583e-89	Core
C4B63_58g229	-3.046693585748	3.80002313866737e-89	Disruptive
C4B63_135g24	-3.64413279344947	8.60748182630183e-85	Disruptive
C4B63_113g42	4.58939195101619	2.20093355124364e-83	Core
C4B63_84g32	-4.790140216572	1.77791348023784e-82	Disruptive
C4B63_101g6	2.80276355063888	1.12509269530734e-80	Core
C4B63_43g170	2.20410071208864	4.69908142546747e-79	Core
C4B63_61g135	2.90471754517368	3.94086914379795e-78	Core
C4B63_148g10	-4.63256036347599	5.53277396578534e-78	Disruptive
C4B63_41g261	-4.00635692905482	1.96202995927882e-77	Disruptive
C4B63_9g383	3.67305223971902	1.22288456274055e-76	Core
C4B63_145g23	-4.22851534182489	3.83951744366735e-76	Disruptive
C4B63_34g324	-6.37165028779805	7.59373713946011e-76	Core
C4B63_87g8	4.32794144870049	3.99346695374207e-75	Core
C4B63_32g43	2.9619620126342	3.29550396458683e-73	Core
C4B63_75g72	-3.70869946725477	1.92003361101679e-72	Disruptive
C4B63_201g18	-5.2557206239953	1.92003361101679e-72	Core
C4B63_68g91	-3.93628484397654	3.55328138781421e-72	Disruptive
C4B63_110g16	2.2298675172681	9.61331205879433e-72	Core
C4B63_336g4	2.5031771368406	9.61331205879433e-72	Core
C4B63_17g205	-4.19807985568949	3.68873567080969e-71	Disruptive
C4B63_70g116	2.46621172717952	5.1365768415527e-71	Core

C4B63_33g192	-5.11974843239028	2.43910527561445e-69	Core
C4B63_40g138	-4.22736024523117	3.10826712543204e-69	Disruptive
C4B63_148g15	-5.25218654354858	7.33728289502809e-69	Disruptive
C4B63_167g33	-3.84262096975289	1.02601942943337e-68	Disruptive
C4B63_63g35	3.43196247118419	1.16422425744614e-68	Core
C4B63_75g105	-4.27314467842901	1.80228713665405e-68	Disruptive
C4B63_56g86	2.42804241150771	1.29802927403368e-66	Core
C4B63_7g299	4.55038856542053	1.5408299034704e-66	Core
C4B63_11g29	2.78332347390135	5.33772045014591e-66	Core
C4B63_19g43	3.10491730075795	1.52690799844392e-65	Core
C4B63_2g547	3.35603412796034	3.89994111724765e-65	Core
C4B63_75g77	-3.66425678678262	4.08935402955155e-64	Disruptive
C4B63_79g110	3.73958951232923	5.38453639029217e-64	Core
C4B63_26g261	2.18622400814612	1.83593334327742e-63	Core
C4B63_41g263	-4.04895524855127	3.21915551355884e-63	Disruptive
C4B63_124g40	-4.73265361026888	5.3104245559368e-63	Disruptive
C4B63_207g15	2.88993437202134	7.02362452565003e-63	Core
C4B63_77g40	-4.16892474354666	8.98300936174355e-63	Disruptive
C4B63_207g13	3.08546528029498	2.50183313594968e-62	Core
C4B63_2g317	-4.72130016026424	3.45953976206187e-62	Disruptive
C4B63_16g155	2.58827008902354	3.45953976206187e-62	Core
C4B63_12g157	3.88738704611823	5.87127969566915e-62	Core
C4B63_46g80	3.81699743943959	1.80836991986591e-61	Core
C4B63_101g3	1.79392641310915	3.32678811035342e-61	Core
C4B63_164g14	-4.01668614507765	8.39664653256879e-61	Disruptive
C4B63_119g19	-4.23001857585789	1.69952646179219e-60	Disruptive
C4B63_34g269	2.86560997002303	3.66045746412811e-60	Disruptive
C4B63_72g38	-4.27401178541962	2.13071013115494e-59	Disruptive
C4B63_51g207	2.63331172005343	3.78132570306502e-59	Core
C4B63_44g146	2.75145771165347	3.47833863795784e-58	Core
C4B63_29g195	-4.3726044359674	4.20874668636744e-58	Core
C4B63_34g122	-6.03169116130265	6.03373364765561e-58	Core
C4B63_13g280	-3.82200290428193	1.81353717651013e-57	Disruptive
C4B63_158g42	-5.48875951887936	3.26143262571997e-57	Core
C4B63_13g292	-4.35585612799878	3.53625049308131e-57	Disruptive
C4B63_1g368	-5.29835937090442	7.66329668405582e-57	Disruptive
C4B63_34g124	3.23011233652365	1.32947430677888e-56	Core
C4B63_119g11	-5.11566492485541	1.39226171946798e-56	Disruptive
C4B63_29g211	-5.04040291777975	2.11813137098154e-56	Core
C4B63_119g31	-4.22611482488168	2.29522615172726e-56	Disruptive
C4B63_41g212	2.03250443462382	1.1566929065958e-55	Core
C4B63_34g275	3.27323143290087	1.6728145322417e-55	Disruptive
C4B63_77g53	-3.85525857940071	2.5224205618016e-55	Disruptive
C4B63_17g257	2.20810919543227	2.91589519874432e-55	Core
C4B63_10g490	1.97891064360277	3.47536947919329e-55	Core
C4B63_87g7	2.1811957807149	1.00881382395815e-54	Core
C4B63_21g240	2.55490470741416	1.01966177157413e-54	Core
C4B63_25g320	-3.03229586913991	2.19724244594392e-54	Core
C4B63_35g99	2.95928907194534	2.83366128891273e-54	Core
C4B63_105g53	-4.42181518896232	2.95027799703218e-54	Disruptive
C4B63_153g14	-3.76223748182195	3.33974395291511e-54	Disruptive
C4B63_14g28	2.17893789639515	3.62601173024983e-54	Core
C4B63_33g156	-4.40197850673804	9.42950483613693e-54	Core
C4B63_41g300	-5.24998371296406	1.80215456108715e-53	Disruptive
C4B63_86g95	-4.57623819963326	3.38509589687483e-53	Disruptive
C4B63_24g202	2.65103077254725	3.80649955393872e-53	Core
C4B63_361g12	3.65763346181667	1.40488769779334e-52	Core
C4B63_144g26	3.12261961452963	1.71146400800959e-52	Core
C4B63_16g181	-3.09020534620524	7.11757662335199e-52	Disruptive
C4B63_97g13	3.01268096431004	2.21055686438887e-51	Core
C4B63_12g221	2.531398271085	1.23006856354966e-50	Core
C4B63_43g92	-3.98063062812967	1.54711722097568e-50	Disruptive
C4B63_3g1125	3.05569885904256	2.57886945610092e-50	Core
C4B63_29g4	2.86550467999643	2.93177369656646e-50	Core

C4B63_93g73	2.03269277305068	3.16064670992744e-50	Core
C4B63_110g48	3.17630165109475	4.50113991350172e-50	Core
C4B63_145g21	-3.249286156561	5.30501217567585e-50	Disruptive
C4B63_42g262	-4.59148481400295	1.05333155983919e-49	Disruptive
C4B63_204g16	-4.33774628001271	1.10222686363102e-49	Disruptive
C4B63_5g433	-4.90093620795171	1.40127596222951e-49	Disruptive
C4B63_105g64	-4.07955543940384	2.03902300886148e-49	Disruptive
C4B63_1g128c	3.57276784160961	2.62712125538976e-49	Core
C4B63_70g50	2.52978638566901	3.2169741056013e-49	Core
C4B63_131g42	-3.29171874886189	3.33966185096349e-49	Disruptive
C4B63_22g322	2.61757825791323	8.46616225268012e-49	Core
C4B63_21g86	1.8328743161422	1.25435800452604e-48	Core
C4B63_2g199	3.12085302715564	1.49690482661307e-48	Core
C4B63_21g165	-3.64374283084444	2.17577864535974e-48	Disruptive
C4B63_84g69	2.99646017639838	3.33842598317267e-48	Core
C4B63_57g113	1.95175889143839	4.40562292931742e-48	Core
C4B63_69g57	3.11066627880904	4.64808031625716e-48	Core
C4B63_2g323	-4.36917953500577	8.19721186795469e-48	Disruptive
C4B63_46g58	2.7596115382573	1.05295515976509e-47	Core
C4B63_109g41	-5.47524988227984	1.15969642085719e-47	Core
C4B63_13g14	-3.63161348639939	1.18909295767587e-47	Disruptive
C4B63_12g170	4.14755591039446	1.23772931368634e-47	Core
C4B63_384g22	-4.34042602175955	1.23772931368634e-47	Disruptive
C4B63_32g51	3.58920293933355	2.10463391283787e-47	Core
C4B63_12g220	2.36091059137269	3.16912232878955e-47	Core
C4B63_28g149	2.99915162080257	3.28164515324041e-47	Core
C4B63_119g5	-4.05802329167267	7.31313160509617e-47	Disruptive
C4B63_6g394	3.02443285874586	1.14570362895711e-46	Core
C4B63_14g229	-3.67227544544486	1.41521983677754e-45	Disruptive
C4B63_38g303	-4.50965474353887	1.94157694659389e-45	Disruptive
C4B63_5g493	-4.78967543563135	1.94993924808282e-45	Disruptive
C4B63_145g8	-3.40398876694899	1.94993924808282e-45	Disruptive
C4B63_8g182	-4.00183405317548	2.00381246442913e-45	Core
C4B63_166g26	-2.57900349994445	5.30129367791388e-45	Disruptive
C4B63_87g138	-3.51633615764851	5.9778771300018e-45	Disruptive
C4B63_39g375	-4.09852531161859	8.56874352690061e-45	Disruptive
C4B63_2g324	-4.30722274238211	1.11272634033852e-44	Disruptive
C4B63_73g69	-4.11856230978276	3.38803379000806e-44	Core
C4B63_13g117	1.66802904762993	3.4562027754891e-44	Core
C4B63_34g1237c	3.07013023412447	6.26154647839571e-44	Disruptive
C4B63_8g560	1.925905472752	1.16575320951058e-43	Core
C4B63_1g788	-4.59245713726055	1.2000287615035e-43	Disruptive
C4B63_157g40	-2.59763895922439	1.53267286053286e-43	Disruptive
C4B63_22g37	2.39844874366011	2.60133489324244e-43	Core
C4B63_34g245	2.23691231733477	2.80782104764209e-43	Disruptive
C4B63_538g1	-5.39240219957689	5.37668948536293e-43	Core
C4B63_34g137	-2.87822003065579	5.97397891490787e-43	Disruptive
C4B63_30g163	2.00395081498385	6.72266541644715e-43	Core
C4B63_49g210	-3.43965063262974	8.17817172345168e-43	Disruptive
C4B63_159g15	-3.57135396174413	8.65918617730166e-43	Disruptive
C4B63_49g106	1.84174069438159	8.8642409562186e-43	Core
C4B63_105g66	-4.5604367322979	9.34201739262385e-43	Disruptive
C4B63_1g473	-3.32203363772439	1.29742439538728e-42	Disruptive
C4B63_212g4	4.72131617502946	1.88345615107488e-42	Core
C4B63_7g298	4.39914811707542	2.65468525117729e-42	Core
C4B63_77g60	-2.41260380054597	4.7642082652659e-42	Disruptive
C4B63_46g181	-4.45035958666405	6.22653112680536e-42	Disruptive
C4B63_1g527	-2.48129352244138	1.18402659013628e-41	Disruptive
C4B63_162g7	-3.20945132310889	1.34897608957231e-41	Disruptive
C4B63_408g15	-3.76878774009151	1.89513963962681e-41	Disruptive
C4B63_34g1226c	3.20853266099535	3.27178655012182e-41	Disruptive
C4B63_9g382	3.15928450269297	3.33828853325128e-41	Core
C4B63_24g260	-5.23865594155748	3.64738839731966e-41	Disruptive
C4B63_105g45	-3.98466245298349	3.91792569539522e-41	Disruptive

C4B63_42g193	-2.03433245121911	4.45421877421545e-41	Core
C4B63_22g28	2.26071427317208	4.76279740647097e-41	Core
C4B63_371g10	-3.81311423574775	6.80748682408084e-41	Disruptive
C4B63_145g11	-3.39689345537099	1.05201338304083e-40	Disruptive
C4B63_122g1	3.46679000472323	1.35029271953146e-40	Core
C4B63_37g419	2.06252817224108	2.17150636458975e-40	Core
C4B63_39g111	-5.04328710970738	2.31533624053129e-40	Disruptive
C4B63_158g35	-5.14126778383665	3.56758586959433e-40	Disruptive
C4B63_8g117	-3.05044591419399	4.89236262882215e-40	Core
C4B63_21g271	2.27126058395779	4.96721154612836e-40	Core
C4B63_82g89	2.68544191014903	8.02170965859455e-40	Core
C4B63_170g1	4.19108541921157	8.51367240595865e-40	Core
C4B63_18g167	2.26233770085185	9.38809337274128e-40	Core
C4B63_138g37	3.18106125821866	1.2057219321234e-39	Core
C4B63_72g79	1.85877453551313	1.37901470487443e-39	Core
C4B63_43g225	3.33328576724323	2.00577183139649e-39	Core
C4B63_58g227	-4.70045697243422	6.15511403254008e-39	Disruptive
C4B63_43g95	-3.80664216367109	6.47625339236584e-39	Disruptive
C4B63_178g41	1.7788461856531	8.93100780576382e-39	Core
C4B63_258g29	2.43022278659351	1.16626694722678e-38	Core
C4B63_8g123	-2.60265154779854	1.23230529773444e-38	Disruptive
C4B63_19g12	-4.00797405328698	1.75242625158463e-38	Core
C4B63_5g131	-4.94132739348802	1.91539475020204e-38	Disruptive
C4B63_387g21	-2.67587029469035	2.11504469507006e-38	Disruptive
C4B63_34g1224c	3.06718087940596	2.23196563998767e-38	Disruptive
C4B63_2g183	3.2915933927999	2.68901721684901e-38	Core
C4B63_18g287	1.92464084804007	3.76092276435955e-38	Core
C4B63_51g234	-3.14977258228872	6.72714526546744e-38	Disruptive
C4B63_23g158	-3.21120584815284	1.00556774219798e-37	Disruptive
C4B63_63g110	2.27053270592211	1.17531001900186e-37	Core
C4B63_30g300	-2.19754477017384	1.58974435560508e-37	Disruptive
C4B63_46g62	3.17167542432136	1.68773235165674e-37	Core
C4B63_34g372	3.60566747942697	2.13311437540831e-37	Core
C4B63_455g9	-2.7979569186012	2.26665819412897e-37	Disruptive
C4B63_210g26	3.5310129396739	2.93092149791553e-37	Core
C4B63_27g337	-5.09625467332641	3.31946860205874e-37	Disruptive
C4B63_77g67	-2.86960639043173	3.79453445845979e-37	Disruptive
C4B63_182g13	2.15567049928704	4.68827051653067e-37	Core
C4B63_168g33	1.46428503221244	4.98500954057998e-37	Core
C4B63_2g661	-4.23778222972261	5.62326469228381e-37	Disruptive
C4B63_34g263	3.87925334212373	1.05518988268822e-36	Disruptive
C4B63_156g20	-4.34683947392776	1.14204670586227e-36	Disruptive
C4B63_82g70	2.58510017727712	1.54498751554558e-36	Core
C4B63_35g363	-4.15445482337412	1.9723332602048e-36	Disruptive
C4B63_22g135	-3.59463020866383	2.13293654340051e-36	Disruptive
C4B63_12g405	2.54149300542652	2.28449124722654e-36	Core
C4B63_8g77	-3.2409487627449	2.33724332798462e-36	Disruptive
C4B63_9g377	3.17834037827266	4.63906002024826e-36	Core
C4B63_2g316	-4.73309233960951	8.8614163613385e-36	Disruptive
C4B63_9g387	2.37170905542394	9.15256476896961e-36	Core
C4B63_43g211	1.84623543742635	1.15940618032358e-35	Core
C4B63_360g18	-3.35963521999336	1.15940618032358e-35	Disruptive
C4B63_119g10	-5.17782092398952	1.34429000883877e-35	Disruptive
C4B63_22g32	2.44334156718421	1.37141838128302e-35	Core
C4B63_7g228	2.2811249579331	1.46801919746343e-35	Core
C4B63_8g61	1.59514750507726	1.46801919746343e-35	Core
C4B63_18g288	1.83088796764896	2.5839299434089e-35	Core
C4B63_84g25	2.83821834693135	3.57933705878942e-35	Core
C4B63_63g102	2.1284756313374	3.84975174499922e-35	Core
C4B63_232g21	2.4600276449028	4.7169736588231e-35	Disruptive
C4B63_78g46	2.45827305128929	5.46051827563509e-35	Core
C4B63_49g168	4.06260464082817	6.00652006361946e-35	Core
C4B63_251g12	2.53755392617668	9.95591271619785e-35	Core
C4B63_155g17	-3.29029096419347	2.07698625230396e-34	Disruptive

C4B63_60g164	2.95004440031636	2.08259937625326e-34	Core
C4B63_153g5	2.40109785139851	2.53586130482381e-34	Core
C4B63_35g334	-3.88361116307192	2.76150691217656e-34	Disruptive
C4B63_49g192	2.07988540565635	2.76150691217656e-34	Core
C4B63_384g21	-4.00648666719031	3.28790281684115e-34	Disruptive
C4B63_4g484	1.59170274247677	3.6585074980264e-34	Core
C4B63_13g282	-2.72461990097317	3.6585074980264e-34	Disruptive
C4B63_281g6	-3.65975802984429	4.59312073237984e-34	Core
C4B63_12g330	1.73397895330367	5.67037930143744e-34	Core
C4B63_16g315	2.09619389701639	6.74858469041467e-34	Core
C4B63_84g67	3.23397199509376	7.74096855658378e-34	Core
C4B63_312g7	3.63838729025492	7.89248183788518e-34	Core
C4B63_109g67	3.3289347212825	9.1079335099531e-34	Core
C4B63_300g22	-5.9140024357064	9.24698097998839e-34	Core
C4B63_231g14	-4.5580335915937	1.42213296987352e-33	Disruptive
C4B63_34g1233c	3.29151149537667	1.47156929942375e-33	Disruptive
C4B63_2g765	1.59232458185701	2.02661918393566e-33	Core
C4B63_39g370	-3.93403448878655	2.04552695866718e-33	Disruptive
C4B63_9g454	1.42753030805585	2.13672452988332e-33	Core
C4B63_1g201	-3.89229247745745	2.59197618958674e-33	Disruptive
C4B63_182g5	2.16082850066593	2.94063338579332e-33	Core
C4B63_80g4	2.13325990713061	3.16310295176823e-33	Core
C4B63_23g10	-3.67886235453042	3.55264650796118e-33	Disruptive
C4B63_5g426	-2.83959362864846	5.08890920578552e-33	Disruptive
C4B63_34g1244c	3.3473194592363	5.44603953368151e-33	Disruptive
C4B63_20g316	2.13515613254288	5.97673092949853e-33	Core
C4B63_77g22	1.85568797378923	6.33907416604547e-33	Core
C4B63_68g74	-3.03564283705283	6.65239156325027e-33	Disruptive
C4B63_2g549	2.92148493518179	6.73755602807643e-33	Core
C4B63_4g490	2.17827958846491	8.95872789985761e-33	Core
C4B63_34g1236c	2.94757894830136	8.95872789985761e-33	Disruptive
C4B63_39g364	-3.94870766831988	9.90439330420963e-33	Disruptive
C4B63_48g144	1.97357203633671	1.13659417366376e-32	Core
C4B63_47g111	2.67195667641681	1.65419334656526e-32	Core
C4B63_109g42	3.27178777229149	2.25802532746195e-32	Core
C4B63_22g137	-2.80271543017627	3.60825029906377e-32	Disruptive
C4B63_21g268	2.21534221205807	4.14218547754091e-32	Core
C4B63_44g218	2.28180566033259	6.05453861897097e-32	Core
C4B63_4g334	-3.30657133617918	6.27273262816234e-32	Core
C4B63_295g26	1.7493275741709	6.70330125299944e-32	Core
C4B63_155g10	2.33237615022602	7.45725579685572e-32	Disruptive
C4B63_4g462	3.01622273737365	8.16845484899692e-32	Core
C4B63_10g266	-4.30324107971088	8.7020279077921e-32	Disruptive
C4B63_226g20	-3.50580470867993	9.02046127649716e-32	Disruptive
C4B63_161g32	2.93447683631071	1.5228544106069e-31	Core
C4B63_122g20	3.37455434572147	1.73098399106072e-31	Core
C4B63_196g38	-5.0061096690121	1.89780943998539e-31	Disruptive
C4B63_111g19	-2.18645973589561	1.99990607449329e-31	Disruptive
C4B63_1g130c	3.22505518272507	2.03470990272378e-31	Core
C4B63_130g12	-2.67702924748448	2.53774451110156e-31	Core
C4B63_11g45	2.81273109915314	3.64538649902273e-31	Core
C4B63_43g175	1.85006093398197	4.37198675244242e-31	Core
C4B63_2g548	3.19628705073738	4.76239825074962e-31	Core
C4B63_30g275	3.41242104672972	5.25593223649303e-31	Core
C4B63_27g282	-4.11737521776432	5.569753357487e-31	Disruptive
C4B63_6g435	2.00530606360636	6.50737301804906e-31	Core
C4B63_6g263	2.26714825525409	7.0979926383546e-31	Core
C4B63_63g143	-3.54686581251553	8.06130796610196e-31	Disruptive
C4B63_22g131	-3.29280838220949	9.26770277041633e-31	Disruptive
C4B63_93g12	-5.14910527677729	9.94198805715115e-31	Disruptive
C4B63_55g287c	2.92961946982592	1.1414792379811e-30	Core
C4B63_1g480	-3.20234000731295	1.2612099452399e-30	Disruptive
C4B63_101g4	2.87410067931773	1.47094870563842e-30	Core
C4B63_24g306	2.0046629513279	1.78014654265849e-30	Core

C4B63_9g279	1.42065762727653	1.84873146176703e-30	Core
C4B63_35g193	-2.15525468420837	2.10333710694824e-30	Disruptive
C4B63_238g9	2.2266633420423	2.26438726636167e-30	Core
C4B63_258g21	2.91376788123892	2.28150957662788e-30	Core
C4B63_45g238	2.47493295084241	2.79753700260795e-30	Core
C4B63_51g117	2.24528672474121	2.93619876316563e-30	Core
C4B63_419g2	-2.92516588012211	3.48422448200209e-30	Disruptive
C4B63_62g3	-4.44059425387633	3.92147228857347e-30	Disruptive
C4B63_5g740	-3.66335615598291	6.51952016501437e-30	Disruptive
C4B63_3g554	2.54263370631075	9.15007151672746e-30	Core
C4B63_105g48	-3.29270645130728	9.34377374624615e-30	Disruptive
C4B63_13g5	-3.82662777170894	1.08325248576335e-29	Disruptive
C4B63_67g58	-3.7080287802437	1.19024215247859e-29	Disruptive
C4B63_82g81	2.24503030698393	1.19101437889513e-29	Core
C4B63_52g312c	2.08606009497186	1.21460757468205e-29	Core
C4B63_13g310	1.87732657904992	1.50003303018e-29	Core
C4B63_5g376	-4.48747293706945	1.69955302644952e-29	Disruptive
C4B63_9g480	1.82622590167147	1.86960091646677e-29	Core
C4B63_156g18	-3.09088053708512	2.14917922121474e-29	Disruptive
C4B63_28g340	-3.41330381214956	2.22045272115128e-29	Disruptive
C4B63_22g738c	2.8709728546584	2.73755260310451e-29	Core
C4B63_7g361	2.20934463094617	3.00848259416202e-29	Core
C4B63_70g119	2.61731764627842	3.76682270400285e-29	Core
C4B63_37g387	-2.92941800224604	3.7988960001609e-29	Disruptive
C4B63_60g78	1.93930776094014	3.92160591364057e-29	Core
C4B63_19g13	-2.90301926633607	4.42233625293673e-29	Core
C4B63_34g248	3.19320886879163	4.50836008397094e-29	Disruptive
C4B63_47g92	-2.99817663204772	4.5532689760394e-29	Disruptive
C4B63_153g41	1.60708665206017	4.5532689760394e-29	Core
C4B63_147g63	-4.04908240578716	4.70854250443087e-29	Disruptive
C4B63_60g204	-3.15484121870501	4.73082420009027e-29	Disruptive
C4B63_45g173	2.7459592923642	4.95294360422235e-29	Core
C4B63_209g4	-2.94606035759097	5.78775158093097e-29	Disruptive
C4B63_203g11	1.90870251748624	9.83013636615337e-29	Core
C4B63_81g19	2.57286232587872	1.26360739981836e-28	Core
C4B63_45g193	2.77307390862173	1.49045439377991e-28	Core
C4B63_34g1223c	3.0383791929683	1.6291114208143e-28	Disruptive
C4B63_333g14	1.01452905571892	1.72675202392248e-28	Core
C4B63_25g202	2.12154644938186	1.74844352052939e-28	Core
C4B63_60g179	-2.58739057507198	2.25525540969303e-28	Disruptive
C4B63_131g47	-2.56793254358528	2.82292676386174e-28	Disruptive
C4B63_9g443	2.03112198171634	2.89401171809454e-28	Core
C4B63_227g12	2.56964878095496	3.96786228122806e-28	Core
C4B63_16g202	-2.92913186560745	4.76927459812172e-28	Disruptive
C4B63_34g297	2.69986399174506	5.03730473403803e-28	Core
C4B63_84g21	1.63064118441788	5.03730473403803e-28	Core
C4B63_27g302	-3.4379672506604	5.21089954307413e-28	Disruptive
C4B63_431g8	2.85958532450698	5.21400146617308e-28	Core
C4B63_24g283	-4.06809464396519	6.63090784911603e-28	Disruptive
C4B63_39g154	-4.24169625570714	9.48138478633017e-28	Disruptive
C4B63_153g8	1.71530459163251	9.78098903488533e-28	Core
C4B63_4g269	-1.93983843132394	1.02156820284891e-27	Core
C4B63_12g227	2.64580608354513	1.07507543099861e-27	Core
C4B63_343g14	-3.25830224132361	1.18851023326473e-27	Disruptive
C4B63_47g128	-3.3924393758384	1.58560083021636e-27	Disruptive
C4B63_9g430	-1.82246090644881	2.29237589045991e-27	Core
C4B63_82g111	-3.1473284665068	2.46876209929581e-27	Disruptive
C4B63_277g2	1.78164303335347	2.82307688601467e-27	Core
C4B63_54g96	-3.11880527468404	5.06218143348814e-27	Disruptive
C4B63_10g455	2.34894939497816	5.36056866290471e-27	Core
C4B63_309g8	2.10441954087137	5.42245249485759e-27	Core
C4B63_12g162	3.92151879071753	9.36220267022593e-27	Core
C4B63_32g206	2.64844533710294	9.4524038410788e-27	Core
C4B63_88g102	-4.22390521243998	1.22941150152919e-26	Disruptive

C4B63_10g296	-1.22011197390403	1.2481124301407e-26	Core
C4B63_21g180	-3.40929399993632	1.3628959981388e-26	Disruptive
C4B63_12g232	1.5143031001198	1.57208370988041e-26	Core
C4B63_251g13	2.74136658809525	1.57208370988041e-26	Core
C4B63_459g9	2.37698216580382	1.57208370988041e-26	Core
C4B63_279g11	2.6182871899487	1.94231463709158e-26	Core
C4B63_12g386	2.00778729907804	1.99023657956353e-26	Core
C4B63_39g55	-4.38521677405365	2.13313345936143e-26	Disruptive
C4B63_25g206	2.45918014309905	3.01220956269369e-26	Core
C4B63_30g222	2.3023887770321	3.11212045957919e-26	Core
C4B63_12g226	2.16004675860523	3.12348129125445e-26	Core
C4B63_56g41	3.6934290061546	3.3938760247803e-26	Core
C4B63_77g50	-2.69404786257686	3.68137649778973e-26	Disruptive
C4B63_22g206	-3.50292274099252	4.76478958439493e-26	Disruptive
C4B63_10g454	2.23465803914371	4.84797745592539e-26	Core
C4B63_3g454	-4.03401802797508	5.56621752102605e-26	Disruptive
C4B63_375g1	2.46982441736076	5.57585524069702e-26	Disruptive
C4B63_20g11	-1.92462814404654	6.34983088844324e-26	Core
C4B63_43g210	1.83243457464107	6.81112780921684e-26	Core
C4B63_187g13	-2.92507058265314	6.81112780921684e-26	Disruptive
C4B63_81g62	2.56703893270954	7.7229389626367e-26	Core
C4B63_84g54	1.73536621182982	8.55824536621244e-26	Core
C4B63_387g20	-3.17315687216291	8.76627467700749e-26	Disruptive
C4B63_43g229	-3.47207987739642	9.52232691162443e-26	Disruptive
C4B63_104g102	3.13793947897164	9.75983822886928e-26	Core
C4B63_46g66	1.91539989748652	1.45070153611952e-25	Core
C4B63_20g162	2.67132799856127	1.45420935496442e-25	Core
C4B63_246g1	-4.71119329182023	1.66659100201349e-25	Core
C4B63_12g109	2.94459108407518	1.77303673616877e-25	Core
C4B63_62g98	-3.2976703964626	1.83311571642758e-25	Disruptive
C4B63_154g5	-3.51763630350903	1.84038754749136e-25	Disruptive
C4B63_143g49	1.86140870664242	2.0231560958372e-25	Core
C4B63_46g75	1.79429807183409	2.20375732537928e-25	Core
C4B63_194g18	1.74086328390791	2.40849944006584e-25	Core
C4B63_406g4	2.67051230326315	2.41934148860512e-25	Core
C4B63_16g17	1.70149346797573	2.55853157207662e-25	Core
C4B63_9g376	3.25994878105212	2.88824074470975e-25	Core
C4B63_28g224	1.79218092254387	2.88824074470975e-25	Core
C4B63_47g131	-3.07595053098223	2.88824074470975e-25	Disruptive
C4B63_37g133	-3.65176158102895	3.35869654640462e-25	Disruptive
C4B63_225g35	1.49380097895905	3.70508442135997e-25	Core
C4B63_10g157	2.67769310284779	4.32285544156432e-25	Core
C4B63_6g537	1.34087758691824	4.39517034962917e-25	Core
C4B63_52g64	2.84291048737445	4.55937909214047e-25	Core
C4B63_5g356	2.47540299217268	4.58067115973288e-25	Disruptive
C4B63_12g20	2.1349458300697	6.43009506375467e-25	Core
C4B63_32g225	1.38398713087844	8.25261405777353e-25	Core
C4B63_76g17	2.97207001898301	8.38682141044257e-25	Core
C4B63_32g259	1.81437322275988	1.02569820687367e-24	Core
C4B63_91g93	-3.87278666281813	1.05727431582978e-24	Disruptive
C4B63_39g336	1.5299056796709	1.11932500701431e-24	Core
C4B63_4g485	1.53898666206432	1.20039802787867e-24	Core
C4B63_9g478	-3.08304122303528	1.26626049857246e-24	Disruptive
C4B63_43g230	-3.76109390200756	1.36450253003718e-24	Disruptive
C4B63_126g15	-3.83553277400556	1.37798199438586e-24	Disruptive
C4B63_14g241	1.16323372227499	1.40957099051527e-24	Core
C4B63_46g113	-3.56533694560378	1.41149546757306e-24	Disruptive
C4B63_417g17	-3.81709860522556	1.41149546757306e-24	Disruptive
C4B63_94g87	-3.20529144195883	1.75842195748479e-24	Disruptive
C4B63_8g285	-3.90721985597768	1.80858960055506e-24	Disruptive
C4B63_22g125	-3.09478774143873	1.81582856292745e-24	Disruptive
C4B63_54g70	2.09837117466447	1.9454320240793e-24	Core
C4B63_19g221	2.86874971709741	2.13126646803373e-24	Core
C4B63_19g185	2.33797488267084	2.45580945000252e-24	Core

C4B63_82g85	1.89488831820549	2.45580945000252e-24	Core
C4B63_447g6	-3.80300291737599	2.61226667026098e-24	Disruptive
C4B63_14g2	15.2359294053271	3.1938861862996e-24	Core
C4B63_60g163	2.83103064799353	3.25554358531532e-24	Core
C4B63_18g239	1.79935110171429	3.63256579443006e-24	Core
C4B63_131g40	-2.02320798344683	3.75835072498901e-24	Disruptive
C4B63_27g278	-3.61892560500479	3.96770010026233e-24	Disruptive
C4B63_13g388	-2.63784576391538	4.05818760459005e-24	Disruptive
C4B63_14g39	1.92601741550838	4.05818760459005e-24	Core
C4B63_58g112	1.52708209938217	4.62598013043931e-24	Core
C4B63_50g243	-4.26541112725458	4.75097561412826e-24	Core
C4B63_4g172	2.09334773347329	4.88204431277085e-24	Core
C4B63_42g61	3.12099527188542	5.28079597202117e-24	Core
C4B63_119g1	-3.51499582094741	5.32388241366292e-24	Disruptive
C4B63_3g943	-3.76838011785859	8.11286001128877e-24	Disruptive
C4B63_53g163	2.63206476628035	8.23735346184155e-24	Core
C4B63_56g144	-4.39872139996678	8.48791159402574e-24	Disruptive
C4B63_3g1101	1.59972731345193	9.49661421736857e-24	Core
C4B63_174g27	-3.64627101553244	9.90156735017736e-24	Disruptive
C4B63_212g14	1.42400927551991	1.01833845123345e-23	Core
C4B63_1g748	-3.04918496521967	1.09088341633762e-23	Disruptive
C4B63_130g42	1.20482472116193	1.12631425142371e-23	Core
C4B63_396g6	2.08333613754304	1.21615429320502e-23	Core
C4B63_43g87	-2.50509344059821	1.31356814244217e-23	Disruptive
C4B63_11g196	-3.70547440845676	1.36015236788075e-23	Disruptive
C4B63_11g484	1.25175666116937	1.53026405738328e-23	Core
C4B63_124g15	-2.08606268225749	1.5459036344404e-23	Disruptive
C4B63_26g240	1.94683774072069	1.61586668264723e-23	Core
C4B63_13g11	-3.99223534304735	2.07622779590164e-23	Disruptive
C4B63_155g14	-3.87614990889952	2.66563467982667e-23	Disruptive
C4B63_35g3	-3.35006209349694	2.7081626521835e-23	Core
C4B63_39g365	-4.04728672675745	2.86207656800124e-23	Disruptive
C4B63_7g356	1.63853676782586	3.23375076409595e-23	Core
C4B63_5g424	-2.89905403299403	3.26289506880018e-23	Disruptive
C4B63_28g222	3.06210496491154	3.28893593650691e-23	Core
C4B63_38g169	1.98909324590646	3.35415962791333e-23	Core
C4B63_23g217	1.08829726938584	3.57021905785117e-23	Core
C4B63_31g260	-2.78891592488831	3.76635799122481e-23	Disruptive
C4B63_1g1100	-3.79374136331325	3.76957116913262e-23	Disruptive
C4B63_22g764c	1.78745440640156	4.93005648129603e-23	Core
C4B63_1g81	-3.13803965177659	5.53603438965677e-23	Disruptive
C4B63_5g432	-4.03973297872479	5.8845714354069e-23	Disruptive
C4B63_37g57	-2.86353620887712	6.75753822279744e-23	Disruptive
C4B63_10g522	1.12757801694813	9.12579252395976e-23	Core
C4B63_6g514	1.80094394367365	9.50959767612578e-23	Core
C4B63_25g203	2.17161741565025	1.07580186508685e-22	Core
C4B63_68g103	-2.71351793177747	1.1412145555639e-22	Disruptive
C4B63_27g3	2.74197016893697	1.17922656320267e-22	Core
C4B63_157g44	-3.06877251676178	1.43294851868808e-22	Disruptive
C4B63_4g184	1.19666384107238	1.57954280357162e-22	Core
C4B63_39g324	2.14071486476559	1.59763847417727e-22	Core
C4B63_97g14	2.28413205390593	1.71877102977959e-22	Core
C4B63_2g318	2.34229932457801	1.9473396643313e-22	Core
C4B63_7g313	1.22314179059367	2.0687556737262e-22	Core
C4B63_32g281	-2.5917991427643	2.2314155501395e-22	Disruptive
C4B63_68g131	-3.04677490253873	2.3244622616777e-22	Disruptive
C4B63_49g227	-3.63117787352849	2.37397448394464e-22	Disruptive
C4B63_9g373	1.88147353474647	2.6431209377052e-22	Core
C4B63_6g273	1.53847215198422	2.68762478626027e-22	Core
C4B63_1g666	-3.71630947191499	3.41124325797229e-22	Disruptive
C4B63_84g68	2.52048667990099	3.53510505696003e-22	Core
C4B63_25g29	2.47376257712672	3.66565218468712e-22	Disruptive
C4B63_94g53	2.27597037518612	4.12363716134431e-22	Core
C4B63_15g19	2.61607270366534	4.93688709187598e-22	Core

C4B63_27g230	1.41541693492935	5.24470566362918e-22	Core
C4B63_197g20	-2.87684182646995	5.24470566362918e-22	Core
C4B63_34g285	2.55412244616954	5.59659480335008e-22	Core
C4B63_106g19	-2.63511234906834	5.64076155101643e-22	Disruptive
C4B63_183g11	1.77394585210825	5.74300798262198e-22	Core
C4B63_31g225	1.5918575281883	5.80527411680805e-22	Core
C4B63_20g114	1.7347559151392	6.07098250208736e-22	Core
C4B63_30g113	-3.42594632553709	6.14645695359284e-22	Core
C4B63_78g52	-2.41833311959928	6.92530603995222e-22	Core
C4B63_237g2	2.35310638131262	7.04974649344298e-22	Core
C4B63_2g33	1.78051211942488	7.31942229616191e-22	Core
C4B63_82g114	-2.74129760822576	8.00702845116945e-22	Disruptive
C4B63_75g56	1.87606564496954	8.87300388973097e-22	Core
C4B63_24g250	-2.94496262694509	9.94018698046133e-22	Disruptive
C4B63_24g215	2.2786987363469	1.00324736438418e-21	Core
C4B63_68g70	-1.73380597135339	1.0131207733619e-21	Core
C4B63_28g223	3.8565646817689	1.1987175581427e-21	Core
C4B63_328g10	1.59214531455521	1.28302919217025e-21	Core
C4B63_19g103	1.96785770764883	1.54989070510663e-21	Core
C4B63_19g220	2.33705317938847	1.64788199064081e-21	Core
C4B63_333g17	14.3464000618405	1.66335448087403e-21	Core
C4B63_19g692c	1.49147597783855	1.69414298366524e-21	Core
C4B63_14g20	-15.8020955977961	1.99060973450768e-21	Core
C4B63_16g302	1.98840942819619	1.99060973450768e-21	Core
C4B63_2g712	2.02844899206092	2.25541225704578e-21	Core
C4B63_46g190	2.34247999891933	2.27884013654572e-21	Core
C4B63_44g231	-2.19211990627414	3.02015374769447e-21	Core
C4B63_20g219	2.23786170319895	3.18022068677587e-21	Core
C4B63_7g438	2.1064854369607	3.21006486227637e-21	Core
C4B63_64g80	-3.40353373730068	3.32691099880416e-21	Disruptive
C4B63_20g10	14.2627462668434	3.837489461709e-21	Core
C4B63_7g81	1.23780659189966	3.93402224095279e-21	Core
C4B63_4g509	1.52386167260157	4.47327574166333e-21	Core
C4B63_2g1634c	1.64580389833846	5.18648512739847e-21	Core
C4B63_13g132	1.79244073850014	5.49800226794228e-21	Core
C4B63_37g420	2.25899557464919	5.49800226794228e-21	Core
C4B63_38g180	2.16661532240825	5.78044460870881e-21	Core
C4B63_26g310	1.77627230069812	5.89740521266014e-21	Core
C4B63_205g52	-3.39005969256866	6.39662956843208e-21	Disruptive
C4B63_196g33	-3.81978263359918	7.11519494014604e-21	Disruptive
C4B63_294g13	2.67243802850646	7.39784033807785e-21	Core
C4B63_19g238	-4.09576860104103	7.66293905601266e-21	Disruptive
C4B63_351g33c	-2.28416827547887	7.66293905601266e-21	Core
C4B63_62g195	-3.51743065224098	7.75185759163953e-21	Disruptive
C4B63_11g131	-3.51853941816617	8.78686608411357e-21	Disruptive
C4B63_34g127	-2.42931513103563	1.10400687961904e-20	Disruptive
C4B63_46g101	2.32647276383109	1.23245179632445e-20	Core
C4B63_6g256	1.24214733057688	1.26029371447742e-20	Core
C4B63_9g445	2.11231516168304	1.26029371447742e-20	Core
C4B63_38g170	1.40035557100156	1.31061334587538e-20	Core
C4B63_8g282	-3.11171456570389	1.32979821099986e-20	Disruptive
C4B63_7g236	2.19465507615334	1.60935944974011e-20	Core
C4B63_101g36	1.6364327096665	1.64478713578951e-20	Core
C4B63_35g131	-2.37609640819107	1.67056630662766e-20	Disruptive
C4B63_246g14	1.55428366596622	2.51725474741364e-20	Core
C4B63_23g21	2.31486405637694	2.53890298518611e-20	Core
C4B63_16g176	-2.35510427716865	2.59780948749331e-20	Core
C4B63_2g782	1.245434153811	2.69038616265346e-20	Core
C4B63_42g180	2.03344727865613	2.9285623244108e-20	Core
C4B63_31g250	-2.21621148712546	2.99289204355387e-20	Disruptive
C4B63_53g146	2.27467788800739	3.32171901048593e-20	Core
C4B63_206g14	2.14974684095543	3.32171901048593e-20	Core
C4B63_54g71	2.05590018579016	3.5770637963948e-20	Core
C4B63_38g53	1.39410922110516	3.74788201130265e-20	Core

C4B63_38g172	1.33402529266003	4.48842430299134e-20	Core
C4B63_247g14	1.83513608443284	4.63896134362918e-20	Core
C4B63_41g154	-2.74605574028396	5.18631958476548e-20	Core
C4B63_235g1	3.71744342120703	5.49573753620276e-20	Core
C4B63_35g143	-2.32308031174564	6.77151989081916e-20	Disruptive
C4B63_212g5	4.37018081926713	6.96049967765674e-20	Core
C4B63_33g100	-2.51957926770466	7.40529306932008e-20	Disruptive
C4B63_271g10	1.34585571984838	7.88524727606766e-20	Core
C4B63_1g1402	1.75828933259373	8.08425535945907e-20	Core
C4B63_58g123	1.98717508894985	8.11308971524313e-20	Core
C4B63_70g139	4.30509792729029	9.05776766640807e-20	Core
C4B63_59g221	-4.30595783639691	9.14176259245252e-20	Disruptive
C4B63_45g129	2.34744618679141	9.54641496128068e-20	Core
C4B63_30g231	1.49694306710854	1.06168786417703e-19	Core
C4B63_42g98	1.53152425836899	1.10334274457125e-19	Core
C4B63_20g178	1.35840311222482	1.18033098869641e-19	Core
C4B63_8g196	1.69654632133847	1.72736232238289e-19	Core
C4B63_8g66	-3.50293691511262	1.72764239866621e-19	Core
C4B63_31g226	1.92121744638552	1.74581319311794e-19	Core
C4B63_83g92	2.62964513720176	1.91228112318603e-19	Core
C4B63_45g174	3.32772367353297	1.97351748042407e-19	Core
C4B63_47g122	-2.82976224602151	2.06648692641752e-19	Disruptive
C4B63_36g86	-3.71666272370589	2.16510381878288e-19	Disruptive
C4B63_50g232	-4.15294669751977	2.50289777204418e-19	Core
C4B63_70g94	2.26874924606305	2.93810353032204e-19	Core
C4B63_250g18	1.52103349924157	3.35221550510776e-19	Core
C4B63_38g173	1.25565236364242	3.56357192914564e-19	Core
C4B63_25g186	2.15413443857147	3.7057580099329e-19	Core
C4B63_212g9	3.51482477293764	3.8012979526555e-19	Core
C4B63_28g32	-1.55728021477625	3.96536749807273e-19	Core
C4B63_82g75	1.32645454003642	4.46753976480664e-19	Core
C4B63_44g221	1.53822738036109	4.89308932103606e-19	Core
C4B63_8g318	-3.10589046506654	4.963391565339e-19	Core
C4B63_8g2646c	1.30920505020949	4.97178234643384e-19	Core
C4B63_172g41	-3.08631526134445	5.04505838193912e-19	Disruptive
C4B63_159g21	-3.26838576524983	5.32873248909025e-19	Disruptive
C4B63_26g139	-2.32575665822647	5.36367464835708e-19	Core
C4B63_208g37	1.70209356809814	5.78773238810341e-19	Core
C4B63_51g112	1.80674609453863	6.32981538990356e-19	Core
C4B63_558g4	-3.28594667138386	6.42701417990824e-19	Disruptive
C4B63_280g17	1.61934190349674	6.73817479332706e-19	Core
C4B63_2g30	1.58659473101646	7.42701043264228e-19	Core
C4B63_6g255	1.62038323025992	8.00212549653036e-19	Core
C4B63_5g155	-3.59008091896784	8.64011319056815e-19	Disruptive
C4B63_13g176	-2.14667447526987	8.87148228193645e-19	Disruptive
C4B63_30g118	1.49409190966933	9.13598570263699e-19	Core
C4B63_1g1037	-3.84530107137156	9.24327258054812e-19	Disruptive
C4B63_1g159	-4.4300083819552	1.05616469612483e-18	Disruptive
C4B63_19g218	1.80108645753064	1.05616469612483e-18	Core
C4B63_59g102	-2.46887537293858	1.17774698180029e-18	Disruptive
C4B63_21g281	2.23381563733444	1.22499210193759e-18	Core
C4B63_19g222	2.97704774623543	1.27104530218894e-18	Core
C4B63_7g117	-1.30412373104851	1.31156287483596e-18	Core
C4B63_69g74	1.35658675541897	1.32689700709891e-18	Core
C4B63_60g180	-2.75209852142225	1.33340374882929e-18	Disruptive
C4B63_7g72	2.54028196853593	1.39702250841107e-18	Core
C4B63_63g86	1.43052451918264	1.50668913713664e-18	Core
C4B63_9g108	-3.7649658251101	1.72189341022666e-18	Disruptive
C4B63_12g171	1.30901930953147	1.72189341022666e-18	Core
C4B63_13g10	-3.33308648380869	1.72266992472082e-18	Disruptive
C4B63_6g104	1.34802033534944	2.01556496442156e-18	Core
C4B63_60g110	-1.88387389551835	2.27061283953673e-18	Disruptive
C4B63_11g262	1.22174643994234	2.7997560642992e-18	Core
C4B63_6g235	1.76947899175941	2.86292540061744e-18	Core

C4B63_43g203	1.48990315470678	2.86292540061744e-18	Core
C4B63_43g202	1.30231505788645	3.12159116677322e-18	Core
C4B63_10g453	1.94205791454401	3.12651473755759e-18	Core
C4B63_18g221	1.24752963955999	3.33431493576419e-18	Core
C4B63_6g117	-1.84448607901331	3.66027738311019e-18	Core
C4B63_2g3	-2.04794206776381	3.68307813064011e-18	Core
C4B63_9g319	2.0849012365936	3.69553742650493e-18	Core
C4B63_295g27	1.23953016354059	3.70025419083928e-18	Core
C4B63_74g4	2.38295025696755	3.71461222976723e-18	Core
C4B63_54g18	1.695238338117	3.71915593726399e-18	Core
C4B63_10g127	2.06821635903321	4.00017091494682e-18	Core
C4B63_38g39	1.74272988526974	4.31237163217496e-18	Core
C4B63_58g38	1.75501853187774	4.42293358095318e-18	Core
C4B63_371g9	-3.35532049445101	4.42293358095318e-18	Disruptive
C4B63_5g754	-3.77797340252823	4.7900751751909e-18	Disruptive
C4B63_88g112	-4.02765356224275	5.13930152436645e-18	Disruptive
C4B63_277g7	1.93452696128322	5.27272709017688e-18	Core
C4B63_98g34	1.93031937290824	5.3993599905551e-18	Disruptive
C4B63_216g7	2.05901482771163	5.54174303988735e-18	Core
C4B63_17g268	1.964528233239	5.78466117210726e-18	Core
C4B63_10g538	-2.48689428676607	5.86378057880017e-18	Disruptive
C4B63_60g90	-2.2549610000907	5.90116978615326e-18	Disruptive
C4B63_46g74	1.28388748958379	6.2235436341377e-18	Core
C4B63_68g107	-2.70348327130035	7.4740233799058e-18	Disruptive
C4B63_106g63	-3.25236118813042	7.82824318300694e-18	Disruptive
C4B63_65g27	-3.44763524150273	7.90545512052479e-18	Core
C4B63_19g228	1.99377681917923	7.93252490614944e-18	Core
C4B63_48g44	1.38079741981067	8.02100459094445e-18	Core
C4B63_24g291	-4.06191756582123	1.15743783313423e-17	Disruptive
C4B63_18g331	2.70248002527309	1.17994886954727e-17	Core
C4B63_100g14	-2.84271114591485	1.49814159824178e-17	Disruptive
C4B63_13g344	1.19604720530353	1.671512923763e-17	Core
C4B63_8g172	-2.78384499272444	1.83058054743925e-17	Disruptive
C4B63_54g99	-3.19466322710883	2.35923998954561e-17	Disruptive
C4B63_43g204	2.06351102993964	2.45908484832421e-17	Core
C4B63_64g50	-3.39234890196782	2.66049399589524e-17	Disruptive
C4B63_68g121	-2.10241868315906	2.83677115162169e-17	Disruptive
C4B63_45g130	2.36721925275276	2.84153795803006e-17	Core
C4B63_37g100	-2.5084009739709	2.8853956503442e-17	Disruptive
C4B63_2g702	1.10289278097397	2.88700494217496e-17	Core
C4B63_5g198	-4.28888289656848	2.95931481858051e-17	Disruptive
C4B63_48g159	1.14421606138844	3.31966375211689e-17	Core
C4B63_116g59	-3.07755142673473	3.44790819493653e-17	Disruptive
C4B63_568g1	-2.58596784308074	3.60470934264018e-17	Disruptive
C4B63_1g106	-2.87520340998232	3.61310057671461e-17	Disruptive
C4B63_62g100	-2.33885457830494	3.72641506967334e-17	Disruptive
C4B63_124g47	-3.69531910483448	3.74227271377134e-17	Disruptive
C4B63_159g4	-1.95914699675895	3.80941343288146e-17	Disruptive
C4B63_107g82	-3.42288876283687	4.24022280389474e-17	Disruptive
C4B63_124g14	-1.94058915708549	4.24858645760247e-17	Disruptive
C4B63_32g30	1.18896772285793	4.25387574924194e-17	Core
C4B63_55g87	1.31486000719783	4.403503423603e-17	Core
C4B63_83g73	1.16911282235548	4.53662072478314e-17	Core
C4B63_26g309	1.63362002795704	4.63136297330083e-17	Core
C4B63_25g240	1.85071186724117	4.67233896128847e-17	Core
C4B63_3g1022	-2.94181293377847	4.82457901022346e-17	Disruptive
C4B63_1g558	-1.92710754384301	4.84380387613551e-17	Core
C4B63_8g152	-2.38288373614788	5.46805337057021e-17	Disruptive
C4B63_31g230	-2.24363597911847	5.64267595132983e-17	Disruptive
C4B63_139g64	-3.5713991029619	5.83459321764053e-17	Disruptive
C4B63_262g21	1.57809257542687	6.77138086652993e-17	Core
C4B63_5g648	-3.76333193834778	6.88578417336815e-17	Disruptive
C4B63_2g77	1.0728914274075	6.9132301413149e-17	Core
C4B63_64g126	-3.48253483559726	7.06688099455362e-17	Disruptive

C4B63_27g292	-4.34857268368657	7.63252772369059e-17	Disruptive
C4B63_8g163	-2.56733645427619	7.89160550991591e-17	Core
C4B63_53g174	-2.10689835057457	8.66168718807234e-17	Core
C4B63_112g36	1.46672296545975	9.78145568238731e-17	Core
C4B63_24g253	-2.54819400949312	9.88401844004775e-17	Disruptive
C4B63_34g211	3.55789979852589	9.9065701274151e-17	Core
C4B63_1g806	-3.54536310481073	1.04998502389705e-16	Disruptive
C4B63_37g119	-3.28270894148695	1.15894430712392e-16	Disruptive
C4B63_69g26	-1.58253584925873	1.17472638904074e-16	Core
C4B63_1g25	1.68082512392907	1.25619411837236e-16	Core
C4B63_34g146	-3.09344406793241	1.34973689779482e-16	Disruptive
C4B63_18g39	2.26570348016306	1.35171471764853e-16	Core
C4B63_35g9	-3.51647593033859	1.38478280687628e-16	Core
C4B63_46g105	1.99858217876745	1.64725292879643e-16	Core
C4B63_11g213	-2.90394969626308	1.66093279587275e-16	Disruptive
C4B63_41g208	1.5337992879133	1.66093279587275e-16	Core
C4B63_8g141	-2.90993449184408	1.74270365213205e-16	Disruptive
C4B63_23g16	2.16586940879444	1.86476132264144e-16	Core
C4B63_15g253	-3.28934837597098	2.04645825115041e-16	Disruptive
C4B63_32g309	-2.63713674977529	2.10608781294014e-16	Disruptive
C4B63_133g46	2.64841746709211	2.12170809417884e-16	Core
C4B63_150g12	-3.2817505071144	2.14585002820192e-16	Disruptive
C4B63_5g167	-5.51964242548206	2.19501434668627e-16	Disruptive
C4B63_167g40	-2.65073050706961	2.20370279784646e-16	Disruptive
C4B63_169g39	-2.83861548306225	2.53872867223159e-16	Disruptive
C4B63_38g310	-2.38765916866707	2.59070351141668e-16	Disruptive
C4B63_15g419	-3.17276783165602	2.97510905110369e-16	Disruptive
C4B63_24g325	1.30880986832873	2.97510905110369e-16	Core
C4B63_35g136	-1.88061486740024	3.10104522818443e-16	Disruptive
C4B63_8g280	-3.91843175640248	3.35441526944795e-16	Disruptive
C4B63_17g85	2.18146260185509	3.69385893371066e-16	Core
C4B63_27g322	-4.72587867705624	3.84482927460641e-16	Disruptive
C4B63_39g233	-2.69055444145465	4.05272181788928e-16	Disruptive
C4B63_25g248	1.30104724497673	4.25723381629369e-16	Core
C4B63_19g183	1.62785520711093	4.30067064964157e-16	Core
C4B63_112g67	-1.92582504947127	4.34386208415127e-16	Core
C4B63_46g189	1.58623425686097	4.36217359444864e-16	Core
C4B63_51g81	1.41737327467439	4.3665742805776e-16	Core
C4B63_5g100	-2.95636287096574	4.59271143839764e-16	Disruptive
C4B63_4g63	-1.91100975084242	4.7618523375495e-16	Core
C4B63_54g93	-1.83328137836321	5.1504308894927e-16	Disruptive
C4B63_19g219	2.19969460815311	5.20591178045915e-16	Core
C4B63_28g322	1.41074508575217	5.20591178045915e-16	Core
C4B63_54g103	-3.24453312944853	5.29307399500608e-16	Disruptive
C4B63_52g163	1.36990531161728	5.40292670173574e-16	Core
C4B63_33g194	-1.71131001516661	5.45860682914304e-16	Disruptive
C4B63_2g229	1.12356737183411	5.60715855489374e-16	Core
C4B63_22g780c	-3.24954588946428	5.61273628227877e-16	Core
C4B63_3g355	-2.95620956821044	6.14464939881519e-16	Disruptive
C4B63_43g86	-3.63910012378822	6.50003612726961e-16	Disruptive
C4B63_70g572c	2.03059177359137	6.8016876578843e-16	Core
C4B63_17g284	1.59187225837907	6.87263525703909e-16	Core
C4B63_47g91	-3.15022464343724	7.07100628808979e-16	Disruptive
C4B63_199g2	1.34430792651969	8.47500241981081e-16	Core
C4B63_8g175	-2.47556147339401	8.65785995721528e-16	Core
C4B63_9g235	1.40476769168198	8.80561224605129e-16	Core
C4B63_47g90	-3.11809708866213	1.0113489258764e-15	Disruptive
C4B63_144g5	-2.92343414364279	1.05109263842941e-15	Disruptive
C4B63_54g24	1.38723822583288	1.06851551697738e-15	Core
C4B63_26g354	2.10707569726666	1.07612349775633e-15	Core
C4B63_70g128	1.563632257006	1.15837231153357e-15	Core
C4B63_23g240	1.25295629795034	1.44803428822894e-15	Core
C4B63_3g819	1.62212329098851	1.45589433044401e-15	Core
C4B63_31g181	1.56492086837827	1.45899205005311e-15	Core

C4B63_19g230	1.98008650690685	1.52259255021912e-15	Core
C4B63_113g62	2.25282926766941	1.58309807307129e-15	Core
C4B63_66g70	-2.39703370164162	1.75575607625978e-15	Disruptive
C4B63_54g101	-2.23938889105586	1.76619931104454e-15	Disruptive
C4B63_53g223	-3.60234462289351	1.81267470722933e-15	Disruptive
C4B63_31g233	-2.47238205299298	1.81795728045865e-15	Disruptive
C4B63_93g6	-2.87544836786595	1.85984527699231e-15	Disruptive
C4B63_44g118	1.00995111566524	2.02520192106979e-15	Core
C4B63_7g428	-1.83818747790076	2.17524942721172e-15	Core
C4B63_8g572	1.08592782763262	2.20970451353982e-15	Core
C4B63_32g181	1.64750026118837	2.21355501371819e-15	Core
C4B63_17g108	-1.07484291059719	2.2840542589121e-15	Core
C4B63_147g50	-1.95666007447662	2.42439389536496e-15	Disruptive
C4B63_19g229	2.08794167437612	2.51028119959619e-15	Core
C4B63_84g82	1.27963654920019	2.55457314876333e-15	Core
C4B63_18g220	-1.18829883650007	2.60603160119649e-15	Core
C4B63_88g114	-2.92984409565238	2.6202559415544e-15	Disruptive
C4B63_219g47	1.47747520044665	2.68778993069369e-15	Core
C4B63_64g186	-3.01250093307187	2.82889192002802e-15	Disruptive
C4B63_22g123	-2.34610090278782	3.3618871799411e-15	Disruptive
C4B63_36g364	-2.59889016607807	3.37202882367846e-15	Disruptive
C4B63_37g382	2.13409224371074	3.37469701850306e-15	Disruptive
C4B63_200g44	-2.32380611030278	3.61229737290102e-15	Core
C4B63_5g689	-3.49705022380994	3.6233456136049e-15	Disruptive
C4B63_225g26	1.36695539744357	3.87052062222683e-15	Core
C4B63_11g230	-3.34928660803677	4.16281488585021e-15	Disruptive
C4B63_258g20	-2.29421237827394	4.16776269757128e-15	Core
C4B63_4g341	-1.36426394594391	4.23402612152434e-15	Core
C4B63_44g227	2.07827128990516	4.24529245187621e-15	Core
C4B63_251g19	3.03765973455396	4.26110972248482e-15	Core
C4B63_3g1134	1.04137173347524	4.33041124657912e-15	Core
C4B63_38g143	1.64795897033631	4.33041124657912e-15	Core
C4B63_12g26	1.15131946493698	4.48848892135598e-15	Core
C4B63_124g48	-3.43984953717134	4.54678003902635e-15	Disruptive
C4B63_258g27	12.3348829716795	5.18279303849363e-15	Core
C4B63_40g176	-2.2393087630999	5.2377884932406e-15	Disruptive
C4B63_49g71	1.83770454171096	5.25905384537933e-15	Core
C4B63_32g138	-3.29085723617795	5.28277679235598e-15	Disruptive
C4B63_27g332	-3.63927416219799	5.28358388671513e-15	Disruptive
C4B63_2g210	1.15191943954438	5.31822240995763e-15	Core
C4B63_34g216	3.35979848468878	5.47585444376763e-15	Disruptive
C4B63_419g4	-2.5126554368812	5.94763367757132e-15	Disruptive
C4B63_153g40	1.7951857452431	6.45448019856947e-15	Core
C4B63_124g18	-2.65546472690482	6.45952469679064e-15	Disruptive
C4B63_11g146	-2.5544627427082	6.4936909574847e-15	Disruptive
C4B63_9g477	-2.8144100971707	6.87861146510629e-15	Disruptive
C4B63_296g22	-2.30568162378199	6.91376938679159e-15	Disruptive
C4B63_141g8	-2.19605607357202	7.09897675483129e-15	Disruptive
C4B63_2g821	1.05725333805319	8.32138686719818e-15	Core
C4B63_8g316	-2.71345551742864	8.57108287890973e-15	Disruptive
C4B63_3g621	-2.95757173716109	8.72413680695594e-15	Disruptive
C4B63_23g130	-1.30949000800464	8.74160744320925e-15	Core
C4B63_54g100	-1.83713601799309	8.85344207621377e-15	Disruptive
C4B63_3g65	-2.91724412801398	9.082465348916e-15	Disruptive
C4B63_26g271	1.54489735086508	9.37833634197895e-15	Core
C4B63_137g3	1.43106866459121	9.75159574369967e-15	Core
C4B63_106g49	-3.31164166063345	1.02843041086203e-14	Disruptive
C4B63_84g22	1.70607347429834	1.12530261817729e-14	Core
C4B63_19g171	1.18210441447348	1.16933652493901e-14	Core
C4B63_37g368	1.32840957002104	1.17908788628911e-14	Core
C4B63_36g100	-2.67954099868276	1.197298797915e-14	Disruptive
C4B63_30g131	1.8163191520431	1.21213195994552e-14	Core
C4B63_159g17	1.67671995955404	1.33055941409585e-14	Core
C4B63_19g211	2.1733177590303	1.40912025821671e-14	Core

C4B63_5g239	-2.88975666632239	1.41006100578859e-14	Disruptive
C4B63_106g40	1.7353793053558	1.46634156128112e-14	Core
C4B63_93g11	-3.86786865181664	1.47889840331638e-14	Disruptive
C4B63_4g188	1.48782216670889	1.53105496901383e-14	Core
C4B63_16g95	1.81076397268241	1.54789097994943e-14	Core
C4B63_81g64	1.4153287685064	1.57952786502836e-14	Core
C4B63_6g2366c	1.27687564847553	1.68633182035785e-14	Core
C4B63_330g9	1.58765934322748	1.68633182035785e-14	Core
C4B63_21g273	1.1251821116733	1.71301509484494e-14	Core
C4B63_8g179	-2.85214686295212	1.72664034206086e-14	Disruptive
C4B63_4g212	1.11031202838196	1.74113899952016e-14	Core
C4B63_3g848	-2.48183560742668	1.75249767220177e-14	Disruptive
C4B63_42g208	-3.37271350926421	1.85051884142158e-14	Core
C4B63_11g338	-3.49083766968283	1.89862667148831e-14	Disruptive
C4B63_6g577	1.21144465244304	1.95564720339778e-14	Core
C4B63_43g3	1.27494086410176	2.06734943119897e-14	Core
C4B63_187g24	-2.56911868405123	2.07210691140855e-14	Disruptive
C4B63_14g92	1.073812729649	2.11606239828241e-14	Core
C4B63_52g135	2.00475925120863	2.15872064243209e-14	Core
C4B63_12g268	-2.22329499655304	2.26716586675475e-14	Disruptive
C4B63_132g12	-3.03544508795531	2.27349681540278e-14	Disruptive
C4B63_50g169	-3.63155697412748	2.4492488203308e-14	Disruptive
C4B63_73g74	1.52285553398629	2.67331772519226e-14	Core
C4B63_249g16	2.31577620040072	2.89505035566483e-14	Core
C4B63_9g472	-1.44709464647995	2.89935133437889e-14	Core
C4B63_27g277	-3.61625792841299	2.9985620170956e-14	Disruptive
C4B63_21g21	1.00764497504392	3.08837820821057e-14	Core
C4B63_30g187	1.73244417967797	3.23441657147632e-14	Core
C4B63_60g104	-2.39010301893831	3.6585414292204e-14	Disruptive
C4B63_4g468	2.03031368837033	3.77452141595205e-14	Core
C4B63_70g63	1.12348138292823	3.81298734712927e-14	Core
C4B63_81g70	13.1966555192339	3.88985089117949e-14	Core
C4B63_198g21	1.48497303263063	4.13871649413288e-14	Core
C4B63_105g68	1.56676413945346	4.1598581598473e-14	Core
C4B63_43g134	1.69245200696937	4.17430802364922e-14	Core
C4B63_65g13	-4.05063605074482	4.21301722563893e-14	Core
C4B63_59g148	1.16085977563428	4.48799160869443e-14	Core
C4B63_1g983	-3.5019573967823	4.62369917523231e-14	Disruptive
C4B63_21g90	1.37530502473467	4.69467719168641e-14	Core
C4B63_26g126	1.35966391294544	4.92715081781959e-14	Core
C4B63_8g86	-2.69090951389021	5.26015924498953e-14	Disruptive
C4B63_277g21	1.78197287132018	5.26015924498953e-14	Core
C4B63_38g171	1.24837964113486	5.5166369314136e-14	Core
C4B63_5g117	-2.62629206992686	5.67806799728053e-14	Disruptive
C4B63_45g139	2.33451032218582	5.69852616095424e-14	Core
C4B63_31g255	-2.03403737796312	5.82608726915398e-14	Disruptive
C4B63_138g27	1.40604180762643	5.86523407865573e-14	Core
C4B63_11g198	-2.75017831532422	6.19089770758486e-14	Disruptive
C4B63_22g153	-2.5014822526218	6.19089770758486e-14	Disruptive
C4B63_51g205	3.04249179776826	6.36357477765987e-14	Core
C4B63_126g31	-3.06863637181744	6.36357477765987e-14	Disruptive
C4B63_6g396	1.30386326681586	6.48402628821905e-14	Core
C4B63_41g153	-1.93550729332534	6.68139585826009e-14	Core
C4B63_107g30	-2.57992305197098	6.72589843218726e-14	Disruptive
C4B63_132g35	-3.59641762812702	6.72845098384982e-14	Disruptive
C4B63_27g301	-3.48628153459854	6.78381612954431e-14	Disruptive
C4B63_17g1078c	1.42987389707291	7.04210577123344e-14	Core
C4B63_15g389	-2.77563593748038	7.12381886109159e-14	Disruptive
C4B63_31g238	-1.98043429040288	7.6265988333116e-14	Disruptive
C4B63_38g192	-1.83831626986628	8.2539909943112e-14	Disruptive
C4B63_27g279	-5.23790712589878	8.30222728881883e-14	Disruptive
C4B63_25g217	1.47864420629298	8.61980970309313e-14	Core
C4B63_51g208	-2.81788024611611	8.78288476104331e-14	Disruptive
C4B63_56g153	-2.27824869014746	8.79673018033143e-14	Disruptive

C4B63_5g752	-3.70959856967032	8.90238764440263e-14	Disruptive
C4B63_5g643	-2.45103837243004	9.01056964945076e-14	Disruptive
C4B63_56g101	1.88805972598299	9.28375033267014e-14	Core
C4B63_69g163	1.06617561409453	1.0133473079368e-13	Core
C4B63_3g455	-2.69058719593425	1.07128424410238e-13	Core
C4B63_111g11	-2.0345068098382	1.11070801210946e-13	Disruptive
C4B63_18g23	-1.25768251035445	1.12671597969551e-13	Core
C4B63_427g3	-3.20234301977373	1.19309791504462e-13	Core
C4B63_241g16	1.54803793938028	1.24606281241453e-13	Core
C4B63_132g24	-2.55057895875381	1.24757024978458e-13	Disruptive
C4B63_16g168	1.59687320328548	1.31302864804052e-13	Core
C4B63_138g18	-1.82180997636861	1.37371069165508e-13	Disruptive
C4B63_396g11	1.97907070377172	1.48199693871558e-13	Core
C4B63_375g42c	1.83632866904021	1.59585668950153e-13	Disruptive
C4B63_38g178	1.94308410044938	1.75160343399106e-13	Core
C4B63_44g132	1.22875410097931	1.75223336786662e-13	Core
C4B63_280g18	1.68063349226899	1.75223336786662e-13	Core
C4B63_9g109	-3.27142629607628	1.84094596957039e-13	Disruptive
C4B63_111g12	-1.73683023866105	1.85452257799397e-13	Disruptive
C4B63_36g128	-2.89799768943905	1.87203663738482e-13	Disruptive
C4B63_66g60	-2.50663234458909	1.92095517463837e-13	Core
C4B63_1g927	-4.17731655417727	2.01945164533658e-13	Disruptive
C4B63_3g964	-3.361185962797	2.03717706970745e-13	Disruptive
C4B63_8g112	-2.52398678935039	2.15258112857499e-13	Disruptive
C4B63_1g1102	-2.56348523106448	2.22154536601761e-13	Disruptive
C4B63_117g59	-2.42401981222884	2.27006270490468e-13	Disruptive
C4B63_111g20	-2.18376463944847	2.27790286295198e-13	Disruptive
C4B63_32g39	1.5677938054228	2.3611413045074e-13	Core
C4B63_28g116	1.61821551187698	2.42320361522854e-13	Core
C4B63_54g81	1.0500343716048	2.42671586128941e-13	Core
C4B63_3g1037	-2.15107846757356	2.46970353777363e-13	Disruptive
C4B63_8g80	-2.46018295914848	2.49961264100172e-13	Disruptive
C4B63_33g309	-3.40463456407178	2.51306298604061e-13	Disruptive
C4B63_271g11	1.60806190473229	2.58433465000388e-13	Core
C4B63_87g42	1.8374562843877	2.76895529250814e-13	Core
C4B63_22g77	1.51873215241229	2.82781587881451e-13	Core
C4B63_68g77	-1.98481897726813	3.01610611929373e-13	Disruptive
C4B63_163g33	2.05622145813234	3.0465175454149e-13	Core
C4B63_5g757	-2.64401519921342	3.06018494511195e-13	Disruptive
C4B63_63g50	1.344143414888	3.07918659618487e-13	Core
C4B63_155g9	-2.50473421909014	3.10615336655025e-13	Disruptive
C4B63_9g474	-2.52837670844668	3.15253605733807e-13	Disruptive
C4B63_10g445	1.86309803388108	3.22350053817984e-13	Core
C4B63_282g14	1.39030938839291	3.25757205076395e-13	Core
C4B63_75g84	-1.78310892522289	3.40189743909106e-13	Disruptive
C4B63_22g96	-1.13146868677998	3.40328874370679e-13	Core
C4B63_1g517	-3.16993359494234	3.53779783443308e-13	Disruptive
C4B63_140g6	1.33182015192465	3.58507372558621e-13	Core
C4B63_3g760	-2.70237864006163	3.60592233661877e-13	Disruptive
C4B63_39g377	-3.16939521545106	3.71590264340478e-13	Disruptive
C4B63_10g439	1.21918546814486	3.77373837405187e-13	Core
C4B63_111g13	-2.70464408298402	3.95367637938738e-13	Disruptive
C4B63_40g116	-1.36976233522731	3.98029487523122e-13	Core
C4B63_2g660	-3.82771567871922	3.98092578663558e-13	Disruptive
C4B63_241g18	1.03987441362857	4.00548286467442e-13	Core
C4B63_35g347	-3.076155242617	4.02524748954277e-13	Disruptive
C4B63_294g12	2.28047326975785	4.16193732846265e-13	Core
C4B63_173g11	-3.02074775363018	4.1627116973624e-13	Disruptive
C4B63_350g4	2.86259979801977	4.1627116973624e-13	Core
C4B63_28g33	1.13501671206764	4.2873939198704e-13	Core
C4B63_29g63	-3.19622686570652	4.36329495469743e-13	Disruptive
C4B63_112g23	1.54219825292553	4.55334368105277e-13	Core
C4B63_3g853	-3.9402630596164	4.55616713367997e-13	Disruptive
C4B63_14g183	1.17684492173148	4.56106328209536e-13	Core

C4B63_60g113	-2.65681981649095	4.59422252443287e-13	Disruptive
C4B63_78g59	-2.16928289144009	4.59422252443287e-13	Disruptive
C4B63_5g91	-2.64979720759011	4.70677434090224e-13	Disruptive
C4B63_14g128	-2.21291362965072	4.84268701045912e-13	Disruptive
C4B63_1g1128	-3.82720064939031	4.86119199316068e-13	Disruptive
C4B63_6g284	2.05314814295742	4.97376005919568e-13	Core
C4B63_45g133	1.22064664695127	5.00649995876272e-13	Core
C4B63_2g711	1.378908408086	5.42192342798256e-13	Core
C4B63_9g272	1.25510309416403	5.50225086192107e-13	Core
C4B63_52g170	1.52922307683357	5.54022042871384e-13	Core
C4B63_24g220	1.17049599420952	6.2852355288899e-13	Core
C4B63_95g34	1.3194082283596	6.58392313934421e-13	Core
C4B63_66g110	-3.22752829069089	6.6440060402595e-13	Disruptive
C4B63_25g220	1.29420187898542	6.84629807028747e-13	Core
C4B63_11g220	1.45058842283771	7.10367002159733e-13	Core
C4B63_39g182	-3.40300013136119	7.1097521865815e-13	Disruptive
C4B63_58g50	1.00551304914059	7.11163955710916e-13	Core
C4B63_72g22	1.65666225736187	7.11163955710916e-13	Core
C4B63_31g133	-2.74448339817166	7.20388285194085e-13	Disruptive
C4B63_62g149	-2.74711894558232	7.25871570656781e-13	Disruptive
C4B63_47g125	-2.05118973951112	7.6192610869264e-13	Disruptive
C4B63_13g287	-2.44724093748666	7.65652389753009e-13	Disruptive
C4B63_53g214	-2.3344766785646	7.67067332959985e-13	Disruptive
C4B63_3g627	-2.90277456302714	7.68500394389857e-13	Disruptive
C4B63_60g109	-2.6084496605686	7.80974530185011e-13	Disruptive
C4B63_62g10	-2.66886480469704	7.94871458497968e-13	Core
C4B63_37g160	-3.90255124428873	8.08946691276661e-13	Disruptive
C4B63_17g115	-1.27505778981942	8.10304100578553e-13	Core
C4B63_76g57	1.0110999962016	8.10304100578553e-13	Core
C4B63_67g98	-3.2269830559969	8.36443820238125e-13	Disruptive
C4B63_19g208	2.00895291145963	8.61366367119671e-13	Core
C4B63_3g577	1.33810939850815	8.74674206894317e-13	Core
C4B63_2g829	1.52565628570684	8.76103572176527e-13	Core
C4B63_16g94	1.37985168129265	9.07902714046495e-13	Core
C4B63_18g151	-1.32251632076719	9.17701496347169e-13	Core
C4B63_1g780	-2.66454330716842	9.44845453802654e-13	Disruptive
C4B63_1g756	-2.00409674895994	9.80972225701593e-13	Core
C4B63_70g95	1.40165722923256	1.01635300137765e-12	Core
C4B63_1g82	-1.8370949691766	1.04219222702655e-12	Disruptive
C4B63_39g312	1.98141822318607	1.06684869008512e-12	Core
C4B63_43g122	1.34858306685991	1.09453122238599e-12	Core
C4B63_60g82	-2.52086806609088	1.15873868129443e-12	Disruptive
C4B63_89g178c	1.16426548445559	1.16165946692769e-12	Core
C4B63_31g257	-1.60765581289614	1.1705245534354e-12	Disruptive
C4B63_303g10	1.15763265648151	1.17591559591256e-12	Core
C4B63_31g263	-2.28445661640838	1.21060491827993e-12	Disruptive
C4B63_65g19	-2.10980655581292	1.21060491827993e-12	Disruptive
C4B63_51g93	1.02176789899523	1.21510702664585e-12	Core
C4B63_387g16	-1.63769198955288	1.22573365535969e-12	Disruptive
C4B63_120g31	-1.6281681904741	1.2903002652821e-12	Disruptive
C4B63_544g7	1.75958749126591	1.36760720039814e-12	Core
C4B63_69g41	-2.05529837476933	1.41551545398282e-12	Disruptive
C4B63_52g63	2.1904968874726	1.43550706920548e-12	Core
C4B63_8g486	1.51941699619205	1.44962148694685e-12	Core
C4B63_197g29	-2.45535885755649	1.50192306434786e-12	Disruptive
C4B63_5g29	-1.94065017592705	1.52088735223655e-12	Core
C4B63_350g3	2.24756491408293	1.5209445630804e-12	Core
C4B63_11g171	1.37975433573254	1.53081578894421e-12	Core
C4B63_128g301c	2.21623372026971	1.55757452163134e-12	Core
C4B63_45g192	1.70330292692663	1.60721183687848e-12	Core
C4B63_64g123	1.41499519434805	1.64836567393939e-12	Core
C4B63_63g33	1.37645025375362	1.68598454792472e-12	Core
C4B63_3g566	-2.63076930773109	1.76736336558893e-12	Disruptive
C4B63_114g35	1.41210315266036	1.86688959223158e-12	Core

C4B63_10g528	1.0998483332408	1.87428943043477e-12	Core
C4B63_70g93	1.29663437092141	1.89610449870396e-12	Core
C4B63_16g142	-2.32265188349065	1.91567534941528e-12	Disruptive
C4B63_113g47	1.14198862281468	1.99744849206277e-12	Core
C4B63_40g178	-2.23875827518163	2.1026744588817e-12	Disruptive
C4B63_126g3	-2.38255284410594	2.10583194785428e-12	Disruptive
C4B63_9g479	-3.20506398623753	2.11889846516106e-12	Disruptive
C4B63_54g26	1.82415936146302	2.13600965973848e-12	Core
C4B63_52g174	-1.19121071815122	2.15208713480888e-12	Core
C4B63_82g94	-1.73485506607401	2.20180571904848e-12	Core
C4B63_94g78	1.55746121491569	2.2124054285355e-12	Core
C4B63_49g176	1.6602676357163	2.22329251093354e-12	Core
C4B63_12g46	-1.59647365012755	2.44747015724741e-12	Disruptive
C4B63_24g365	1.2450554335901	2.45424793709931e-12	Core
C4B63_29g185	1.5824917445328	2.47548480814291e-12	Core
C4B63_121g78	-4.26155994111713	2.48681821790925e-12	Disruptive
C4B63_54g91	-2.11500072281394	2.70043185501384e-12	Disruptive
C4B63_73g70	1.45015407272993	2.75400361365415e-12	Core
C4B63_294g2	3.03571251272539	2.81439426474974e-12	Core
C4B63_52g177	1.15945267245947	2.88859426074491e-12	Core
C4B63_21g284	1.28804384459488	2.89682290456343e-12	Core
C4B63_37g118	-3.99607399173831	2.97735092708056e-12	Disruptive
C4B63_209g8	-1.80460504687864	3.02187584851301e-12	Disruptive
C4B63_3g207	-3.2077104079924	3.06827068421057e-12	Disruptive
C4B63_7g93	1.64784770415224	3.0737714228636e-12	Core
C4B63_44g133	1.03443070775745	3.12176115774847e-12	Core
C4B63_169g32	-2.81296903755262	3.16694494072087e-12	Disruptive
C4B63_3g1104	1.15082126507339	3.20809127800066e-12	Core
C4B63_75g82	-1.89957101512031	3.24368433720879e-12	Disruptive
C4B63_41g221	1.47837050976783	3.33498051572639e-12	Core
C4B63_14g106	1.36856702661224	3.39466893391404e-12	Core
C4B63_28g342	-2.12616291017359	3.43114474167604e-12	Disruptive
C4B63_76g23	2.91109722122719	3.43114474167604e-12	Core
C4B63_1g86	-1.66094921344958	3.53672168473662e-12	Disruptive
C4B63_11g32	1.2007849979488	3.62145680761288e-12	Core
C4B63_10g293	-1.81085804067359	3.72512783489604e-12	Core
C4B63_111g5	-2.63675795566833	3.82279601531442e-12	Disruptive
C4B63_35g353	1.59286889222103	3.98372320703522e-12	Core
C4B63_3g499	-3.78815230839891	4.40592232376889e-12	Disruptive
C4B63_219g34	2.20248745367503	4.51101222154367e-12	Core
C4B63_25g329	1.29711854800394	4.5697180774408e-12	Core
C4B63_6g311	-1.02323159573736	4.7044827220652e-12	Core
C4B63_309g17	-2.50225132890483	4.86627069450403e-12	Disruptive
C4B63_77g10	1.39398512329592	5.1244738241958e-12	Core
C4B63_162g31	-2.63061917524218	5.13018709708683e-12	Core
C4B63_54g84	-2.39069865857179	5.22173907363299e-12	Disruptive
C4B63_47g154	1.07075949066468	5.2819504388252e-12	Core
C4B63_72g33	-3.01640831368081	5.3904356666476e-12	Core
C4B63_11g382	-3.87034917245565	5.81164508859747e-12	Disruptive
C4B63_5g698	-2.98913196934953	5.91106549386263e-12	Disruptive
C4B63_3g844	-3.19139182529875	6.12650082388776e-12	Disruptive
C4B63_26g125	1.18696115834271	6.19358905789482e-12	Core
C4B63_3g496	-3.46753620583509	6.33605501201057e-12	Disruptive
C4B63_9g372	1.8174126964223	6.51500838101135e-12	Core
C4B63_19g200	1.4962496762279	6.79833207421663e-12	Core
C4B63_27g177	1.05851137504064	6.89773251081028e-12	Core
C4B63_5g62	-4.2033236443206	7.24609000871913e-12	Disruptive
C4B63_2g205	2.1908916688784	7.32631045681805e-12	Core
C4B63_37g66	-4.06381806551324	7.39856712474782e-12	Disruptive
C4B63_28g161	1.29180159937377	7.78058991036418e-12	Core
C4B63_36g170	-1.56488627327809	7.97468455885348e-12	Disruptive
C4B63_1g872	-3.29122028563956	7.97688504551495e-12	Disruptive
C4B63_613g1	-2.47360960526821	8.12530488338588e-12	Disruptive
C4B63_29g77	-3.13485658356032	8.3211194298612e-12	Disruptive

C4B63_114g36	1.0660152520106	8.32900709420461e-12	Core
C4B63_183g15	1.09618896390235	8.40192491319918e-12	Core
C4B63_8g64	1.79032187506641	8.45372139214129e-12	Core
C4B63_18g19	1.57072232336361	8.52621886779896e-12	Core
C4B63_68g136	1.3923800047148	8.69114199062061e-12	Core
C4B63_101g28	1.42116498147222	9.24120219527566e-12	Core
C4B63_480g13	-1.86265708735283	9.25849344061281e-12	Core
C4B63_417g23	-4.50920378712559	9.71040390561194e-12	Disruptive
C4B63_128g24	1.61756533704089	9.83448169904719e-12	Core
C4B63_64g142	-1.75552851251275	1.0045761052364e-11	Disruptive
C4B63_10g112	1.38846343393815	1.01084296295964e-11	Core
C4B63_11g93	1.18889523811724	1.02279723943893e-11	Core
C4B63_278g8	2.01265991820235	1.03862284679183e-11	Core
C4B63_93g9	-10.7740158504427	1.04936881944925e-11	Disruptive
C4B63_1g665	-2.24639863889973	1.06938365736276e-11	Disruptive
C4B63_170g15	1.17964287897189	1.13266019042082e-11	Core
C4B63_66g157	-3.53717568548506	1.17535057942387e-11	Disruptive
C4B63_45g184	1.21727456145934	1.17740298819891e-11	Core
C4B63_51g92	2.20731167652667	1.1792340403092e-11	Core
C4B63_106g53	-2.7333902529893	1.1840665899576e-11	Disruptive
C4B63_37g77	-3.02330794116713	1.20922658732641e-11	Disruptive
C4B63_38g94	1.87540768240465	1.20969158349629e-11	Core
C4B63_46g53	1.20621652081674	1.21941846997509e-11	Core
C4B63_22g103	1.20648338879043	1.22306449821357e-11	Core
C4B63_66g18	-3.01477331810115	1.22925297790746e-11	Disruptive
C4B63_45g128	1.99529688765557	1.2341635671491e-11	Core
C4B63_27g266	-2.41375632318489	1.24804438812521e-11	Disruptive
C4B63_20g186	1.12987325069149	1.26896982017813e-11	Core
C4B63_66g145	-3.94085518524272	1.34064112643347e-11	Disruptive
C4B63_219g48	1.25261570544003	1.35166995474965e-11	Core
C4B63_6g113	1.24733028572227	1.38634938823747e-11	Core
C4B63_5g125	-3.14270147483424	1.41523409578139e-11	Disruptive
C4B63_259g12	1.81772421731622	1.44137678361564e-11	Core
C4B63_59g110	-3.44791054171131	1.45990401753193e-11	Disruptive
C4B63_19g112	1.09075854185164	1.50853382470392e-11	Core
C4B63_336g6	2.23286649541498	1.50853382470392e-11	Core
C4B63_114g19	1.55022621608635	1.54339441052987e-11	Core
C4B63_2g197	1.9365286947181	1.56161813948685e-11	Core
C4B63_1g701	-2.56214589753799	1.56809720442336e-11	Disruptive
C4B63_14g119	-2.37567135431375	1.58050805913246e-11	Disruptive
C4B63_459g7	2.43153992838768	1.62285515931767e-11	Core
C4B63_188g42	-1.6384199298617	1.64336405971425e-11	Core
C4B63_34g140	-2.47070171860219	1.64896417272737e-11	Disruptive
C4B63_1g1319	-3.11426314631197	1.66672080869919e-11	Disruptive
C4B63_40g97	1.20307555761412	1.76165666657919e-11	Core
C4B63_1g1025	-3.09746119786191	1.77927388585865e-11	Disruptive
C4B63_54g186	1.06188070571596	1.82412331837382e-11	Core
C4B63_361g15	-1.93653947144489	1.8332374284084e-11	Disruptive
C4B63_1g1004	-2.97441581144063	1.8488411704075e-11	Disruptive
C4B63_17g102	1.75005105829171	1.94274817154829e-11	Core
C4B63_142g4	2.49514069946072	1.96106391372834e-11	Core
C4B63_47g165	1.39039335967621	2.00147373667273e-11	Disruptive
C4B63_1g185	-3.10605658296985	2.0093176741346e-11	Disruptive
C4B63_333g9	2.3132264057795	2.01649187298243e-11	Disruptive
C4B63_130g39	-1.29174017965257	2.0359302959369e-11	Disruptive
C4B63_319g13	-2.28545685558269	2.04429252895483e-11	Disruptive
C4B63_1g1051	-2.86038041703048	2.10300844511426e-11	Disruptive
C4B63_138g113c	1.59989673281277	2.13914352500977e-11	Core
C4B63_108g11	1.3797574952535	2.16233816734012e-11	Core
C4B63_104g74	1.77108883200963	2.1650347088818e-11	Core
C4B63_219g46	1.54603583023551	2.20227450669125e-11	Core
C4B63_59g106	-2.56360813773621	2.22143510830133e-11	Disruptive
C4B63_56g64	1.17145723824329	2.24601930800661e-11	Core
C4B63_26g263	1.37313225027986	2.28079062989006e-11	Core

C4B63_37g98	-3.24801407032168	2.33455491802475e-11	Disruptive
C4B63_54g62	1.79169953090824	2.42981044653108e-11	Core
C4B63_95g6	-1.11713207883745	2.46891361938857e-11	Disruptive
C4B63_27g133	1.29218737985508	2.49474974637903e-11	Core
C4B63_22g175	1.30735874836519	2.64309449117791e-11	Core
C4B63_197g26	-2.10336045794679	2.72753392495936e-11	Disruptive
C4B63_44g208	-1.40653808184161	2.76743767988071e-11	Core
C4B63_28g245	-1.72218014217135	2.88483659938536e-11	Core
C4B63_1g112	-2.61745116160643	3.00360424503137e-11	Disruptive
C4B63_1g1233	-3.11709762158691	3.01077021608574e-11	Disruptive
C4B63_16g331	1.10792829054783	3.08134874257961e-11	Core
C4B63_109g47	-2.17566791226788	3.26805838902457e-11	Disruptive
C4B63_14g5	-1.70990033696232	3.26925456522145e-11	Core
C4B63_238g17	1.84413857222251	3.27963799484448e-11	Core
C4B63_7g227	-1.10509045976204	3.37597082708548e-11	Core
C4B63_19g100	1.08491443057152	3.3997640622906e-11	Core
C4B63_1g200	-2.71770451212764	3.42540071552725e-11	Disruptive
C4B63_3g1002	-3.96049375245039	3.48669009000816e-11	Disruptive
C4B63_39g135	-3.49937420533222	3.57786317860924e-11	Disruptive
C4B63_5g393	-2.43448515967501	3.61623466360707e-11	Disruptive
C4B63_33g64	-3.83965177403095	3.63460862738918e-11	Disruptive
C4B63_53g143	2.54123372255166	3.71521803464492e-11	Core
C4B63_4g347	1.6425992311859	3.81584769640515e-11	Core
C4B63_8g185	-3.0808068983404	3.81584769640515e-11	Disruptive
C4B63_8g133	-2.46886253812691	3.90395553694283e-11	Disruptive
C4B63_20g1412c	1.3651874328007	3.91133223904512e-11	Core
C4B63_152g41	1.56454178296533	4.21026554460271e-11	Core
C4B63_1g1302	-2.58758665296151	4.25991648987647e-11	Disruptive
C4B63_64g140	-2.6619878922271	4.32007442514510e-11	Disruptive
C4B63_88g26	-2.72969977713489	4.37261035579608e-11	Disruptive
C4B63_1g476	2.09764166556638	4.50647879459371e-11	Core
C4B63_2g284	-3.76712426668939	4.56024454000599e-11	Disruptive
C4B63_4g132	1.29290460063043	4.59889961282832e-11	Core
C4B63_3g300	-3.18326739617505	4.6666269062502e-11	Disruptive
C4B63_75g91	-1.68843701476669	4.67439737989452e-11	Disruptive
C4B63_7g118	-1.66069725271461	4.89915128463326e-11	Core
C4B63_5g641	-2.86053736481988	4.95411926419272e-11	Disruptive
C4B63_1g1079	-3.59229052145376	4.96509783126892e-11	Disruptive
C4B63_305g8	-1.98950045297589	5.06193456197209e-11	Disruptive
C4B63_3g791	-2.36296222316764	5.69620317819887e-11	Disruptive
C4B63_19g215	2.14822018948204	5.70237928984308e-11	Core
C4B63_19g91	1.33598041570468	5.70237928984308e-11	Core
C4B63_33g273	-2.65848798716396	5.79998031253793e-11	Disruptive
C4B63_22g162	1.75922268045544	5.92067722928584e-11	Core
C4B63_15g12	1.67702766186366	5.92870945289041e-11	Core
C4B63_1g1095	-3.56581336842464	6.01052198357051e-11	Disruptive
C4B63_20g324	-1.67885775140892	6.02455969889612e-11	Core
C4B63_8g121	-2.68847958892481	6.13330018537703e-11	Disruptive
C4B63_30g219	1.80328154957759	6.19297681255822e-11	Core
C4B63_350g1	1.51181363122313	6.28133406735542e-11	Core
C4B63_7g66	-1.27060253255071	6.43913874962687e-11	Core
C4B63_18g293	1.30404251563974	6.4715280431748e-11	Core
C4B63_104g101	3.00672567740129	6.51235006277808e-11	Core
C4B63_77g15	1.2680260563836	6.54762577795054e-11	Core
C4B63_9g96	-2.8453303402711	6.75807029142496e-11	Disruptive
C4B63_144g17	-2.43354814821566	6.94374114823992e-11	Disruptive
C4B63_19g216	2.3806632936625	7.07459061022794e-11	Core
C4B63_30g261	1.53127858805279	7.07459061022794e-11	Core
C4B63_43g196	1.29340283948198	7.14281695587908e-11	Core
C4B63_40g92	1.13866166313437	7.22134111145927e-11	Core
C4B63_9g100	-2.82018099057976	7.25786352665086e-11	Disruptive
C4B63_14g62	1.86414387021164	7.49344398933359e-11	Core
C4B63_90g102	-3.58680910048406	7.62612182326654e-11	Core
C4B63_52g78	-1.33907600319737	7.75377366564063e-11	Core

C4B63_14g126	-2.29792397231732	8.50654802198235e-11	Disruptive
C4B63_26g50	-2.40902083392058	8.50654802198235e-11	Disruptive
C4B63_37g172	-2.33178118620988	8.56685608900249e-11	Disruptive
C4B63_155g21	-2.91999766008597	8.67586094363327e-11	Disruptive
C4B63_181g25	1.62957145557851	9.23765191196767e-11	Core
C4B63_27g306	-2.52875130850792	9.41533302847181e-11	Disruptive
C4B63_116g18	1.0100171031048	9.41533302847181e-11	Core
C4B63_97g31	1.02316233090827	9.69638848949294e-11	Core
C4B63_65g124	-2.56019948483317	9.88722165531284e-11	Disruptive
C4B63_64g63	1.32195680487669	9.91130756100419e-11	Core
C4B63_56g77	1.25152339691964	1.01812728234674e-10	Core
C4B63_201g20	1.09776338458153	1.02104361342131e-10	Core
C4B63_126g22	-3.02211822633965	1.07108114365254e-10	Disruptive
C4B63_41g245	1.29401624984146	1.11480323416133e-10	Core
C4B63_53g181	-3.81808565444774	1.13243580920651e-10	Disruptive
C4B63_5g90	-3.27825418787085	1.1449053771215e-10	Disruptive
C4B63_14g140	-1.9340222518199	1.16725064892623e-10	Disruptive
C4B63_48g15	2.051302846604	1.18959256680868e-10	Core
C4B63_5g349	-2.24014585754786	1.1939007954575e-10	Disruptive
C4B63_35g178	-1.5434554758179	1.27898115253419e-10	Core
C4B63_10g527	1.09841608998907	1.32363325682196e-10	Core
C4B63_51g126	1.16359734267256	1.37974554156713e-10	Core
C4B63_22g83	1.56808870024043	1.39130426244185e-10	Core
C4B63_66g170	-2.62901532681579	1.41532731280546e-10	Disruptive
C4B63_116g57	-3.3430491542631	1.44585098907717e-10	Disruptive
C4B63_43g160	1.27341192653787	1.45995818346147e-10	Core
C4B63_19g227	1.49049555672062	1.4690418837917e-10	Core
C4B63_10g406	1.02970425685337	1.52075741587005e-10	Core
C4B63_93g25	1.65789067231673	1.58942180980789e-10	Disruptive
C4B63_50g193	-2.57592254385913	1.61806059177329e-10	Disruptive
C4B63_303g6	1.26954705356668	1.63787800781664e-10	Core
C4B63_1g1282	-3.21895335973277	1.67635022474236e-10	Disruptive
C4B63_110g6	1.79885753820178	1.69082382904469e-10	Core
C4B63_86g56	-3.97317633444296	1.80509469706343e-10	Core
C4B63_2g153	-1.41777890470047	1.85366359793708e-10	Core
C4B63_188g152c	2.16648677459133	1.85572229071173e-10	Core
C4B63_1g537	-2.60619172238132	1.85686268387829e-10	Disruptive
C4B63_319g10	-2.02114836133706	1.86467336305606e-10	Disruptive
C4B63_8g101	-2.66979387565544	1.87990372402066e-10	Disruptive
C4B63_37g169	-3.83131181110468	1.89301227733661e-10	Disruptive
C4B63_20g208	1.35589430147023	1.90128932511145e-10	Core
C4B63_56g142	-3.116366131255	1.96388843903189e-10	Disruptive
C4B63_34g284	-1.22593977432269	1.97774087063287e-10	Core
C4B63_1g1045	-3.14538227285698	1.98593989810549e-10	Disruptive
C4B63_23g17	2.1419560980999	2.04037210645343e-10	Core
C4B63_26g272	1.72911505028256	2.06410897523375e-10	Core
C4B63_15g379	-2.98612445687948	2.06844519218772e-10	Disruptive
C4B63_397g11	1.33163299991342	2.07104316044165e-10	Core
C4B63_52g143	1.20487992872446	2.12343897697422e-10	Core
C4B63_22g78	-1.43106704150977	2.17449814014366e-10	Core
C4B63_35g148	-1.72064977728975	2.26292801164864e-10	Disruptive
C4B63_57g38	-2.00697128282141	2.2919855804108e-10	Disruptive
C4B63_343g13	-2.64293653984989	2.3562982806341e-10	Disruptive
C4B63_13g978c	1.30506417864682	2.37431837929722e-10	Core
C4B63_387g12	-9.75956265267122	2.43643677571993e-10	Disruptive
C4B63_1g491	-3.54819806697722	2.47437130027839e-10	Disruptive
C4B63_91g63	-2.78851275065089	2.49111866057261e-10	Disruptive
C4B63_45g99	1.44979498927865	2.5281068638902e-10	Core
C4B63_222g5	-2.45498167491324	2.52882870323496e-10	Disruptive
C4B63_117g67	-2.75207169349683	2.61309217061609e-10	Disruptive
C4B63_19g184	1.36271123332741	2.74643508182904e-10	Core
C4B63_6g412	1.91998102837889	2.95956959993745e-10	Core
C4B63_33g163	1.55811666768119	3.01954568068641e-10	Disruptive
C4B63_24g236	2.01734749355961	3.03205655464239e-10	Core

C4B63_116g34	-2.95378904179923	3.09449823698848e-10	Disruptive
C4B63_66g12	1.32916020833522	3.12568831595756e-10	Core
C4B63_106g72	-2.66976233398998	3.13594624910233e-10	Disruptive
C4B63_18g279	1.03960246027627	3.14987258287493e-10	Core
C4B63_386g23	-2.54929481693855	3.19233920310372e-10	Disruptive
C4B63_12g197	1.34757730005075	3.19496078024009e-10	Core
C4B63_39g238	-3.9364316407508	3.22874101781919e-10	Disruptive
C4B63_50g132	-3.35533480396986	3.26393674873258e-10	Disruptive
C4B63_5g135	-3.47145432197158	3.33907936231012e-10	Disruptive
C4B63_51g222	-2.1362890750867	3.35470141513384e-10	Disruptive
C4B63_34g239	3.49804601504038	3.37919140600711e-10	Disruptive
C4B63_12g334	1.4594481535119	3.39154177466465e-10	Core
C4B63_299g9	-2.77929170993845	3.39490323876586e-10	Disruptive
C4B63_51g225	-2.240310318624	3.43114891166095e-10	Disruptive
C4B63_33g5	1.85242051629818	3.43382886931917e-10	Core
C4B63_116g4	1.11562862582688	3.45313883357723e-10	Core
C4B63_36g183	-1.92339770047304	3.51443869641646e-10	Disruptive
C4B63_127g37	1.3520886340555	3.58559299994337e-10	Core
C4B63_24g344	-1.69332105647367	3.58902602086633e-10	Disruptive
C4B63_8g93	-2.24760717514657	3.58940899199856e-10	Disruptive
C4B63_57g83	1.06633009970053	3.62614215133104e-10	Core
C4B63_8g100	-3.04974746548947	3.64287596105923e-10	Disruptive
C4B63_58g132	1.57696054715328	3.66532107137014e-10	Core
C4B63_165g18	1.1410984893947	3.66532107137014e-10	Core
C4B63_41g246	1.18543861901597	3.69812924477171e-10	Core
C4B63_78g31	1.12924085397884	3.70902203377522e-10	Core
C4B63_19g27	1.80578371665646	3.71583247075969e-10	Core
C4B63_46g10	-2.03235908248359	3.80707737072728e-10	Core
C4B63_66g146	-2.71234146160866	3.83549632088201e-10	Disruptive
C4B63_1g94	-1.52136494527523	3.84018986279962e-10	Disruptive
C4B63_4g502	1.23043097388798	3.90125346039397e-10	Core
C4B63_1g300	-1.9494868562067	3.94865309653338e-10	Disruptive
C4B63_116g36	1.20230265856587	3.98632671113081e-10	Core
C4B63_40g72	1.32799640157634	4.06759233488844e-10	Core
C4B63_65g113	-3.5262310860094	4.10473927297426e-10	Disruptive
C4B63_7g105	1.07156417888858	4.13976805402424e-10	Core
C4B63_18g213	1.0349538129545	4.18307510416842e-10	Core
C4B63_13g199	-1.19005002417243	4.23709043455764e-10	Core
C4B63_5g506	-2.88521275857864	4.31867827922773e-10	Disruptive
C4B63_191g7	1.3389417504938	4.39810472644651e-10	Core
C4B63_353g3	1.08053130780915	4.41257210232231e-10	Core
C4B63_13g325	1.25752304570621	4.41795451447443e-10	Core
C4B63_15g484	-2.43042357914514	4.46013049583064e-10	Disruptive
C4B63_15g79	-3.27218821204973	4.51480894007684e-10	Disruptive
C4B63_3g237	-3.22880622077776	4.59781551731238e-10	Disruptive
C4B63_255g15	1.72772213751775	4.6759732207495e-10	Core
C4B63_15g308	-2.76523656582617	4.68157881225442e-10	Disruptive
C4B63_1g629	-3.12921133558437	4.93865141360062e-10	Disruptive
C4B63_211g37	2.06953401233139	4.99254194780866e-10	Core
C4B63_94g80	-2.47391239841064	5.24244294196096e-10	Disruptive
C4B63_251g8	-2.04627847659011	5.29798066582451e-10	Core
C4B63_8g92	-2.83010642408257	5.36902620612097e-10	Disruptive
C4B63_33g298	1.20749719219928	5.41478197795597e-10	Core
C4B63_83g37	-1.60294941989114	5.56613461845304e-10	Disruptive
C4B63_3g567	-2.61424218783701	5.64933026786533e-10	Disruptive
C4B63_41g123	-1.62433763345941	5.65084358741437e-10	Core
C4B63_11g25	-1.42925346402579	5.68544685489898e-10	Core
C4B63_2g744	-1.00110060722193	5.71713740159234e-10	Core
C4B63_216g12	-3.24907827846874	5.86245397364151e-10	Core
C4B63_136g24	-1.7380682391374	6.15926669547554e-10	Disruptive
C4B63_142g27	1.2844055102774	6.25488577277744e-10	Core
C4B63_29g133	-2.3710828815882	6.43448041283195e-10	Disruptive
C4B63_341g15	-5.21220465054381	6.53786571186811e-10	Core
C4B63_60g89	-1.81274188432131	6.60181632943907e-10	Disruptive

C4B63_17g320	1.14975991309247	6.67824262842998e-10	Core
C4B63_27g168	1.0056197749256	6.79306820333491e-10	Core
C4B63_29g450	-2.42097638117896	6.85563270944912e-10	Disruptive
C4B63_180g9	-1.35827645968463	6.89005834977106e-10	Disruptive
C4B63_37g159	-3.57695775528039	6.96563341288979e-10	Disruptive
C4B63_30g290	-1.57715348330276	7.01230310608988e-10	Core
C4B63_42g42	1.25094752968231	7.33391993821715e-10	Core
C4B63_51g217	-1.7417577143151	7.6204127163272e-10	Disruptive
C4B63_16g290	1.28124884042254	7.85870377376538e-10	Core
C4B63_7g209	1.93424762434997	8.04828992797505e-10	Core
C4B63_23g1438c	1.76896097753654	8.04828992797505e-10	Core
C4B63_39g88	-2.53522888284991	8.04828992797505e-10	Disruptive
C4B63_14g138	-2.2540397777436	8.11183716188139e-10	Disruptive
C4B63_201g2	-3.92392681851677	8.25762626496655e-10	Disruptive
C4B63_23g98	-1.63617274348906	8.41389204948526e-10	Core
C4B63_294g8	2.01320047429869	8.80837601954564e-10	Core
C4B63_238g16	1.83733816160333	8.95711323077033e-10	Core
C4B63_35g109	1.55798204870201	9.23501800483661e-10	Core
C4B63_45g132	1.4319128159724	9.28430145611245e-10	Core
C4B63_12g201	1.03633014492256	9.30236086027798e-10	Core
C4B63_23g132	-1.56238084602541	9.40803097358553e-10	Core
C4B63_33g184	1.20424324427643	9.49002506600726e-10	Core
C4B63_180g11	-1.60005697242381	9.53622368204143e-10	Disruptive
C4B63_131g43	1.03995409669465	9.70260408437134e-10	Core
C4B63_46g96	1.90152458261587	9.82364551700292e-10	Core
C4B63_29g13	-4.4189909442725	9.83582837348557e-10	Disruptive
C4B63_13g193	1.32121641630405	9.89902295470856e-10	Core
C4B63_65g60	-3.9724348168952	1.00177061089127e-09	Disruptive
C4B63_104g94	-1.83644202712105	1.00679276401974e-09	Core
C4B63_12g345	1.09743400553108	1.02792983900665e-09	Core
C4B63_5g308	-2.74790698440458	1.03905302967417e-09	Disruptive
C4B63_93g39	-1.96155296035576	1.05583407203381e-09	Core
C4B63_28g168	1.09225534386002	1.066990744211e-09	Core
C4B63_9g515	1.49979330603568	1.11266671056755e-09	Core
C4B63_35g364	1.2906512439927	1.14559036876465e-09	Core
C4B63_54g35	1.28241143088895	1.14893477023829e-09	Core
C4B63_43g161	1.12527985535305	1.1676206213948e-09	Core
C4B63_2g725	-1.27140985758224	1.1701158504393e-09	Core
C4B63_218g26	1.38738195630105	1.1701158504393e-09	Core
C4B63_2g412	2.051411658269	1.27463929313769e-09	Core
C4B63_15g415	-2.29123963730457	1.29715499044538e-09	Disruptive
C4B63_5g34	-2.2215650311258	1.31524020863324e-09	Disruptive
C4B63_13g25	-1.78183386268084	1.31885898053095e-09	Disruptive
C4B63_86g61	-2.6873262594454	1.32922915566993e-09	Disruptive
C4B63_180g12	1.02750607517433	1.40486934452782e-09	Core
C4B63_68g98	-2.0674599453618	1.40712539786193e-09	Disruptive
C4B63_36g177	-3.04789340833408	1.42025329152135e-09	Disruptive
C4B63_142g26	1.51340253804541	1.47531960104535e-09	Core
C4B63_42g103	1.53006651687489	1.49338361512563e-09	Core
C4B63_118g25	-3.21983853281725	1.52473051133319e-09	Disruptive
C4B63_3g681	-3.6454631266855	1.55909247240478e-09	Disruptive
C4B63_98g8	1.69903192885056	1.56847547824094e-09	Disruptive
C4B63_1g957	-2.96702357263416	1.60423096354314e-09	Disruptive
C4B63_57g42	-3.27491448554635	1.60423096354314e-09	Disruptive
C4B63_27g327	-2.52228187081998	1.60676512101316e-09	Disruptive
C4B63_29g141	-2.49097855565078	1.70104364740087e-09	Disruptive
C4B63_24g313	1.43701604937182	1.74495375203858e-09	Core
C4B63_131g53	1.06282019730307	1.79321716119708e-09	Core
C4B63_15g156	-2.61084764440309	1.85493498784323e-09	Disruptive
C4B63_148g27	-9.32700080100086	1.88931494240316e-09	Core
C4B63_19g210	3.55037345002394	1.90348944493328e-09	Core
C4B63_3g870	-2.72355346017104	1.95586551154359e-09	Disruptive
C4B63_96g90	-5.74754585739973	2.08996644796391e-09	Disruptive
C4B63_3g724	-2.68296672886084	2.23095295794039e-09	Disruptive

C4B63_141g14	1.19382942618227	2.23744020131645e-09	Core
C4B63_15g356	1.29932133285273	2.25597441361959e-09	Core
C4B63_24g287	-2.87462080768677	2.314506434207e-09	Disruptive
C4B63_59g213	-3.06836471618207	2.34045142268481e-09	Disruptive
C4B63_5g142	-3.11446002541425	2.34459240415854e-09	Disruptive
C4B63_170g16	1.35707551788597	2.37927600185274e-09	Core
C4B63_24g356	1.40172689609652	2.40556667312004e-09	Core
C4B63_44g191	1.13582942510452	2.4685440379788e-09	Core
C4B63_8g495	1.12328429808624	2.47897204326595e-09	Core
C4B63_15g223	-3.16581577597407	2.49807254865757e-09	Disruptive
C4B63_19g101	1.14264305700875	2.50953986251373e-09	Core
C4B63_107g46	-5.67345114514576	2.60081685315462e-09	Disruptive
C4B63_73g54	1.16433277062512	2.61973690852098e-09	Core
C4B63_25g253	1.1620881573375	2.62147981363329e-09	Core
C4B63_253g16	1.26838938770514	2.64464532107198e-09	Core
C4B63_135g30	1.08781750926227	2.6909876000388e-09	Core
C4B63_128g59	1.64155504167213	2.73895666631356e-09	Core
C4B63_59g224	-2.27548917033869	2.79605433502704e-09	Disruptive
C4B63_33g68	-2.84112826070405	2.83426773301017e-09	Disruptive
C4B63_310g9	-2.04964548574983	2.96186165541423e-09	Disruptive
C4B63_23g223	-1.04676897464441	3.0324652546644e-09	Core
C4B63_74g55	-1.72088977109598	3.05871689560803e-09	Disruptive
C4B63_120g23	-4.80381656990731	3.07147212977879e-09	Disruptive
C4B63_5g367	-2.28345116774356	3.12073471793275e-09	Disruptive
C4B63_2g633	-3.17488356123787	3.35118338308255e-09	Disruptive
C4B63_60g77	2.09021299789659	3.35118338308255e-09	Core
C4B63_60g198	1.59081835950514	3.38134536751654e-09	Core
C4B63_37g415	-3.60163927708694	3.42509331808019e-09	Disruptive
C4B63_427g5	1.37106822109355	3.4882934333881e-09	Core
C4B63_47g34	1.8251091319883	3.49923008169253e-09	Core
C4B63_323g5	-2.28844601762799	3.50181493107146e-09	Disruptive
C4B63_11g305c	1.0124623768658	3.51101409844098e-09	Core
C4B63_196g44	-2.20429696700765	3.56125670310603e-09	Disruptive
C4B63_1g1134	-3.00428294202619	3.62953488280918e-09	Disruptive
C4B63_9g333	1.59824308172713	3.66491586458148e-09	Core
C4B63_40g110	1.83458365380025	3.68082205076044e-09	Core
C4B63_24g203	1.88667504528303	3.81851791205063e-09	Core
C4B63_29g48	-4.41456312502345	3.88972632764133e-09	Disruptive
C4B63_5g398	-2.0006658382887	4.03543195724293e-09	Disruptive
C4B63_5g215	-2.08956564130432	4.12227188392388e-09	Disruptive
C4B63_251g4	-1.82817419577683	4.22621634307367e-09	Core
C4B63_5g39	-3.07587406037741	4.25063925380841e-09	Disruptive
C4B63_139g23	-3.38792522528944	4.25063925380841e-09	Disruptive
C4B63_4g252	1.58017601277983	4.27281789925815e-09	Core
C4B63_2g783	1.30388304822185	4.31673155222208e-09	Core
C4B63_40g177	-9.74695794925869	4.3218226845371e-09	Disruptive
C4B63_67g29	-2.51476819717202	4.39920676120961e-09	Disruptive
C4B63_358g15	2.10631797717933	4.43277469299078e-09	Core
C4B63_134g44	-1.55254127547297	4.64133107056519e-09	Disruptive
C4B63_3g847	-3.0231688980831	4.87884755976265e-09	Disruptive
C4B63_51g213	-1.51730515226785	4.99699545879471e-09	Disruptive
C4B63_27g265	-2.95213715688204	5.05555547189025e-09	Disruptive
C4B63_95g64	-2.09332542923427	5.19342641591786e-09	Core
C4B63_3g774	-2.20328523322421	5.53624683033431e-09	Disruptive
C4B63_23g189	-1.48187961858546	5.59706003703705e-09	Core
C4B63_104g99	2.65704608399982	5.70077005160855e-09	Core
C4B63_15g86	-2.86257476454119	5.79766572217998e-09	Disruptive
C4B63_2g4104c	2.27198120222	5.90959836271664e-09	Core
C4B63_5g210	-2.58195334710206	5.96359023395544e-09	Disruptive
C4B63_151g48	1.16623728037192	6.09635785853007e-09	Core
C4B63_11g371	-2.58514412113995	6.11143845278918e-09	Disruptive
C4B63_223g15	-2.98180679025762	6.11461893706072e-09	Disruptive
C4B63_78g55	1.14144987823855	6.26729788947391e-09	Core
C4B63_12g158	2.30315580358677	6.90235527690706e-09	Core

C4B63_44g179	1.09198703941511	7.02215247312426e-09	Core
C4B63_320g3	-3.22438701220914	7.3187426957965e-09	Disruptive
C4B63_43g74	1.2983313481347	7.36937562881009e-09	Core
C4B63_2g23	-1.0594209881405	7.41982285859886e-09	Core
C4B63_5g419	1.98058524900217	7.53039901254e-09	Core
C4B63_38g307	1.16529807941705	7.6603724592185e-09	Core
C4B63_34g1240c	3.18179695859516	7.81963680102794e-09	Disruptive
C4B63_90g81	-2.4975051255201	7.81963680102794e-09	Disruptive
C4B63_58g126	2.07015892741469	7.8587907338557e-09	Core
C4B63_115g9	-3.01624418726299	8.01040456547968e-09	Disruptive
C4B63_23g241	1.02074885812323	8.0116389439724e-09	Core
C4B63_49g107	-1.04330548368754	8.13346662793997e-09	Core
C4B63_403g6	1.10278171942417	8.44432229987703e-09	Core
C4B63_5g486	-5.66751456736338	8.55768892742331e-09	Disruptive
C4B63_2g450	1.09600173084078	8.90090177942699e-09	Core
C4B63_296g27	-1.98207587247862	8.93871168396161e-09	Disruptive
C4B63_68g97	-2.27484490531231	9.5015253191368e-09	Disruptive
C4B63_1g1255	-2.34914499044844	9.52644993278384e-09	Disruptive
C4B63_32g322	1.15111271295121	1.00985043694784e-08	Core
C4B63_71g171	-3.03403840595509	1.01747434809924e-08	Disruptive
C4B63_37g151	-3.08076600079331	1.02052881699319e-08	Disruptive
C4B63_18g152	-1.48775248406866	1.03931102516911e-08	Core
C4B63_15g224	-2.02957995788086	1.04271984591113e-08	Disruptive
C4B63_29g59	-2.92744190898145	1.04528696272399e-08	Disruptive
C4B63_76g31	1.00234555297815	1.07169429397968e-08	Core
C4B63_39g110	-2.43395391939245	1.07748521270887e-08	Disruptive
C4B63_42g202	1.25411129929642	1.07963460592247e-08	Core
C4B63_51g104	1.40008329639427	1.10675603795764e-08	Core
C4B63_167g34	1.11099980193	1.16026111394185e-08	Core
C4B63_1g1238	-2.83315381034858	1.16394282001813e-08	Disruptive
C4B63_15g269	-2.06197735713064	1.21065807078303e-08	Disruptive
C4B63_4g95	1.02771630824536	1.21546868678952e-08	Core
C4B63_26g280	1.49443151358296	1.21593908698692e-08	Core
C4B63_29g283	-2.23845381906112	1.22063053144043e-08	Disruptive
C4B63_129g31	2.33693703658752	1.2405343505745e-08	Disruptive
C4B63_40g19	1.94869042328904	1.24361788201831e-08	Core
C4B63_256g19	1.16251685727514	1.24924649635194e-08	Core
C4B63_29g356	-3.13727170905733	1.26125811167296e-08	Disruptive
C4B63_277g17	2.86504761726541	1.26317000941099e-08	Core
C4B63_86g75	1.34211277201119	1.27869135485481e-08	Core
C4B63_50g145	-3.16203001727342	1.28693400576697e-08	Disruptive
C4B63_15g133	-1.92341111282735	1.34089720902331e-08	Disruptive
C4B63_143g20	-1.40457049865661	1.3809775093636e-08	Core
C4B63_32g326	1.17166054037468	1.38572269644521e-08	Core
C4B63_205g30	-2.13536504728414	1.38815837249462e-08	Core
C4B63_70g52	2.96486717464602	1.38976517593934e-08	Core
C4B63_5g43	-2.25531756356106	1.39788656976826e-08	Disruptive
C4B63_5g787	-1.18265048398959	1.43844777247719e-08	Disruptive
C4B63_41g241	1.06290755360111	1.45842535783905e-08	Core
C4B63_44g163	-1.41424578533015	1.49751572420493e-08	Core
C4B63_142g14	1.22404386589336	1.52284694425652e-08	Core
C4B63_44g158	-1.77846638881905	1.56818386639606e-08	Core
C4B63_71g189	-3.16531718117674	1.66896628903049e-08	Disruptive
C4B63_60g159	1.43761194390013	1.69647225674983e-08	Core
C4B63_615g5	2.75870190245728	1.71814223893033e-08	Core
C4B63_131g50	-2.15844512155681	1.83056671354012e-08	Disruptive
C4B63_37g383	1.38080332281405	1.85935499000371e-08	Disruptive
C4B63_32g209	1.74151023190899	1.88939466208035e-08	Core
C4B63_44g123	1.19405740925339	1.90911908069861e-08	Core
C4B63_6g267	1.85808588163928	1.91885093596507e-08	Core
C4B63_121g35	-2.32841652699391	1.95302353422533e-08	Disruptive
C4B63_224g19	-2.00130527152249	2.11843603757504e-08	Core
C4B63_274g9	1.39928405839135	2.17919584675679e-08	Core
C4B63_88g23	-3.14987269975755	2.19245719179551e-08	Disruptive

C4B63_1g997	-2.64962942443082	2.19390169717736e-08	Disruptive
C4B63_241g24	1.10393687480291	2.23254338963035e-08	Core
C4B63_147g55	-2.06693201588606	2.2682436594844e-08	Disruptive
C4B63_97g37	1.30832114035897	2.29890753528569e-08	Core
C4B63_21g285	1.42816051299479	2.31719145581089e-08	Core
C4B63_242g10	1.66407761435583	2.43856027582887e-08	Core
C4B63_422g11	2.25071370278925	2.4670577053468e-08	Core
C4B63_15g33	-2.79212291082775	2.50691040687077e-08	Disruptive
C4B63_1g1265	-2.57117711424552	2.54620553739754e-08	Disruptive
C4B63_141g33	-2.57199696964385	2.56623777437576e-08	Disruptive
C4B63_9g518	-1.00976751829066	2.57606480395291e-08	Core
C4B63_74g44	-3.20467083398054	2.57606480395291e-08	Disruptive
C4B63_33g61	-3.08168154124812	2.59570665411704e-08	Disruptive
C4B63_132g15	-2.87673625681953	2.64677260917083e-08	Disruptive
C4B63_319g9	-1.38570410159018	2.64677260917083e-08	Disruptive
C4B63_61g127	1.65455456916253	2.64819683084112e-08	Core
C4B63_87g5	-1.19268119064625	2.64819683084112e-08	Core
C4B63_34g333	2.20365965689747	2.68787465810976e-08	Disruptive
C4B63_3g1033	-2.41106773723563	2.71480697563751e-08	Disruptive
C4B63_29g448	-2.00945423528767	2.71578792005385e-08	Disruptive
C4B63_6g129	-9.02085281858984	2.73780655795242e-08	Core
C4B63_11g426	-2.2779165401898	2.80185410855148e-08	Disruptive
C4B63_431g7	1.23174082274549	2.80630786555485e-08	Core
C4B63_38g92	1.04620364756855	2.82843968845606e-08	Core
C4B63_5g317	-3.15079085381062	2.86563946980946e-08	Disruptive
C4B63_201g1	-3.06968151687677	2.86835011241387e-08	Disruptive
C4B63_46g95	1.97534451236595	2.88746038929104e-08	Core
C4B63_29g56	-1.97937861914824	2.92854539159403e-08	Disruptive
C4B63_1g44	-1.57921793781442	2.9565442112979e-08	Core
C4B63_39g137	-2.43173965903105	2.99987395716705e-08	Disruptive
C4B63_277g3	1.75778082295702	3.07044433222763e-08	Core
C4B63_97g35	-8.80306584120433	3.08204309107033e-08	Core
C4B63_1g839	-2.50089151013238	3.11375583958107e-08	Disruptive
C4B63_56g98	1.39602554423143	3.11375583958107e-08	Core
C4B63_157g30	1.38369486902998	3.13278712461261e-08	Core
C4B63_386g12	-2.86482007760093	3.23138645727661e-08	Disruptive
C4B63_132g13	-3.16212159045605	3.37594353209166e-08	Disruptive
C4B63_24g199	-1.12128728166394	3.42465837783706e-08	Core
C4B63_40g171	-1.75446966331248	3.42745387327257e-08	Disruptive
C4B63_5g372	-2.5923262232084	3.55567283841633e-08	Disruptive
C4B63_402g10	1.28895038797214	3.5784849543304e-08	Core
C4B63_10g161	-1.10437574161129	3.60341820217417e-08	Core
C4B63_36g184	-2.40510461150373	3.6579549743267e-08	Disruptive
C4B63_118g24	-2.73701660717795	3.80971876366768e-08	Disruptive
C4B63_48g29	2.18480073115927	3.8470131690136e-08	Core
C4B63_266g12	-8.85810039180283	3.87919179160717e-08	Core
C4B63_4g242	-1.27743777233702	3.91576580099559e-08	Core
C4B63_98g33	-2.11422266009182	3.98650653102266e-08	Disruptive
C4B63_8g168	-2.17392678762126	4.03819318084469e-08	Disruptive
C4B63_15g385	1.40570055162632	4.06497387265552e-08	Core
C4B63_51g237	-1.20841620067029	4.08395500025787e-08	Disruptive
C4B63_4g475	1.09969550511621	4.19139901756398e-08	Core
C4B63_2g174	1.04259679383106	4.19544724468396e-08	Core
C4B63_9g494	1.13814826338769	4.25618383823502e-08	Core
C4B63_1g406	-3.19319297046154	4.31290852916907e-08	Disruptive
C4B63_2g648	1.26866816697462	4.38289919181128e-08	Core
C4B63_62g182	-3.12664459695979	4.38289919181128e-08	Disruptive
C4B63_66g71	-2.21726357292298	4.43242635843152e-08	Disruptive
C4B63_31g228	1.47209664900563	4.45669230777748e-08	Core
C4B63_61g147	1.50856194313427	4.51802363546667e-08	Core
C4B63_27g321	-2.85415249016535	4.5595506812938e-08	Disruptive
C4B63_3g560	-2.42339776954548	4.721536479203e-08	Disruptive
C4B63_36g230	-2.53109432109369	4.95388784293733e-08	Disruptive
C4B63_66g52	-8.79660577818171	4.96831475964626e-08	Disruptive

C4B63_46g78	-1.42297474931596	5.04757918162285e-08	Core
C4B63_3g779	-2.5526879274357	5.13518842340338e-08	Disruptive
C4B63_8g2652c	2.31425912484296	5.28915662174962e-08	Core
C4B63_64g132	-3.71383621031713	5.35191430103351e-08	Disruptive
C4B63_29g21	-5.31034532469306	5.49207188046398e-08	Disruptive
C4B63_5g692	-2.45496014846114	5.54460115308346e-08	Disruptive
C4B63_87g29	1.68353029946057	5.5702198638708e-08	Core
C4B63_62g102	-2.0644505723725	5.57143252182272e-08	Disruptive
C4B63_10g530	1.40839031787744	5.60434442115156e-08	Core
C4B63_37g114	-2.36834507851061	5.75633757327729e-08	Disruptive
C4B63_43g84	1.49005792211342	5.7948889127275e-08	Core
C4B63_314g13	1.42875911380116	5.92841439178725e-08	Core
C4B63_230g12	1.06161341010498	5.96140726337503e-08	Core
C4B63_1g676	-2.90662077750476	6.05157115246092e-08	Disruptive
C4B63_23g37	-1.07708037102297	6.12234447255321e-08	Core
C4B63_79g2	-1.79243082956185	6.33668912714361e-08	Core
C4B63_156g11	1.29973555405773	6.36120486140664e-08	Core
C4B63_37g188	-3.06470393939972	6.45634914187501e-08	Disruptive
C4B63_136g25	-1.68753116184936	6.46534083640015e-08	Disruptive
C4B63_27g204	1.49873536740406	6.50083644315799e-08	Core
C4B63_29g16	-2.73073658845254	6.50774228754134e-08	Disruptive
C4B63_59g129	1.41308866666304	6.54812891371223e-08	Core
C4B63_181g4	1.27838553586761	6.67278559919728e-08	Core
C4B63_32g40	1.42176741851357	6.67540349107741e-08	Core
C4B63_3g769	-2.27731998479407	6.7645002528598e-08	Disruptive
C4B63_36g149	-2.67699019570188	6.92367825149231e-08	Disruptive
C4B63_62g175	-2.99309343965999	6.94795336923774e-08	Disruptive
C4B63_85g48	1.42035876541717	6.97437601574084e-08	Core
C4B63_119g39	1.07994668692618	6.98969429985918e-08	Core
C4B63_10g458	1.11422261869806	7.01964944229794e-08	Core
C4B63_1g1261	-2.34054953616033	7.1407959535743e-08	Core
C4B63_41g231	-1.16656767373914	7.18128359056385e-08	Core
C4B63_403g4	1.23016424694332	7.24798940520931e-08	Core
C4B63_44g125	1.23171438215715	7.43585502405552e-08	Core
C4B63_64g79	-2.31387099755812	7.58908026039659e-08	Disruptive
C4B63_37g401	1.11723866085503	7.68138881360399e-08	Core
C4B63_20g161	1.64889721553177	7.69017710650333e-08	Core
C4B63_50g142	-2.62724836089747	7.73666252353356e-08	Disruptive
C4B63_41g152	-1.95578525026335	7.85134891191768e-08	Core
C4B63_104g100	2.40492706488267	7.89806169202752e-08	Core
C4B63_29g366	-2.88476020806105	7.89909808116987e-08	Disruptive
C4B63_32g53	1.75586888700761	8.05625255041774e-08	Core
C4B63_109g88	2.38519124906951	8.11415163963339e-08	Disruptive
C4B63_178g34	1.45400794853555	8.15970932427519e-08	Disruptive
C4B63_10g474	-1.01403417067218	8.4807161923146e-08	Core
C4B63_3g991	1.79892198101628	8.52384068867595e-08	Core
C4B63_196g41	-1.64276986401519	8.59822899799437e-08	Disruptive
C4B63_37g122	-2.18715285477248	8.68334423649777e-08	Disruptive
C4B63_93g60	-1.48838731790604	8.77362952513188e-08	Core
C4B63_6g611	-2.02522757085388	8.78269741732173e-08	Disruptive
C4B63_50g127	-2.61503253030863	8.79786045928692e-08	Disruptive
C4B63_23g18	1.13354625607893	8.81177155803916e-08	Core
C4B63_55g190	1.03766431218655	9.00092916504734e-08	Core
C4B63_91g39	1.25205670378621	9.07396774345201e-08	Core
C4B63_64g195	-3.3123557561688	9.08747583018935e-08	Disruptive
C4B63_25g296	1.14611380854135	9.16856129018685e-08	Core
C4B63_470g6	-8.86388149791064	9.16856129018685e-08	Core
C4B63_240g8	-2.56864846895409	9.20505864143408e-08	Disruptive
C4B63_13g276	1.21431311747048	9.2550725696087e-08	Core
C4B63_55g66	1.09240353724579	9.25796382737446e-08	Core
C4B63_48g14	1.48874041502382	9.28782846487982e-08	Core
C4B63_259g10	1.15391431402131	9.3231006852741e-08	Core
C4B63_19g207	1.49182099152297	9.39929627501452e-08	Core
C4B63_9g459	1.24681059076762	9.51641928855676e-08	Core

C4B63_69g34	-2.40342058019982	9.55051084357754e-08	Disruptive
C4B63_11g177	-2.57251033685407	9.60749426168357e-08	Disruptive
C4B63_7g332	-1.0903957817209	9.92427366698461e-08	Core
C4B63_73g45	1.20644988372092	9.9461933925906e-08	Core
C4B63_49g116	1.21261263078748	1.01397317983676e-07	Core
C4B63_3g575	-2.28578377448768	1.03257697953793e-07	Disruptive
C4B63_89g54	-1.4341011292769	1.04143167641647e-07	Core
C4B63_55g180	1.31553166298158	1.05685986754597e-07	Core
C4B63_3g1046	-2.44969144041125	1.05812690081971e-07	Disruptive
C4B63_12g362	-2.13931948040774	1.08062069210349e-07	Core
C4B63_16g166	1.03628783968709	1.09002919419104e-07	Core
C4B63_11g281	-3.13719401146954	1.09429697989792e-07	Disruptive
C4B63_211g28	1.13225573872016	1.09450744385119e-07	Core
C4B63_26g233	1.12833537288752	1.10336047527438e-07	Core
C4B63_27g296	-2.57319210287994	1.10336047527438e-07	Disruptive
C4B63_351g6	1.05345459088375	1.1142780085235e-07	Core
C4B63_76g52	1.15863266427661	1.11600327688709e-07	Core
C4B63_6g595	1.01435940408089	1.14758801370896e-07	Core
C4B63_58g125	1.04918230421534	1.14842637815164e-07	Core
C4B63_2g693	1.27111013054398	1.16036187148249e-07	Core
C4B63_8g235	-2.63073146975728	1.16917405313688e-07	Disruptive
C4B63_78g28	1.17133449647595	1.18329628914164e-07	Core
C4B63_15g353	-2.4193951565248	1.22171111341945e-07	Disruptive
C4B63_66g151	1.26054683424507	1.25433653016054e-07	Core
C4B63_37g389	1.33295070656079	1.26106254509615e-07	Core
C4B63_15g17	1.39841751008199	1.26392261710061e-07	Core
C4B63_150g11	-1.70061776834006	1.27973760888954e-07	Disruptive
C4B63_8g213	1.02557105763047	1.29194697487369e-07	Core
C4B63_8g259	-3.15364780392997	1.29616643828781e-07	Disruptive
C4B63_95g50	-1.04043491135575	1.3082058636824e-07	Core
C4B63_286g15	1.81572388645866	1.3304563708219e-07	Core
C4B63_408g13	-8.69630570330887	1.3311714927108e-07	Disruptive
C4B63_5g121	-2.56658920839632	1.34093232740346e-07	Disruptive
C4B63_65g61	-2.73748925653536	1.39985656613611e-07	Disruptive
C4B63_87g94	-1.60057443423641	1.402566572013e-07	Core
C4B63_40g17	-1.49708373947235	1.44881065657246e-07	Core
C4B63_10g312	-1.38522393052967	1.44995932661761e-07	Core
C4B63_49g112	1.42966899281694	1.45468499743058e-07	Core
C4B63_3g864	-1.90208883201099	1.48666813719025e-07	Disruptive
C4B63_14g113	-1.21885469511004	1.5134505041325e-07	Core
C4B63_31g256	-2.12756467413942	1.53276301580821e-07	Disruptive
C4B63_100g73	-3.51905452729534	1.53408505596929e-07	Disruptive
C4B63_1g562	-2.42535866026805	1.53778944157417e-07	Disruptive
C4B63_22g66	1.04128672995653	1.55376268021018e-07	Core
C4B63_38g62	-1.05791091977469	1.58828015368939e-07	Core
C4B63_57g39	-2.52936137116043	1.59241686693853e-07	Disruptive
C4B63_37g109	-2.81125192767631	1.59538930332143e-07	Disruptive
C4B63_156g14	-1.77531906074805	1.59538930332143e-07	Core
C4B63_23g1250c	1.10109319532283	1.5982471305198e-07	Core
C4B63_33g272	-2.38652741436805	1.60698044758989e-07	Disruptive
C4B63_257g7	1.22660619107554	1.62624997331691e-07	Core
C4B63_259g18	-1.32941978009372	1.71943644295631e-07	Disruptive
C4B63_210g35	1.37874033078686	1.72510147610053e-07	Core
C4B63_90g60	-1.60245435639654	1.72587098321891e-07	Core
C4B63_156g6	-4.00890336294183	1.73037592858124e-07	Disruptive
C4B63_14g101	1.65199011343254	1.75303036413494e-07	Core
C4B63_55g177	1.01206068926605	1.75962114441927e-07	Core
C4B63_34g379	-1.65276007562762	1.78587958002554e-07	Disruptive
C4B63_26g311	1.84927955555533	1.79168398220074e-07	Core
C4B63_1g1315	-2.43943068829488	1.84121061827514e-07	Disruptive
C4B63_33g43	-6.11032712578562	1.84607198632395e-07	Disruptive
C4B63_3g957	-2.64199163081722	1.85078870164929e-07	Disruptive
C4B63_50g188	-2.7143033750918	1.87079001861449e-07	Disruptive
C4B63_483g4	1.06236412716939	1.93903131838279e-07	Core

C4B63_62g165	-3.22287907934064	1.96325938461174e-07	Disruptive
C4B63_34g331	1.86743504687188	1.97277802683699e-07	Disruptive
C4B63_165g10	-2.40285327631846	2.05732291678653e-07	Disruptive
C4B63_27g56	-2.50112891818741	2.09181955283816e-07	Disruptive
C4B63_12g243	1.30206404773996	2.11550980227508e-07	Core
C4B63_125g16	-1.96298798252064	2.12118917978695e-07	Disruptive
C4B63_50g101	-3.20843316409177	2.1844281427544e-07	Disruptive
C4B63_14g70	1.03421728649213	2.19676937595375e-07	Core
C4B63_121g75	-2.17069613497387	2.20524417732805e-07	Disruptive
C4B63_2g1604c	1.02617724412722	2.21629563391476e-07	Core
C4B63_15g455	-2.4486663985129	2.22397489218078e-07	Disruptive
C4B63_163g21	1.42174713180859	2.28566013935873e-07	Core
C4B63_16g188	1.15606075510217	2.29380542359982e-07	Core
C4B63_11g20	1.38756412042326	2.29751242761989e-07	Core
C4B63_51g210	-1.42147623221579	2.33417333098384e-07	Disruptive
C4B63_27g300	-2.20961557711478	2.34616515837456e-07	Disruptive
C4B63_126g13	-1.91705807059465	2.35006457720282e-07	Disruptive
C4B63_193g14	1.27766254222698	2.3565135667088e-07	Core
C4B63_34g1506c	1.92391588751604	2.40423025297672e-07	Disruptive
C4B63_36g310	-2.32737852721842	2.42573190820884e-07	Disruptive
C4B63_4g281	1.07379540534464	2.4655724301572e-07	Core
C4B63_24g1258c	1.79097613201762	2.53144978851025e-07	Core
C4B63_15g369	-2.80587607301157	2.53669351562739e-07	Disruptive
C4B63_168g17	1.06475836220068	2.54628920920139e-07	Core
C4B63_74g138	-2.48009151283269	2.56267302957585e-07	Disruptive
C4B63_41g203	1.68979083159458	2.68648179087932e-07	Core
C4B63_10g457	1.14823153902598	2.70135724229687e-07	Core
C4B63_58g130	1.12313247409902	2.71092914200282e-07	Core
C4B63_18g326	1.12407810896679	2.72991531437792e-07	Core
C4B63_50g230	-2.58359610342001	2.73709418868213e-07	Disruptive
C4B63_2g50	1.84999707088307	2.74131728310569e-07	Core
C4B63_106g41	1.67743771182054	2.74317033548264e-07	Core
C4B63_112g35	1.16630441034338	2.78061197983757e-07	Core
C4B63_17g118	-1.12059641287188	2.78402152002886e-07	Core
C4B63_1g671	-2.32715187235491	2.79331905905386e-07	Disruptive
C4B63_145g28	1.43939743765385	2.82309784829558e-07	Core
C4B63_49g109	1.17968581312222	2.86157149706981e-07	Core
C4B63_50g98	-2.4135659428278	2.8627514170718e-07	Disruptive
C4B63_308g5	-1.48977078303516	2.86962121410505e-07	Core
C4B63_1g1058	-2.29103504242446	2.87928172456011e-07	Disruptive
C4B63_326g24	-2.29545771851269	2.88647852036423e-07	Core
C4B63_20g204	1.13316189628363	3.00983786893129e-07	Core
C4B63_6g607	1.08783362822778	3.04000968364764e-07	Core
C4B63_41g296	1.0391783928106	3.10564291310336e-07	Core
C4B63_1g544	-2.59050493228285	3.12542354059312e-07	Disruptive
C4B63_114g38	1.21035163005629	3.13807911355654e-07	Core
C4B63_59g52	-3.94304477112829	3.14242300821445e-07	Disruptive
C4B63_1g225	-2.87347011464786	3.32162984955958e-07	Disruptive
C4B63_99g97	-1.8209535035165	3.40216253946524e-07	Disruptive
C4B63_470g8	-1.46929496731701	3.40423196839371e-07	Core
C4B63_4g545	1.84159086403701	3.49564309038148e-07	Core
C4B63_1g1146	-3.00578843241356	3.52108566014081e-07	Disruptive
C4B63_123g31	-1.49521196618309	3.66474176398784e-07	Core
C4B63_42g55	-1.40679311943415	3.73059225054192e-07	Core
C4B63_23g6	-2.00978706033604	3.73599060170647e-07	Core
C4B63_1g1342	-2.16003738595811	3.74854527528619e-07	Disruptive
C4B63_7g443	1.02225183572068	3.74854527528619e-07	Core
C4B63_1g1002	-1.98161425217483	3.82877206348755e-07	Disruptive
C4B63_1g526	-2.8450968339862	3.87796859112204e-07	Disruptive
C4B63_42g251	1.24955474031266	3.87919613695498e-07	Core
C4B63_42g67	1.27401150468632	4.04876697512506e-07	Core
C4B63_121g27	-1.9245047681514	4.08206082213889e-07	Disruptive
C4B63_9g243	1.09132448526695	4.08378838510639e-07	Core
C4B63_72g28	-1.66425317824558	4.0942571943324e-07	Disruptive

C4B63_197g31	-1.43669709949801	4.12927276768684e-07	Disruptive
C4B63_21g163	1.12547964175097	4.13237136636508e-07	Core
C4B63_3g1044	-1.93584752259378	4.17093528169734e-07	Disruptive
C4B63_100g59	-1.93741220350389	4.23680054272425e-07	Disruptive
C4B63_96g56	-2.9408268355039	4.27779496319135e-07	Disruptive
C4B63_9g209	1.26096277928956	4.34252250988074e-07	Core
C4B63_330g10	1.68519793673588	4.407321376962e-07	Core
C4B63_70g86	1.21096241735424	4.41825758068412e-07	Core
C4B63_28g240	-1.65673524316576	4.50911207170079e-07	Core
C4B63_6g2482c	1.2058819469851	4.52837677114658e-07	Core
C4B63_3g865	-1.95067865638956	4.53505901629669e-07	Disruptive
C4B63_45g248	-1.68895641723267	4.5748133365531e-07	Core
C4B63_154g15	-2.02679390745831	4.65265391179711e-07	Disruptive
C4B63_5g388	-2.41503203221222	4.74172259654817e-07	Disruptive
C4B63_130g19	-1.26392659951497	4.79049296008082e-07	Disruptive
C4B63_169g46	-6.00531975653057	4.85542383457596e-07	Disruptive
C4B63_139g13	-4.052378646218	4.90638691519467e-07	Disruptive
C4B63_330g3	2.66063242088288	4.98668998870831e-07	Core
C4B63_15g503	-2.76009366688115	4.99248122065222e-07	Disruptive
C4B63_137g43	-1.77893458988687	5.00749302826854e-07	Core
C4B63_21g249	-1.13661473605879	5.02352817826621e-07	Core
C4B63_31g264	1.46041480912549	5.03052807958287e-07	Core
C4B63_1g1263	-2.19376483494226	5.03308604634478e-07	Disruptive
C4B63_44g232	1.34323441026154	5.13634206868436e-07	Core
C4B63_201g21	1.22117103724479	5.16780097256548e-07	Core
C4B63_41g248	1.52652042516199	5.22282042118894e-07	Core
C4B63_29g134	-1.81918690261754	5.22687179494887e-07	Disruptive
C4B63_60g160	1.39148014596563	5.22687179494887e-07	Core
C4B63_1g1397	-1.57245116601025	5.35603075006588e-07	Disruptive
C4B63_12g266	-1.71821248023303	5.36762315766083e-07	Disruptive
C4B63_23g1324c	1.55705147779717	5.38067976093927e-07	Core
C4B63_29g107	-2.55419150216818	5.4070994382147e-07	Disruptive
C4B63_240g12	-1.57720546056198	5.4070994382147e-07	Disruptive
C4B63_64g133	-1.54665347753915	5.58808460874279e-07	Disruptive
C4B63_18g322	1.3317653061529	5.59350706286689e-07	Core
C4B63_9g511	1.1449189016305	5.59630329706415e-07	Core
C4B63_44g188	-1.66959770457113	5.59630950164088e-07	Core
C4B63_17g327	1.97369628454356	5.6111630589901e-07	Core
C4B63_55g53	-8.02527492449966	5.74751977170733e-07	Core
C4B63_147g68	1.25629930867752	5.76563866140933e-07	Core
C4B63_172g38	-1.9985884674911	5.93411190060225e-07	Disruptive
C4B63_14g129	-2.12261766849472	5.9750335378403e-07	Disruptive
C4B63_28g225	2.57507460241044	6.05850683696374e-07	Core
C4B63_53g59	-2.92570558249507	6.15110899638643e-07	Disruptive
C4B63_27g220	1.08361820243231	6.19215409698035e-07	Core
C4B63_7g226	-1.05642245147387	6.27562714335066e-07	Core
C4B63_39g157	-2.51893529373255	6.27562714335066e-07	Disruptive
C4B63_39g341	1.16998865652628	6.42262675953506e-07	Core
C4B63_5g184	-2.51422755130449	6.43938600754066e-07	Disruptive
C4B63_3g1128	-1.78328746526057	6.59410632104776e-07	Core
C4B63_11g124	-2.33329599030207	6.81217749718719e-07	Disruptive
C4B63_25g330	-1.14983475419761	6.8142544776613e-07	Core
C4B63_62g138	-4.48048530048074	6.88863086258224e-07	Disruptive
C4B63_127g46	1.60556712811017	6.89910169865008e-07	Core
C4B63_1g1084	-1.9063432340168	6.96594625539013e-07	Disruptive
C4B63_16g317	1.69597303759585	6.97475007821736e-07	Core
C4B63_603g9	-8.16679743492098	6.99189961609168e-07	Core
C4B63_27g82	-8.01882594874098	7.02257412790396e-07	Disruptive
C4B63_68g83	1.13799938724904	7.16993341172158e-07	Core
C4B63_20g339	-1.59056569324431	7.2418144651354e-07	Core
C4B63_107g92	-2.77459987859544	7.29706078921041e-07	Disruptive
C4B63_6g612	-3.05170738721169	7.43537681421214e-07	Disruptive
C4B63_1g564	-2.10758065490615	7.48717564844348e-07	Disruptive
C4B63_39g153	-3.32149656376903	7.59499625157067e-07	Disruptive

C4B63_75g100	1.28620777733278	7.62787773032849e-07	Core
C4B63_74g106	-4.44749383833505	7.7507385599292e-07	Disruptive
C4B63_15g332	-2.4268898629214	7.75957044218895e-07	Disruptive
C4B63_18g61	1.20813305501665	7.97359886793843e-07	Core
C4B63_61g131	1.03372391174329	8.17760531169112e-07	Core
C4B63_46g17	1.06275767290962	8.20134869794643e-07	Core
C4B63_12g200	1.042512000218	8.24274483147417e-07	Core
C4B63_6g149	1.18238714303011	8.2837669180307e-07	Core
C4B63_65g149	-1.84095799000642	8.30518917582699e-07	Disruptive
C4B63_2g437	1.11639930685335	8.35313705093847e-07	Core
C4B63_35g123	1.49845584212599	8.36520877286759e-07	Core
C4B63_29g330	-2.39771191294924	8.37423783095567e-07	Disruptive
C4B63_11g53	1.33514335860514	8.51295494995978e-07	Core
C4B63_1g1078	-2.38357132762122	8.71034660632531e-07	Disruptive
C4B63_33g146	1.32153065195456	8.71034660632531e-07	Core
C4B63_88g29	-2.21849417004273	8.74767023549629e-07	Disruptive
C4B63_5g251	-3.95133717587515	8.82137342835245e-07	Disruptive
C4B63_461g12	-3.54090592483445	8.82835744305225e-07	Disruptive
C4B63_24g169	-1.6853073064046	8.83985235054705e-07	Core
C4B63_132g19	-2.84931736091502	8.86135692829255e-07	Disruptive
C4B63_138g13	-1.35204501078705	8.97489878861454e-07	Disruptive
C4B63_93g43	1.12045767815424	9.14154547825547e-07	Core
C4B63_32g1409c	-1.1192724838397	9.29171031184206e-07	Core
C4B63_29g91	-2.50766850751794	9.29584140526634e-07	Disruptive
C4B63_183g32	2.29085135184896	9.29584140526634e-07	Core
C4B63_24g308	1.22864114735109	9.34738373871753e-07	Core
C4B63_5g538	-2.40137637328892	9.38437211245103e-07	Disruptive
C4B63_34g301	-1.1465307759668	9.54723602228994e-07	Core
C4B63_1g1307	-2.13682158215198	9.60740090574804e-07	Disruptive
C4B63_6g397	1.15273796502201	9.6099130962258e-07	Core
C4B63_21g282	1.15732597921308	9.61840359752158e-07	Core
C4B63_50g151	-2.47291005789932	9.64049643942142e-07	Disruptive
C4B63_115g12	-2.54189357673143	9.6655293219725e-07	Disruptive
C4B63_11g423	-1.90357239239765	9.82534031257816e-07	Disruptive
C4B63_127g44	1.49632403977296	9.90573171077213e-07	Core
C4B63_52g138	1.58508482247615	1.01825978401039e-06	Core
C4B63_108g41	1.02742230158812	1.01825978401039e-06	Core
C4B63_7g116	-1.05965873526073	1.02878298329589e-06	Core
C4B63_36g231	-3.66602900956306	1.02952197774326e-06	Disruptive
C4B63_44g181	-1.43591554154961	1.03169471036266e-06	Core
C4B63_103g44	1.47115543959249	1.04639818875236e-06	Core
C4B63_139g8	-7.80018877886118	1.11284211030562e-06	Disruptive
C4B63_86g84	1.34046398875042	1.11413456361911e-06	Core
C4B63_30g255	-2.58699119802988	1.12214268020251e-06	Core
C4B63_1g1090	-4.31543121358638	1.12564080727172e-06	Disruptive
C4B63_115g73	-2.2021992302174	1.14626465652245e-06	Disruptive
C4B63_177g27	1.14995825496107	1.15730282995011e-06	Core
C4B63_3g1036	-2.53191347268994	1.15857049430448e-06	Disruptive
C4B63_29g372	-1.92648704549539	1.19656890269364e-06	Disruptive
C4B63_62g194	-2.48457273553025	1.21539094093026e-06	Disruptive
C4B63_17g193	1.32020207638615	1.21932089752455e-06	Core
C4B63_32g265	1.25600677210198	1.24192039603146e-06	Core
C4B63_1g1244	-2.87908277605741	1.24382532900804e-06	Disruptive
C4B63_24g263	1.1157201524748	1.24705785527877e-06	Core
C4B63_19g254	-1.7429831802794	1.28529377690671e-06	Core
C4B63_1g1324	-2.69256779894219	1.28607720329847e-06	Disruptive
C4B63_99g68	-2.6709477228229	1.29204084867543e-06	Disruptive
C4B63_38g174	1.07638842132875	1.29394232857131e-06	Core
C4B63_59g45	-2.32134449162967	1.29431340854279e-06	Disruptive
C4B63_118g10	-1.46943894835065	1.30057052601447e-06	Disruptive
C4B63_39g104	-2.49686987462721	1.32157817156464e-06	Disruptive
C4B63_322g10	-8.0362281891646	1.34118265287109e-06	Core
C4B63_41g289	1.00863979609711	1.3488954472343e-06	Core
C4B63_126g10	1.1782505911112	1.34939468656327e-06	Core

C4B63_73g65	1.07902458214916	1.3524230316686e-06	Core
C4B63_37g80	-3.24234686666902	1.35411750947384e-06	Disruptive
C4B63_1g968	-1.83556052853952	1.36678889052983e-06	Disruptive
C4B63_17g231	1.20097356559718	1.36678889052983e-06	Core
C4B63_90g98	1.07411194865369	1.42994511830111e-06	Core
C4B63_15g116	-2.75611216662784	1.4411134170115e-06	Disruptive
C4B63_17g208	-8.07105551667124	1.45778855947089e-06	Disruptive
C4B63_144g10	-1.79800273415538	1.48143265337517e-06	Disruptive
C4B63_58g37	1.23786276752227	1.50082577894216e-06	Core
C4B63_1g607	-2.08766835326772	1.51543778107587e-06	Disruptive
C4B63_11g232	-2.42405099814722	1.53393451103743e-06	Disruptive
C4B63_3g80	-3.07056298208789	1.53629879692325e-06	Disruptive
C4B63_60g116	1.44053834532926	1.53629879692325e-06	Core
C4B63_15g400	-2.41759702891587	1.53886757864175e-06	Core
C4B63_5g44	-1.19146879378494	1.55395349369206e-06	Disruptive
C4B63_62g157	-3.47401978653303	1.55646600279455e-06	Disruptive
C4B63_88g97	-2.1401786087713	1.56994386128436e-06	Disruptive
C4B63_13g336	1.10360683723716	1.57212989896509e-06	Core
C4B63_71g117	-2.60539244308314	1.57934594786327e-06	Disruptive
C4B63_126g4	-2.27442975155439	1.58316340085478e-06	Disruptive
C4B63_1g813	-2.3792412030646	1.59230168189283e-06	Disruptive
C4B63_101g68	-2.36252656077027	1.6039142013621e-06	Disruptive
C4B63_19g217	1.48215926309492	1.60668514349938e-06	Core
C4B63_276g4	-1.68188226184178	1.64083899457665e-06	Disruptive
C4B63_46g107	1.29883651445286	1.65793505227224e-06	Core
C4B63_11g316	-2.83127208366963	1.66826878877995e-06	Disruptive
C4B63_1g1316	-3.36846236770892	1.68579722447452e-06	Disruptive
C4B63_64g141	-1.48135510251392	1.7179255328045e-06	Disruptive
C4B63_41g164	-2.01355520125072	1.73312798753577e-06	Core
C4B63_41g217	1.45313988844465	1.73854785728886e-06	Core
C4B63_174g17	-1.94305956516571	1.74041949423524e-06	Disruptive
C4B63_108g19	-1.60821119167051	1.74493839848742e-06	Disruptive
C4B63_44g214	-1.49755520534578	1.76389535393862e-06	Core
C4B63_186g23	2.00651526275078	1.76606064345462e-06	Core
C4B63_36g204	-1.75483349674052	1.78500934930579e-06	Disruptive
C4B63_13g335	1.13355733910893	1.80138188790863e-06	Core
C4B63_12g182	1.2599930453786	1.81939239910851e-06	Core
C4B63_8g108	-2.17581604301472	1.82369304394492e-06	Disruptive
C4B63_65g14	-2.80133355306676	1.84632006598353e-06	Core
C4B63_334g34	-2.80918158627919	1.86947872237783e-06	Disruptive
C4B63_1g1311	-2.2897478671611	1.88831234083382e-06	Disruptive
C4B63_1g732	-2.50744393639985	1.89191802316934e-06	Disruptive
C4B63_1g929	-3.15912580361968	1.90958696579109e-06	Disruptive
C4B63_108g18	-1.48329943713817	1.90958696579109e-06	Disruptive
C4B63_155g12	1.01752846811326	1.93803007016284e-06	Core
C4B63_27g261	-4.79441793430054	1.95316081350162e-06	Disruptive
C4B63_5g375	-2.01226098505503	1.96848577814471e-06	Disruptive
C4B63_108g6	1.92406537017481	2.0317785036766e-06	Core
C4B63_110g7	1.17119514734647	2.03331504263103e-06	Core
C4B63_13g260	1.32914767271403	2.03347339163396e-06	Core
C4B63_109g54	-1.62570421458438	2.03347339163396e-06	Disruptive
C4B63_33g40	-2.19315736056297	2.03733647770719e-06	Disruptive
C4B63_29g69	-2.62283545136018	2.05240311409027e-06	Disruptive
C4B63_79g92	1.44191813063454	2.05308252248192e-06	Core
C4B63_326g20	-3.2002536726656	2.05333991545252e-06	Core
C4B63_207g17	-1.21014515215207	2.06109123494762e-06	Core
C4B63_5g221	-3.61684548824622	2.08358559912535e-06	Disruptive
C4B63_3g1132	-1.89014565539738	2.12250416357783e-06	Disruptive
C4B63_6g258	1.02930020654578	2.1320217597277e-06	Core
C4B63_19g98	1.38207201876169	2.13874199861492e-06	Core
C4B63_70g78	-1.13454631671486	2.15128299117737e-06	Core
C4B63_13g322	1.32062175204344	2.16822480626066e-06	Core
C4B63_31g90	1.17590032118775	2.18007359569674e-06	Core
C4B63_5g227	-2.86811314986323	2.20651983418087e-06	Disruptive

C4B63_22g169	1.13683757907883	2.2134839027501e-06	Core
C4B63_77g28	-1.54738548734392	2.22109219943866e-06	Core
C4B63_37g89	-1.70225937730596	2.23364117178803e-06	Disruptive
C4B63_151g54	1.12032180241917	2.2429014175999e-06	Core
C4B63_27g127	1.45604390204037	2.25334362258495e-06	Core
C4B63_16g291	1.05495185696268	2.26630478477499e-06	Core
C4B63_76g60	1.29456398314225	2.31206670553719e-06	Core
C4B63_71g178	-2.49944207542688	2.31785611902189e-06	Disruptive
C4B63_60g146	1.37613971603094	2.35282113422178e-06	Core
C4B63_80g47	1.49284414696092	2.35950438634321e-06	Core
C4B63_193g17	1.29450280786052	2.40402896610308e-06	Core
C4B63_265g1	-4.02485921551052	2.47814158146671e-06	Core
C4B63_19g213	1.89309990900342	2.49657808735993e-06	Core
C4B63_65g41	-2.48509113044695	2.50292711213176e-06	Disruptive
C4B63_74g14	-2.7880522556057	2.54709023533878e-06	Disruptive
C4B63_3g888	-1.52565619667499	2.55740240289595e-06	Disruptive
C4B63_53g140	2.51558178850938	2.56567196423572e-06	Core
C4B63_40g91	1.15474956794108	2.57339175532652e-06	Core
C4B63_71g114	-2.92025570191101	2.60253353654674e-06	Disruptive
C4B63_24g194	1.67030830446616	2.60280428381408e-06	Core
C4B63_21g239c	1.05242298326036	2.64438443936228e-06	Core
C4B63_29g290	-3.30755721497873	2.69480722694418e-06	Disruptive
C4B63_175g15	-1.38469814023043	2.71841326582207e-06	Core
C4B63_47g160	-1.40832740603374	2.76933748573195e-06	Disruptive
C4B63_330g5	1.36349381788902	2.80176189667954e-06	Core
C4B63_1g779	-2.31700571249214	2.81705276054279e-06	Disruptive
C4B63_3g814	-2.91651759382011	2.82649116371093e-06	Disruptive
C4B63_169g35	-1.94922384094498	2.84894089936637e-06	Disruptive
C4B63_39g146	-2.98074110268135	2.86834388104497e-06	Disruptive
C4B63_3g580	-1.61232026939933	2.8748626439842e-06	Disruptive
C4B63_64g57	-2.49829726028502	2.87860472328137e-06	Disruptive
C4B63_1g849	-3.38744035683929	3.01840596031299e-06	Disruptive
C4B63_5g130	-2.47298536905565	3.07946558894179e-06	Disruptive
C4B63_1g794	-1.70311313737359	3.16059578561607e-06	Disruptive
C4B63_232g12	24.1807105145156	3.18327215135389e-06	Disruptive
C4B63_52g80	1.22346925810474	3.18678750300665e-06	Core
C4B63_195g13	1.18520944914561	3.22858678133224e-06	Core
C4B63_211g32	1.12509900506272	3.22858678133224e-06	Core
C4B63_56g149	1.15787635573346	3.24852353945522e-06	Core
C4B63_3g646	-2.02866522655123	3.27569901705119e-06	Disruptive
C4B63_20g344	-1.64015917763315	3.29485523142529e-06	Core
C4B63_29g362	-1.93493992604568	3.30349923290045e-06	Disruptive
C4B63_41g122	1.52773448952362	3.35311990390594e-06	Core
C4B63_8g82	-1.6093184618622	3.35757904710225e-06	Disruptive
C4B63_178g44	1.21128996729057	3.3970213084865e-06	Core
C4B63_76g16	1.02234476411091	3.5046998272357e-06	Core
C4B63_39g228	-2.00771988233937	3.52631591836003e-06	Disruptive
C4B63_11g376	-1.76120824348264	3.53046236523709e-06	Disruptive
C4B63_59g208	-3.30935432546638	3.54375305789736e-06	Disruptive
C4B63_31g92	1.0166752094794	3.57886060506341e-06	Core
C4B63_3g1118	1.26616689651526	3.59844697076538e-06	Core
C4B63_5g274	-2.35831459031997	3.68672149050811e-06	Disruptive
C4B63_98g38	-1.58124759060101	3.74240847702317e-06	Disruptive
C4B63_6g257	1.20435771608754	3.7508864949455e-06	Core
C4B63_1g1110	-2.1668765692924	3.75380199885975e-06	Disruptive
C4B63_485g5	1.90777841362359	3.76653775987136e-06	Core
C4B63_29g34	-2.05882181193908	3.76917663314972e-06	Disruptive
C4B63_101g65	-2.44189647325442	3.77219422654333e-06	Disruptive
C4B63_119g34	1.20785176238504	3.81410967530941e-06	Core
C4B63_11g336	-2.20696029105232	3.81716503842878e-06	Disruptive
C4B63_109g60	-1.26129428924493	3.85662680395324e-06	Disruptive
C4B63_59g231	-2.67165118314426	3.88697797381343e-06	Disruptive
C4B63_9g447	1.13794291084393	3.91531116175413e-06	Core
C4B63_5g774	1.33134217932981	4.01357754744517e-06	Core

C4B63_22g75	-1.24072658869859	4.11224009074503e-06	Core
C4B63_1g382	-2.49177353834528	4.13219332413561e-06	Disruptive
C4B63_4g65	1.2416276791101	4.13237544038475e-06	Core
C4B63_3g448	-2.38012356318989	4.1406764662446e-06	Disruptive
C4B63_1g246	-2.36721202162533	4.14743589455482e-06	Disruptive
C4B63_37g106	-1.67098810951925	4.15564540667367e-06	Disruptive
C4B63_15g317	-2.64173353257322	4.16773504438222e-06	Disruptive
C4B63_40g179	-1.24080652133253	4.2998841600356e-06	Disruptive
C4B63_39g98	-1.95516224839335	4.31945276518285e-06	Disruptive
C4B63_11g343	-1.5866337249956	4.43151760602328e-06	Disruptive
C4B63_17g233	1.03444847161487	4.44589446875951e-06	Core
C4B63_61g137	1.05156976530978	4.48747539522376e-06	Core
C4B63_3g686	-3.07068901270732	4.51561024041224e-06	Disruptive
C4B63_3g595	-2.42667299527334	4.61445307915394e-06	Disruptive
C4B63_202g28	1.55791720856917	4.62147680640812e-06	Core
C4B63_319g8	-1.31170026612778	4.63230554457811e-06	Disruptive
C4B63_3g380	-2.98343067601438	4.63578101860925e-06	Disruptive
C4B63_67g69	-3.57156352193009	4.65209134522078e-06	Disruptive
C4B63_1g818	-2.21115208932776	4.69281643676887e-06	Disruptive
C4B63_85g54	1.17217123518848	4.70564429808263e-06	Core
C4B63_105g54	1.31489217480437	4.70564429808263e-06	Core
C4B63_105g51	-1.49833306453809	4.71417419912224e-06	Disruptive
C4B63_44g162	-1.52849838780286	4.72385524698583e-06	Core
C4B63_64g55	-1.87029317358141	4.80613041853648e-06	Disruptive
C4B63_101g13	-1.06809391387447	4.91043844134143e-06	Core
C4B63_3g743	-3.85415754139812	4.92638447774285e-06	Disruptive
C4B63_135g35	-1.65535114231677	4.95418875188243e-06	Disruptive
C4B63_10g512	1.14529122407359	4.95754039455446e-06	Core
C4B63_150g18	-2.38598959729647	4.98035003488859e-06	Disruptive
C4B63_228g39	-1.58063719205463	5.04603389333962e-06	Core
C4B63_125g60	-2.99823357616094	5.08065199342862e-06	Disruptive
C4B63_17g324	-2.00297864002345	5.10005884034154e-06	Core
C4B63_35g135	-1.71776951202941	5.13241950070348e-06	Disruptive
C4B63_7g410	1.28993390272161	5.15747453863476e-06	Core
C4B63_66g51	-2.91031060684366	5.22731610096434e-06	Disruptive
C4B63_36g363	-2.37728680230629	5.3009682460045e-06	Disruptive
C4B63_80g53	1.4319388713046	5.38543105284942e-06	Core
C4B63_1g809	-2.14827728824743	5.50851774517919e-06	Disruptive
C4B63_1g1346	-2.99755317670742	5.55875720675738e-06	Disruptive
C4B63_1g495	-1.86996415693583	5.59310905241762e-06	Disruptive
C4B63_13g19	1.10805600396433	5.61136482520985e-06	Core
C4B63_5g77	-1.54961416243083	5.73164028420652e-06	Disruptive
C4B63_8g253	-2.97507086464542	5.75412155916414e-06	Disruptive
C4B63_9g242	1.28984555264481	5.81779708025812e-06	Core
C4B63_161g33	1.31179522495992	5.92030869227705e-06	Core
C4B63_128g40	1.06425108974206	5.97797564350492e-06	Core
C4B63_12g34	-3.03096834793061	6.2666302444621e-06	Core
C4B63_25g238	1.38017620547816	6.26993582783454e-06	Core
C4B63_25g315	-1.15983495331948	6.29588307418511e-06	Core
C4B63_23g122	1.11723420528669	6.29838049521074e-06	Core
C4B63_15g268	-1.97356587979487	6.31394014422703e-06	Disruptive
C4B63_36g38	1.26269994208356	6.35900068659061e-06	Core
C4B63_90g107	-3.519761359013	6.50370689240739e-06	Disruptive
C4B63_39g65	-2.26288862812092	6.6308761445111e-06	Disruptive
C4B63_37g369	-1.1827058883817	6.82011341810113e-06	Core
C4B63_16g109	1.88086649614987	6.84818980348884e-06	Core
C4B63_33g47	-2.34235068096305	6.98415742757142e-06	Disruptive
C4B63_62g23	-2.67214515133614	7.04894729727082e-06	Disruptive
C4B63_50g227	-2.4421734837878	7.08469834589464e-06	Disruptive
C4B63_54g87	-3.02643121438751	7.25952697027534e-06	Disruptive
C4B63_15g306	-2.71990225030875	7.29788741123706e-06	Disruptive
C4B63_313g16	2.38865653944036	7.32856219623599e-06	Core
C4B63_1g38	-1.68548702494856	7.4725307853839e-06	Core
C4B63_212g8	-7.14634322305249	7.50412684170629e-06	Core

C4B63_203g5	1.12665722423154	7.5523256012715e-06	Core
C4B63_26g55	-1.71978441656914	7.72263998456866e-06	Disruptive
C4B63_12g35	-1.47179187918364	7.8667817278218e-06	Disruptive
C4B63_15g26	-2.42457503526262	7.8667817278218e-06	Disruptive
C4B63_37g189	-1.47027167076192	8.01599497187924e-06	Disruptive
C4B63_39g147	-2.32852652108665	8.14411646722197e-06	Disruptive
C4B63_25g236	1.0761518381676	8.18557838540299e-06	Core
C4B63_27g291	-2.70490415562291	8.19039844150236e-06	Disruptive
C4B63_189g23	-1.40318016895603	8.20959635321087e-06	Disruptive
C4B63_64g68	-1.56573702751698	8.25320116501252e-06	Disruptive
C4B63_38g259	1.82520764843955	8.37981930897606e-06	Core
C4B63_3g391	-2.11233765447519	8.4003634397464e-06	Disruptive
C4B63_392g24	1.94466455006715	8.4003634397464e-06	Core
C4B63_51g226	-2.15976449816489	8.40570587348299e-06	Disruptive
C4B63_96g86	-2.00487482503979	8.55919232873312e-06	Disruptive
C4B63_101g64	-2.10992704973955	8.57950428159458e-06	Disruptive
C4B63_17g97	1.23734424165552	8.75199705379695e-06	Core
C4B63_71g131	-7.38466087977516	8.75199705379695e-06	Disruptive
C4B63_3g1066	-2.67370741420936	8.80716667408114e-06	Disruptive
C4B63_26g1224c	-2.1222558729284	8.83970860233007e-06	Core
C4B63_122g39	-5.86868729501833	8.8843706534104e-06	Disruptive
C4B63_1g1198	-2.79255049122799	8.89732155878352e-06	Disruptive
C4B63_32g233	1.17131897715778	8.91392580438333e-06	Core
C4B63_13g22	-7.49558276894906	8.94273450354695e-06	Disruptive
C4B63_13g367	-2.30858156804654	9.08905552980919e-06	Disruptive
C4B63_33g44	-3.24765896308433	9.18211209106343e-06	Disruptive
C4B63_3g73	-2.87164117612291	9.25957174312186e-06	Disruptive
C4B63_159g9	-1.51320100778836	9.35757266800813e-06	Disruptive
C4B63_289g3	-1.71544959537047	9.52254937750422e-06	Core
C4B63_354g14	1.06523189493247	9.52254937750422e-06	Core
C4B63_11g496	1.2156427807263	9.7590795374826e-06	Core
C4B63_13g277	1.3000205713724	9.86007905745006e-06	Core
C4B63_160g23	-1.09334731423166	9.92254171018747e-06	Disruptive
C4B63_12g45	-2.4307179928459	1.00414995087973e-05	Core
C4B63_12g412	1.03174152190168	1.00497077620446e-05	Core
C4B63_5g352	2.17137575756539	1.01265497909866e-05	Core
C4B63_16g205	1.67967590369825	1.02402590029813e-05	Core
C4B63_133g28	1.2852706358344	1.02459168667953e-05	Core
C4B63_134g48	-7.37029869751668	1.02496344533799e-05	Disruptive
C4B63_94g84	1.09594920206019	1.04387859530885e-05	Core
C4B63_3g919	-2.1347529776607	1.05086656011797e-05	Core
C4B63_5g326	-2.18407431325101	1.07220574821299e-05	Disruptive
C4B63_9g476	-7.27580767024412	1.07979907205963e-05	Disruptive
C4B63_139g11	-7.51804565366512	1.08023483171054e-05	Disruptive
C4B63_43g1	1.12822959111055	1.0885124302831e-05	Core
C4B63_36g142	1.15452635446043	1.12672464354774e-05	Core
C4B63_91g74	-2.44935647051769	1.12672464354774e-05	Disruptive
C4B63_21g87	1.52339441040657	1.15142137905295e-05	Core
C4B63_334g25	-1.65690627520664	1.16036758303731e-05	Disruptive
C4B63_20g34	1.1040310989156	1.16376492532308e-05	Core
C4B63_92g16	-1.91808835498256	1.18903884159594e-05	Core
C4B63_1g582	-1.87821747386778	1.1984466935819e-05	Disruptive
C4B63_3g790	-2.55279823202474	1.19872767699509e-05	Disruptive
C4B63_1g661	-2.31690577637123	1.20384321325026e-05	Disruptive
C4B63_13g16	8.53187277317563	1.21474270591202e-05	Disruptive
C4B63_3g815	-2.37041549594633	1.23612752316593e-05	Disruptive
C4B63_225g28	-7.1013806124259	1.24023920631869e-05	Core
C4B63_87g89	-1.4341349179375	1.25268244792447e-05	Core
C4B63_41g194	1.02090450028388	1.25278609971943e-05	Core
C4B63_96g81	-2.0268995534183	1.25837854947805e-05	Disruptive
C4B63_27g54	-2.47452581903846	1.26250354962859e-05	Disruptive
C4B63_39g144	-2.27990225353393	1.26250354962859e-05	Disruptive
C4B63_20g331	-2.14949729917922	1.2683722596265e-05	Core
C4B63_13g12	-1.60523913268219	1.28302462769753e-05	Disruptive

C4B63_144g22	-7.12835508520997	1.29362508052601e-05	Core
C4B63_143g3	-1.54507972190461	1.29708958044173e-05	Core
C4B63_11g147	-1.81100509897712	1.30191294763279e-05	Disruptive
C4B63_35g337	1.09133699833764	1.30830452623929e-05	Core
C4B63_8g162	-1.87178759851339	1.32041803565809e-05	Disruptive
C4B63_39g352	2.08354587073628	1.32041803565809e-05	Core
C4B63_8g505	1.20011713675358	1.34022002112035e-05	Core
C4B63_141g26	1.17997248432921	1.34277372053408e-05	Core
C4B63_7g145	1.23774276712537	1.34664128946887e-05	Core
C4B63_2g510	-1.17721756388413	1.35626007194517e-05	Core
C4B63_24g245	1.59477767597777	1.36530815380172e-05	Core
C4B63_64g191	1.05557672526179	1.36611319252966e-05	Core
C4B63_247g17	1.3372143621853	1.37454665099592e-05	Core
C4B63_29g327	-2.18631373147495	1.38437537303982e-05	Disruptive
C4B63_32g186	-2.28573206465048	1.38437537303982e-05	Disruptive
C4B63_1g341	-1.57529467463366	1.39158161082293e-05	Disruptive
C4B63_80g54	1.03616816346852	1.40019122578432e-05	Core
C4B63_8g252	-4.0756349310991	1.40110759063702e-05	Disruptive
C4B63_1g1320	-2.84550164450825	1.42518620144433e-05	Disruptive
C4B63_56g38	1.4858910080876	1.42829097915568e-05	Core
C4B63_17g176	-1.12114980087804	1.45911956206472e-05	Core
C4B63_369g4	-2.37030177602438	1.45911956206472e-05	Core
C4B63_183g20	1.70108830035111	1.47639933076854e-05	Core
C4B63_30g173	1.31199426711485	1.4863836775499e-05	Core
C4B63_118g4	1.60733598284933	1.48664250721629e-05	Core
C4B63_3g843	-2.1459184739725	1.50972497789622e-05	Disruptive
C4B63_27g269	-2.1353070978819	1.51600915032705e-05	Disruptive
C4B63_25g331	1.39178763067764	1.51784164588315e-05	Disruptive
C4B63_104g103	1.38569677843618	1.54030809300638e-05	Core
C4B63_1g964	-2.60157083430358	1.59066611193693e-05	Disruptive
C4B63_72g7	1.09665653811672	1.59144514895753e-05	Core
C4B63_34g390	1.13576602590775	1.59412388399755e-05	Core
C4B63_32g139	-2.03351718634695	1.59763739024459e-05	Disruptive
C4B63_16g4	-7.26229602465134	1.60588204281317e-05	Core
C4B63_39g128	-1.9125810259586	1.61081185711136e-05	Disruptive
C4B63_113g27	1.42901709765325	1.6219548097314e-05	Core
C4B63_26g284	1.26407798266912	1.70252554016155e-05	Core
C4B63_55g200	-1.6403787251749	1.70565929203936e-05	Disruptive
C4B63_31g85	1.24960276458155	1.70790993347213e-05	Core
C4B63_85g47	1.15076297894077	1.70790993347213e-05	Core
C4B63_40g112	1.06117340722551	1.73047495493179e-05	Core
C4B63_281g7	-1.84752812521891	1.73047495493179e-05	Core
C4B63_14g231	-1.47013853191002	1.73576146547493e-05	Disruptive
C4B63_59g101	-3.03769970888906	1.75637127952796e-05	Disruptive
C4B63_17g109	-1.17047396770351	1.78819962125466e-05	Core
C4B63_516g4	1.3286810478753	1.78904370530668e-05	Core
C4B63_12g151	1.70281969229557	1.7936965897285e-05	Core
C4B63_29g223	1.1357607371031	1.81407602426628e-05	Disruptive
C4B63_25g160	1.07025447940917	1.81524787492864e-05	Core
C4B63_50g242	-3.10911846482701	1.82040543438728e-05	Disruptive
C4B63_3g858	-4.43967093093069	1.82367270132172e-05	Disruptive
C4B63_15g337	-2.16283183530956	1.82454841502581e-05	Disruptive
C4B63_58g113	-1.34934996815999	1.82917383171138e-05	Core
C4B63_36g114	-2.29263244564451	1.83750276322254e-05	Disruptive
C4B63_1g239	-1.09210647997765	1.87530627159085e-05	Disruptive
C4B63_233g30	-1.18281876929396	1.92740127732235e-05	Core
C4B63_351g1	-7.50618588647092	1.93038124708978e-05	Core
C4B63_91g67	-2.60701683012917	1.93222029712432e-05	Disruptive
C4B63_123g77	-4.28478865250779	1.93457485268063e-05	Disruptive
C4B63_11g116	-1.80959865930192	1.93832322559782e-05	Disruptive
C4B63_74g137	-1.91237568014942	1.94264175245138e-05	Disruptive
C4B63_91g59	1.36643870500242	1.95855787959755e-05	Core
C4B63_1g388	-2.0456730196122	1.98010314311989e-05	Disruptive
C4B63_25g968c	1.08120193194922	2.00523423535985e-05	Core

C4B63_42g214	-1.5548773210915	2.00523423535985e-05	Core
C4B63_1g514	-2.24511325981477	2.01937245886985e-05	Disruptive
C4B63_3g999	-2.43882655961511	2.0565507208086e-05	Disruptive
C4B63_113g39	1.71502709737021	2.05666686246493e-05	Core
C4B63_3g911	-1.66692402456681	2.0768778987194e-05	Disruptive
C4B63_202g11	1.14362833621208	2.08105059300738e-05	Core
C4B63_122g41	-3.60847832301218	2.09183140176039e-05	Disruptive
C4B63_5g256	-2.2316815896125	2.09494946346162e-05	Disruptive
C4B63_172g24	1.04398082056428	2.10415317930709e-05	Core
C4B63_138g2	1.29643889243797	2.10837140474166e-05	Core
C4B63_71g158	-3.19649293444368	2.15177324450204e-05	Disruptive
C4B63_235g12	-2.205971160216	2.16983976542056e-05	Core
C4B63_70g127	-1.21369927610553	2.18831901147946e-05	Core
C4B63_5g262	1.70826397304794	2.25430560163657e-05	Core
C4B63_56g154	-2.34080219511461	2.27626897295714e-05	Disruptive
C4B63_21g253	1.07404455672327	2.30926777666618e-05	Core
C4B63_16g136	2.24702628228344	2.31488118668561e-05	Core
C4B63_60g133	1.19842783206843	2.33334994377184e-05	Core
C4B63_103g68	-2.18167356673585	2.36333109587532e-05	Disruptive
C4B63_196g32	-1.48308781937733	2.37267621480751e-05	Disruptive
C4B63_5g297	-2.33929312206227	2.3742535721031e-05	Disruptive
C4B63_19g225	1.37275753924919	2.39673523380717e-05	Core
C4B63_15g399	-3.01018242474224	2.3990441358351e-05	Disruptive
C4B63_41g120	-1.65188972022736	2.40293438800288e-05	Core
C4B63_615g4	3.24443192215323	2.43495121750046e-05	Core
C4B63_67g14	-2.9265531174449	2.43551153281698e-05	Disruptive
C4B63_301g4	1.88636371266791	2.47719182497696e-05	Core
C4B63_5g151	-2.1055025499663	2.54296362426937e-05	Disruptive
C4B63_14g178	1.34117398441624	2.56771556800514e-05	Core
C4B63_14g60	1.12393973404838	2.56934301667922e-05	Core
C4B63_1g111	-2.89267865445006	2.62847525385715e-05	Disruptive
C4B63_170g23	-1.1378166858489	2.64488089486867e-05	Core
C4B63_30g117	1.04784833729526	2.67073113781379e-05	Core
C4B63_69g40	-2.02012643118519	2.7019028001964e-05	Disruptive
C4B63_120g71	1.06402736447317	2.7019028001964e-05	Core
C4B63_60g197	1.38283183832806	2.72116202809099e-05	Core
C4B63_551g2	1.15037352166727	2.81907147728506e-05	Core
C4B63_67g75	-3.38234795852169	2.83429464443561e-05	Disruptive
C4B63_63g40	-1.24567403510911	2.91863640270374e-05	Core
C4B63_36g105	1.30345614813816	2.92673497079591e-05	Core
C4B63_36g111	-2.3045479654153	2.95414798804848e-05	Disruptive
C4B63_85g60	1.02909113924063	2.9924768924024e-05	Core
C4B63_62g159	-1.93268969923791	3.0125352866471e-05	Disruptive
C4B63_71g5	-6.62761175831331	3.0125352866471e-05	Disruptive
C4B63_15g34	-1.90225962318945	3.02744073746749e-05	Disruptive
C4B63_3g932	-2.06509837773805	3.03310121834348e-05	Disruptive
C4B63_37g205	-4.34353585925262	3.03491539415296e-05	Disruptive
C4B63_100g2	1.11123837086331	3.03491539415296e-05	Core
C4B63_336g2	2.18498084320124	3.07638765686732e-05	Core
C4B63_5g780	-1.15253613793973	3.10500258222703e-05	Disruptive
C4B63_29g84	-2.42493142173308	3.11425385299017e-05	Disruptive
C4B63_165g11	-1.47588869627874	3.11656912783032e-05	Disruptive
C4B63_56g94	1.0555168196878	3.11694064792948e-05	Core
C4B63_1g1237	-3.03210676431622	3.12086655720778e-05	Disruptive
C4B63_71g126	-3.87447998679779	3.1356542620807e-05	Disruptive
C4B63_69g49	-1.47165837628335	3.13823062057538e-05	Disruptive
C4B63_10g472	1.41775906628072	3.14077114183343e-05	Core
C4B63_147g47	1.14111557147255	3.14900871550736e-05	Core
C4B63_19g821c	1.25247179533754	3.18485941287214e-05	Core
C4B63_24g204	1.12887740555857	3.20268714653463e-05	Core
C4B63_121g88	-2.11688858019563	3.28371952666306e-05	Disruptive
C4B63_1g702	-1.36109360330273	3.34541356200991e-05	Disruptive
C4B63_36g369	-1.60415201386674	3.36375216368375e-05	Disruptive
C4B63_1g97	-1.14002085393726	3.3847113946129e-05	Disruptive

C4B63_1g1145	-1.93815535682757	3.39463326365123e-05	Disruptive
C4B63_56g90	1.17784610184718	3.39493614874096e-05	Core
C4B63_33g148	-1.34416840273235	3.4051315447206e-05	Disruptive
C4B63_138g33	-7.17339607856304	3.40999911669144e-05	Core
C4B63_96g41	-2.47477327096583	3.42421211375353e-05	Disruptive
C4B63_4g175	-1.38350955539388	3.51195873053081e-05	Core
C4B63_213g8	-3.06888909535347	3.55674395459682e-05	Disruptive
C4B63_621g1	1.30210083768822	3.56540472422424e-05	Core
C4B63_7g140	-1.18302300681884	3.57334390838846e-05	Core
C4B63_50g220	-2.26335841100557	3.58417652604656e-05	Disruptive
C4B63_15g50	-1.84649010337433	3.59633107954905e-05	Disruptive
C4B63_228g47	1.73024104341704	3.6077636920807e-05	Core
C4B63_3g298	1.5106932607956	3.63336204997522e-05	Disruptive
C4B63_3g956	-1.97358248818714	3.68071497049589e-05	Disruptive
C4B63_67g107	-2.40905997829805	3.72579635894579e-05	Disruptive
C4B63_5g156	-2.02645084309756	3.72692250953869e-05	Disruptive
C4B63_8g197	1.12157468534911	3.73640467762923e-05	Core
C4B63_92g67	1.12764264190326	3.73875447676865e-05	Core
C4B63_113g65	1.46785244180079	3.74360188749673e-05	Core
C4B63_29g135	-1.93764664534653	3.74784852471605e-05	Disruptive
C4B63_12g163	1.24839242079445	3.78763933836e-05	Core
C4B63_15g313	-2.87794796304908	3.82114840984975e-05	Disruptive
C4B63_96g40	-2.32035008248855	3.82558630618799e-05	Disruptive
C4B63_67g78	-3.03117684635533	3.8547294270624e-05	Disruptive
C4B63_37g396	1.46337637507269	3.91493180109711e-05	Core
C4B63_151g43	-1.93739260967711	3.9804706935819e-05	Disruptive
C4B63_109g58	-1.76131877710742	4.04423692324893e-05	Disruptive
C4B63_30g162	1.97312682530119	4.07330040878144e-05	Core
C4B63_95g2	-2.56759270780768	4.08320536261733e-05	Disruptive
C4B63_33g38	-2.3812330927241	4.10797684819377e-05	Disruptive
C4B63_3g937	-2.01433021948857	4.13977338625131e-05	Disruptive
C4B63_60g75	1.78659733039079	4.23536629839636e-05	Core
C4B63_5g72	-1.88245420214745	4.27030877120729e-05	Disruptive
C4B63_5g371	-2.32583287797933	4.27544554772233e-05	Disruptive
C4B63_33g293	-1.4357564894623	4.32329389276126e-05	Disruptive
C4B63_63g27	-1.1120302349215	4.3328054216446e-05	Core
C4B63_3g766	-1.70890055801225	4.36628733571534e-05	Disruptive
C4B63_107g104	-2.81931252891356	4.36628733571534e-05	Disruptive
C4B63_29g287	-3.18134401924429	4.4804755614307e-05	Disruptive
C4B63_26g358	-1.23258777876326	4.5108352441003e-05	Disruptive
C4B63_399g10	-1.13246307685641	4.53540076127905e-05	Disruptive
C4B63_142g5	1.11056302925543	4.55479059665895e-05	Core
C4B63_1g591	-2.61418796332271	4.58545112008532e-05	Disruptive
C4B63_167g31	1.11688749869474	4.6004981327222e-05	Core
C4B63_258g22	-2.20616694936229	4.60104579210998e-05	Core
C4B63_3g447	-3.52080664828635	4.6352130939238e-05	Disruptive
C4B63_87g82	-1.66759872210062	4.64787758343246e-05	Core
C4B63_61g119	1.19219188979619	4.69122083993435e-05	Core
C4B63_44g151	-1.52896619912164	4.71899172980699e-05	Core
C4B63_50g238	-2.75366230214833	4.7614034624631e-05	Disruptive
C4B63_36g52	-1.95558465864215	4.80308986683745e-05	Disruptive
C4B63_75g106	1.18543315608142	4.84384106297662e-05	Core
C4B63_80g67	1.21653601334738	4.84761248678158e-05	Core
C4B63_274g21	-1.66767411380558	4.84761248678158e-05	Core
C4B63_50g170	-1.79381666634052	4.85316370684956e-05	Disruptive
C4B63_16g78	-1.53188681052207	4.88567773854901e-05	Disruptive
C4B63_27g264	-1.98947552061316	4.94036062632268e-05	Disruptive
C4B63_78g5	-21.305763202387	4.9627460964372e-05	Core
C4B63_330g8	1.63580524285409	4.98143714722712e-05	Core
C4B63_19g214	2.11962460465756	5.10705625262157e-05	Core
C4B63_121g79	8.43130279430665	5.1465767242742e-05	Core
C4B63_43g212	1.12227577508169	5.165466117907e-05	Core
C4B63_336g5	1.98114962056633	5.28591925690503e-05	Core
C4B63_6g265	1.05641059496279	5.28712389229161e-05	Core

C4B63_96g27	-1.74724907548592	5.29531807307298e-05	Disruptive
C4B63_44g216	-1.04635186652023	5.35294030247549e-05	Core
C4B63_240g3	-1.73628303768992	5.38911892043626e-05	Disruptive
C4B63_146g45	-2.5280114530442	5.45177405681933e-05	Disruptive
C4B63_73g75	1.70028730861912	5.48797341275753e-05	Core
C4B63_59g201	-2.83836675604634	5.50403006652445e-05	Disruptive
C4B63_85g90	1.04367794259857	5.52089056192916e-05	Core
C4B63_141g24	1.91316262077034	5.52430914884075e-05	Core
C4B63_33g306	-7.19333590573791	5.57989770394634e-05	Disruptive
C4B63_5g699	-2.45077081956437	5.62413100609822e-05	Disruptive
C4B63_13g200	1.07197342646994	5.63509874827133e-05	Core
C4B63_178g47	1.58317805024358	5.64607604179384e-05	Core
C4B63_544g5	1.11597500435286	5.67224783477766e-05	Core
C4B63_34g1235c	3.87348484882369	5.67450305716704e-05	Disruptive
C4B63_125g57	-2.40215690099854	5.73565403096823e-05	Disruptive
C4B63_37g121	-1.37349972516187	5.75426665020252e-05	Disruptive
C4B63_29g85	-2.09810377481466	5.76566253293491e-05	Disruptive
C4B63_22g142	-1.88003462972842	5.8057484367461e-05	Disruptive
C4B63_51g83	-7.32446867944119	5.82212791621096e-05	Core
C4B63_1g1166	-2.2074800213732	5.85852661932271e-05	Disruptive
C4B63_7g1173c	1.41324585541383	5.88568832550262e-05	Core
C4B63_49g199	-1.12569640983381	5.92404407574354e-05	Disruptive
C4B63_136g6	-1.96496259231541	5.9759859099344e-05	Disruptive
C4B63_8g129	-1.8905871808206	6.02390013413564e-05	Disruptive
C4B63_39g69	-1.6905614825934	6.02433900371391e-05	Disruptive
C4B63_164g3	-1.6106979167742	6.05079942938385e-05	Disruptive
C4B63_53g71	-4.94450058756865	6.1412542933703e-05	Disruptive
C4B63_259g135c	1.41846195415999	6.24474940683154e-05	Core
C4B63_260g27	-1.04379526767629	6.24756004486611e-05	Disruptive
C4B63_121g43	-2.66023938103249	6.25237028359537e-05	Disruptive
C4B63_53g60	-2.4363605560941	6.25645774217957e-05	Disruptive
C4B63_108g21	-1.66431245806806	6.25645774217957e-05	Disruptive
C4B63_1g281	-1.63180377233979	6.28426639175957e-05	Disruptive
C4B63_51g221	-1.85830092766535	6.28426639175957e-05	Disruptive
C4B63_53g70	-6.91518973012683	6.28426639175957e-05	Disruptive
C4B63_36g66	-1.95764205127891	6.37495684396647e-05	Disruptive
C4B63_169g40	-1.66313570109576	6.37495684396647e-05	Disruptive
C4B63_36g30	-1.70851018881247	6.42037457590208e-05	Core
C4B63_20g325	-1.49164982071983	6.42116108941486e-05	Core
C4B63_58g70	1.10482511979487	6.54964800310284e-05	Core
C4B63_2g298	-1.37499017602498	6.56076587383614e-05	Core
C4B63_214g7	-1.85394775753506	6.64058772917187e-05	Core
C4B63_11g187	-3.82065467086154	6.74095248480221e-05	Disruptive
C4B63_50g239	-3.09982231709296	6.76502424583428e-05	Disruptive
C4B63_5g590	-1.74501643341682	6.87437056064384e-05	Disruptive
C4B63_111g36	-6.57478091675253	6.90087340469427e-05	Core
C4B63_427g1	1.52186341433689	6.95084020170535e-05	Core
C4B63_60g87	-2.13981373459707	6.98144882742269e-05	Disruptive
C4B63_16g182	-1.4024531104813	6.98582307157767e-05	Disruptive
C4B63_11g145	-2.11633901448043	7.05332767470717e-05	Disruptive
C4B63_134g39	-1.246601914647	7.25772188107153e-05	Disruptive
C4B63_43g159	1.17865940698373	7.29974907790834e-05	Core
C4B63_37g373	-1.03605661103482	7.37564933211362e-05	Core
C4B63_355g21	-7.01883122214859	7.60456091933644e-05	Core
C4B63_29g205	1.14875151205777	7.65765805563259e-05	Core
C4B63_64g35	-6.92774570610061	7.67926445265192e-05	Disruptive
C4B63_45g123	1.12648946158587	7.68224718974307e-05	Core
C4B63_67g24	-2.51670255980469	7.7140044441291e-05	Disruptive
C4B63_223g4	-2.77223654449533	7.74505248746843e-05	Disruptive
C4B63_2g39c	-6.71973410697538	7.79512933105873e-05	Core
C4B63_102g58	-6.67033949886615	7.85042168847582e-05	Core
C4B63_354g13	1.04686721155537	7.85764011512906e-05	Core
C4B63_36g121	-7.00419043426527	7.87752150233142e-05	Disruptive
C4B63_15g158	-2.38365250273312	7.94507104739358e-05	Disruptive

C4B63_15g31	-3.05073893182054	8.07639228351293e-05	Disruptive
C4B63_22g313	1.03226039734525	8.07639228351293e-05	Core
C4B63_29g139	-2.91836778990019	8.08810771878999e-05	Disruptive
C4B63_1g1099	-1.22117172960339	8.14607336884886e-05	Disruptive
C4B63_3g1071	-2.93793186901253	8.14765522040818e-05	Disruptive
C4B63_52g62	1.01446421668649	8.24510012571687e-05	Core
C4B63_5g631	-2.82569102666735	8.33231870930402e-05	Disruptive
C4B63_8g75	-1.51115881894991	8.34285043454278e-05	Disruptive
C4B63_65g73	-2.89624127493979	8.44213677027897e-05	Disruptive
C4B63_3g1092	1.16483357461986	8.52355163903324e-05	Core
C4B63_59g105	-2.008742911681	8.60409976929361e-05	Disruptive
C4B63_11g251	-1.97315146752554	8.6181787867594e-05	Disruptive
C4B63_80g29	-1.01473401617769	8.65490209993951e-05	Core
C4B63_26g152	1.03335032304838	8.79326510170353e-05	Core
C4B63_12g242	1.00053109258904	8.79716137855494e-05	Core
C4B63_105g65	1.45323593964699	8.81366057995751e-05	Core
C4B63_27g331	-2.27185580371975	8.82084103851784e-05	Disruptive
C4B63_37g86	-2.98520250673494	8.83795324359065e-05	Disruptive
C4B63_121g70	-2.156195129725	8.85508349306891e-05	Disruptive
C4B63_37g97	-2.32450378596138	9.02272723875213e-05	Disruptive
C4B63_13g247	-1.04318141813885	9.05853619906181e-05	Core
C4B63_15g122	-2.22841037142355	9.2002784332317e-05	Disruptive
C4B63_79g43	1.48377501741255	9.21644518210448e-05	Core
C4B63_295g15	1.01174952902904	9.29750630118118e-05	Core
C4B63_1g351	-1.7574592653671	9.32400390959903e-05	Disruptive
C4B63_103g72	-2.81856181551944	9.36710727379273e-05	Disruptive
C4B63_1g1219	-1.89351659157916	9.44876014577458e-05	Disruptive
C4B63_112g34	-3.01977401258391	9.50862581021521e-05	Core
C4B63_13g366	-2.77131842391354	9.52737853374608e-05	Disruptive
C4B63_121g1	-2.2814702329438	9.52737853374608e-05	Disruptive
C4B63_241g23	4.43061279818397	9.61249338029919e-05	Disruptive
C4B63_20g747c	1.46688649813603	9.66651723808608e-05	Core
C4B63_266g13	1.0322254002389	9.66651723808608e-05	Core
C4B63_39g106	-2.14597952351617	9.75492938408063e-05	Disruptive
C4B63_18g324	-1.25683197290957	9.77475472933589e-05	Core
C4B63_106g50	-1.9494915480092	9.7995675993327e-05	Disruptive
C4B63_195g15	-1.69880274449987	9.88053333968944e-05	Disruptive
C4B63_115g23	-1.99833764079614	9.90635350964164e-05	Disruptive
C4B63_5g298	-1.77771883629515	9.94738320637142e-05	Disruptive
C4B63_50g180	-1.89290878278918	0.000100475992543776	Disruptive
C4B63_4g439	-1.09666429267402	0.000101714878110908	Core
C4B63_36g55	-2.26272027094035	0.000102258929831798	Disruptive
C4B63_88g101	-1.81110494503423	0.000103195887259857	Disruptive
C4B63_156g4	-1.70660113496991	0.000103680800225378	Disruptive
C4B63_26g143	-1.14384117061991	0.000104425536292707	Disruptive
C4B63_66g17	-2.27741641077096	0.000105133342576877	Disruptive
C4B63_158g45	1.12413586262272	0.000105875333898298	Core
C4B63_403g3	1.2809645550979	0.000105991796924509	Core
C4B63_84g83	-1.16669331183979	0.000107529406219769	Core
C4B63_76g66	1.64310062329426	0.000107776316196424	Core
C4B63_5g28	-1.92869289653638	0.000109823008746709	Disruptive
C4B63_31g241	-1.32049884819196	0.000110778277881999	Disruptive
C4B63_154g3	1.92830449996808	0.000110778277881999	Core
C4B63_299g15	-1.9423170195687	0.000111220811544379	Disruptive
C4B63_124g13	-1.72022954556585	0.000111792811291711	Disruptive
C4B63_53g165	1.8437757489309	0.000112529840104509	Core
C4B63_296g16	-2.40103998687416	0.000112983883967794	Disruptive
C4B63_4g135	1.00366024188839	0.000113033156867611	Core
C4B63_14g204	-1.07112121497675	0.000113903758043001	Core
C4B63_331g5	1.59156302101094	0.000114057307467571	Core
C4B63_15g331	-1.99121792395355	0.000115983125806675	Disruptive
C4B63_1g556	-1.91176264379678	0.000116964510297552	Disruptive
C4B63_27g208	1.18191517238189	0.000119076742369913	Core
C4B63_39g276	-2.18014136500887	0.000119131710708596	Disruptive

C4B63_39g175	-2.22910937264769	0.000119479038006331	Disruptive
C4B63_39g231	-2.54738546554532	0.000120667888076543	Disruptive
C4B63_72g103	1.53251413764791	0.000120861756575092	Core
C4B63_86g44	-1.93935778982822	0.000121173116780208	Disruptive
C4B63_5g642	-2.88036055301937	0.000121713481731049	Disruptive
C4B63_38g191	-1.04103305670578	0.000124061501278269	Disruptive
C4B63_91g42	2.37086970945221	0.000124777289811035	Core
C4B63_36g194	-7.34384582170633	0.000126156354460113	Disruptive
C4B63_276g9	1.49153742780382	0.000128437675598358	Core
C4B63_194g28	1.06189476340546	0.000129709874785792	Core
C4B63_66g54	-1.9659826607726	0.000129950659531401	Disruptive
C4B63_27g167	-1.19462623162558	0.000130658001858041	Core
C4B63_2g81	1.2418084400056	0.000130995957031271	Core
C4B63_29g286	-3.11450765142944	0.000131190133084333	Disruptive
C4B63_34g161	1.35326891118688	0.000131969405736097	Disruptive
C4B63_1g550	-2.09204235718714	0.000132890728194606	Disruptive
C4B63_33g60	-3.13656752949863	0.000133532742008342	Disruptive
C4B63_98g16	-1.00147590656151	0.000133701837641633	Core
C4B63_93g44	1.21816376364841	0.000133716636403978	Core
C4B63_39g138	-1.80440870625864	0.000134085591885513	Core
C4B63_20g343	1.26110295998179	0.000134332335744362	Core
C4B63_38g72	1.36376584231872	0.000134919413412035	Core
C4B63_5g313	-2.16288429848099	0.000136476780361833	Disruptive
C4B63_84g174c	1.37353976394003	0.000136722055097938	Core
C4B63_3g1059	-1.74543297779859	0.000137123645417566	Disruptive
C4B63_5g226	-3.16563461666012	0.000140235847099416	Disruptive
C4B63_371g15	1.3078925048365	0.000140838841688249	Core
C4B63_25g269	-1.29719209551699	0.000141455981788562	Disruptive
C4B63_6g319	-1.30269571892937	0.000143209524081543	Core
C4B63_621g2	1.39415227943485	0.000144603881365434	Core
C4B63_65g69	-3.08949980297133	0.000145117796476533	Disruptive
C4B63_26g42	-2.94906009663004	0.000145589704530316	Disruptive
C4B63_215g13	-2.60926396505061	0.000145631997618547	Disruptive
C4B63_111g8	-2.26171855205659	0.000146103336440981	Disruptive
C4B63_16g134	1.45001484200743	0.000147303283227168	Core
C4B63_10g443	-1.14111912324625	0.000150883409378528	Core
C4B63_50g93	-3.29681172311039	0.000151728688885013	Disruptive
C4B63_207g3	-1.17544220526047	0.000152877010131108	Core
C4B63_14g246	1.24218589644164	0.000153187431258673	Core
C4B63_209g15	1.2431078752349	0.000154973560330535	Core
C4B63_79g1	1.09474021434247	0.000155658612158677	Core
C4B63_33g2	2.36531762135759	0.000156025148434728	Core
C4B63_45g240	-1.13261851252835	0.000156919739561237	Core
C4B63_3g806	-1.56417900824607	0.000159395340348387	Disruptive
C4B63_65g44	-3.90258304702666	0.00016160508990066	Disruptive
C4B63_3g857	1.02020980389684	0.000161684406518802	Disruptive
C4B63_10g535	-2.42947864133918	0.000162358971186183	Disruptive
C4B63_53g227	-3.00054452117973	0.000164569644619579	Disruptive
C4B63_23g4	1.45748175139293	0.000166377383941691	Core
C4B63_24g210	-19.9066443860668	0.000166438592309098	Core
C4B63_3g614	-1.7319818216055	0.000166514162315954	Disruptive
C4B63_4g441	-1.06721276296771	0.000167754551923122	Core
C4B63_60g80	1.18383254347696	0.000169509054480822	Core
C4B63_8g186	-1.15027530529912	0.000172246995237286	Disruptive
C4B63_67g23	-3.56442123976613	0.000172978763757355	Disruptive
C4B63_6g148	1.09844700664484	0.000174205456444431	Core
C4B63_4g356	1.12780517067319	0.000175511928290721	Core
C4B63_1g833	-2.75111352973086	0.000175708500615684	Disruptive
C4B63_103g48	1.78798612398609	0.000175880229274088	Disruptive
C4B63_200g28	-4.70045372761275	0.000176038086051553	Core
C4B63_8g167	-2.10034530551897	0.000178497812635155	Disruptive
C4B63_74g54	-1.65750602662476	0.000179266943523109	Disruptive
C4B63_522nc5	1.47468330430337	0.000179945448005972	Core
C4B63_67g92	-2.68833655231546	0.000180768026995963	Disruptive

C4B63_26g267	1.00224394249069	0.000181133189217499	Core
C4B63_151g38	1.46968142746731	0.000181854277921226	Disruptive
C4B63_71g177	-2.71146533442333	0.000182388928686821	Disruptive
C4B63_143g7	1.28654933840627	0.000182816743149821	Core
C4B63_244g7	1.18174496489923	0.000188380780057345	Disruptive
C4B63_558g9	1.65990785634065	0.000188425062536982	Core
C4B63_34g243	3.16774100728917	0.000190839965685594	Disruptive
C4B63_189g34	1.18279828807474	0.000191343236703019	Core
C4B63_30g254	-2.06013372289483	0.000192161823817375	Core
C4B63_27g253	-1.56926684544373	0.000192924601962129	Disruptive
C4B63_23g195	1.02852811736984	0.000195723250666373	Core
C4B63_1g901	-2.28057965661583	0.000196850981363594	Disruptive
C4B63_262g18	1.55665916605445	0.000197031448186185	Disruptive
C4B63_11g74	1.05418148881017	0.000198790422455348	Core
C4B63_29g292	-1.22380730349783	0.000198790422455348	Disruptive
C4B63_3g737	-4.19907288515587	0.000201351145547685	Disruptive
C4B63_30g291	1.14369803637872	0.000201653228868719	Core
C4B63_145g2	-6.58095385813066	0.00020272662600142	Core
C4B63_112g39	-1.06309322293225	0.000205175399335968	Core
C4B63_67g76	-3.35019226601672	0.000212195489253029	Disruptive
C4B63_3g574	-1.35046824479504	0.000212792784863848	Disruptive
C4B63_288g5	1.07920168533256	0.000214950228997431	Core
C4B63_39g160	-2.04006422402661	0.000215778397647412	Disruptive
C4B63_3g915	-2.05179920056257	0.000217384027896464	Disruptive
C4B63_111g16	-3.25494982030722	0.000217384027896464	Disruptive
C4B63_49g196	-6.31735680858327	0.000217587852469376	Core
C4B63_45g120	1.02852735872615	0.000218489955186511	Core
C4B63_62g19	-4.67642474516178	0.000218540389181474	Disruptive
C4B63_159g2	1.68190940375978	0.000221575449480847	Core
C4B63_71g192	-1.70226627695494	0.000222007450619167	Disruptive
C4B63_5g731	-2.2102498157895	0.000223750022096303	Disruptive
C4B63_1g1094	-2.79592444065048	0.000224437762168504	Disruptive
C4B63_3g618	-1.62663620551232	0.000229105695501897	Disruptive
C4B63_41g121	-1.65966455874026	0.000231140330483669	Core
C4B63_62g181	-6.42068427994952	0.000233459935800207	Disruptive
C4B63_124g6	-1.2263736156717	0.000235317902906115	Core
C4B63_120g26	1.11878817616429	0.000238795997408566	Core
C4B63_1g1323	-6.56610723671518	0.000243538031976905	Disruptive
C4B63_96g94	-6.29665848297502	0.000243931660830551	Disruptive
C4B63_156g3	-2.82550585300827	0.000244365272737919	Disruptive
C4B63_15g485	-3.94660441127978	0.000246547889009696	Disruptive
C4B63_7g417	1.26439828600201	0.000248698292744071	Core
C4B63_455g14	1.69007767493456	0.000250886191129479	Core
C4B63_8g143	-1.08956091913276	0.00025127464835356	Disruptive
C4B63_37g143	-1.43162132980898	0.00025323150967612	Disruptive
C4B63_18g283	1.04822785889839	0.000253245178420168	Core
C4B63_3g899	-2.06243818080496	0.000254394719555757	Disruptive
C4B63_16g79	1.4622437898256	0.000254569688556611	Core
C4B63_40g107	1.43060885657884	0.000254752103755102	Core
C4B63_5g374	-1.60953118853661	0.000257351581215791	Disruptive
C4B63_16g100	1.10633794410678	0.000262742512070784	Core
C4B63_1g585	-1.64129191754691	0.000262790079229107	Disruptive
C4B63_1g196	-1.70285935974096	0.000263315715532407	Disruptive
C4B63_308g1	1.44703747234091	0.000265358124898095	Core
C4B63_22g143	-1.40076433666078	0.000265366216338309	Disruptive
C4B63_15g397	-1.86468056348632	0.000265643252457889	Disruptive
C4B63_67g26	-1.84218568899821	0.000266319864887331	Disruptive
C4B63_20g179	1.17480717804923	0.000267155935873077	Core
C4B63_63g49	1.34210506109388	0.000267678740466909	Core
C4B63_15g260	-2.31731288616309	0.000268179677815151	Disruptive
C4B63_45g175	2.78606742092981	0.000272938342103644	Core
C4B63_65g125	-2.83308286456519	0.000274960706571434	Disruptive
C4B63_2g418	1.12455411108096	0.00027569896085928	Core
C4B63_46g185	-1.30960253460337	0.000279223162861757	Disruptive

C4B63_47g102	1.25485049271197	0.000282578071084383	Core
C4B63_60g190	1.21723583169112	0.000283887150730333	Core
C4B63_54g179	1.1876893913744	0.000285866056846885	Core
C4B63_234g13	-1.11045606501337	0.000288033288628913	Core
C4B63_197g25	-1.12743296359869	0.000290603277939293	Disruptive
C4B63_1g508	-2.04956315241458	0.000292093763559281	Disruptive
C4B63_71g188	-1.67530886679434	0.000292093763559281	Disruptive
C4B63_8g2705c	1.33328887263738	0.000292907872191632	Core
C4B63_71g157	-2.53064109478693	0.000298416933678452	Disruptive
C4B63_202g23	-10.3489268204632	0.000298651839396878	Core
C4B63_139g14	-1.47132039674664	0.000305209108298937	Disruptive
C4B63_122g44	-3.02470227045325	0.000306566932645622	Disruptive
C4B63_8g240	-1.66828665942205	0.000306678005698623	Disruptive
C4B63_3g379	-6.4025845448814	0.000307881591204786	Disruptive
C4B63_8g203	1.42405106686463	0.000314147296263948	Core
C4B63_11g195	-3.29386192717337	0.000317461554112373	Disruptive
C4B63_2g203	1.14961083332091	0.000318965833397994	Core
C4B63_5g495	-1.89533810471561	0.000320309107756923	Disruptive
C4B63_67g210	-2.11822432669711	0.000322224684242253	Disruptive
C4B63_263g5	-6.30517822565499	0.00032324840549913	Core
C4B63_33g67	-1.64928925159513	0.000323741319215302	Disruptive
C4B63_38g147	1.09242123681563	0.000325018278332273	Core
C4B63_1g604	-2.1191083055932	0.000329028695258279	Disruptive
C4B63_137g16	1.65483403853057	0.000330022446543385	Core
C4B63_11g151	-1.41801962153914	0.00033015174713262	Disruptive
C4B63_128g56	-1.51216308587645	0.000331368798277907	Core
C4B63_3g729	1.49649679604775	0.000332488336123465	Disruptive
C4B63_15g321	-2.55238694366345	0.000332839912495617	Disruptive
C4B63_132g20	-3.29282422946593	0.000333363225506737	Disruptive
C4B63_32g177	-1.81550943451333	0.000334282877787231	Disruptive
C4B63_3g863	-2.19126603321633	0.000335874996141763	Disruptive
C4B63_28g334	-2.56835047288124	0.000335975687385623	Core
C4B63_402g14	1.09863455165525	0.000338985788026646	Core
C4B63_9g217	4.24228131660219	0.000339029814687368	Core
C4B63_29g447	-2.6832635416894	0.000339106250405199	Disruptive
C4B63_11g121	-1.96487043474171	0.000341716824784572	Disruptive
C4B63_110g42	1.06778385454845	0.00034185091915975	Core
C4B63_14g192	1.24545156938017	0.000341852237792212	Core
C4B63_111g23	1.81366342329813	0.000348499517437228	Core
C4B63_36g46	-6.42144647132774	0.000350987768475056	Disruptive
C4B63_3g833	-2.34147232259193	0.00035889243898565	Disruptive
C4B63_1g1285	-1.98012809837157	0.000359111217262087	Disruptive
C4B63_224g17	-1.43122283546895	0.000359240025203808	Core
C4B63_7g135	1.05637585603876	0.000362648868525517	Core
C4B63_46g41	1.01625931978447	0.000362937215726368	Core
C4B63_3g1003	-1.75638977118552	0.000363735941081655	Disruptive
C4B63_406g6	1.00297326765688	0.000368114933315336	Core
C4B63_147g44	1.30392911439412	0.000371389066010567	Core
C4B63_29g300	-1.99433388234183	0.000372267926813726	Disruptive
C4B63_189g41	-1.45960010293084	0.000373068846087225	Disruptive
C4B63_66g144	-2.06698799518858	0.00037448430068394	Disruptive
C4B63_45g143	-1.44495703938113	0.000378564279910232	Core
C4B63_3g494	-2.47094745029085	0.00037988154268561	Disruptive
C4B63_66g167	-1.97956842661143	0.000382252626269528	Disruptive
C4B63_2g530	1.12884662451523	0.000385246947194709	Core
C4B63_106g1	1.73757292253992	0.000393073643765628	Core
C4B63_29g184	1.07242075893972	0.000404982786986734	Core
C4B63_39g149	-6.22801999833432	0.000412877860180194	Disruptive
C4B63_53g75	-1.74856956681061	0.000413686110498953	Disruptive
C4B63_235g14	-1.14175912257141	0.000415058937644611	Core
C4B63_52g133	2.04175602211398	0.000417769704365949	Core
C4B63_29g288	-2.05939123850567	0.000422328423428557	Disruptive
C4B63_5g225	-3.2826429111324	0.00042293892199926	Disruptive
C4B63_63g38	-1.07057117175654	0.000428361464591696	Core

C4B63_199g15	1.04102049558466	0.000429952779773705	Core
C4B63_2g736	-1.18462658749959	0.000435028237434981	Core
C4B63_19g257	1.074460155248	0.000435028237434981	Core
C4B63_33g27	-2.61312367585409	0.000435288218776785	Disruptive
C4B63_12g2035c	1.6384362671049	0.00043608698209775	Core
C4B63_69g157	1.08312437433965	0.000443695114499651	Core
C4B63_1g986	-2.49436398489927	0.000444903219430173	Disruptive
C4B63_246g15	1.1132052424229	0.000447701276394627	Core
C4B63_251g5	-1.27772525285696	0.000449694603290438	Core
C4B63_406g5	1.86371800281089	0.000451483523465803	Core
C4B63_308g2	1.45941396801713	0.000453096454118304	Core
C4B63_235g2	2.26195118121264	0.000453352995438267	Core
C4B63_20g176	1.33839204736944	0.000457379897973816	Core
C4B63_125g66	-2.7914272034827	0.000457540566465963	Disruptive
C4B63_256g23	-4.42013252584675	0.000458007598701703	Core
C4B63_34g329	1.40448800121572	0.000461458280127689	Disruptive
C4B63_8g106	-2.05759292151218	0.000461881926842707	Disruptive
C4B63_253g8	1.00078982447655	0.00046418156967193	Core
C4B63_112g71	1.88833725085805	0.000467694465675497	Core
C4B63_115g68	-1.10348059105545	0.000469634635461952	Disruptive
C4B63_25g1482c	1.11741778210772	0.000470079855791384	Core
C4B63_83g91	2.03403924452387	0.000470371420416651	Core
C4B63_152g34	1.31390920897645	0.000473934590543349	Core
C4B63_107g77	-1.60778289614804	0.000478391278166695	Disruptive
C4B63_5g96	-2.43218962856063	0.000481207106783355	Disruptive
C4B63_135g34	-2.52806496364832	0.000482998151561073	Disruptive
C4B63_79g20	1.02785674028127	0.000485323002783754	Core
C4B63_39g61	-2.07177542904912	0.000488377125390544	Disruptive
C4B63_20g635c	1.30511390323296	0.000488416516792905	Core
C4B63_1g1161	-1.53541409090221	0.000494934591384697	Disruptive
C4B63_5g380	-2.07012686389937	0.000497435001094085	Disruptive
C4B63_3g685	-6.33300497431653	0.000508055987871973	Disruptive
C4B63_39g64	-3.36240697914086	0.000508912794387737	Disruptive
C4B63_8g96	-1.57743609749435	0.000517192086449611	Disruptive
C4B63_422g10	1.63388710265107	0.000517192086449611	Core
C4B63_11g370	-2.44612668500909	0.000527315298245579	Disruptive
C4B63_402g15	-1.73312331290691	0.000529964339823655	Core
C4B63_3g846	-3.06602912519199	0.000534229328920228	Disruptive
C4B63_99g88	-6.31712111882972	0.000534430101618892	Disruptive
C4B63_8g243	-3.3603106193227	0.000536396358509518	Disruptive
C4B63_14g193	1.21118300081275	0.000538712667377993	Core
C4B63_22g138	-1.07733150654108	0.000545640403506639	Disruptive
C4B63_13g394	1.36493067016709	0.000551796304420245	Core
C4B63_2nc182	1.1756567932358	0.000555636448928884	Core
C4B63_37g156	-1.67679769831323	0.000555934587250467	Disruptive
C4B63_15g82	-1.90100293293191	0.000564741648530771	Disruptive
C4B63_251g11	1.57542556961277	0.000572683767506427	Core
C4B63_41g299	1.08179249019658	0.00057511078369591	Core
C4B63_208g38	1.49022354785907	0.000575143797838	Core
C4B63_71g130	-2.03226386496095	0.000575632143850704	Disruptive
C4B63_222g3	1.82345658857204	0.000575632143850704	Disruptive
C4B63_4g283	-6.4020522076107	0.000583348123040005	Core
C4B63_208g12	-1.3787471288592	0.000593286278005126	Core
C4B63_5g281	-2.51313327712136	0.000598707397273194	Disruptive
C4B63_100g63	1.13512215679526	0.00060156673806554	Core
C4B63_182g24	1.92077928297004	0.00060156673806554	Core
C4B63_37g134	-2.15584590726579	0.000603833412479039	Disruptive
C4B63_27g320	-3.25940582089809	0.000624592361156938	Disruptive
C4B63_38g297	1.31459445171441	0.000624635340313981	Core
C4B63_11g276	-2.34048122125063	0.000625907193963594	Disruptive
C4B63_42g111	1.09273208871963	0.000625907193963594	Core
C4B63_143g21	1.60066222055351	0.000631170023834531	Core
C4B63_6g155	-1.10571635562776	0.000632480606679757	Core
C4B63_241g12	1.28526129384111	0.000636891634648487	Core

C4B63_39g109	-2.13142013692523	0.00063738058444969	Disruptive
C4B63_226g23	1.64052857188881	0.000640676250113095	Core
C4B63_3g711	-1.77201473918895	0.000653048672358578	Disruptive
C4B63_103g52	-3.9408052779528	0.000655935068553818	Disruptive
C4B63_52g79	-1.01329054491084	0.000657344983151831	Core
C4B63_74g135	-2.10937885818993	0.000657344983151831	Disruptive
C4B63_82g99	-1.36557610312129	0.000667904923549357	Core
C4B63_11g492	-1.34656313485637	0.000668889044248719	Core
C4B63_41g117	-2.54465468037173	0.000672584579134099	Core
C4B63_99g85	-6.42155387906258	0.00067478945643172	Disruptive
C4B63_53g57	-2.76781155067944	0.000682146606805527	Disruptive
C4B63_121g66	-2.14739702412008	0.000700149851995357	Disruptive
C4B63_29g297	-1.47161839739512	0.000703855166853986	Disruptive
C4B63_2g773	1.27750654887076	0.00070935801859472	Core
C4B63_1g367	-1.74261972562978	0.000713154874097269	Disruptive
C4B63_67g183	-2.51629086888426	0.000714555121946085	Disruptive
C4B63_300g102c	2.16620570904985	0.000715558912946271	Disruptive
C4B63_41g129	-1.1548730490452	0.000719267833429313	Core
C4B63_282g21	1.46284356714119	0.000726058456345957	Core
C4B63_119g26	-1.67220855005589	0.000728469716322962	Disruptive
C4B63_236g24	1.04294632188428	0.000732186276374809	Core
C4B63_48g693c	1.2617953417158	0.000735821886115773	Core
C4B63_5g123	-2.00038856497489	0.000741204449955089	Disruptive
C4B63_341g11	-1.15479203937005	0.000741551188253017	Core
C4B63_76g64	1.26613011964359	0.000741666803293342	Core
C4B63_1g555	-2.38015806972881	0.000750479337499609	Disruptive
C4B63_94g86	1.25408534955516	0.000751217850860342	Core
C4B63_99g57	1.17422771214035	0.000755468161121936	Core
C4B63_3g755	-2.28270520828008	0.000763736020351656	Disruptive
C4B63_1g848	-1.58645555979258	0.000765794583069762	Disruptive
C4B63_5g373	-2.1971716694767	0.000782826799929971	Disruptive
C4B63_50g138	2.44413805568853	0.00078368118998802	Disruptive
C4B63_22g159	3.01362157746216	0.000785497087443446	Disruptive
C4B63_192g14	1.60823211187887	0.00079145238002501	Core
C4B63_319g11	-3.96601253215812	0.000800607020105841	Disruptive
C4B63_36g124	-3.60543947554806	0.000801256003855048	Disruptive
C4B63_2g41	1.15474694780789	0.000801614900898217	Core
C4B63_15g272	1.14842936613779	0.000801614900898217	Core
C4B63_29g38	-1.62500403165911	0.000801614900898217	Disruptive
C4B63_74g16	-3.81105717188994	0.00081074464978005	Disruptive
C4B63_1g1200	-2.48441614772718	0.000812353079773216	Disruptive
C4B63_306g8	2.02207625747765	0.000820002720031431	Disruptive
C4B63_544g6	1.64061207539565	0.000824465882246181	Core
C4B63_65g103	-2.9306145893458	0.000833066298228237	Disruptive
C4B63_3g667	-3.79515251444456	0.000837839146906357	Disruptive
C4B63_53g177	-1.85802926037277	0.000838329050028977	Disruptive
C4B63_71g132	-2.08391414312444	0.000841570157973216	Disruptive
C4B63_34g387	1.05380957926201	0.000845402427743979	Core
C4B63_425g13	-1.41409509625322	0.000849029894660043	Core
C4B63_153g21	-3.02469357389908	0.000856606432267536	Core
C4B63_6g285	-1.09058316961614	0.000865131372316282	Core
C4B63_338g3	-3.19949328289065	0.00086663768621307	Disruptive
C4B63_306g9	1.92268173463476	0.000868391705746286	Disruptive
C4B63_218g31	-6.35154197638199	0.00087085493804803	Core
C4B63_62g13	1.53676009717143	0.000873224079569268	Core
C4B63_202g13	1.01729952207994	0.000884278128387894	Core
C4B63_106g46	-1.89159464257711	0.000895494757227532	Disruptive
C4B63_350g13	-1.8047095444395	0.00090788932978588	Core
C4B63_74g50	-6.27787821695958	0.000921919078585219	Disruptive
C4B63_9g335	-1.19766682135182	0.000931238716314371	Core
C4B63_35g113	1.25845325879516	0.000933154923326336	Core
C4B63_131g57	-6.12739113829955	0.000936490509289182	Disruptive
C4B63_93g38	1.2565055727109	0.000954930405552499	Core
C4B63_33g313	-1.93812199535604	0.000956472429172695	Disruptive

C4B63_2g181	1.12270819103079	0.000960094150249567	Core
C4B63_55g101	-1.19909311090332	0.000966752866277743	Core
C4B63_60g128	1.479930849294	0.000967527530239788	Core
C4B63_36g116	-3.75008009319335	0.000969148354301857	Disruptive
C4B63_103g69	-3.92706241711	0.000971280141077603	Disruptive
C4B63_71g133	-1.8204735650114	0.000971429727475997	Disruptive
C4B63_16g1	-5.85774696758117	0.000983737359052235	Core
C4B63_225g31	1.02110352144517	0.000983737359052235	Core
C4B63_64g184	-1.79367943620565	0.000984414210067599	Disruptive
C4B63_107g81	-3.47834835951448	0.000987506247832073	Disruptive
C4B63_91g75	-1.79805569715527	0.000997078731234406	Disruptive

GeneID	log2FoldChange	padj	Compartment
C4B63_522nc5	3.61866281854828	5.71060504264224e-19	Core
C4B63_333g17	13.8201701775769	1.55437516301869e-17	Core
C4B63_29g4	1.68868939351653	3.66843037413173e-16	Core
C4B63_406g4	2.2686163397494	3.66843037413173e-16	Core
C4B63_275g6	1.40948812361575	9.90591370833609e-16	Core
C4B63_101g4	2.12335161541135	4.52708773185163e-15	Core
C4B63_23g109	1.3679984618195	7.16405531022368e-15	Core
C4B63_20g302	1.51135181215174	9.93193294153573e-15	Core
C4B63_49g71	1.94568052243697	9.93193294153573e-15	Core
C4B63_16g331	1.3756427453948	1.63166286349422e-14	Core
C4B63_104g78	1.3320420620036	1.90355200826091e-14	Core
C4B63_18g221	1.16177919855659	5.87102716730216e-14	Core
C4B63_34g364	1.41078763521255	5.87102716730216e-14	Core
C4B63_83g85	1.08653986365874	5.87102716730216e-14	Core
C4B63_238g9	1.52003724158508	6.1720894710356e-13	Core
C4B63_212g8	-11.2770920036881	1.30344995947718e-11	Core
C4B63_52g93	1.44519033872718	2.33090983691837e-11	Disruptive
C4B63_3g1101	1.16633029709743	2.9367164144594e-11	Core
C4B63_9g322	1.18696953141116	2.9367164144594e-11	Core
C4B63_34g302	1.72794157102467	2.9367164144594e-11	Core
C4B63_46g43	1.15119680655692	3.08867507666229e-11	Core
C4B63_45g236	1.17735547093554	5.61314737566548e-11	Core
C4B63_52g170	1.50561578599733	6.00085914354324e-11	Core
C4B63_44g146	1.23622107057945	7.84956426506833e-11	Core
C4B63_46g53	1.24592339577901	1.39835933760774e-10	Core
C4B63_55g181	1.16345775640648	2.18836616918849e-10	Core
C4B63_70g95	1.3549360306531	3.32988650882125e-10	Core
C4B63_95g19	1.11348320619776	3.68040653241664e-10	Core
C4B63_44g179	1.24866658091507	1.08900786356216e-09	Core
C4B63_48g133	1.60101131966817	1.16331202474702e-09	Core
C4B63_397g11	1.38733304776282	1.16331202474702e-09	Core
C4B63_41g197	1.17493832454228	1.30431908963659e-09	Core
C4B63_44g221	1.1440280919289	2.14241581554162e-09	Core
C4B63_19g91	1.33702604246651	2.33387741774822e-09	Core
C4B63_55g180	1.58323080524821	5.5311264066342e-09	Core
C4B63_40g92	1.12305354641641	5.75553049636969e-09	Core
C4B63_86g56	-3.95999536514747	6.13990723920722e-09	Core
C4B63_16g95	1.48615309936501	1.39594659483679e-08	Core
C4B63_55g179	1.05732070069427	1.42843649759578e-08	Core
C4B63_4g349	1.19502271930054	1.74243980967235e-08	Core
C4B63_25g199	2.45727693042953	1.74243980967235e-08	Disruptive
C4B63_21g282	1.45344699201005	2.48486009269161e-08	Core
C4B63_84g82	1.00364737467208	3.02012896579702e-08	Core
C4B63_122g1	1.55172790288704	3.50054281368257e-08	Core
C4B63_160g33	1.08777611648255	3.58754973148097e-08	Core
C4B63_85g83	1.27570659429917	4.39828769594672e-08	Core
C4B63_49g176	1.43252307302078	5.73594589195463e-08	Core
C4B63_17g244	1.24344354216688	1.17752488049397e-07	Core
C4B63_24g236	1.88871406375155	1.17752488049397e-07	Core
C4B63_20g10	1.29861537205663	1.44345122396289e-07	Core
C4B63_55g56	1.54783756361303	1.63352633495014e-07	Core
C4B63_81g70	5.21250997419088	1.63850089305556e-07	Core
C4B63_114g34	1.04091196993949	1.78322899747821e-07	Core
C4B63_43g161	1.07254258791842	1.98188371021439e-07	Core
C4B63_603g9	-9.30738673093395	2.1846210219673e-07	Core
C4B63_90g49	1.42287971803066	3.10886779923471e-07	Core
C4B63_113g27	1.84809380944912	3.10886779923471e-07	Core
C4B63_148g27	-8.7569116104012	4.0581947325848e-07	Core
C4B63_32g233	1.47776231724124	4.46142779024063e-07	Core
C4B63_45g132	1.3099573580829	5.5635894868088e-07	Core
C4B63_54g35	1.17846616908936	5.77717296331964e-07	Core
C4B63_45g235	1.09999138698467	6.04909174885113e-07	Core
C4B63_6g514	1.03729841879821	7.0360993118381e-07	Core

C4B63_54g26	1.45278111164991	7.10251434237756e-07	Core
C4B63_9g319	1.35080514166862	7.22670510704767e-07	Core
C4B63_6g129	-8.90875017737297	7.43442737248608e-07	Core
C4B63_387g12	-8.50068147093811	8.76071663718796e-07	Disruptive
C4B63_102g33	1.07456515585839	9.1843069980615e-07	Core
C4B63_61g136	1.31426759780358	1.01911476344833e-06	Core
C4B63_14g246	1.72743825340268	1.05664720329308e-06	Core
C4B63_81g64	-1.01469624291915	1.1663164170104e-06	Core
C4B63_55g53	-8.54914192826536	1.44608970794791e-06	Core
C4B63_25g200	2.41238083425208	1.72649646525515e-06	Disruptive
C4B63_8g64	1.41870617309819	1.97535372901722e-06	Core
C4B63_32g229	1.59869145629587	2.22336596533132e-06	Core
C4B63_70g52	2.77058429403255	2.3555243447238e-06	Core
C4B63_113g25	1.24824928869326	2.6355985322721e-06	Core
C4B63_27g191	1.22840281794039	2.7558600975565e-06	Core
C4B63_144g22	-8.42017328961314	2.7558600975565e-06	Core
C4B63_24g194	1.84327689428571	2.8462284548877e-06	Core
C4B63_113g62	1.49943626702621	3.72886375799432e-06	Core
C4B63_275g7	1.14598889792131	3.72886375799432e-06	Core
C4B63_225g28	-8.23588992137308	4.38457853163841e-06	Core
C4B63_42g111	1.59230389667905	4.49892109735698e-06	Core
C4B63_34g284	1.01199107584836	4.75651426335093e-06	Core
C4B63_97g35	-8.17933749639651	4.95777537565157e-06	Core
C4B63_98g8	1.46383571688856	5.19530200418593e-06	Disruptive
C4B63_51g92	1.6849871212972	5.81197955417417e-06	Core
C4B63_52g135	1.36496077576863	6.02941899527531e-06	Core
C4B63_58g130	1.11415390778634	6.07746934392451e-06	Core
C4B63_10g455	1.14799384506589	6.23012462529355e-06	Core
C4B63_138g37	1.20375517137701	6.48386212655612e-06	Core
C4B63_25g217	1.02583540120098	6.80813398217806e-06	Core
C4B63_74g4	1.39230587716225	1.17159523827319e-05	Core
C4B63_111g36	-7.97917564289986	1.18750331521241e-05	Core
C4B63_34g1223c	-1.3995090403121	1.20212465800415e-05	Disruptive
C4B63_7g343	1.29372302666477	1.49864071389201e-05	Core
C4B63_12g405	1.01734749006804	1.86967779881397e-05	Core
C4B63_54g62	1.32319009501662	1.98993432807131e-05	Core
C4B63_7g340	1.3388906668908	2.25241086727572e-05	Core
C4B63_19g211	1.38839594669872	2.37353244710909e-05	Core
C4B63_114g38	1.14076162376002	2.37353244710909e-05	Core
C4B63_470g6	-7.99260966397904	2.37353244710909e-05	Core
C4B63_10g480	1.32498255589477	2.74819063265615e-05	Core
C4B63_300g17	1.16480982853316	2.75883869082458e-05	Core
C4B63_40g110	1.49323247490135	3.04717392061982e-05	Core
C4B63_51g217	1.37125737497208	3.11694770364869e-05	Disruptive
C4B63_45g234	1.03133274023885	3.17341329861473e-05	Core
C4B63_56g41	1.62729103497403	3.27066913774918e-05	Core
C4B63_615g5	2.33039800146295	3.52434540716489e-05	Core
C4B63_10g512	1.18029382406626	3.63492153629063e-05	Core
C4B63_23g122	1.13778415779372	5.40445919056139e-05	Core
C4B63_72g84	1.48484910431079	5.60830393055001e-05	Core
C4B63_10g454	1.00643408888933	6.08490777814781e-05	Core
C4B63_130g19	-1.15493449032871	6.10647361013364e-05	Disruptive
C4B63_122g20	1.35669325541933	6.12710861276864e-05	Core
C4B63_139g8	-7.33084290453344	6.30506456817183e-05	Disruptive
C4B63_19g254	-1.64488346786997	6.71563721302314e-05	Core
C4B63_45g104	1.00064169929226	6.87699112129496e-05	Core
C4B63_15g5	1.19246546744182	7.82568343156965e-05	Core
C4B63_40g112	1.10267669361102	8.47531580721082e-05	Core
C4B63_45g103	1.10259403683034	8.71473528507197e-05	Core
C4B63_49g113	1.07370725337238	8.71473528507197e-05	Core
C4B63_34g1236c	-1.03701765794298	8.83863978178904e-05	Disruptive
C4B63_528g4	7.75812389885422	8.83863978178904e-05	Core
C4B63_102g58	-7.41749048192275	9.84063698404277e-05	Core
C4B63_322g10	-7.42575590095612	0.000102377629805471	Core

C4B63_34g304	1.45891131911855	0.000104827381825945	Core
C4B63_5g419	1.53473881870887	0.000131585333521056	Core
C4B63_25g313	1.05139581732838	0.000146460804337244	Core
C4B63_94g89	2.10126527989458	0.000146460804337244	Disruptive
C4B63_22g75	1.19923234790685	0.000147981158758713	Core
C4B63_93g9	-7.08776545974311	0.000170014015574688	Disruptive
C4B63_24g215	1.05378310008608	0.000176548097855264	Core
C4B63_2g39c	-7.24729826603324	0.000188243634796609	Core
C4B63_19g221	1.24481168306303	0.000221609990448985	Core
C4B63_52g37	1.05693504411907	0.000293853177452528	Core
C4B63_266g12	-6.84967184835901	0.000359900215065196	Core
C4B63_83g92	1.27053716167284	0.000372044129419711	Core
C4B63_26g252	1.143609610341	0.000376678781187898	Core
C4B63_2g292	1.0121991522067	0.0004184194461304	Core
C4B63_202g23	-11.5884289470055	0.000434486764497125	Core
C4B63_147g45	1.15380592760354	0.000443784648262725	Core
C4B63_40g19	1.43613018285281	0.000451491165860059	Core
C4B63_51g213	1.1047703356327	0.000453725667146977	Disruptive
C4B63_66g52	-6.74849847104102	0.000469875616923866	Disruptive
C4B63_295g15	1.06630090930231	0.000469875616923866	Core
C4B63_2g50	1.498331542365	0.000474866732254665	Core
C4B63_32g226	1.35777441972794	0.00048625556573885	Core
C4B63_19g208	1.18701986207536	0.000505960204840947	Core
C4B63_19g216	1.53097136530507	0.000509983133574061	Core
C4B63_14g141	1.17030091245446	0.000580913196848102	Core
C4B63_19g27	1.19663312363348	0.000580913196848102	Core
C4B63_31g89	1.0183435096792	0.000582504458046525	Core
C4B63_79g1	1.15897713391776	0.000610719677802393	Core
C4B63_21g281	1.04959462763105	0.000638547972190967	Core
C4B63_241g7	-1.03028280752531	0.000679883055823383	Core
C4B63_19g215	1.35250901964965	0.000681341225053781	Core
C4B63_76g18	1.21649922982145	0.000697075251333312	Core
C4B63_355g21	-7.03897926506789	0.000699412270079334	Core
C4B63_47g117	1.55533343885568	0.000773298814636973	Core
C4B63_79g30	1.00521389138549	0.000797846292170998	Core
C4B63_63g27	-1.07380025975665	0.000898435727075066	Core
C4B63_7g209	1.26262978568208	0.000978537301380068	Core
C4B63_24g210	-20.439162360129	0.000988775395233924	Core
C4B63_41g218	1.09700192058325	0.000988775395233924	Core

Chr	Start	End	Region length (nt)	Region length (kb)	Genome context
TcBrA4_Chr1	0	27266	27267	27	TAD + SSR
TcBrA4_Chr1	110076	174717	64642	65	TAD
TcBrA4_Chr1	207093	213712	6620	7	Unknown internal
TcBrA4_Chr1	255291	337670	82380	82	TAD + SSR
TcBrA4_Chr1	460256	473018	12763	13	SSR
TcBrA4_Chr1	580612	606659	26048	26	TAD + SSR
TcBrA4_Chr1	636154	640071	3918	4	TAD + SSR
TcBrA4_Chr1	661683	662502	820	1	Unknown internal
TcBrA4_Chr1	903411	918092	14682	15	TAD
TcBrA4_Chr1	934571	952267	17697	18	Unknown internal
TcBrA4_Chr1	999286	1002507	3222	3	Unknown internal
TcBrA4_Chr1	1059947	1096482	36536	37	SSR
TcBrA4_Chr1	1166652	1216606	49955	50	TAD
TcBrA4_Chr1	1311817	1321010	9194	9	TAD
TcBrA4_Chr1	1418535	1463745	45211	45	TAD
TcBrA4_Chr1	1784342	1795804	11463	11	TAD + SSR
TcBrA4_Chr1	1817259	1826660	9402	9	TAD + SSR
TcBrA4_Chr1	1982973	1987760	4788	5	TAD
TcBrA4_Chr1	2088680	2099245	10566	11	TAD
TcBrA4_Chr1	2207238	2208940	1703	2	SSR
TcBrA4_Chr1	2252515	2253111	597	1	Unknown internal
TcBrA4_Chr1	2554084	2554978	895	1	SSR
TcBrA4_Chr1	2576710	2584001	7292	7	Unknown internal
TcBrA4_Chr1	2689927	2703204	13278	13	TAD + SSR
TcBrA4_Chr4	619	10092	9474	9	SSR
TcBrA4_Chr4	27165	34106	6942	7	Unknown internal
TcBrA4_Chr4	167632	175011	7380	7	TAD
TcBrA4_Chr4	234032	277578	43547	44	TAD
TcBrA4_Chr4	459395	465748	6354	6	Unknown internal
TcBrA4_Chr4	485910	504856	18947	19	TAD
TcBrA4_Chr4	686913	687821	909	1	Unknown internal
TcBrA4_Chr4	730315	746222	15908	16	TAD
TcBrA4_Chr4	762496	775268	12773	13	Unknown internal
TcBrA4_Chr4	845685	863418	17734	18	TAD
TcBrA4_Chr4	979666	997775	18110	18	TAD
TcBrA4_Chr4	1009208	1048768	39561	40	TAD
TcBrA4_Chr4	1132384	1147788	15405	15	SSR
TcBrA4_Chr4	1205080	1214798	9719	10	TAD
TcBrA4_Chr4	1330393	1345532	15140	15	TAD
TcBrA4_Chr4	1459065	1476434	17370	17	TAD + SSR
TcBrA4_Chr4	1533049	1553834	20786	21	TAD
TcBrA4_Chr4	1582988	1588920	5933	6	Unknown internal
TcBrA4_Chr5	9192	16162	6971	7	Unknown internal
TcBrA4_Chr5	82991	103045	20055	20	TAD + SSR
TcBrA4_Chr5	170982	171506	525	1	Unknown internal
TcBrA4_Chr5	227206	290179	62974	63	TAD + SSR
TcBrA4_Chr5	325728	335783	10056	10	Unknown internal
TcBrA4_Chr5	407828	413891	6064	6	TAD + SSR
TcBrA4_Chr5	452977	461240	8264	8	TAD
TcBrA4_Chr5	551409	554965	3557	4	Unknown internal
TcBrA4_Chr5	576442	582071	5630	6	Unknown internal
TcBrA4_Chr5	596731	597643	913	1	TAD
TcBrA4_Chr5	616575	618552	1978	2	TAD
TcBrA4_Chr5	699662	737552	37891	38	TAD
TcBrA4_Chr5	768259	782927	14669	15	TAD
TcBrA4_Chr5	792578	794952	2375	2	Unknown internal

TcBrA4_Chr5	818824	821181	2358	2	Unknown internal
TcBrA4_Chr5	853454	867314	13861	14	TAD + SSR
TcBrA4_Chr5	1101166	1115456	14291	14	TAD + SSR
TcBrA4_Chr5	1296190	1296924	735	1	Unknown internal
TcBrA4_Chr5	1319102	1340319	21218	21	TAD
TcBrA4_Chr5	1361738	1371439	9702	10	SSR
TcBrA4_Chr5	1385189	1391357	6169	6	Unknown internal
TcBrA4_Chr5	1401282	1404483	3202	3	SSR
TcBrA4_Chr7	256898	269914	13017	13	TAD
TcBrA4_Chr7	311425	311927	503	1	SSR
TcBrA4_Chr7	451601	463111	11511	12	TAD + SSR
TcBrA4_Chr7	521590	523308	1719	2	Unknown internal
TcBrA4_Chr7	551573	552132	560	1	SSR
TcBrA4_Chr7	656554	676940	20387	20	TAD
TcBrA4_Chr7	984891	989135	4245	4	TAD
TcBrA4_Chr7	1029266	1033242	3977	4	Unknown internal
TcBrA4_Chr7	1063101	1064144	1044	1	Unknown internal
TcBrA4_Chr7	1293357	1302690	9334	9	SSR
TcBrA4_Chr8	76898	108662	31765	32	SSR
TcBrA4_Chr8	122214	136185	13972	14	TAD + SSR
TcBrA4_Chr8	151876	159700	7825	8	Unknown internal
TcBrA4_Chr8	353654	370498	16845	17	TAD + SSR
TcBrA4_Chr8	418265	424025	5761	6	Unknown internal
TcBrA4_Chr8	461815	503818	42004	42	TAD
TcBrA4_Chr8	567068	579460	12393	12	TAD
TcBrA4_Chr8	592574	593340	767	1	Unknown internal
TcBrA4_Chr8	746594	791072	44479	44	TAD
TcBrA4_Chr8	983090	999607	16518	17	Unknown internal
TcBrA4_Chr8	1023004	1043292	20289	20	TAD + SSR
TcBrA4_Chr8	1056237	1062531	6295	6	TAD + SSR
TcBrA4_Chr8	1200122	1206751	6630	7	SSR
TcBrA4_Chr8	1334030	1336822	2793	3	TAD
TcBrA4_Chr10	58146	67405	9260	9	Unknown internal
TcBrA4_Chr10	99044	112680	13637	14	TAD
TcBrA4_Chr10	294683	314454	19772	20	TAD
TcBrA4_Chr10	353375	353911	537	1	Unknown internal
TcBrA4_Chr10	559444	578764	19321	19	TAD
TcBrA4_Chr10	648800	658865	10066	10	TAD
TcBrA4_Chr10	701922	728903	26982	27	TAD
TcBrA4_Chr10	778262	800044	21783	22	TAD + SSR
TcBrA4_Chr10	826462	827181	720	1	Unknown internal
TcBrA4_Chr10	1059079	1072906	13828	14	TAD
TcBrA4_Chr14	106871	132441	25571	26	TAD
TcBrA4_Chr14	281512	294765	13254	13	TAD
TcBrA4_Chr14	649416	681758	32343	32	TAD + SSR
TcBrA4_Chr14	709695	733384	23690	24	TAD + SSR
TcBrA4_Chr14	924466	926821	2356	2	TAD + SSR
TcBrA4_Chr15	211277	212148	872	1	Unknown internal
TcBrA4_Chr15	269697	270543	847	1	TAD
TcBrA4_Chr15	548367	548890	524	1	TAD
TcBrA4_Chr15	627546	640696	13151	13	TAD
TcBrA4_Chr15	687517	698519	11003	11	SSR
TcBrA4_Chr15	762770	776007	13238	13	TAD
TcBrA4_Chr15	880721	902878	22158	22	TAD + SSR
TcBrA4_Chr15	913915	934171	20257	20	Unknown internal
TcBrA4_Chr16	406961	435478	28518	29	TAD
TcBrA4_Chr16	509041	509577	537	1	TAD

TcBrA4_Chr16	781882	796004	14123	14	TAD + SSR
TcBrA4_Chr16	807841	813037	5197	5	SSR
TcBrA4_Chr16	872460	886076	13617	14	TAD + SSR
TcBrA4_Chr17	75247	76671	1425	1	TAD
TcBrA4_Chr17	113183	113723	541	1	Unknown internal
TcBrA4_Chr17	127137	127685	549	1	Unknown internal
TcBrA4_Chr17	318631	325985	7355	7	SSR
TcBrA4_Chr17	423958	429799	5842	6	TAD
TcBrA4_Chr17	491320	491901	582	1	TAD
TcBrA4_Chr17	653757	661670	7914	8	Unknown internal
TcBrA4_Chr17	669019	681901	12883	13	SSR
TcBrA4_Chr17	711521	719157	7637	8	TAD
TcBrA4_Chr17	766648	778442	11795	12	SSR
TcBrA4_Chr17	805516	829067	23552	24	TAD + SSR
TcBrA4_Chr17	861762	874317	12556	13	Unknown internal
TcBrA4_Chr18	16239	23884	7646	8	SSR
TcBrA4_Chr18	28159	33545	5387	5	SSR
TcBrA4_Chr18	88010	113335	25326	25	TAD
TcBrA4_Chr18	191423	208205	16783	17	TAD
TcBrA4_Chr18	232858	237898	5041	5	Unknown internal
TcBrA4_Chr18	242377	244780	2404	2	Unknown internal
TcBrA4_Chr18	297784	324350	26567	27	TAD
TcBrA4_Chr18	341065	345588	4524	5	SSR
TcBrA4_Chr18	474833	485280	10448	10	SSR
TcBrA4_Chr18	558819	566816	7998	8	Unknown internal
TcBrA4_Chr18	751918	753747	1830	2	Unknown internal
TcBrA4_Chr18	791688	807481	15794	16	TAD
TcBrA4_Chr22	245134	255990	10857	11	TAD
TcBrA4_Chr22	350984	375044	24061	24	TAD + SSR
TcBrA4_Chr22	437435	441913	4479	4	TAD
TcBrA4_Chr22	485773	491056	5284	5	SSR
TcBrA4_Chr22	644943	645547	605	1	TAD
TcBrA4_Chr23	94021	108846	14826	15	TAD
TcBrA4_Chr23	210238	250504	40267	40	TAD
TcBrA4_Chr23	487641	492191	4551	5	SSR
TcBrA4_Chr23	511438	517321	5884	6	Unknown internal
TcBrA4_Chr23	564429	577544	13116	13	TAD
TcBrA4_Chr24	124991	134780	9790	10	Unknown internal
TcBrA4_Chr24	146275	170425	24151	24	TAD + SSR
TcBrA4_Chr24	195283	203610	8328	8	Unknown internal
TcBrA4_Chr24	475627	486752	11126	11	TAD
TcBrA4_Chr24	551931	583062	31132	31	TAD
TcBrA4_Chr24	628752	668887	40136	40	TAD
TcBrA4_Chr24	716268	732505	16238	16	TAD
TcBrA4_Chr24	756600	758593	1994	2	SSR
TcBrA4_Chr28	74787	131363	56577	57	TAD + SSR
TcBrA4_Chr28	150172	158171	8000	8	Unknown internal
TcBrA4_Chr28	220029	236813	16785	17	TAD + SSR
TcBrA4_Chr28	344971	361018	16048	16	TAD
TcBrA4_Chr28	479026	490772	11747	12	TAD + SSR
TcBrA4_Chr28	504353	529045	24693	25	TAD + SSR
TcBrA4_Chr28	559209	560648	1440	1	SSR
TcBrA4_Chr28	631083	641735	10653	11	Unknown internal
TcBrA4_Chr28	675098	687404	12307	12	TAD
			Average length (kb) =	**13,87**	

	%	
SSR	16	35
CFD + SSR	19	
CFD	38	65
Unknown internal	27	

Chr	Start	End	Region length (nt)	Region length (kb)	Number of genes
TcBrA4_Chr1	27266	110076	82811	83	38
TcBrA4_Chr1	174717	207093	32377	32	9
TcBrA4_Chr1	213712	255291	41580	42	21
TcBrA4_Chr1	337670	460256	122587	123	60
TcBrA4_Chr1	473018	580612	107595	108	43
TcBrA4_Chr1	606659	636154	29496	29	16
TcBrA4_Chr1	640071	661683	21613	22	8
TcBrA4_Chr1	662502	903411	240910	241	106
TcBrA4_Chr1	918092	934571	16480	16	2
TcBrA4_Chr1	952267	999286	47020	47	18
TcBrA4_Chr1	1002507	1059947	57441	57	23
TcBrA4_Chr1	1096482	1166652	70171	70	35
TcBrA4_Chr1	1216606	1311817	95212	95	48
TcBrA4_Chr1	1321010	1418535	97526	98	30
TcBrA4_Chr1	1463745	1784342	320598	321	153
TcBrA4_Chr1	1795804	1817259	21456	21	10
TcBrA4_Chr1	1826660	1982973	156314	156	76
TcBrA4_Chr1	1987760	2088680	100921	101	55
TcBrA4_Chr1	2099245	2207238	107994	108	56
TcBrA4_Chr1	2208940	2252515	43576	44	21
TcBrA4_Chr1	2253111	2554084	300974	301	153
TcBrA4_Chr1	2554978	2576710	21733	22	16
TcBrA4_Chr1	2584001	2689927	105927	106	40
TcBrA4_Chr1	2703204	2738928	35725	36	11
TcBrA4_Chr4	0	619	620	1	2
TcBrA4_Chr4	10092	27165	17074	17	6
TcBrA4_Chr4	34106	167632	133527	134	77
TcBrA4_Chr4	175011	234032	59022	59	33
TcBrA4_Chr4	277578	459395	181818	182	84
TcBrA4_Chr4	465748	485910	20163	20	11
TcBrA4_Chr4	504856	686913	182058	182	81
TcBrA4_Chr4	687821	730315	42495	42	14
TcBrA4_Chr4	746222	762496	16275	16	11
TcBrA4_Chr4	775268	845685	70418	70	41
TcBrA4_Chr4	863418	979666	116249	116	55
TcBrA4_Chr4	997775	1009208	11434	11	2
TcBrA4_Chr4	1048768	1132384	83617	84	36
TcBrA4_Chr4	1147788	1205080	57293	57	20
TcBrA4_Chr4	1214798	1330393	115596	116	47
TcBrA4_Chr4	1345532	1459065	113534	114	53
TcBrA4_Chr4	1476434	1533049	56616	57	27
TcBrA4_Chr4	1553834	1582988	29155	29	13
TcBrA4_Chr4	1588920	1618606	29687	30	7
TcBrA4_Chr5	0	9192	9193	9	2
TcBrA4_Chr5	16162	82991	66830	67	33
TcBrA4_Chr5	103045	170982	67938	68	31
TcBrA4_Chr5	171506	227206	55701	56	28
TcBrA4_Chr5	290179	325728	35550	36	17
TcBrA4_Chr5	335783	407828	72046	72	26
TcBrA4_Chr5	413891	452977	39087	39	20
TcBrA4_Chr5	461240	551409	90170	90	31
TcBrA4_Chr5	554965	576442	21478	21	9
TcBrA4_Chr5	582071	596731	14661	15	8
TcBrA4_Chr5	597643	616575	18933	19	11
TcBrA4_Chr5	618552	699662	81111	81	36
TcBrA4_Chr5	737552	768259	30708	31	14

TcBrA4_Chr5	782927	792578	9652	10	6
TcBrA4_Chr5	794952	818824	23873	24	12
TcBrA4_Chr5	821181	853454	32274	32	14
TcBrA4_Chr5	867314	1101166	233853	234	106
TcBrA4_Chr5	1115456	1296190	180735	181	78
TcBrA4_Chr5	1296924	1319102	22179	22	17
TcBrA4_Chr5	1340319	1361738	21420	21	12
TcBrA4_Chr5	1371439	1385189	13751	14	11
TcBrA4_Chr5	1391357	1401282	9926	10	8
TcBrA4_Chr5	1404483	1444478	39996	40	10
TcBrA4_Chr5	1488859	1492459	3601	4	2
TcBrA4_Chr7	0	256898	256899	257	114
TcBrA4_Chr7	269914	311425	41512	42	27
TcBrA4_Chr7	311927	451601	139675	140	59
TcBrA4_Chr7	463111	521590	58480	58	27
TcBrA4_Chr7	523308	551573	28266	28	17
TcBrA4_Chr7	552132	656554	104423	104	45
TcBrA4_Chr7	676940	984891	307952	308	135
TcBrA4_Chr7	989135	1029266	40132	40	16
TcBrA4_Chr7	1033242	1063101	29860	30	15
TcBrA4_Chr7	1064144	1293357	229214	229	99
TcBrA4_Chr7	1302690	1340100	37411	37	14
TcBrA4_Chr7	1340713	1356690	15978	16	7
TcBrA4_Chr7	1357334	1369405	12072	12	8
TcBrA4_Chr8	39503	76898	37396	37	22
TcBrA4_Chr8	108662	122214	13553	14	6
TcBrA4_Chr8	136185	151876	15692	16	8
TcBrA4_Chr8	159700	353654	193955	194	73
TcBrA4_Chr8	370498	418265	47768	48	18
TcBrA4_Chr8	424025	461815	37791	38	21
TcBrA4_Chr8	503818	567068	63251	63	31
TcBrA4_Chr8	579460	592574	13115	13	5
TcBrA4_Chr8	593340	746594	153255	153	67
TcBrA4_Chr8	791072	983090	192019	192	84
TcBrA4_Chr8	999607	1023004	23398	23	3
TcBrA4_Chr8	1043292	1056237	12946	13	0
TcBrA4_Chr8	1062531	1200122	137592	138	62
TcBrA4_Chr8	1206751	1334030	127280	127	61
TcBrA4_Chr10	15558	44017	28460	28	12
TcBrA4_Chr10	44589	58146	13558	14	4
TcBrA4_Chr10	67405	99044	31640	32	21
TcBrA4_Chr10	112680	294683	182004	182	90
TcBrA4_Chr10	314454	353375	38922	39	20
TcBrA4_Chr10	353911	559444	205534	206	93
TcBrA4_Chr10	578764	648800	70037	70	36
TcBrA4_Chr10	658865	701922	43058	43	25
TcBrA4_Chr10	728903	778262	49360	49	27
TcBrA4_Chr10	800044	826462	26419	26	13
TcBrA4_Chr10	827181	1059079	231899	232	98
TcBrA4_Chr10	1072906	1097740	24835	25	16
TcBrA4_Chr14	5286	106871	101586	102	41
TcBrA4_Chr14	132441	281512	149072	149	63
TcBrA4_Chr14	294765	649416	354652	355	136
TcBrA4_Chr14	681758	709695	27938	28	12
TcBrA4_Chr14	733384	924466	191083	191	91
TcBrA4_Chr14	926821	975858	49038	49	21
TcBrA4_Chr15	0	211277	211278	211	117

TcBrA4_Chr15	212148	269697	57550	58	34
TcBrA4_Chr15	270543	548367	277825	278	122
TcBrA4_Chr15	548890	627546	78657	79	38
TcBrA4_Chr15	640696	687517	46822	47	25
TcBrA4_Chr15	698519	762770	64252	64	38
TcBrA4_Chr15	776007	880721	104715	105	54
TcBrA4_Chr15	902878	913915	11038	11	9
TcBrA4_Chr15	934171	968793	34623	35	15
TcBrA4_Chr16	0	4154	4155	4	1
TcBrA4_Chr16	4744	22443	17700	18	7
TcBrA4_Chr16	23016	406961	383946	384	173
TcBrA4_Chr16	435478	509041	73564	74	35
TcBrA4_Chr16	509577	781882	272306	272	99
TcBrA4_Chr16	796004	807841	11838	12	7
TcBrA4_Chr16	813037	872460	59424	59	27
TcBrA4_Chr16	888952	892867	3916	4	0
TcBrA4_Chr16	895042	920255	25214	25	12
TcBrA4_Chr16	920864	927191	6328	6	3
TcBrA4_Chr17	0	1	2		0
TcBrA4_Chr17	4170	8806	4637	5	3
TcBrA4_Chr17	10499	22179	11681	12	6
TcBrA4_Chr17	23013	25772	2760	3	1
TcBrA4_Chr17	26479	75247	48769	49	19
TcBrA4_Chr17	76671	113183	36513	37	11
TcBrA4_Chr17	113723	127137	13415	13	6
TcBrA4_Chr17	127685	318631	190947	191	86
TcBrA4_Chr17	325985	423958	97974	98	45
TcBrA4_Chr17	429799	491320	61522	62	31
TcBrA4_Chr17	491901	653757	161857	162	73
TcBrA4_Chr17	661670	669019	7350	7	1
TcBrA4_Chr17	681901	711521	29621	30	9
TcBrA4_Chr17	719157	766648	47492	47	28
TcBrA4_Chr17	778442	805516	27075	27	10
TcBrA4_Chr17	829067	861762	32696	33	12
TcBrA4_Chr17	874317	895557	21241	21	9
TcBrA4_Chr17	903072	914771	11700	12	8
TcBrA4_Chr18	0	16239	16240	16	7
TcBrA4_Chr18	23884	28159	4276	4	4
TcBrA4_Chr18	33545	88010	54466	54	26
TcBrA4_Chr18	113335	191423	78089	78	37
TcBrA4_Chr18	208205	232858	24654	25	12
TcBrA4_Chr18	237898	242377	4480	4	2
TcBrA4_Chr18	244780	297784	53005	53	24
TcBrA4_Chr18	324350	341065	16716	17	10
TcBrA4_Chr18	345588	474833	129246	129	59
TcBrA4_Chr18	485280	558819	73540	74	41
TcBrA4_Chr18	566816	751918	185103	185	90
TcBrA4_Chr18	753747	791688	37942	38	18
TcBrA4_Chr18	807481	909794	102314	102	53
TcBrA4_Chr22	0	1	2		0
TcBrA4_Chr22	14576	245134	230559	231	107
TcBrA4_Chr22	255990	350984	94995	95	42
TcBrA4_Chr22	375044	430969	55926	56	26
TcBrA4_Chr22	431753	437435	5683	6	4
TcBrA4_Chr22	441913	451832	9920	10	7
TcBrA4_Chr22	452874	485773	32900	33	16
TcBrA4_Chr22	491056	644943	153888	154	71

TcBrA4_Chr22	645547	681663	36117	36	22
TcBrA4_Chr22	684883	694012	9130	9	3
TcBrA4_Chr22	696735	705570	8836	9	6
TcBrA4_Chr22	709079	715560	6482	6	3
TcBrA4_Chr22	719650	754999	35350	35	10
TcBrA4_Chr22	755594	759468	3875	4	2
TcBrA4_Chr22	760009	784345	24337	24	9
TcBrA4_Chr22	785136	800083	14948	15	7
TcBrA4_Chr22	812640	815970	3331	3	5
TcBrA4_Chr23	638	94021	93384	93	37
TcBrA4_Chr23	97519	105784	8266	8	2
TcBrA4_Chr23	108846	210238	101393	101	49
TcBrA4_Chr23	213179	221377	8199	8	5
TcBrA4_Chr23	223036	227769	4734	5	2
TcBrA4_Chr23	230591	249917	19327	19	11
TcBrA4_Chr23	250504	363594	113091	113	44
TcBrA4_Chr23	365515	393431	27917	28	11
TcBrA4_Chr23	394083	417849	23767	24	13
TcBrA4_Chr23	418373	487641	69269	69	32
TcBrA4_Chr23	492191	511438	19248	19	12
TcBrA4_Chr23	517321	564429	47109	47	23
TcBrA4_Chr24	0	1	2		0
TcBrA4_Chr24	23760	33288	9529	10	5
TcBrA4_Chr24	34318	37068	2751	3	3
TcBrA4_Chr24	37618	47070	9453	9	2
TcBrA4_Chr24	50804	67448	16645	17	10
TcBrA4_Chr24	86457	124991	38535	39	18
TcBrA4_Chr24	134780	146275	11496	11	5
TcBrA4_Chr24	170425	195283	24859	25	11
TcBrA4_Chr24	203610	228716	25107	25	12
TcBrA4_Chr24	229695	238181	8487	8	3
TcBrA4_Chr24	240319	260990	20672	21	7
TcBrA4_Chr24	262230	287773	25544	26	9
TcBrA4_Chr24	305745	319460	13716	14	3
TcBrA4_Chr24	320363	337424	17062	17	3
TcBrA4_Chr24	342562	475627	133066	133	37
TcBrA4_Chr24	486752	551931	65180	65	24
TcBrA4_Chr24	552520	559726	7207	7	5
TcBrA4_Chr24	562046	572888	10843	11	5
TcBrA4_Chr24	573461	578361	4901	5	5
TcBrA4_Chr24	583062	628752	45691	46	19
TcBrA4_Chr24	668887	716268	47382	47	19
TcBrA4_Chr24	732505	756600	24096	24	7
TcBrA4_Chr24	758593	778187	19595	20	9
TcBrA4_Chr28	7399	22811	15413	15	5
TcBrA4_Chr28	23684	41504	17821	18	12
TcBrA4_Chr28	42654	44874	2221	2	2
TcBrA4_Chr28	45739	69385	23647	24	8
TcBrA4_Chr28	69995	74787	4793	5	2
TcBrA4_Chr28	131363	150172	18810	19	9
TcBrA4_Chr28	158171	220029	61859	62	25
TcBrA4_Chr28	236813	344971	108159	108	50
TcBrA4_Chr28	361018	479026	118009	118	41
TcBrA4_Chr28	490772	504353	13582	14	10
TcBrA4_Chr28	529045	559209	30165	30	19
TcBrA4_Chr28	560648	631083	70436	70	28
TcBrA4_Chr28	641735	675098	33364	33	10

TcBrA4_Chr28	687404	704825	17422	17	6
TcBrA4_Chr28	711609	711759	151		1

Mean length (kb) = 64,24
Min length (kb) = 1
Max length (kb) = 384

Name	5’→ 3’ sequence	Size (bp)
FD_Rv_loop5_1	GTACTGAATACTAGAGTGGATCCAGAG	27
FD_Rv_loop5_3	CTCCTGGTTTTGTTGCAGCAATTATG	26
FD_Rv_loop5_1_CTL	CTTCATCGTCTTTCTTGTCTCTCCAAA	27
FD_Fw_loop11_2	AGTTCACTTTCTATAAGACCCGTC	24
FD_Fw_loop11_4	CTCAATCTTCTCGGCAATGGTG	22
FD_Fw_loop11_2_CTL	CACTATATACACAAAAGATGGAGAAATCCT	30
