# Supplementary material for: Genome-wide chromatin interaction map for Trypanosoma cruzi
Source: Nat Microbiol. 2023 Oct 12;8(11):2103–14. doi: 10.1038/s41564-023-01483-y (PMC10627812; doi:10.1038/s41564-023-01483-y)
Supplement: Supplementary file 2 — Reporting Summary [file 41564_2023_1483_MOESM2_ESM.pdf]

nature portfolio

Corresponding author(s): Carlos Robello

Last updated by author(s): Aug 16, 2023

Reporting Summary

Nature Portfolio wishes to improve the reproducibility of the work that we publish. This form provides structure for consistency and transparency in reporting. For further information on Nature Portfolio policies, see our Editorial Policies and the Editorial Policy Checklist.

Statistics

For all statistical analyses, confirm that the following items are present in the figure legend, table legend, main text, or Methods section.

n/a	Confirmed	
☐	☒	The exact sample size (*n*) for each experimental group/condition, given as a discrete number and unit of measurement
☐	☒	A statement on whether measurements were taken from distinct samples or whether the same sample was measured repeatedly
☐	☒	The statistical test(s) used AND whether they are one- or two-sided<br>*Only common tests should be described solely by name; describe more complex techniques in the Methods section.*
☒	☐	A description of all covariates tested
☐	☒	A description of any assumptions or corrections, such as tests of normality and adjustment for multiple comparisons
☐	☒	A full description of the statistical parameters including central tendency (e.g. means) or other basic estimates (e.g. regression coefficient) AND variation (e.g. standard deviation) or associated estimates of uncertainty (e.g. confidence intervals)
☐	☒	For null hypothesis testing, the test statistic (e.g. *F*, *t*, *r*) with confidence intervals, effect sizes, degrees of freedom and *P* value noted<br>*Give P values as exact values whenever suitable.*
☒	☐	For Bayesian analysis, information on the choice of priors and Markov chain Monte Carlo settings
☒	☐	For hierarchical and complex designs, identification of the appropriate level for tests and full reporting of outcomes
☐	☒	Estimates of effect sizes (e.g. Cohen's *d*, Pearson's *r*), indicating how they were calculated

Our web collection on statistics for biologists contains articles on many of the points above.

Software and code

Policy information about availability of computer code

Data collection	Raw-signal data for the methylation datasets were collected as multi-FAST5 files using MinKNOW Core v3.6.5
Data analysis	Oxford Nanopore Technologies fast5 API software (ont_fast5_api v3.1.6)<br>Guppy v3.6.0 (ONT, dna_r9.4.1_450bps_modbases_dam-dcm-cpg_hac base-calling profile)<br>DeepMod v0.1.3 (10.1038/s41467-019-10168-2)<br>BEDTools v2.27.1 (10.1093/bioinformatics/btq033)<br>fasterq-dump v3.0.0 (https://github.com/ncbi/sra-tools)<br>FastQC v0.11.9 (https://www.bioinformatics.babraham.ac.uk/projects/fastqc/)<br>Cutadapt v2.7 (10.14806/ej.17.1.200)<br>Bowtie v1.3.1 (10.1186/gb-2009-10-3-r25)<br>Picard tools v2.25.5 (https://broadinstitute.github.io/picard/)<br>Nucleosome Dynamics v1.0 (10.1093/nar/gkz759)<br>Hisat2 v2.1.0 (10.1038/nmeth.3317)<br>SAMtools v1.16.1 (10.1093/bioinformatics/btp352)<br>Salmon v1.5.1 (10.1038/nmeth.4197)<br>DESeq2 v1.38.3 (10.1186/s13059-014-0550-8)<br>mousetrap v3.2.1 (https://github.com/pascalkieslich/mousetrap)<br>UTRme v1.1.0 (10.3389/fgene.2018.00671)<br>MACS2 v2.2.7.1 (10.1186/gb-2008-9-9-r137)<br>HiC-Pro v3.1.0 (10.1186/s13059-015-0831-x)<br>TADtools (10.1093/bioinformatics/btw368)

April 2023

HiCExplorer v3.7.2 (10.1038/s41467-017-02525-w)
FAN-C v0.9.26b2 (10.1186/s13059-020-02215-9)
pyGenomeTracks v3.7 (10.1093/bioinformatics/btaa692)
R v4.1.3 (https://www.r-project.org/)
GIMP 2.10.32 (https://www.gimp.org/)

For manuscripts utilizing custom algorithms or software that are central to the research but not yet described in published literature, software must be made available to editors and reviewers. We strongly encourage code deposition in a community repository (e.g. GitHub). See the Nature Portfolio guidelines for submitting code & software for further information.

Data

Policy information about availability of data

All manuscripts must include a data availability statement. This statement should provide the following information, where applicable:

- Accession codes, unique identifiers, or web links for publicly available datasets
- A description of any restrictions on data availability
- For clinical datasets or third party data, please ensure that the statement adheres to our policy

Illumina and Oxford Nanopore Technologies sequencing datasets generated for this study were deposited at NCBI: RNA-seq (BioProject ID PRJNA850400) and whole genome DNA sequencing (BioProject ID PRJNA935260).

Trypanosoma cruzi Brazil A4 Hi-C and RNA-seq data are publicly available at NCBI under the accession numbers SRR11803985 and SRR12792489 (doi: 10.1371/journal.ppat.1009254). RNA-seq reads from different subcellular compartments (doi: 10.3389/fcell.2017.00008) are available at NCBI under accession numbers SRR4232036-SRR4232038. MNase-seq datasets (doi: 10.1371/journal.ppat.1009272) are publicly available at NCBI (accession numbers: SRR12710803 - SRR12710808).

T. brucei Hi-C and RNA-seq datasets (doi: 10.1038/s41564-020-00833-4 and doi:10.1038/s41586-018-0619-8) are available at NCBI SRA (accession numbers: ERR3712002, ERR3712009, SRR7721317, SRR7721318, and SRR5809498 - SRR5809500). RBP1 Chip-Seq reads (doi: 10.1016/j.celrep.2021.110221) are available under SRR9022833, SRR9022834, SRR13260277, SRR13260279 accession numbers at NCBI.

Genome FASTA and GFF files were retrieved from TritrypDB (https://tritrypdb.org/tritrypdb/app)

Research involving human participants, their data, or biological material

Policy information about studies with human participants or human data. See also policy information about sex, gender (identity/presentation), and sexual orientation and race, ethnicity and racism.

Reporting on sex and gender	n/a
Reporting on race, ethnicity, or other socially relevant groupings	n/a
Population characteristics	n/a
Recruitment	n/a
Ethics oversight	n/a

Note that full information on the approval of the study protocol must also be provided in the manuscript.

Field-specific reporting

Please select the one below that is the best fit for your research. If you are not sure, read the appropriate sections before making your selection.

☒ Life sciences ☐ Behavioural & social sciences ☐ Ecological, evolutionary & environmental sciences

For a reference copy of the document with all sections, see nature.com/documents/nr-reporting-summary-flat.pdf

Life sciences study design

All studies must disclose on these points even when the disclosure is negative.

Sample size	We do not need to determine the sample size in this study.
Data exclusions	The DNA methylation analysis was performed in the genomic positions with >5 read sequencing coverage. RNA-seq mapped reads with a mapping quality score (MapQ) <10 were discarded. In the RNA low coverage regions analysis, all regions of the genome with zero coverage with <500 bp length were filtered out. DNA sequences not assembled into chromosomes (small contigs) were not considered for the analyses.
Replication	All attempts at replication were successful. RNA-seq and whole genome DNA sequencing were carried out in duplicates. 3C experiments were

Replication	performed in two independent biological replicates.
Randomization	No randomization was applied.
Blinding	DNA methylation analysis was done in a blinded fashion.

Reporting for specific materials, systems and methods

We require information from authors about some types of materials, experimental systems and methods used in many studies. Here, indicate whether each material, system or method listed is relevant to your study. If you are not sure if a list item applies to your research, read the appropriate section before selecting a response.

Materials & experimental systems

n/a	Involved in the study
☒	☐ Antibodies
☐	☒ Eukaryotic cell lines
☒	☐ Palaeontology and archaeology
☒	☐ Animals and other organisms
☒	☐ Clinical data
☒	☐ Dual use research of concern
☒	☐ Plants

Methods

n/a	Involved in the study
☒	☐ ChIP-seq
☒	☐ Flow cytometry
☒	☐ MRI-based neuroimaging

Eukaryotic cell lines

Policy information about cell lines and Sex and Gender in Research

Cell line source(s)	Trypanosoma cruzi Dm28c corresponds to Dm28c2018 clone, obtained in the laboratory of authors, and used as reference for the T. cruzi genome in TriTrypdb. Vero cells are an African Green Monkey Kidney Cell Line, and is the most common used cell line for propagation of Trypanosoma cruzi amastigotes and trypomastigotes.
Authentication	RNA-seq and Genomic DNA sequencing.
Mycoplasma contamination	Parasites and Vero cells were not tested for Mycoplasma.
Commonly misidentified lines (See ICLAC register)	No commonly misidentified cell lines were used in this study.
