# Supplementary figures and images for: Genome-wide chromatin interaction map for Trypanosoma cruzi
Source: Nat Microbiol. 2023 Oct 12;8(11):2103–14. doi: 10.1038/s41564-023-01483-y (PMC10627812; doi:10.1038/s41564-023-01483-y)

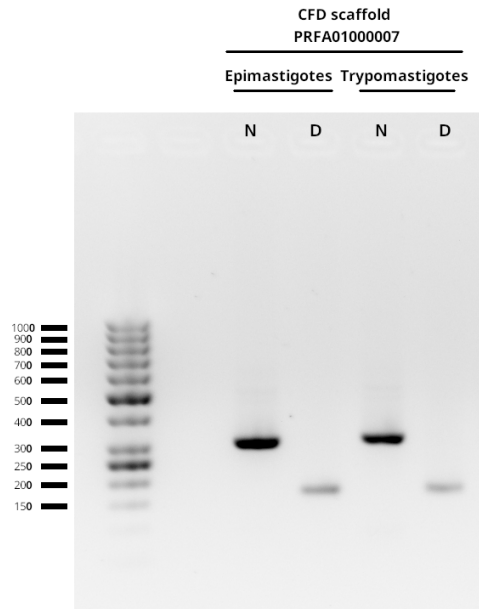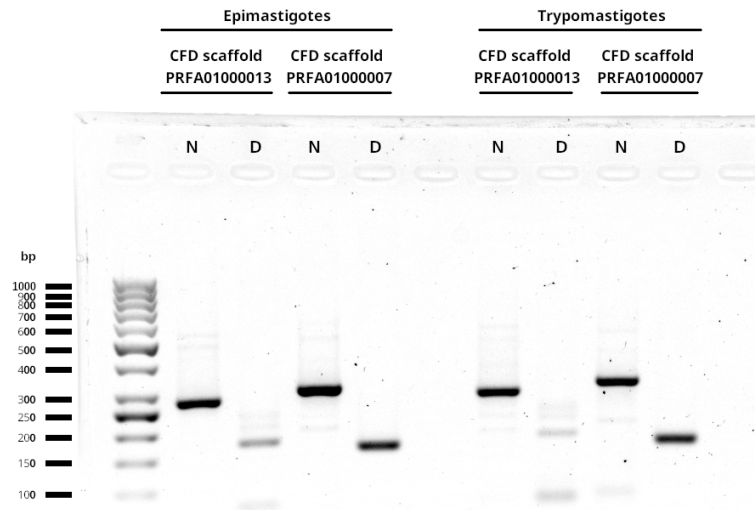

Supplement: Supplementary file 3 — Statistical source data and agarose gel. [file 41564_2023_1483_MOESM3_ESM.zip › uncropped_agarose_gels.pdf]
